# Supplementary material for: Transcriptomic and functional analysis of ANGPTL4 overexpression in pancreatic cancer nominates targets that reverse chemoresistance
Source: BMC Cancer. 2023 Jun 8;23:524. doi: 10.1186/s12885-023-11010-1 (PMC10251551; doi:10.1186/s12885-023-11010-1)

Supplemental Figure S1: a) Kaplan-Meier curve of Recurrence/Disease Free Survival (RFS) using GEPIA PPAD dataset. Logrank p=0.047 **b)** Protein abundance of ANGPTL4 in cell lysate and supernatant as measured by ALPHAlisa assay for normalization by cell input. MP2_ANGPTL4_OE vs MP2_ANPGTL4_KD p=0.0075 and 0.0018 (**) for lysate and supernatant. MP2_ANGPTL4_OE vs MP2 p=0.0055 (**) for lysate. **c)** Heatmap of RNA-seq data for 1198 DEG from MP2_ANGPTL4_OE vs MP2_ANGPTL4_KD with thresholds: baseMean>10 padj<0.05, and log_2 fold change +/-0.7. **d)** Kaplan-Meier plot for overall survival using TCGA-PAAD data. Patients with top (red) and bottom (blue) 25% average *ANGPTL4* gene expression and *APOL1* plus *ITGB4* gene expression (p=0.015, log-rank test).

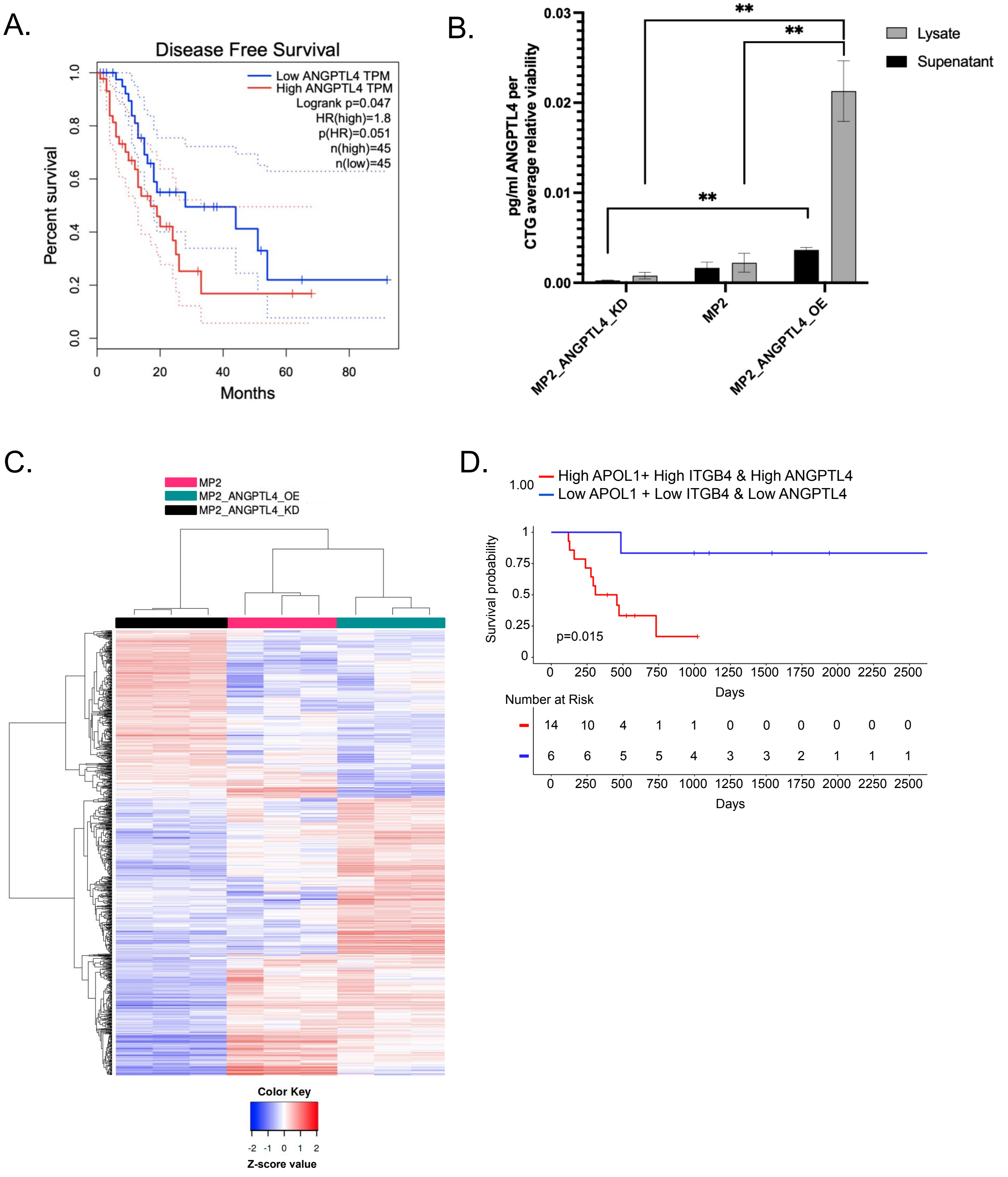

Supplement: Supplementary file 1 — Additional file 1: Figure S1.pdf [file 12885_2023_11010_MOESM1_ESM.pdf]
