# Supplementary material for: Transcriptomic and functional analysis of ANGPTL4 overexpression in pancreatic cancer nominates targets that reverse chemoresistance
Source: BMC Cancer. 2023 Jun 8;23:524. doi: 10.1186/s12885-023-11010-1 (PMC10251551; doi:10.1186/s12885-023-11010-1)

Supplemental Figure 2: **a)** ITGB4 expression measured by qPCR and normalized by the housekeeping gene *ACTB*. All data points are plotted relative to the ANGPTL4_OE line. **b)** *APOL1* expression measured by qPCR and normalized by the housekeeping gene *ACTB*. All data points are plotted relative to the ANGPTL4_OE line. **c)** Cell viability over time normalized to control MP2 cells (lt. pink) at time 0 for MP2_NTC (pink), MP2_ANGPTL4_OE+DsiRNA_NTC (turquoise), MP2_ANGPTL4_OE_ITGB4_KD (lavender), and MP2_ANGPTL4_OE_APOL1_KD (purple).

A.

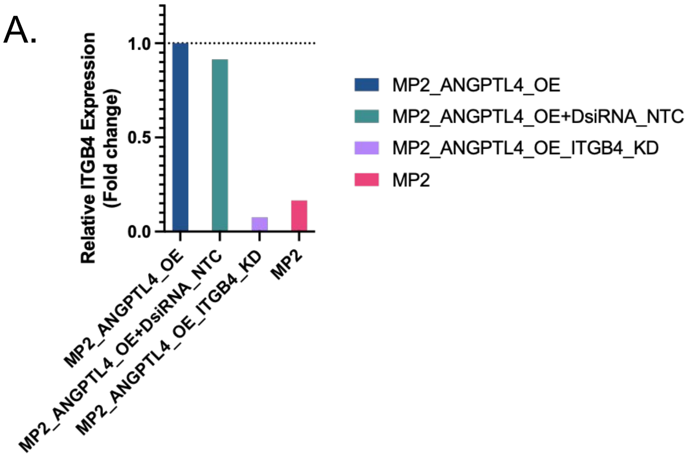

B.

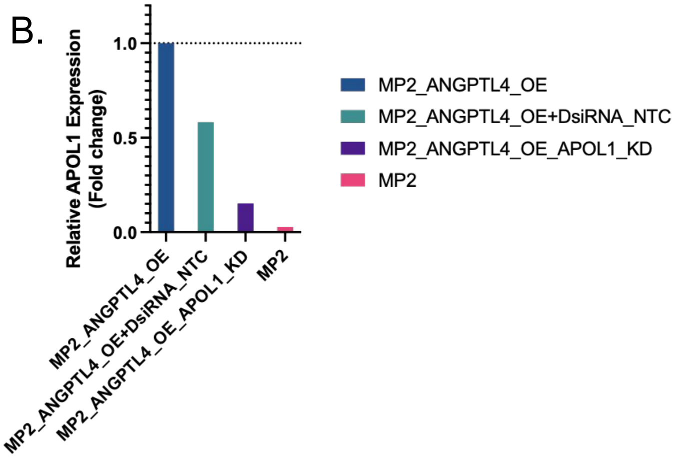

C.

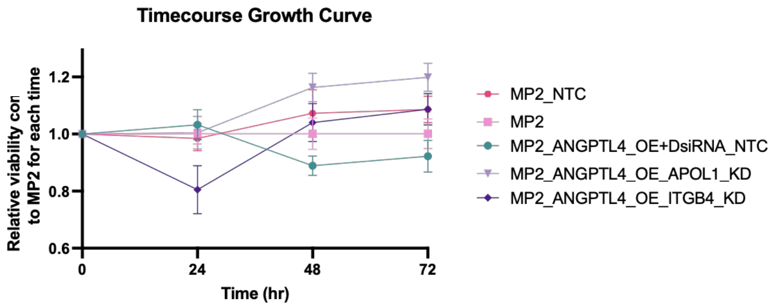

Supplement: Supplementary file 2 — Additional file 2: Figure S2.pdf [file 12885_2023_11010_MOESM2_ESM.pdf]
