# Supplementary material for: Transcriptomic and functional analysis of ANGPTL4 overexpression in pancreatic cancer nominates targets that reverse chemoresistance
Source: BMC Cancer. 2023 Jun 8;23:524. doi: 10.1186/s12885-023-11010-1 (PMC10251551; doi:10.1186/s12885-023-11010-1)
Supplement: Supplementary file 3 — Additional file 3: Table S1.txt [file 12885_2023_11010_MOESM3_ESM.pdf]

Supplementary Table 1: P-values associated with qPCR and viability data

Figure Association	Experimental Measure	Comparison	P-value	Signifigance Flags
1B	qPCR ANGPTL4 Fold Change	MP2_ANGPTL4_KD vs MP2	<0.0001	****
1B	qPCR ANGPTL4 Fold Change	MP2_ANGPTL4_KD vs MP2_ANGPTL4_OE	<0.0001	****
1B	qPCR ANGPTL4 Fold Change	MP2_ANGPTL4_OE vs MP2	<0.0001	****
2A	Relative Viability	0nM Gemcitabine MP2_NTC vs 5nM Gemcitabine MP2_ANGPTL4_KD	0.0007	***
2A	Relative Viability	0nM Gemcitabine MP2_NTC vs 12.5nM Gemcitabine MP2_ANGPTL4_KD	<0.0001	****
2A	Relative Viability	0nM Gemcitabine MP2_NTC vs 5nM Gemcitabine MP2_ANGPTL4_OE	<0.0001	****
2A	Relative Viability	0nM Gemcitabine MP2_NTC vs 12.5nM Gemcitabine MP2_ANGPTL4_OE	0.0021	**
2A	Relative Viability	5nM Gemcitabine MP2_ANGPTL4_KD vs 5nM Gemcitabine MP2_ANGPTL4_OE	0.0005	***
2A	Relative Viability	12.5nM Gemcitabine MP2_ANGPTL4_KD vs 12.5nM Gemcitabine MP2_ANGPTL4_OE	0.0005	***
3C	qPCR relative ANGPTL4 Expression-Fold Change	MP2 vs MP2_ANGPTL4_OE_DsiRNA_NTC	<0.0001	****
3C	qPCR relative ANGPTL4 Expression-Fold Change	MP2_ANGPTL4_OE_DsiRNA_NTC vs MP2_ANGPTL4_OE_APOL1_KD	<0.0001	****
3C	qPCR relative ANGPTL4 Expression-Fold Change	MP2_ANGPTL4_OE_DsiRNA_NTC vs MP2_ANGPTL4_OE_ITGB4_KD	<0.0001	****
3D	Relative Viability	5nM Gemcitabine MP2_ANGPTL4_KD vs 5nM Gemcitabine MP2_ANGPTL4_OE_DsiRNA_NTC	<0.0001	****
3D	Relative Viability	5nM Gemcitabine MP2_ANGPTL4_OE_DsiRNA_NTC vs 5nM MP2_ANGPTL4_OE_APOL1_KD	0.0002	***
3D	Relative Viability	5nM Gemcitabine MP2_ANGPTL4_OE_DsiRNA_NTC vs 5nM MP2_ANGPTL4_OE_ITGB4_KD	<0.0001	****
3D	Relative Viability	12.5nM Gemcitabine MP2_ANGPTL4_KD vs 12.5nM Gemcitabine MP2_ANGPTL4_OE_DsiRNA_NTC	<0.0001	****
3D	Relative Viability	12.5nM Gemcitabine MP2_ANGPTL4_OE_DsiRNA_NTC vs 12.5nM MP2_ANGPTL4_OE_APOL1_KD	<0.0001	****
3D	Relative Viability	12.5nM Gemcitabine MP2_ANGPTL4_OE_DsiRNA_NTC vs 12.5nM MP2_ANGPTL4_OE_ITGB4_KD	<0.0001	****
4C	t1/2 gap	MP2_NTC vs MP2_ANGPTL4_OE_DsiRNA_NTC	0.0064	**
4C	t1/2 gap	MP2_ANGPTL4_OE_DsiRNA_NTC vs 12.5nM MP2_ANGPTL4_OE_APOL1_KD	0.0057	**
4C	t1/2 gap	MP2_ANGPTL4_OE_DsiRNA_NTC vs 12.5nM MP2_ANGPTL4_OE_ITGB4_KD	0.0169	**
