# Supplementary material for: Transcriptomic and functional analysis of ANGPTL4 overexpression in pancreatic cancer nominates targets that reverse chemoresistance
Source: BMC Cancer. 2023 Jun 8;23:524. doi: 10.1186/s12885-023-11010-1 (PMC10251551; doi:10.1186/s12885-023-11010-1)
Supplement: Supplementary file 4 — Additional file 4: Table S2.txt [file 12885_2023_11010_MOESM4_ESM.pdf]

Supplementary Table 2: DEG from MP2_ANGPTL4_OE vs MP2_ANGPTL4_KD analysis with or without gemcitabine treatment

Ensembl Gene	HGNC Symbol	baseMean	log2 FoldChange	lfcSE	stat	pvalue	padj	Gemcitabine Treatment
ENSG00000125398	SOX9	339.357	2.209	0.105	21.087	1.05E-98	1.41E-94	no
ENSG00000159200	RCAN1	161.447	2.928	0.145	20.151	2.64E-90	1.76E-86	no
ENSG00000177606	JUN	442.895	1.465	0.077	18.935	5.85E-80	2.60E-76	no
ENSG00000137831	UACA	595.936	1.675	0.089	18.887	1.47E-79	4.89E-76	no
ENSG00000023445	BIRC3	139.420	2.291	0.125	18.399	1.35E-75	3.59E-72	no
ENSG00000127603	MACF1	714.172	1.464	0.081	18.182	7.14E-74	1.59E-70	no
ENSG00000119917	IFIT3	91.516	3.099	0.171	18.151	1.26E-73	2.39E-70	no
ENSG00000163661	PTX3	49.016	4.081	0.235	17.360	1.65E-67	2.74E-64	no
ENSG00000118515	SGK1	148.656	2.271	0.133	17.110	1.25E-65	1.86E-62	no
ENSG00000172137	CALB2	70.722	2.868	0.178	16.154	1.07E-58	1.42E-55	no
ENSG00000119922	IFIT2	120.866	2.743	0.172	15.991	1.47E-57	1.78E-54	no
ENSG00000175745	NR2F1	439.988	2.109	0.132	15.946	3.02E-57	3.35E-54	no
ENSG00000075223	SEMA3C	148.607	2.454	0.155	15.874	9.65E-57	9.89E-54	no
ENSG00000156453	PCDH1	96.811	3.564	0.225	15.853	1.33E-56	1.27E-53	no
ENSG00000154263	ABCA10	36.405	4.635	0.293	15.830	1.92E-56	1.71E-53	no
ENSG00000105810	CDK6	198.946	2.972	0.189	15.756	6.28E-56	5.23E-53	no
ENSG00000100852	ARHGAP5	343.284	1.631	0.104	15.713	1.22E-55	9.59E-53	no
ENSG00000080824	HSP90AA1	5750.170	-1.002	0.064	-15.698	1.55E-55	1.15E-52	no
ENSG00000075711	DLG1	303.613	1.565	0.100	15.624	5.00E-55	3.51E-52	no
ENSG00000041353	RAB27B	130.622	2.479	0.160	15.516	2.72E-54	1.81E-51	no
ENSG00000196141	SPATS2L	392.103	1.612	0.104	15.487	4.26E-54	2.70E-51	no
ENSG00000137962	ARHGAP29	523.492	1.529	0.099	15.412	1.36E-53	8.22E-51	no
ENSG00000139318	DUSP6	145.802	-2.069	0.137	-15.123	1.14E-51	6.60E-49	no
ENSG00000141682	PMAIP1	96.891	2.196	0.150	14.634	1.69E-48	9.40E-46	no
ENSG00000116717	GADD45A	177.352	1.472	0.101	14.596	2.97E-48	1.58E-45	no
ENSG00000117592	PRDX6	357.748	-1.140	0.079	-14.376	7.35E-47	3.77E-44	no
ENSG00000140105	WARS1	362.491	1.336	0.093	14.315	1.77E-46	8.71E-44	no
ENSG00000187608	ISG15	103.432	2.516	0.177	14.237	5.38E-46	2.56E-43	no
ENSG00000163735	CXCL5	57.101	2.698	0.192	14.021	1.16E-44	5.33E-42	no
ENSG00000163347	CLDN1	81.998	2.309	0.167	13.792	2.86E-43	1.27E-40	no
ENSG00000135114	OASL	32.690	4.251	0.309	13.763	4.26E-43	1.83E-40	no
ENSG00000146648	EGFR	299.075	1.290	0.094	13.668	1.57E-42	6.54E-40	no
ENSG00000065613	SLK	579.073	1.071	0.078	13.647	2.10E-42	8.47E-40	no
ENSG00000205413	SAMD9	74.455	4.096	0.301	13.602	3.92E-42	1.53E-39	no
ENSG00000138646	HERC5	46.361	3.231	0.238	13.585	4.91E-42	1.87E-39	no
ENSG00000135318	NT5E	187.939	1.904	0.142	13.416	4.85E-41	1.80E-38	no
ENSG00000156510	HKDC1	104.578	-2.599	0.195	-13.316	1.87E-40	6.74E-38	no
ENSG00000189403	HMGB1	444.777	-1.115	0.084	-13.231	5.84E-40	2.05E-37	no
ENSG00000130766	SESN2	178.352	1.821	0.139	13.114	2.73E-39	9.32E-37	no
ENSG00000026508	CD44	874.240	0.971	0.074	13.047	6.63E-39	2.21E-36	no

ENSG00000117724	CENPF	1443.623	-0.830	0.064	-13.042	7.09E-39	2.30E-36	no
ENSG00000163739	CXCL1	39.192	2.837	0.219	12.950	2.36E-38	7.47E-36	no
ENSG00000132470	ITGB4	100.207	2.168	0.168	12.894	4.87E-38	1.51E-35	no
ENSG00000135046	ANXA1	412.100	1.021	0.080	12.834	1.06E-37	3.20E-35	no
ENSG00000170689	HOXB9	64.610	2.509	0.196	12.801	1.61E-37	4.77E-35	no
ENSG00000126777	KTN1	2524.085	0.829	0.066	12.587	2.48E-36	7.19E-34	no
ENSG00000183668	PSG9	29.969	-4.287	0.341	-12.580	2.74E-36	7.76E-34	no
ENSG00000075618	FSCN1	157.715	1.843	0.151	12.229	2.18E-34	6.06E-32	no
ENSG00000075391	RASAL2	115.531	-2.176	0.179	-12.132	7.15E-34	1.94E-31	no
ENSG00000116133	DHCR24	169.768	-1.402	0.116	-12.060	1.72E-33	4.58E-31	no
ENSG00000124942	AHNAK	439.064	0.978	0.081	12.026	2.61E-33	6.81E-31	no
ENSG00000181104	F2R	181.861	-1.524	0.127	-11.957	5.94E-33	1.52E-30	no
ENSG00000142089	IFITM3	481.099	1.448	0.122	11.821	3.05E-32	7.66E-30	no
ENSG00000167772	ANGPTL4	29.969	3.379	0.287	11.785	4.69E-32	1.16E-29	no
ENSG00000130779	CLIP1	493.116	0.993	0.084	11.781	4.90E-32	1.19E-29	no
ENSG00000127947	PTPN12	306.965	0.886	0.076	11.708	1.16E-31	2.77E-29	no
ENSG00000108342	CSF3	19.686	3.067	0.264	11.624	3.10E-31	7.25E-29	no
ENSG00000072571	HMMR	255.950	-1.043	0.090	-11.612	3.59E-31	8.25E-29	no
ENSG00000162366	PDZK1IP1	29.958	3.628	0.314	11.553	7.13E-31	1.61E-28	no
ENSG00000107201	DDX58	51.948	2.570	0.223	11.527	9.64E-31	2.14E-28	no
ENSG00000050344	NFE2L3	71.258	2.078	0.181	11.466	1.95E-30	4.27E-28	no
ENSG00000167522	ANKRD11	909.273	0.815	0.071	11.458	2.14E-30	4.54E-28	no
ENSG00000104332	SFRP1	27.576	3.883	0.339	11.458	2.14E-30	4.54E-28	no
ENSG00000115267	IFIH1	41.210	2.616	0.229	11.412	3.64E-30	7.57E-28	no
ENSG00000166750	SLFN5	65.462	2.155	0.189	11.403	4.03E-30	8.27E-28	no
ENSG00000110047	EHD1	130.784	1.719	0.151	11.366	6.19E-30	1.25E-27	no
ENSG00000067082	KLF6	378.170	0.821	0.073	11.324	9.96E-30	1.98E-27	no
ENSG00000196526	AFAP1	79.723	1.934	0.172	11.221	3.21E-29	6.30E-27	no
ENSG00000154027	AK5	28.914	3.012	0.269	11.185	4.81E-29	9.29E-27	no
ENSG00000123094	RASSF8	172.841	1.636	0.146	11.175	5.43E-29	1.03E-26	no
ENSG00000090861	AARS1	305.041	1.118	0.100	11.134	8.55E-29	1.60E-26	no
ENSG00000105993	DNAJB6	174.925	1.165	0.105	11.065	1.85E-28	3.42E-26	no
ENSG00000175048	ZDHHC14	92.811	1.895	0.172	10.999	3.86E-28	7.05E-26	no
ENSG00000134057	CCNB1	391.313	-1.009	0.092	-10.934	7.97E-28	1.40E-25	no
ENSG00000173193	PARP14	108.206	1.683	0.154	10.934	7.95E-28	1.40E-25	no
ENSG00000075213	SEMA3A	312.088	1.246	0.114	10.935	7.81E-28	1.40E-25	no
ENSG00000156011	PSD3	103.303	1.897	0.174	10.927	8.52E-28	1.47E-25	no
ENSG00000110619	CARS1	339.682	1.313	0.120	10.922	9.08E-28	1.55E-25	no
ENSG00000113810	SMC4	1417.539	-0.760	0.070	-10.883	1.40E-27	2.35E-25	no
ENSG00000070814	TCOF1	240.185	-1.071	0.099	-10.834	2.38E-27	3.97E-25	no
ENSG00000235609	n/a	40.588	-2.284	0.211	-10.808	3.16E-27	5.20E-25	no
ENSG00000117984	CTSD	363.134	1.408	0.130	10.790	3.83E-27	6.23E-25	no
ENSG00000186591	UBE2H	236.755	1.169	0.108	10.783	4.13E-27	6.62E-25	no

ENSG00000123240	OPTN	166.154	1.174	0.110	10.706	9.52E-27	1.51E-24	no
ENSG00000134146	DPH6	49.999	-2.451	0.230	-10.658	1.60E-26	2.50E-24	no
ENSG00000111145	ELK3	117.773	1.374	0.129	10.616	2.50E-26	3.88E-24	no
ENSG00000153827	TRIP12	512.769	0.948	0.089	10.608	2.73E-26	4.17E-24	no
ENSG00000111331	OAS3	42.949	2.509	0.239	10.516	7.27E-26	1.10E-23	no
ENSG00000147251	DOCK11	136.232	1.202	0.115	10.478	1.09E-25	1.63E-23	no
ENSG00000181467	RAP2B	166.948	1.709	0.165	10.390	2.76E-25	4.09E-23	no
ENSG00000137628	DDX60	61.775	2.566	0.247	10.381	3.03E-25	4.38E-23	no
ENSG00000164611	PTTG1	309.045	-0.930	0.090	-10.382	2.99E-25	4.38E-23	no
ENSG00000110888	CAPRIN2	428.918	1.273	0.123	10.352	4.09E-25	5.80E-23	no
ENSG00000167641	PPP1R14A	95.843	-3.141	0.303	-10.353	4.07E-25	5.80E-23	no
ENSG00000100994	PYGB	423.073	1.334	0.129	10.335	4.88E-25	6.84E-23	no
ENSG00000168461	RAB31	170.245	1.246	0.121	10.323	5.54E-25	7.68E-23	no
ENSG00000011422	PLAUR	131.985	2.039	0.198	10.279	8.75E-25	1.20E-22	no
ENSG00000167601	AXL	245.721	1.215	0.118	10.259	1.08E-24	1.46E-22	no
ENSG00000139433	GLTP	82.601	1.494	0.146	10.249	1.20E-24	1.62E-22	no
ENSG00000101457	DNTTIP1	135.358	1.478	0.144	10.238	1.34E-24	1.78E-22	no
ENSG00000154133	ROBO4	29.565	2.573	0.252	10.221	1.60E-24	2.10E-22	no
ENSG00000109321	AREG	454.603	-1.112	0.110	-10.112	4.90E-24	6.40E-22	no
ENSG00000105939	ZC3HAV1	205.340	0.963	0.095	10.111	4.95E-24	6.40E-22	no
ENSG00000170485	NPAS2	54.788	2.582	0.256	10.082	6.64E-24	8.51E-22	no
ENSG00000181381	DDX60L	45.840	2.100	0.209	10.057	8.53E-24	1.08E-21	no
ENSG00000126709	IFI6	41.801	2.331	0.232	10.054	8.85E-24	1.11E-21	no
ENSG00000189060	H1-0	103.915	1.755	0.176	9.980	1.86E-23	2.30E-21	no
ENSG00000144802	NFKBIZ	37.663	2.753	0.276	9.971	2.05E-23	2.50E-21	no
ENSG00000163110	PDLIM5	165.656	1.070	0.108	9.945	2.65E-23	3.21E-21	no
ENSG00000130066	SAT1	369.063	1.008	0.101	9.943	2.71E-23	3.26E-21	no
ENSG00000134138	MEIS2	57.569	-2.671	0.270	-9.906	3.92E-23	4.66E-21	no
ENSG00000155368	DBI	366.890	-0.872	0.089	-9.797	1.16E-22	1.37E-20	no
ENSG00000166510	CCDC68	45.340	2.057	0.210	9.779	1.38E-22	1.62E-20	no
ENSG00000120694	HSPH1	865.135	-0.986	0.102	-9.713	2.66E-22	3.08E-20	no
ENSG00000111057	KRT18	1526.946	0.704	0.073	9.702	2.97E-22	3.41E-20	no
ENSG00000161243	FBXO27	111.710	2.361	0.243	9.699	3.05E-22	3.47E-20	no
ENSG00000159167	STC1	41.811	-2.395	0.247	-9.698	3.07E-22	3.47E-20	no
ENSG00000110330	BIRC2	163.381	1.159	0.120	9.630	5.97E-22	6.68E-20	no
ENSG00000181649	PHLDA2	293.590	1.152	0.120	9.568	1.09E-21	1.21E-19	no
ENSG00000048544	MRPS10	240.680	0.923	0.096	9.566	1.11E-21	1.22E-19	no
ENSG00000110958	PTGES3	515.282	-0.792	0.083	-9.564	1.13E-21	1.24E-19	no
ENSG00000117395	EBNA1BP2	406.782	-0.781	0.082	-9.552	1.27E-21	1.38E-19	no
ENSG00000135842	NIBAN1	91.798	1.652	0.173	9.550	1.29E-21	1.39E-19	no
ENSG00000197451	HNRNPAB	545.680	-0.788	0.083	-9.535	1.50E-21	1.59E-19	no
ENSG00000169908	TM4SF1	499.184	0.818	0.086	9.474	2.70E-21	2.86E-19	no
ENSG00000198959	TGM2	180.267	1.251	0.133	9.412	4.89E-21	5.13E-19	no

ENSG00000116285	ERRFI1	667.530	0.721	0.077	9.371	7.19E-21	7.49E-19	no
ENSG00000138678	GPAT3	78.062	1.677	0.179	9.345	9.19E-21	9.49E-19	no
ENSG00000137804	NUSAP1	309.187	-0.834	0.090	-9.276	1.76E-20	1.81E-18	no
ENSG00000110090	CPT1A	91.930	2.008	0.218	9.220	2.97E-20	3.02E-18	no
ENSG00000164032	H2AZ1	564.878	-1.089	0.118	-9.201	3.55E-20	3.58E-18	no
ENSG00000148180	GSN	97.885	1.658	0.181	9.160	5.17E-20	5.18E-18	no
ENSG00000175471	MCTP1	39.289	3.069	0.335	9.154	5.47E-20	5.44E-18	no
ENSG00000120068	HOXB8	94.497	2.593	0.284	9.119	7.61E-20	7.46E-18	no
ENSG00000106105	GARS1	398.991	0.815	0.091	8.994	2.38E-19	2.30E-17	no
ENSG00000138685	FGF2	45.520	1.640	0.183	8.983	2.63E-19	2.52E-17	no
ENSG00000106366	SERPINE1	445.748	1.718	0.192	8.963	3.16E-19	3.01E-17	no
ENSG00000185745	IFIT1	36.512	2.094	0.234	8.962	3.19E-19	3.02E-17	no
ENSG00000223949	ROR1-AS1	32.242	-2.286	0.255	-8.956	3.38E-19	3.15E-17	no
ENSG00000120075	HOXB5	124.243	1.806	0.203	8.905	5.36E-19	4.96E-17	no
ENSG00000170456	DENND5B	84.317	1.677	0.189	8.895	5.84E-19	5.37E-17	no
ENSG00000164056	SPRY1	56.897	2.121	0.239	8.889	6.16E-19	5.62E-17	no
ENSG00000189223	PAX8-AS1	23.365	2.566	0.289	8.885	6.36E-19	5.77E-17	no
ENSG00000113070	HBEGF	84.188	1.641	0.185	8.873	7.13E-19	6.42E-17	no
ENSG00000103257	SLC7A5	205.448	1.310	0.148	8.862	7.88E-19	7.00E-17	no
ENSG00000101255	TRIB3	93.875	1.442	0.163	8.849	8.83E-19	7.79E-17	no
ENSG00000196154	S100A4	374.271	1.001	0.114	8.800	1.36E-18	1.19E-16	no
ENSG00000133321	PLAAT4	16.122	3.691	0.420	8.791	1.49E-18	1.29E-16	no
ENSG00000048052	HDAC9	42.298	2.008	0.230	8.722	2.73E-18	2.35E-16	no
ENSG00000139734	DIAPH3	165.776	-0.981	0.113	-8.700	3.31E-18	2.83E-16	no
ENSG00000156273	BACH1	36.013	1.897	0.218	8.684	3.83E-18	3.25E-16	no
ENSG00000157259	GATAD1	142.325	1.122	0.130	8.655	4.92E-18	4.12E-16	no
ENSG00000132963	POMP	312.055	-0.839	0.097	-8.624	6.48E-18	5.40E-16	no
ENSG00000115541	HSPE1	436.385	-1.178	0.137	-8.575	9.90E-18	8.14E-16	no
ENSG00000113558	SKP1	372.482	-0.719	0.084	-8.568	1.05E-17	8.62E-16	no
ENSG00000126934	MAP2K2	233.020	1.088	0.128	8.496	1.97E-17	1.58E-15	no
ENSG00000171848	RRM2	195.899	-1.086	0.129	-8.399	4.51E-17	3.58E-15	no
ENSG00000013810	TACC3	177.724	-1.045	0.125	-8.375	5.51E-17	4.34E-15	no
ENSG00000141367	CLTC	450.783	-0.766	0.092	-8.364	6.08E-17	4.77E-15	no
ENSG00000271503	CCL5	10.729	4.276	0.514	8.315	9.16E-17	7.14E-15	no
ENSG00000128965	CHAC1	50.149	1.811	0.218	8.310	9.59E-17	7.43E-15	no
ENSG00000135373	EHF	65.538	1.475	0.178	8.306	9.87E-17	7.60E-15	no
ENSG00000145555	MYO10	222.900	0.894	0.108	8.276	1.27E-16	9.68E-15	no
ENSG00000163840	DTX3L	118.707	1.075	0.131	8.230	1.87E-16	1.42E-14	no
ENSG00000116062	MSH6	248.140	-0.735	0.089	-8.222	2.00E-16	1.50E-14	no
ENSG00000117472	TSPAN1	34.754	1.945	0.238	8.171	3.06E-16	2.29E-14	no
ENSG00000081041	CXCL2	15.145	3.573	0.438	8.167	3.16E-16	2.35E-14	no
ENSG00000109971	HSPA8	626.495	-0.818	0.100	-8.159	3.38E-16	2.50E-14	no
ENSG00000133119	RFC3	119.447	-1.068	0.132	-8.119	4.69E-16	3.45E-14	no

ENSG00000165527	ARF6	204.730	0.824	0.102	8.076	6.69E-16	4.87E-14	no
ENSG00000166710	B2M	978.148	0.832	0.103	8.077	6.66E-16	4.87E-14	no
ENSG00000124145	SDC4	197.324	1.021	0.127	8.054	8.00E-16	5.76E-14	no
ENSG00000143621	ILF2	235.968	-0.918	0.114	-8.027	1.00E-15	7.17E-14	no
ENSG00000169710	FASN	324.998	-0.752	0.094	-8.016	1.09E-15	7.76E-14	no
ENSG00000106546	AHR	34.028	-3.632	0.453	-8.012	1.13E-15	7.98E-14	no
ENSG00000104738	MCM4	265.707	-1.018	0.127	-8.006	1.19E-15	8.36E-14	no
ENSG00000140941	MAP1LC3B	177.454	0.956	0.119	8.001	1.24E-15	8.69E-14	no
ENSG00000187098	MITF	30.326	-2.968	0.371	-7.993	1.32E-15	9.21E-14	no
ENSG00000163762	TM4SF18	46.887	1.691	0.212	7.963	1.68E-15	1.16E-13	no
ENSG00000140403	DNAJA4	13.262	4.215	0.531	7.932	2.16E-15	1.49E-13	no
ENSG00000242195	SRRM1P2	67.808	-1.716	0.217	-7.896	2.89E-15	1.97E-13	no
ENSG00000143476	DTL	84.056	-1.279	0.163	-7.849	4.18E-15	2.84E-13	no
ENSG00000196950	SLC39A10	164.831	1.083	0.139	7.804	5.98E-15	4.02E-13	no
ENSG00000117298	ECE1	369.130	0.896	0.115	7.795	6.42E-15	4.30E-13	no
ENSG00000077782	FGFR1	75.197	-1.199	0.154	-7.791	6.64E-15	4.42E-13	no
ENSG00000087586	AURKA	238.063	-0.809	0.104	-7.745	9.52E-15	6.25E-13	no
ENSG00000204592	HLA-E	133.737	0.926	0.120	7.734	1.04E-14	6.81E-13	no
ENSG00000273706	LHX1	127.982	-1.036	0.134	-7.732	1.06E-14	6.90E-13	no
ENSG00000143815	LBR	116.641	-1.106	0.143	-7.710	1.26E-14	8.14E-13	no
ENSG00000071575	TRIB2	48.093	1.862	0.242	7.695	1.41E-14	9.05E-13	no
ENSG00000180530	NRIP1	64.931	-1.982	0.258	-7.688	1.49E-14	9.51E-13	no
ENSG00000121060	TRIM25	157.347	0.918	0.120	7.670	1.72E-14	1.08E-12	no
ENSG00000197121	PGAP1	33.980	2.308	0.301	7.660	1.86E-14	1.17E-12	no
ENSG00000221869	CEBPD	36.952	2.162	0.283	7.647	2.06E-14	1.29E-12	no
ENSG00000100219	XBP1	99.308	1.090	0.143	7.646	2.08E-14	1.29E-12	no
ENSG00000153815	CMIP	211.814	0.818	0.107	7.642	2.13E-14	1.32E-12	no
ENSG00000111335	OAS2	11.094	3.836	0.503	7.630	2.34E-14	1.44E-12	no
ENSG00000151849	CENPJ	80.286	-1.185	0.155	-7.629	2.37E-14	1.45E-12	no
ENSG00000113368	LMNB1	188.127	-0.956	0.125	-7.621	2.52E-14	1.53E-12	no
ENSG00000116161	CACYBP	236.816	-0.856	0.113	-7.593	3.13E-14	1.90E-12	no
ENSG00000137285	TUBB2B	36.388	3.168	0.418	7.586	3.29E-14	1.97E-12	no
ENSG00000171365	CLCN5	65.549	1.355	0.179	7.571	3.71E-14	2.20E-12	no
ENSG00000175592	FOSL1	173.942	1.125	0.149	7.563	3.95E-14	2.34E-12	no
ENSG00000102189	EEA1	190.571	0.965	0.128	7.544	4.55E-14	2.68E-12	no
ENSG00000123975	CKS2	131.251	-1.027	0.136	-7.540	4.69E-14	2.75E-12	no
ENSG00000164543	STK17A	150.731	0.914	0.121	7.533	4.95E-14	2.89E-12	no
ENSG00000070669	ASNS	152.013	0.948	0.126	7.500	6.40E-14	3.71E-12	no
ENSG00000077152	UBE2T	51.482	-1.947	0.260	-7.497	6.54E-14	3.77E-12	no
ENSG00000002079	MYH16	15.842	2.425	0.324	7.495	6.65E-14	3.80E-12	no
ENSG00000144824	PHLDB2	638.499	0.758	0.101	7.492	6.76E-14	3.85E-12	no
ENSG00000143799	PARP1	330.206	-0.758	0.101	-7.487	7.03E-14	3.98E-12	no
ENSG00000184900	SUMO3	279.482	0.717	0.096	7.453	9.15E-14	5.14E-12	no

ENSG00000074211	PPP2R2C	45.950	-1.432	0.192	-7.439	1.01E-13	5.67E-12	no
ENSG00000163171	CDC42EP3	248.106	-1.010	0.136	-7.436	1.04E-13	5.78E-12	no
ENSG00000101911	PRPS2	87.199	-1.143	0.154	-7.416	1.21E-13	6.70E-12	no
ENSG00000162733	DDR2	96.566	1.247	0.169	7.391	1.45E-13	8.01E-12	no
ENSG00000169429	CXCL8	28.413	3.062	0.415	7.371	1.69E-13	9.22E-12	no
ENSG00000089685	BIRC5	246.593	-0.847	0.115	-7.368	1.74E-13	9.44E-12	no
ENSG00000112984	KIF20A	59.365	-1.578	0.215	-7.357	1.88E-13	1.02E-11	no
ENSG00000142945	KIF2C	103.691	-1.069	0.146	-7.317	2.54E-13	1.36E-11	no
ENSG00000088826	SMOX	40.989	1.423	0.196	7.262	3.82E-13	2.03E-11	no
ENSG00000042493	CAPG	133.883	1.400	0.193	7.246	4.30E-13	2.27E-11	no
ENSG00000109452	INPP4B	21.944	2.739	0.379	7.237	4.57E-13	2.41E-11	no
ENSG00000130589	HELZ2	82.030	1.071	0.148	7.236	4.63E-13	2.43E-11	no
ENSG00000188549	CCDC9B	42.417	1.516	0.210	7.211	5.55E-13	2.89E-11	no
ENSG00000089127	OAS1	10.147	4.471	0.620	7.211	5.55E-13	2.89E-11	no
ENSG00000154134	ROBO3	34.176	1.827	0.253	7.208	5.68E-13	2.94E-11	no
ENSG00000184349	EFNA5	53.554	-1.646	0.228	-7.207	5.72E-13	2.96E-11	no
ENSG00000136108	CKAP2	229.587	-0.804	0.112	-7.204	5.83E-13	3.00E-11	no
ENSG00000105327	BBC3	113.295	1.105	0.154	7.166	7.72E-13	3.91E-11	no
ENSG00000106278	PTPRZ1	19.690	2.598	0.363	7.166	7.70E-13	3.91E-11	no
ENSG00000122034	GTF3A	207.096	-0.747	0.105	-7.128	1.02E-12	5.10E-11	no
ENSG00000018408	WWTR1	340.848	0.707	0.099	7.114	1.12E-12	5.59E-11	no
ENSG00000123066	MED13L	161.992	1.135	0.160	7.101	1.23E-12	6.07E-11	no
ENSG00000144118	RALB	147.436	0.949	0.134	7.102	1.23E-12	6.07E-11	no
ENSG00000051108	HERPUD1	68.204	1.309	0.184	7.097	1.28E-12	6.25E-11	no
ENSG00000132386	SERPINF1	18.415	2.250	0.318	7.081	1.43E-12	6.99E-11	no
ENSG00000100906	NFKBIA	387.539	0.822	0.116	7.058	1.69E-12	8.22E-11	no
ENSG00000136193	SCRN1	139.297	1.176	0.167	7.056	1.71E-12	8.29E-11	no
ENSG00000269190	FBXO17	119.984	1.126	0.160	7.042	1.89E-12	9.12E-11	no
ENSG00000221963	APOL6	42.562	1.499	0.213	7.027	2.11E-12	1.01E-10	no
ENSG00000198707	CEP290	166.470	0.801	0.114	7.028	2.09E-12	1.01E-10	no
ENSG00000180263	FGD6	56.912	1.430	0.203	7.028	2.10E-12	1.01E-10	no
ENSG00000113658	SMAD5	189.942	-0.743	0.106	-7.004	2.49E-12	1.19E-10	no
ENSG00000117868	ESYT2	123.129	0.933	0.133	6.998	2.59E-12	1.23E-10	no
ENSG00000170035	UBE2E3	82.739	1.245	0.178	6.997	2.61E-12	1.23E-10	no
ENSG00000154518	ATP5MC3	458.225	-0.721	0.104	-6.962	3.35E-12	1.56E-10	no
ENSG00000082516	GEMIN5	131.756	-0.750	0.108	-6.955	3.52E-12	1.64E-10	no
ENSG00000204397	CARD16	23.929	-1.958	0.283	-6.922	4.44E-12	2.06E-10	no
ENSG00000184584	STING1	14.626	3.882	0.561	6.917	4.62E-12	2.14E-10	no
ENSG00000151491	EPS8	70.878	2.074	0.300	6.911	4.83E-12	2.22E-10	no
ENSG00000101782	RIOK3	126.725	0.947	0.137	6.907	4.96E-12	2.28E-10	no
ENSG00000171316	CHD7	136.525	1.014	0.147	6.892	5.52E-12	2.53E-10	no
ENSG00000067066	SP100	97.485	0.908	0.132	6.882	5.92E-12	2.70E-10	no
ENSG00000125826	RBCK1	179.947	0.795	0.115	6.880	5.97E-12	2.71E-10	no

ENSG00000198700	IPO9	230.129	-0.755	0.110	-6.874	6.24E-12	2.83E-10	no
ENSG00000141447	OSBPL1A	98.405	0.919	0.134	6.849	7.42E-12	3.34E-10	no
ENSG00000083642	PDS5B	139.989	-0.882	0.129	-6.836	8.12E-12	3.64E-10	no
ENSG00000131495	NDUFA2	138.899	-0.849	0.124	-6.830	8.48E-12	3.78E-10	no
ENSG00000049249	TNFRSF9	14.223	2.432	0.356	6.830	8.49E-12	3.78E-10	no
ENSG00000149485	FADS1	149.671	1.079	0.158	6.826	8.70E-12	3.87E-10	no
ENSG00000171320	ESCO2	52.428	-1.220	0.179	-6.817	9.32E-12	4.11E-10	no
ENSG00000196914	ARHGEF12	155.821	0.874	0.128	6.816	9.36E-12	4.12E-10	no
ENSG00000175582	RAB6A	110.182	0.991	0.145	6.808	9.87E-12	4.33E-10	no
ENSG00000182809	CRIP2	81.013	-1.293	0.190	-6.796	1.07E-11	4.69E-10	no
ENSG00000113048	MRPS27	121.607	-0.932	0.137	-6.795	1.08E-11	4.71E-10	no
ENSG00000117399	CDC20	204.479	-0.706	0.104	-6.786	1.15E-11	4.98E-10	no
ENSG00000118640	VAMP8	41.593	2.253	0.333	6.765	1.34E-11	5.74E-10	no
ENSG00000184743	ATL3	94.323	1.277	0.189	6.758	1.40E-11	5.98E-10	no
ENSG00000133657	ATP13A3	308.027	-0.724	0.107	-6.745	1.53E-11	6.53E-10	no
ENSG00000125740	FOSB	67.021	1.344	0.200	6.732	1.68E-11	7.12E-10	no
ENSG00000230453	ANKRD18B	14.998	2.259	0.336	6.724	1.77E-11	7.50E-10	no
ENSG00000090266	NDUFB2	200.303	-0.803	0.120	-6.671	2.55E-11	1.07E-09	no
ENSG00000134954	ETS1	77.960	1.192	0.179	6.658	2.78E-11	1.16E-09	no
ENSG00000094804	CDC6	77.746	-1.190	0.179	-6.653	2.88E-11	1.20E-09	no
ENSG00000135378	PRRG4	33.113	1.877	0.283	6.638	3.18E-11	1.31E-09	no
ENSG00000112118	MCM3	169.595	-0.779	0.118	-6.631	3.33E-11	1.37E-09	no
ENSG00000043039	BARX2	14.526	2.401	0.363	6.624	3.49E-11	1.43E-09	no
ENSG00000176890	TYMS	127.292	-0.851	0.128	-6.620	3.60E-11	1.47E-09	no
ENSG00000188290	HES4	33.677	1.877	0.284	6.607	3.91E-11	1.59E-09	no
ENSG00000173575	CHD2	161.735	0.801	0.121	6.601	4.08E-11	1.65E-09	no
ENSG00000071539	TRIP13	71.742	-1.045	0.158	-6.594	4.27E-11	1.72E-09	no
ENSG00000087074	PPP1R15A	162.218	0.995	0.151	6.591	4.38E-11	1.76E-09	no
ENSG00000130303	BST2	32.864	2.430	0.369	6.590	4.41E-11	1.77E-09	no
ENSG00000250072	SH3TC2-DT	61.923	1.232	0.187	6.579	4.75E-11	1.89E-09	no
ENSG00000175197	DDIT3	88.801	1.309	0.200	6.559	5.42E-11	2.16E-09	no
ENSG00000122026	RPL21	574.659	-0.814	0.124	-6.557	5.47E-11	2.17E-09	no
ENSG00000023191	RNH1	160.440	0.736	0.112	6.551	5.71E-11	2.26E-09	no
ENSG00000196368	NUDT11	21.822	-2.083	0.318	-6.544	5.97E-11	2.35E-09	no
ENSG00000151748	SAV1	98.316	1.102	0.169	6.529	6.63E-11	2.61E-09	no
ENSG00000112096	SOD2	68.304	1.068	0.164	6.513	7.36E-11	2.89E-09	no
ENSG00000180914	OXTR	12.378	2.738	0.421	6.500	8.05E-11	3.14E-09	no
ENSG00000182919	C11orf54	50.427	1.463	0.225	6.498	8.16E-11	3.18E-09	no
ENSG00000189143	CLDN4	249.231	0.887	0.137	6.490	8.56E-11	3.31E-09	no
ENSG00000184661	CDCA2	114.754	-0.847	0.131	-6.488	8.68E-11	3.35E-09	no
ENSG00000175352	NRIP3	28.439	2.226	0.344	6.471	9.75E-11	3.74E-09	no
ENSG00000134690	CDCA8	123.364	-0.843	0.130	-6.467	9.99E-11	3.82E-09	no
ENSG00000164442	CITED2	43.511	2.443	0.378	6.459	1.06E-10	4.02E-09	no

ENSG00000128272	ATF4	469.272	0.886	0.137	6.452	1.10E-10	4.17E-09	no
ENSG00000115415	STAT1	161.309	0.776	0.120	6.450	1.12E-10	4.22E-09	no
ENSG00000124201	ZNFX1	146.862	0.888	0.138	6.450	1.12E-10	4.22E-09	no
ENSG00000138166	DUSP5	54.294	3.096	0.481	6.433	1.25E-10	4.70E-09	no
ENSG00000112715	VEGFA	117.858	1.010	0.157	6.428	1.29E-10	4.83E-09	no
ENSG00000172183	ISG20	22.432	1.817	0.283	6.422	1.34E-10	5.00E-09	no
ENSG00000126524	SBDS	189.053	0.740	0.115	6.422	1.35E-10	5.01E-09	no
ENSG00000028203	VEZT	121.229	0.862	0.134	6.421	1.36E-10	5.04E-09	no
ENSG00000167703	SLC43A2	19.583	-1.908	0.298	-6.411	1.44E-10	5.33E-09	no
ENSG00000172216	CEBPB	103.452	1.246	0.195	6.404	1.51E-10	5.54E-09	no
ENSG00000104856	RELB	44.676	1.444	0.226	6.385	1.71E-10	6.27E-09	no
ENSG00000110422	HIPK3	68.229	1.242	0.195	6.377	1.81E-10	6.57E-09	no
ENSG00000168610	STAT3	219.845	0.975	0.153	6.373	1.85E-10	6.71E-09	no
ENSG00000023171	GRAMD1B	25.689	-1.691	0.266	-6.363	1.98E-10	7.15E-09	no
ENSG00000163565	IFI16	45.342	2.109	0.333	6.335	2.38E-10	8.53E-09	no
ENSG00000169679	BUB1	150.409	-0.763	0.121	-6.317	2.67E-10	9.56E-09	no
ENSG00000171604	CXXC5	96.837	-1.325	0.210	-6.315	2.70E-10	9.64E-09	no
ENSG00000169230	PRELID1	255.705	-0.710	0.113	-6.312	2.76E-10	9.83E-09	no
ENSG00000214900	LINC01588	19.829	1.878	0.298	6.308	2.83E-10	1.00E-08	no
ENSG00000152422	XRCC4	68.626	-1.048	0.166	-6.293	3.10E-10	1.10E-08	no
ENSG00000253276	CCDC71L	212.122	-1.583	0.252	-6.285	3.29E-10	1.16E-08	no
ENSG00000126787	DLGAP5	162.879	-0.769	0.122	-6.279	3.40E-10	1.19E-08	no
ENSG00000161888	SPC24	32.411	-1.837	0.294	-6.247	4.18E-10	1.45E-08	no
ENSG00000133789	SWAP70	126.302	0.866	0.139	6.248	4.14E-10	1.45E-08	no
ENSG00000145358	DDIT4L	12.735	-2.613	0.419	-6.239	4.42E-10	1.53E-08	no
ENSG00000139618	BRCA2	119.148	-0.822	0.132	-6.233	4.57E-10	1.58E-08	no
ENSG00000186472	PCLO	25.715	-2.169	0.348	-6.233	4.59E-10	1.58E-08	no
ENSG00000169247	SH3TC2	20.910	1.871	0.301	6.213	5.20E-10	1.79E-08	no
ENSG00000198626	RYR2	46.657	-1.731	0.279	-6.209	5.32E-10	1.83E-08	no
ENSG00000224099	n/a	21.881	2.260	0.364	6.202	5.57E-10	1.91E-08	no
ENSG00000166803	PCLAF	201.781	-0.813	0.131	-6.192	5.95E-10	2.02E-08	no
ENSG00000060982	BCAT1	85.424	1.272	0.206	6.185	6.21E-10	2.10E-08	no
ENSG00000183696	UPP1	123.541	1.098	0.178	6.185	6.20E-10	2.10E-08	no
ENSG00000186660	ZFP91	190.988	0.781	0.127	6.161	7.23E-10	2.44E-08	no
ENSG00000135365	PHF21A	93.691	0.891	0.145	6.130	8.78E-10	2.95E-08	no
ENSG00000139437	TCHP	81.959	1.000	0.163	6.119	9.42E-10	3.15E-08	no
ENSG00000116857	TMEM9	77.011	-0.893	0.146	-6.116	9.58E-10	3.20E-08	no
ENSG00000186480	INSIG1	79.028	-1.155	0.189	-6.111	9.92E-10	3.30E-08	no
ENSG00000129534	MIS18BP1	174.401	-0.709	0.116	-6.110	9.94E-10	3.30E-08	no
ENSG00000166432	ZMAT1	25.245	1.671	0.274	6.104	1.04E-09	3.44E-08	no
ENSG00000031698	SARS1	311.467	0.785	0.129	6.077	1.22E-09	4.03E-08	no
ENSG00000169871	TRIM56	68.286	1.147	0.189	6.075	1.24E-09	4.06E-08	no
ENSG00000090615	GOLGA3	139.150	0.713	0.118	6.056	1.40E-09	4.56E-08	no

ENSG00000164463	CREBRF	90.515	0.865	0.143	6.053	1.42E-09	4.62E-08	no
ENSG00000156587	UBE2L6	28.588	1.492	0.247	6.053	1.42E-09	4.63E-08	no
ENSG00000171680	PLEKHG5	44.883	1.432	0.238	6.026	1.68E-09	5.42E-08	no
ENSG00000152253	SPC25	45.282	-1.171	0.195	-6.021	1.74E-09	5.59E-08	no
ENSG00000140350	ANP32A	238.862	-0.727	0.121	-6.018	1.76E-09	5.65E-08	no
ENSG00000198742	SMURF1	46.395	1.355	0.226	6.007	1.89E-09	6.05E-08	no
ENSG00000082641	NFE2L1	109.271	0.844	0.142	5.953	2.64E-09	8.37E-08	no
ENSG00000182568	SATB1	14.531	2.100	0.353	5.941	2.84E-09	8.93E-08	no
ENSG00000091317	CMTM6	169.194	0.886	0.149	5.938	2.89E-09	9.09E-08	no
ENSG00000107438	PDLIM1	80.576	1.385	0.233	5.935	2.94E-09	9.20E-08	no
ENSG00000166801	FAM111A	94.142	1.034	0.174	5.931	3.02E-09	9.44E-08	no
ENSG00000156384	SFR1	26.567	1.447	0.244	5.929	3.04E-09	9.48E-08	no
ENSG00000275342	PRAG1	27.782	2.138	0.361	5.924	3.14E-09	9.75E-08	no
ENSG00000080503	SMARCA2	36.959	1.967	0.332	5.922	3.18E-09	9.82E-08	no
ENSG00000117586	TNFSF4	15.978	-3.074	0.519	-5.922	3.18E-09	9.82E-08	no
ENSG00000171552	BCL2L1	63.262	1.539	0.261	5.904	3.55E-09	1.08E-07	no
ENSG00000138347	MYPN	75.149	-0.871	0.147	-5.904	3.56E-09	1.08E-07	no
ENSG00000107554	DNMBP	51.518	1.329	0.225	5.903	3.58E-09	1.09E-07	no
ENSG00000136051	WASHC4	103.437	0.871	0.148	5.892	3.81E-09	1.15E-07	no
ENSG00000080823	MOK	402.390	0.847	0.144	5.887	3.94E-09	1.19E-07	no
ENSG00000073417	PDE8A	32.483	1.563	0.266	5.879	4.12E-09	1.23E-07	no
ENSG00000173141	MRPL57	100.928	-0.729	0.124	-5.878	4.16E-09	1.24E-07	no
ENSG00000237187	NR2F1-AS1	24.194	1.945	0.331	5.871	4.33E-09	1.29E-07	no
ENSG00000166340	TPP1	99.954	0.948	0.162	5.854	4.80E-09	1.42E-07	no
ENSG00000185022	MAFF	67.239	1.125	0.192	5.853	4.82E-09	1.43E-07	no
ENSG00000143153	ATP1B1	231.955	0.914	0.156	5.851	4.89E-09	1.44E-07	no
ENSG00000075218	GTSE1	76.992	-1.018	0.174	-5.849	4.95E-09	1.46E-07	no
ENSG00000143248	RGS5	301.565	-0.713	0.122	-5.848	4.98E-09	1.46E-07	no
ENSG00000156970	BUB1B	93.113	-0.966	0.165	-5.844	5.09E-09	1.49E-07	no
ENSG00000260027	HOXB7	44.567	1.695	0.290	5.841	5.20E-09	1.52E-07	no
ENSG00000130513	GDF15	369.813	0.814	0.139	5.840	5.23E-09	1.53E-07	no
ENSG00000112983	BRD8	164.067	-0.751	0.129	-5.835	5.38E-09	1.56E-07	no
ENSG00000166548	TK2	42.832	1.166	0.200	5.831	5.51E-09	1.60E-07	no
ENSG00000111358	GTF2H3	95.612	0.766	0.131	5.825	5.71E-09	1.65E-07	no
ENSG00000072274	TFRC	191.448	-0.740	0.127	-5.822	5.82E-09	1.68E-07	no
ENSG00000100342	APOL1	16.947	2.481	0.426	5.818	5.94E-09	1.71E-07	no
ENSG00000120948	TARDBP	89.722	-0.911	0.158	-5.786	7.23E-09	2.07E-07	no
ENSG00000185306	C12orf56	14.160	2.157	0.374	5.775	7.70E-09	2.20E-07	no
ENSG00000065328	MCM10	68.434	-1.062	0.184	-5.772	7.84E-09	2.24E-07	no
ENSG00000074657	ZNF532	24.275	2.083	0.362	5.757	8.54E-09	2.43E-07	no
ENSG00000182095	TNRC18	67.821	1.037	0.180	5.750	8.91E-09	2.52E-07	no
ENSG00000013364	MVP	48.713	1.336	0.232	5.749	9.00E-09	2.53E-07	no
ENSG00000072682	P4HA2	39.119	-1.201	0.209	-5.750	8.95E-09	2.53E-07	no

ENSG00000163507	CIP2A	129.178	-0.758	0.132	-5.746	9.15E-09	2.57E-07	no
ENSG00000175161	CADM2	27.292	1.561	0.272	5.742	9.34E-09	2.62E-07	no
ENSG00000182742	HOXB4	44.562	1.987	0.346	5.742	9.36E-09	2.62E-07	no
ENSG00000113594	LIFR	46.563	1.273	0.222	5.742	9.37E-09	2.62E-07	no
ENSG00000138642	HERC6	13.369	2.158	0.376	5.739	9.55E-09	2.66E-07	no
ENSG00000204388	HSPA1B	44.149	-1.554	0.271	-5.738	9.61E-09	2.67E-07	no
ENSG00000117650	NEK2	85.914	-0.952	0.166	-5.734	9.80E-09	2.71E-07	no
ENSG00000177200	CHD9	146.038	0.711	0.124	5.732	9.92E-09	2.74E-07	no
ENSG00000076382	SPAG5	53.531	-1.150	0.201	-5.729	1.01E-08	2.79E-07	no
ENSG00000176393	RNPEP	80.926	-1.037	0.181	-5.726	1.03E-08	2.84E-07	no
ENSG00000162772	ATF3	50.449	1.624	0.284	5.724	1.04E-08	2.85E-07	no
ENSG00000178397	FAM220A	40.725	1.129	0.198	5.707	1.15E-08	3.15E-07	no
ENSG00000091483	FH	73.370	-1.025	0.180	-5.707	1.15E-08	3.15E-07	no
ENSG00000168003	SLC3A2	202.781	0.805	0.141	5.703	1.18E-08	3.22E-07	no
ENSG00000076685	NT5C2	100.661	1.019	0.179	5.696	1.22E-08	3.33E-07	no
ENSG00000213465	ARL2	82.328	0.892	0.157	5.693	1.25E-08	3.37E-07	no
ENSG00000120885	CLU	188.876	-0.788	0.139	-5.678	1.36E-08	3.66E-07	no
ENSG00000198363	ASPH	457.485	-0.719	0.127	-5.669	1.43E-08	3.82E-07	no
ENSG00000096092	TMEM14A	47.522	-1.341	0.237	-5.651	1.59E-08	4.23E-07	no
ENSG00000254911	SCARNA9	116.034	0.945	0.167	5.645	1.65E-08	4.39E-07	no
ENSG00000130813	SHFL	33.857	1.323	0.235	5.637	1.73E-08	4.57E-07	no
ENSG00000114861	FOXP1	43.371	1.220	0.217	5.629	1.81E-08	4.77E-07	no
ENSG00000006459	KDM7A	20.969	1.685	0.300	5.620	1.91E-08	5.02E-07	no
ENSG00000085832	EPS15	91.952	0.800	0.143	5.606	2.07E-08	5.40E-07	no
ENSG00000142798	HSPG2	37.566	1.306	0.233	5.604	2.09E-08	5.44E-07	no
ENSG00000085662	AKR1B1	93.028	-0.784	0.140	-5.603	2.11E-08	5.48E-07	no
ENSG00000163359	COL6A3	40.723	1.062	0.190	5.588	2.30E-08	5.93E-07	no
ENSG00000177707	NECTIN3	51.121	-1.241	0.222	-5.588	2.30E-08	5.93E-07	no
ENSG00000125618	PAX8	14.817	1.969	0.353	5.584	2.36E-08	6.06E-07	no
ENSG00000249784	SCARNA22	206.496	1.174	0.211	5.573	2.51E-08	6.43E-07	no
ENSG00000131711	MAP1B	180.795	0.989	0.178	5.566	2.60E-08	6.67E-07	no
ENSG00000166833	NAV2	92.301	1.260	0.227	5.561	2.69E-08	6.87E-07	no
ENSG00000073111	MCM2	123.079	-1.070	0.192	-5.557	2.74E-08	6.97E-07	no
ENSG00000121310	ECHDC2	52.107	1.206	0.217	5.555	2.77E-08	7.03E-07	no
ENSG00000067798	NAV3	25.808	-1.669	0.301	-5.547	2.91E-08	7.36E-07	no
ENSG00000101187	SLCO4A1	131.353	0.724	0.130	5.546	2.92E-08	7.37E-07	no
ENSG00000177556	ATOX1	111.648	-0.789	0.142	-5.545	2.94E-08	7.42E-07	no
ENSG00000170776	AKAP13	127.076	0.883	0.159	5.542	2.99E-08	7.52E-07	no
ENSG00000137807	KIF23	136.515	-0.773	0.140	-5.524	3.32E-08	8.33E-07	no
ENSG00000079459	FDFT1	113.034	-0.737	0.133	-5.523	3.33E-08	8.34E-07	no
ENSG00000125864	BFSP1	23.692	1.529	0.277	5.522	3.34E-08	8.36E-07	no
ENSG00000269834	ZNF528-AS1	20.385	1.790	0.325	5.513	3.52E-08	8.79E-07	no
ENSG00000138496	PARP9	33.877	1.232	0.224	5.510	3.58E-08	8.93E-07	no

ENSG00000137812	KNL1	117.039	-0.814	0.149	-5.478	4.30E-08	1.06E-06	no
ENSG00000147854	UHRF2	114.163	0.796	0.145	5.479	4.27E-08	1.06E-06	no
ENSG00000109787	KLF3	92.899	0.790	0.144	5.473	4.42E-08	1.08E-06	no
ENSG00000125144	MT1G	15.969	-3.605	0.659	-5.471	4.47E-08	1.09E-06	no
ENSG00000106070	GRB10	59.524	1.033	0.189	5.470	4.51E-08	1.10E-06	no
ENSG00000197586	ENTPD6	66.082	1.046	0.192	5.455	4.91E-08	1.19E-06	no
ENSG00000110066	KMT5B	94.765	0.846	0.155	5.455	4.90E-08	1.19E-06	no
ENSG00000164626	KCNK5	25.674	1.344	0.246	5.452	4.98E-08	1.21E-06	no
ENSG00000197956	S100A6	3011.598	-0.917	0.168	-5.452	4.99E-08	1.21E-06	no
ENSG00000105971	CAV2	108.654	1.113	0.205	5.442	5.27E-08	1.27E-06	no
ENSG00000125347	IRF1	57.214	0.948	0.175	5.433	5.53E-08	1.33E-06	no
ENSG00000140044	JDP2	23.587	1.620	0.298	5.431	5.59E-08	1.34E-06	no
ENSG00000169021	UQCRFS1	98.309	-0.814	0.150	-5.428	5.69E-08	1.37E-06	no
ENSG00000064666	CNN2	148.689	-0.750	0.138	-5.426	5.76E-08	1.38E-06	no
ENSG00000114554	PLXNA1	125.308	-0.744	0.137	-5.416	6.10E-08	1.46E-06	no
ENSG00000164402	SEPTIN8	61.608	-0.924	0.171	-5.413	6.21E-08	1.48E-06	no
ENSG00000180537	RNF182	24.381	1.714	0.317	5.403	6.56E-08	1.56E-06	no
ENSG00000197324	LRP10	162.335	0.866	0.160	5.395	6.87E-08	1.63E-06	no
ENSG00000105953	OGDH	109.040	0.755	0.140	5.391	7.01E-08	1.66E-06	no
ENSG00000167644	C19orf33	236.220	0.754	0.140	5.383	7.31E-08	1.73E-06	no
ENSG00000104356	POP1	83.398	-0.923	0.171	-5.383	7.33E-08	1.73E-06	no
ENSG00000144426	NBEAL1	55.999	1.116	0.208	5.374	7.71E-08	1.81E-06	no
ENSG00000118503	TNFAIP3	11.743	1.881	0.350	5.373	7.76E-08	1.82E-06	no
ENSG00000171517	LPAR3	23.627	1.395	0.260	5.369	7.93E-08	1.85E-06	no
ENSG00000213593	TMX2	92.281	0.946	0.176	5.365	8.08E-08	1.89E-06	no
ENSG00000152465	NMT2	127.566	-0.710	0.132	-5.361	8.29E-08	1.93E-06	no
ENSG00000158805	ZNF276	31.785	1.450	0.271	5.349	8.84E-08	2.05E-06	no
ENSG00000188313	PLSCR1	42.327	1.204	0.225	5.345	9.03E-08	2.09E-06	no
ENSG00000137563	GGH	49.991	-1.268	0.238	-5.330	9.81E-08	2.26E-06	no
ENSG00000139112	GABARAPL1	43.478	1.304	0.245	5.324	1.02E-07	2.34E-06	no
ENSG00000125746	EML2	93.110	0.840	0.158	5.316	1.06E-07	2.44E-06	no
ENSG00000179862	CITED4	53.884	1.064	0.200	5.312	1.09E-07	2.49E-06	no
ENSG00000074319	TSG101	79.307	0.920	0.174	5.292	1.21E-07	2.74E-06	no
ENSG00000196584	XRCC2	73.453	-0.867	0.164	-5.281	1.29E-07	2.90E-06	no
ENSG00000168209	DDIT4	132.698	1.065	0.202	5.279	1.30E-07	2.92E-06	no
ENSG00000115758	ODC1	211.288	-0.729	0.138	-5.269	1.37E-07	3.07E-06	no
ENSG00000108509	CAMTA2	33.220	1.189	0.226	5.265	1.40E-07	3.13E-06	no
ENSG00000132842	AP3B1	115.177	-0.711	0.136	-5.249	1.53E-07	3.40E-06	no
ENSG00000183955	KMT5A	90.998	0.808	0.154	5.243	1.58E-07	3.49E-06	no
ENSG00000142875	PRKACB	47.738	-1.572	0.300	-5.243	1.58E-07	3.49E-06	no
ENSG00000197965	MPZL1	83.896	-0.842	0.161	-5.241	1.59E-07	3.51E-06	no
ENSG00000168078	PBK	96.779	-0.753	0.144	-5.240	1.60E-07	3.53E-06	no
ENSG00000070882	OSBPL3	57.112	0.961	0.183	5.236	1.64E-07	3.59E-06	no

ENSG00000111665	CDCA3	69.479	-0.947	0.181	-5.230	1.70E-07	3.70E-06	no
ENSG00000069702	TGFBR3	31.381	1.311	0.251	5.228	1.72E-07	3.73E-06	no
ENSG00000173917	HOXB2	24.613	1.791	0.343	5.226	1.73E-07	3.75E-06	no
ENSG00000118971	CCND2	20.415	2.459	0.472	5.210	1.89E-07	4.08E-06	no
ENSG00000166401	SERPINB8	28.698	1.326	0.254	5.210	1.89E-07	4.08E-06	no
ENSG00000171492	LRRC8D	53.883	1.029	0.198	5.204	1.95E-07	4.19E-06	no
ENSG00000095383	TBC1D2	29.632	1.379	0.265	5.199	2.00E-07	4.29E-06	no
ENSG00000024422	EHD2	67.231	0.918	0.177	5.197	2.02E-07	4.34E-06	no
ENSG00000143977	SNRPG	161.784	-0.964	0.186	-5.190	2.10E-07	4.49E-06	no
ENSG00000169855	ROBO1	29.656	-1.369	0.264	-5.188	2.13E-07	4.53E-06	no
ENSG00000149428	HYOU1	175.752	0.745	0.144	5.172	2.31E-07	4.91E-06	no
ENSG00000071242	RPS6KA2	14.517	2.159	0.418	5.168	2.36E-07	5.01E-06	no
ENSG00000247596	TWF2	59.612	1.000	0.194	5.167	2.38E-07	5.03E-06	no
ENSG00000170677	SOCS6	106.887	0.799	0.155	5.165	2.40E-07	5.07E-06	no
ENSG00000103995	CEP152	117.710	-0.885	0.171	-5.163	2.43E-07	5.11E-06	no
ENSG00000138061	CYP1B1	73.894	-1.263	0.245	-5.154	2.54E-07	5.34E-06	no
ENSG00000153551	CMTM7	72.234	0.985	0.191	5.146	2.66E-07	5.55E-06	no
ENSG00000138134	STAMBPL1	15.510	2.154	0.419	5.143	2.70E-07	5.63E-06	no
ENSG00000197302	ZNF720	50.985	0.956	0.186	5.139	2.76E-07	5.72E-06	no
ENSG00000177383	MAGEF1	68.928	-0.816	0.159	-5.135	2.82E-07	5.84E-06	no
ENSG00000140836	ZFHX3	105.653	0.740	0.144	5.130	2.90E-07	5.99E-06	no
ENSG00000163508	EOMES	12.104	-1.742	0.341	-5.114	3.16E-07	6.48E-06	no
ENSG00000171720	HDAC3	92.401	-0.726	0.142	-5.102	3.36E-07	6.87E-06	no
ENSG00000123219	CENPK	72.958	-0.946	0.186	-5.088	3.62E-07	7.35E-06	no
ENSG00000114446	IFT57	77.753	-0.880	0.173	-5.085	3.67E-07	7.40E-06	no
ENSG00000117691	NENF	114.833	-0.700	0.138	-5.085	3.67E-07	7.40E-06	no
ENSG00000075336	TIMM21	41.787	-1.193	0.234	-5.086	3.66E-07	7.40E-06	no
ENSG00000011105	TSPAN9	23.668	1.340	0.264	5.082	3.74E-07	7.51E-06	no
ENSG00000121152	NCAPH	73.047	-0.995	0.196	-5.080	3.78E-07	7.59E-06	no
ENSG00000196535	MYO18A	102.975	0.861	0.170	5.078	3.81E-07	7.62E-06	no
ENSG00000173848	NET1	106.924	0.709	0.140	5.065	4.09E-07	8.14E-06	no
ENSG00000177666	PNPLA2	73.208	0.967	0.191	5.065	4.08E-07	8.14E-06	no
ENSG00000152818	UTRN	102.333	1.043	0.206	5.056	4.27E-07	8.48E-06	no
ENSG00000132646	PCNA	138.303	-0.858	0.170	-5.036	4.76E-07	9.40E-06	no
ENSG00000240230	COX19	91.643	0.812	0.161	5.033	4.84E-07	9.52E-06	no
ENSG00000261373	VPS9D1-AS1	21.339	1.460	0.290	5.033	4.84E-07	9.52E-06	no
ENSG00000234745	HLA-B	440.335	0.722	0.144	5.021	5.15E-07	1.01E-05	no
ENSG00000112759	SLC29A1	105.683	-0.934	0.186	-5.012	5.39E-07	1.05E-05	no
ENSG00000008256	CYTH3	61.041	0.975	0.195	5.010	5.44E-07	1.06E-05	no
ENSG00000123096	SSPN	20.332	1.545	0.309	5.002	5.68E-07	1.10E-05	no
ENSG00000163435	ELF3	47.249	-1.611	0.322	-4.997	5.83E-07	1.13E-05	no
ENSG00000100139	MICALL1	38.565	0.977	0.196	4.997	5.84E-07	1.13E-05	no
ENSG00000178078	STAP2	16.243	2.121	0.425	4.992	5.97E-07	1.15E-05	no

ENSG00000181744	DIPK2A	11.601	-3.001	0.602	-4.987	6.12E-07	1.18E-05	no
ENSG00000143674	MAP3K21	17.559	-2.356	0.473	-4.982	6.30E-07	1.21E-05	no
ENSG00000212464	SNORA12	37.301	1.687	0.339	4.979	6.38E-07	1.22E-05	no
ENSG00000136146	MED4	93.362	-0.707	0.142	-4.972	6.61E-07	1.26E-05	no
ENSG00000170802	FOXN2	47.028	0.948	0.191	4.972	6.64E-07	1.27E-05	no
ENSG00000209082	MT-TL1	2408.153	1.198	0.241	4.970	6.71E-07	1.28E-05	no
ENSG00000057019	DCBLD2	364.749	-0.807	0.163	-4.963	6.96E-07	1.32E-05	no
ENSG00000257218	GATC	66.964	0.814	0.164	4.962	6.99E-07	1.33E-05	no
ENSG00000128567	PODXL	164.094	1.386	0.280	4.943	7.69E-07	1.46E-05	no
ENSG00000169306	IL1RAPL1	16.472	-2.024	0.410	-4.936	7.97E-07	1.50E-05	no
ENSG00000132669	RIN2	53.783	1.054	0.213	4.937	7.93E-07	1.50E-05	no
ENSG00000162426	SLC45A1	15.532	1.647	0.334	4.935	7.99E-07	1.50E-05	no
ENSG00000154874	CCDC144B	13.993	1.727	0.350	4.934	8.04E-07	1.51E-05	no
ENSG00000239039	SNORD13	83.464	0.970	0.197	4.922	8.57E-07	1.60E-05	no
ENSG00000146918	NCAPG2	122.114	-0.724	0.147	-4.921	8.62E-07	1.61E-05	no
ENSG00000111981	ULBP1	33.227	1.263	0.257	4.920	8.66E-07	1.62E-05	no
ENSG00000107560	RAB11FIP2	72.318	0.771	0.157	4.900	9.58E-07	1.78E-05	no
ENSG00000110497	AMBRA1	19.489	1.435	0.293	4.899	9.64E-07	1.79E-05	no
ENSG00000152219	ARL14EP	85.528	0.866	0.177	4.894	9.86E-07	1.82E-05	no
ENSG00000260549	MT1L	70.222	1.673	0.342	4.894	9.89E-07	1.82E-05	no
ENSG00000156253	RWDD2B	57.061	0.930	0.190	4.894	9.88E-07	1.82E-05	no
ENSG00000196182	STK40	54.856	0.839	0.172	4.893	9.93E-07	1.82E-05	no
ENSG00000185112	FAM43A	57.068	1.143	0.234	4.889	1.02E-06	1.86E-05	no
ENSG00000139083	ETV6	32.068	1.289	0.264	4.882	1.05E-06	1.92E-05	no
ENSG00000080031	PTPRH	30.917	1.287	0.264	4.882	1.05E-06	1.92E-05	no
ENSG00000120334	CENPL	34.968	-1.367	0.280	-4.876	1.08E-06	1.97E-05	no
ENSG00000006016	CRLF1	36.219	-1.380	0.283	-4.875	1.09E-06	1.98E-05	no
ENSG00000155313	USP25	46.781	1.040	0.213	4.873	1.10E-06	1.99E-05	no
ENSG00000258655	ARHGAP5-AS1	11.456	1.822	0.375	4.864	1.15E-06	2.08E-05	no
ENSG00000081923	ATP8B1	15.469	5.949	1.224	4.862	1.16E-06	2.09E-05	no
ENSG00000050820	BCAR1	82.464	0.892	0.184	4.862	1.16E-06	2.09E-05	no
ENSG00000120093	HOXB3	58.243	1.222	0.252	4.854	1.21E-06	2.18E-05	no
ENSG00000184205	TSPYL2	68.858	-1.040	0.214	-4.853	1.21E-06	2.18E-05	no
ENSG00000156052	GNAQ	37.853	-1.800	0.372	-4.845	1.26E-06	2.26E-05	no
ENSG00000107863	ARHGAP21	99.372	0.787	0.163	4.832	1.35E-06	2.41E-05	no
ENSG00000149809	TM7SF2	37.120	1.080	0.224	4.832	1.35E-06	2.41E-05	no
ENSG00000108511	HOXB6	39.271	1.371	0.284	4.830	1.36E-06	2.42E-05	no
ENSG00000136826	KLF4	26.997	1.445	0.299	4.830	1.36E-06	2.42E-05	no
ENSG00000136928	GABBR2	11.517	-2.237	0.463	-4.828	1.38E-06	2.45E-05	no
ENSG00000157483	MYO1E	69.543	0.774	0.160	4.825	1.40E-06	2.48E-05	no
ENSG00000122378	PRXL2A	62.364	0.914	0.190	4.818	1.45E-06	2.56E-05	no
ENSG00000189337	KAZN	54.575	-0.982	0.204	-4.817	1.46E-06	2.57E-05	no
ENSG00000132635	PCED1A	57.692	1.174	0.244	4.816	1.46E-06	2.58E-05	no

ENSG00000215039	CD27-AS1	19.273	1.328	0.276	4.813	1.48E-06	2.61E-05	no
ENSG00000117697	NSL1	48.411	-1.039	0.216	-4.807	1.53E-06	2.69E-05	no
ENSG00000147224	PRPS1	65.006	-0.949	0.197	-4.806	1.54E-06	2.69E-05	no
ENSG00000139725	RHOF	47.684	1.036	0.216	4.801	1.58E-06	2.76E-05	no
ENSG00000188641	DPYD	99.498	-0.885	0.184	-4.799	1.60E-06	2.79E-05	no
ENSG00000161671	EMC10	47.481	1.456	0.303	4.797	1.61E-06	2.81E-05	no
ENSG00000089248	ERP29	153.333	0.706	0.147	4.794	1.63E-06	2.85E-05	no
ENSG00000224945	n/a	19.292	1.182	0.246	4.793	1.64E-06	2.85E-05	no
ENSG00000189057	FAM111B	85.587	-0.927	0.193	-4.792	1.65E-06	2.86E-05	no
ENSG00000110987	BCL7A	43.488	1.067	0.223	4.791	1.66E-06	2.87E-05	no
ENSG00000149311	ATM	103.505	0.732	0.154	4.765	1.89E-06	3.25E-05	no
ENSG00000075461	CACNG4	16.713	-1.787	0.375	-4.765	1.89E-06	3.25E-05	no
ENSG00000162599	NFIA	52.397	1.158	0.243	4.761	1.92E-06	3.31E-05	no
ENSG00000133302	SLF1	69.174	-0.876	0.184	-4.760	1.94E-06	3.32E-05	no
ENSG00000139926	FRMD6	38.137	1.634	0.344	4.751	2.02E-06	3.46E-05	no
ENSG00000214063	TSPAN4	116.659	0.757	0.159	4.751	2.02E-06	3.46E-05	no
ENSG00000241685	ARPC1A	87.544	0.770	0.162	4.750	2.04E-06	3.48E-05	no
ENSG00000100387	RBX1	95.188	-0.780	0.165	-4.737	2.17E-06	3.70E-05	no
ENSG00000271122	HERPUD2-AS1	24.441	1.190	0.252	4.730	2.25E-06	3.81E-05	no
ENSG00000156603	MED19	74.429	0.822	0.174	4.726	2.29E-06	3.88E-05	no
ENSG00000198130	HIBCH	45.042	-0.905	0.192	-4.721	2.35E-06	3.97E-05	no
ENSG00000174243	DDX23	75.555	-0.845	0.179	-4.715	2.42E-06	4.08E-05	no
ENSG00000182606	TRAK1	39.804	1.001	0.213	4.702	2.58E-06	4.31E-05	no
ENSG00000047188	YTHDC2	63.934	-0.845	0.180	-4.698	2.62E-06	4.38E-05	no
ENSG00000059378	PARP12	18.643	1.636	0.349	4.684	2.82E-06	4.68E-05	no
ENSG00000172725	CORO1B	80.094	0.833	0.178	4.683	2.83E-06	4.70E-05	no
ENSG00000204217	BMPR2	78.087	0.849	0.181	4.678	2.90E-06	4.79E-05	no
ENSG00000023330	ALAS1	46.836	1.177	0.252	4.673	2.97E-06	4.91E-05	no
ENSG00000068323	TFE3	59.390	0.811	0.174	4.667	3.06E-06	5.04E-05	no
ENSG00000120451	SNX19	40.270	1.160	0.249	4.665	3.08E-06	5.06E-05	no
ENSG00000166920	C15orf48	52.598	2.997	0.645	4.648	3.36E-06	5.49E-05	no
ENSG00000110435	PDHX	60.792	0.820	0.176	4.645	3.41E-06	5.57E-05	no
ENSG00000144468	RHBDD1	17.411	1.674	0.361	4.644	3.42E-06	5.58E-05	no
ENSG00000143740	SNAP47	34.646	-1.021	0.220	-4.636	3.55E-06	5.78E-05	no
ENSG00000003147	ICA1	15.388	1.841	0.398	4.627	3.71E-06	6.02E-05	no
ENSG00000196611	MMP1	15.449	-2.200	0.476	-4.618	3.87E-06	6.26E-05	no
ENSG00000277791	PSMB3	112.080	-0.892	0.193	-4.613	3.97E-06	6.40E-05	no
ENSG00000154265	ABCA5	15.330	1.683	0.365	4.613	3.98E-06	6.41E-05	no
ENSG00000142408	CACNG8	127.801	0.701	0.152	4.612	3.99E-06	6.41E-05	no
ENSG00000151503	NCAPD3	69.996	-0.818	0.177	-4.610	4.02E-06	6.46E-05	no
ENSG00000189171	S100A13	150.115	-0.819	0.178	-4.605	4.12E-06	6.60E-05	no
ENSG00000129003	VPS13C	83.259	0.748	0.163	4.595	4.32E-06	6.89E-05	no
ENSG00000168496	FEN1	78.285	-0.822	0.179	-4.594	4.36E-06	6.93E-05	no

ENSG00000228594	FNDC10	13.487	1.616	0.352	4.593	4.37E-06	6.94E-05	no
ENSG00000143158	MPC2	60.863	-0.816	0.178	-4.590	4.42E-06	7.01E-05	no
ENSG00000196204	RNF216P1	60.274	0.847	0.185	4.584	4.55E-06	7.19E-05	no
ENSG00000032742	IFT88	36.233	-1.116	0.244	-4.581	4.64E-06	7.30E-05	no
ENSG00000174804	FZD4	11.404	1.746	0.381	4.579	4.67E-06	7.34E-05	no
ENSG00000136868	SLC31A1	55.371	0.837	0.183	4.568	4.92E-06	7.69E-05	no
ENSG00000162413	KLHL21	40.345	0.957	0.210	4.561	5.08E-06	7.93E-05	no
ENSG00000197321	SVIL	60.727	0.935	0.205	4.562	5.08E-06	7.93E-05	no
ENSG00000163001	CFAP36	79.228	-0.743	0.163	-4.551	5.33E-06	8.28E-05	no
ENSG00000163918	RFC4	43.933	-0.876	0.194	-4.528	5.96E-06	9.20E-05	no
ENSG00000196976	LAGE3	103.660	-0.785	0.174	-4.521	6.16E-06	9.49E-05	no
ENSG00000127564	PKMYT1	49.425	-0.936	0.207	-4.517	6.27E-06	9.62E-05	no
ENSG00000136450	SRSF1	160.008	-1.111	0.246	-4.511	6.44E-06	9.87E-05	no
ENSG00000122952	ZWINT	55.869	-0.863	0.191	-4.510	6.48E-06	9.92E-05	no
ENSG00000116584	ARHGEF2	146.548	0.757	0.168	4.509	6.53E-06	9.98E-05	no
ENSG00000173207	CKS1B	202.286	-0.921	0.204	-4.507	6.58E-06	1.00E-04	no
ENSG00000113645	WWC1	88.862	-0.914	0.203	-4.504	6.68E-06	1.02E-04	no
ENSG00000080200	CRYBG3	67.339	0.830	0.185	4.493	7.02E-06	1.06E-04	no
ENSG00000240065	PSMB9	38.727	1.107	0.247	4.486	7.26E-06	1.09E-04	no
ENSG00000170955	CAVIN3	26.996	1.105	0.247	4.484	7.34E-06	1.10E-04	no
ENSG00000177830	CHID1	78.731	0.794	0.177	4.481	7.43E-06	1.11E-04	no
ENSG00000172888	ZNF621	57.705	0.823	0.184	4.480	7.48E-06	1.12E-04	no
ENSG00000178531	CTXN1	45.577	-1.041	0.233	-4.472	7.76E-06	1.16E-04	no
ENSG00000254635	WAC-AS1	45.215	0.953	0.214	4.465	8.02E-06	1.19E-04	no
ENSG00000242802	AP5Z1	54.582	0.781	0.175	4.461	8.15E-06	1.21E-04	no
ENSG00000198925	ATG9A	41.627	0.982	0.220	4.458	8.26E-06	1.22E-04	no
ENSG00000184986	TMEM121	15.077	-1.534	0.344	-4.458	8.29E-06	1.22E-04	no
ENSG00000197712	FAM114A1	65.564	0.849	0.190	4.457	8.31E-06	1.23E-04	no
ENSG00000031691	CENPQ	52.669	-0.964	0.217	-4.453	8.47E-06	1.25E-04	no
ENSG00000147155	EBP	81.425	-0.712	0.160	-4.453	8.48E-06	1.25E-04	no
ENSG00000168404	MLKL	18.684	1.446	0.325	4.452	8.50E-06	1.25E-04	no
ENSG00000137965	IFI44	10.619	2.647	0.596	4.445	8.77E-06	1.29E-04	no
ENSG00000117593	DARS2	40.178	-0.919	0.207	-4.442	8.93E-06	1.31E-04	no
ENSG00000108679	LGALS3BP	88.548	0.845	0.190	4.440	8.99E-06	1.31E-04	no
ENSG00000130921	MTRFR	82.912	0.746	0.168	4.438	9.09E-06	1.33E-04	no
ENSG00000088367	EPB41L1	37.217	-0.934	0.211	-4.422	9.79E-06	1.42E-04	no
ENSG00000148841	ITPRIP	29.259	1.167	0.264	4.415	1.01E-05	1.46E-04	no
ENSG00000108852	MPP2	16.627	-1.522	0.345	-4.413	1.02E-05	1.47E-04	no
ENSG00000065457	ADAT1	42.675	0.902	0.204	4.411	1.03E-05	1.48E-04	no
ENSG00000272068	BCAN-AS1	34.787	0.908	0.206	4.407	1.05E-05	1.50E-04	no
ENSG00000171960	PPIH	51.390	-0.998	0.227	-4.406	1.05E-05	1.51E-04	no
ENSG00000084112	SSH1	94.412	0.772	0.175	4.403	1.07E-05	1.53E-04	no
ENSG00000115902	SLC1A4	23.250	1.198	0.272	4.401	1.08E-05	1.54E-04	no

ENSG00000157514	TSC22D3	21.418	1.943	0.443	4.388	1.14E-05	1.63E-04	no
ENSG00000078902	TOLLIP	54.444	0.940	0.214	4.386	1.15E-05	1.64E-04	no
ENSG00000111321	LTBR	108.172	0.802	0.183	4.386	1.16E-05	1.64E-04	no
ENSG00000157193	LRP8	45.034	-0.846	0.193	-4.381	1.18E-05	1.68E-04	no
ENSG00000164237	CMBL	36.320	0.980	0.224	4.379	1.19E-05	1.68E-04	no
ENSG00000016391	CHDH	11.111	2.084	0.476	4.378	1.20E-05	1.69E-04	no
ENSG00000233085	n/a	97.920	0.747	0.171	4.377	1.20E-05	1.70E-04	no
ENSG00000111700	SLCO1B3	19.187	1.348	0.308	4.374	1.22E-05	1.72E-04	no
ENSG00000059758	CDK17	30.264	1.144	0.262	4.364	1.27E-05	1.79E-04	no
ENSG00000169567	HINT1	719.669	-0.725	0.166	-4.358	1.31E-05	1.84E-04	no
ENSG00000103196	CRISPLD2	26.530	1.286	0.295	4.354	1.34E-05	1.87E-04	no
ENSG00000213186	TRIM59	22.146	5.393	1.240	4.350	1.36E-05	1.90E-04	no
ENSG00000143641	GALNT2	61.213	-0.938	0.216	-4.348	1.37E-05	1.91E-04	no
ENSG00000117226	GBP3	16.684	1.815	0.418	4.346	1.39E-05	1.92E-04	no
ENSG00000168505	GBX2	11.990	-1.437	0.331	-4.341	1.42E-05	1.96E-04	no
ENSG00000135763	URB2	26.254	-1.329	0.306	-4.338	1.44E-05	1.99E-04	no
ENSG00000145362	ANK2	17.689	1.396	0.322	4.334	1.46E-05	2.01E-04	no
ENSG00000099337	KCNK6	20.615	1.391	0.321	4.334	1.46E-05	2.01E-04	no
ENSG00000125257	ABCC4	59.158	-0.818	0.189	-4.331	1.48E-05	2.04E-04	no
ENSG00000157693	TMEM268	18.662	1.169	0.271	4.315	1.60E-05	2.18E-04	no
ENSG00000145050	MANF	81.737	-0.891	0.207	-4.314	1.60E-05	2.19E-04	no
ENSG00000164919	COX6C	313.832	-0.791	0.184	-4.308	1.64E-05	2.24E-04	no
ENSG00000006756	ARSD	54.449	0.795	0.185	4.305	1.67E-05	2.27E-04	no
ENSG00000105011	ASF1B	61.695	-0.907	0.211	-4.302	1.70E-05	2.30E-04	no
ENSG00000140525	FANCI	60.134	-0.804	0.187	-4.299	1.71E-05	2.32E-04	no
ENSG00000149636	DSN1	60.382	-0.854	0.199	-4.294	1.76E-05	2.37E-04	no
ENSG00000169926	KLF13	39.327	1.010	0.235	4.290	1.79E-05	2.41E-04	no
ENSG00000257178	n/a	19.223	1.851	0.432	4.285	1.83E-05	2.45E-04	no
ENSG00000005801	ZNF195	62.943	0.850	0.198	4.283	1.85E-05	2.47E-04	no
ENSG00000143179	UCK2	71.240	-0.731	0.171	-4.279	1.88E-05	2.52E-04	no
ENSG00000158402	CDC25C	44.425	-0.880	0.206	-4.276	1.91E-05	2.55E-04	no
ENSG00000035499	DEPDC1B	52.979	-0.979	0.230	-4.264	2.01E-05	2.68E-04	no
ENSG00000197256	KANK2	58.681	-1.077	0.253	-4.264	2.01E-05	2.68E-04	no
ENSG00000233493	TMEM238	33.968	1.030	0.242	4.261	2.04E-05	2.72E-04	no
ENSG00000104783	KCNN4	71.412	0.758	0.178	4.258	2.07E-05	2.75E-04	no
ENSG00000147862	NFIB	115.635	0.775	0.182	4.254	2.10E-05	2.78E-04	no
ENSG00000228742	LINC02577	32.876	-1.000	0.235	-4.249	2.14E-05	2.84E-04	no
ENSG00000148926	ADM	95.383	1.628	0.385	4.231	2.33E-05	3.06E-04	no
ENSG00000204116	CHIC1	11.528	3.104	0.734	4.229	2.35E-05	3.09E-04	no
ENSG00000176593	n/a	19.464	1.362	0.322	4.224	2.40E-05	3.15E-04	no
ENSG00000198830	HMGN2	72.261	-0.838	0.199	-4.217	2.48E-05	3.25E-04	no
ENSG00000106415	GLCCI1	14.737	2.235	0.531	4.209	2.57E-05	3.36E-04	no
ENSG00000139684	ESD	90.157	-0.861	0.205	-4.205	2.61E-05	3.41E-04	no

ENSG00000136048	DRAM1	21.959	1.268	0.302	4.200	2.67E-05	3.49E-04	no
ENSG00000063322	MED29	248.385	0.716	0.170	4.199	2.68E-05	3.49E-04	no
ENSG00000175115	PACS1	54.694	0.989	0.236	4.198	2.70E-05	3.51E-04	no
ENSG00000179833	SERTAD2	51.100	1.002	0.239	4.188	2.81E-05	3.65E-04	no
ENSG00000024526	DEPDC1	91.189	-0.743	0.178	-4.183	2.88E-05	3.74E-04	no
ENSG00000164172	MOCS2	52.017	-0.887	0.212	-4.181	2.90E-05	3.76E-04	no
ENSG00000215788	TNFRSF25	23.766	1.130	0.270	4.178	2.95E-05	3.81E-04	no
ENSG00000171864	PRND	18.586	-1.623	0.389	-4.176	2.96E-05	3.83E-04	no
ENSG00000176208	ATAD5	85.727	-0.724	0.174	-4.171	3.03E-05	3.92E-04	no
ENSG00000082515	MRPL22	107.346	-0.760	0.182	-4.169	3.06E-05	3.95E-04	no
ENSG00000023839	ABCC2	32.539	0.816	0.196	4.167	3.08E-05	3.97E-04	no
ENSG00000137500	CCDC90B	64.404	0.721	0.173	4.166	3.09E-05	3.98E-04	no
ENSG00000165480	SKA3	38.990	-0.948	0.227	-4.166	3.10E-05	3.98E-04	no
ENSG00000051341	POLQ	45.385	-0.850	0.204	-4.165	3.12E-05	4.00E-04	no
ENSG00000240972	MIF	29.687	-1.239	0.298	-4.163	3.14E-05	4.03E-04	no
ENSG00000113356	POLR3G	59.971	-0.711	0.171	-4.158	3.22E-05	4.11E-04	no
ENSG00000107960	STN1	23.481	1.217	0.293	4.157	3.22E-05	4.11E-04	no
ENSG00000153879	CEBPG	101.760	0.758	0.182	4.156	3.24E-05	4.13E-04	no
ENSG00000131174	COX7B	205.479	-0.710	0.171	-4.151	3.31E-05	4.21E-04	no
ENSG00000172977	KAT5	47.193	1.137	0.274	4.147	3.37E-05	4.27E-04	no
ENSG00000196754	S100A2	44.182	-0.873	0.210	-4.146	3.38E-05	4.27E-04	no
ENSG00000148985	PGAP2	41.228	1.036	0.250	4.145	3.39E-05	4.29E-04	no
ENSG00000180917	CMTR2	55.187	0.795	0.192	4.142	3.44E-05	4.34E-04	no
ENSG00000169733	RFNG	72.858	0.737	0.178	4.140	3.48E-05	4.37E-04	no
ENSG00000029153	ARNTL2	29.363	1.829	0.443	4.134	3.57E-05	4.49E-04	no
ENSG00000170385	SLC30A1	33.014	1.033	0.250	4.128	3.65E-05	4.58E-04	no
ENSG00000261824	LINC00662	49.287	0.826	0.200	4.125	3.70E-05	4.64E-04	no
ENSG00000165572	KBTBD6	24.231	-1.152	0.280	-4.121	3.77E-05	4.71E-04	no
ENSG00000123104	ITPR2	93.077	0.702	0.170	4.120	3.79E-05	4.73E-04	no
ENSG00000175606	TMEM70	35.832	-1.120	0.272	-4.118	3.83E-05	4.77E-04	no
ENSG00000167972	ABCA3	13.046	1.655	0.402	4.117	3.83E-05	4.78E-04	no
ENSG00000205885	C1RL-AS1	26.323	1.216	0.296	4.104	4.05E-05	5.03E-04	no
ENSG00000173598	NUDT4	37.668	0.898	0.219	4.103	4.07E-05	5.05E-04	no
ENSG00000134250	NOTCH2	74.342	-0.701	0.171	-4.100	4.12E-05	5.11E-04	no
ENSG00000120708	TGFBI	70.573	0.822	0.200	4.100	4.13E-05	5.11E-04	no
ENSG00000138735	PDE5A	22.276	-3.587	0.877	-4.088	4.35E-05	5.36E-04	no
ENSG00000130208	APOC1	32.223	1.005	0.246	4.087	4.36E-05	5.37E-04	no
ENSG00000015475	BID	68.613	-0.727	0.178	-4.085	4.41E-05	5.43E-04	no
ENSG00000119899	SLC17A5	37.376	0.915	0.224	4.081	4.48E-05	5.50E-04	no
ENSG00000154144	TBRG1	76.453	0.751	0.184	4.081	4.49E-05	5.51E-04	no
ENSG00000267270	PARD6G-AS1	11.335	-1.619	0.397	-4.077	4.56E-05	5.56E-04	no
ENSG00000179348	GATA2	41.393	-0.836	0.206	-4.067	4.77E-05	5.79E-04	no
ENSG00000102897	LYRM1	24.556	1.049	0.258	4.063	4.85E-05	5.86E-04	no

ENSG00000123609	NMI	72.742	0.767	0.189	4.058	4.95E-05	5.97E-04	no
ENSG00000156535	CD109	69.154	0.814	0.201	4.056	5.00E-05	6.01E-04	no
ENSG00000076513	ANKRD13A	58.571	0.875	0.216	4.049	5.14E-05	6.17E-04	no
ENSG00000126822	PLEKHG3	31.102	-1.304	0.322	-4.048	5.17E-05	6.19E-04	no
ENSG00000093009	CDC45	27.842	-1.068	0.264	-4.043	5.27E-05	6.30E-04	no
ENSG00000155324	GRAMD2B	33.279	-1.113	0.275	-4.043	5.28E-05	6.30E-04	no
ENSG00000139629	GALNT6	23.384	1.282	0.317	4.040	5.35E-05	6.39E-04	no
ENSG00000135116	HRK	10.462	1.734	0.430	4.035	5.45E-05	6.48E-04	no
ENSG00000140931	CMTM3	46.713	0.790	0.196	4.034	5.49E-05	6.51E-04	no
ENSG00000166333	ILK	50.123	0.826	0.205	4.034	5.49E-05	6.51E-04	no
ENSG00000210140	MT-TC	21.884	1.175	0.291	4.033	5.50E-05	6.51E-04	no
ENSG00000161800	RACGAP1	38.301	-1.153	0.286	-4.034	5.49E-05	6.51E-04	no
ENSG00000160783	PMF1	19.746	-1.490	0.371	-4.016	5.91E-05	6.95E-04	no
ENSG00000170779	CDCA4	49.924	-0.885	0.221	-4.015	5.95E-05	6.99E-04	no
ENSG00000165996	HACD1	30.115	0.945	0.235	4.013	6.00E-05	7.03E-04	no
ENSG00000145604	SKP2	55.740	-0.905	0.226	-4.013	6.00E-05	7.03E-04	no
ENSG00000067715	SYT1	38.240	0.913	0.228	4.003	6.26E-05	7.31E-04	no
ENSG00000198858	R3HDM4	53.081	0.883	0.221	3.996	6.44E-05	7.50E-04	no
ENSG00000129757	CDKN1C	20.137	1.524	0.382	3.993	6.53E-05	7.57E-04	no
ENSG00000188483	IER5L	48.799	-0.894	0.224	-3.984	6.77E-05	7.84E-04	no
ENSG00000166452	AKIP1	52.503	0.789	0.198	3.982	6.83E-05	7.89E-04	no
ENSG00000140199	SLC12A6	28.582	1.254	0.315	3.980	6.88E-05	7.94E-04	no
ENSG00000176046	NUPR1	31.760	1.676	0.422	3.976	7.02E-05	8.09E-04	no
ENSG00000149716	LTO1	23.791	1.130	0.285	3.971	7.16E-05	8.24E-04	no
ENSG00000133131	MORC4	56.898	-0.789	0.199	-3.967	7.28E-05	8.36E-04	no
ENSG00000160957	RECQL4	36.389	-0.986	0.249	-3.961	7.47E-05	8.56E-04	no
ENSG00000181264	TLCD5	35.395	0.933	0.236	3.961	7.47E-05	8.56E-04	no
ENSG00000128708	HAT1	62.487	-0.773	0.195	-3.959	7.51E-05	8.60E-04	no
ENSG00000104147	OIP5	32.791	-1.256	0.318	-3.955	7.64E-05	8.73E-04	no
ENSG00000165507	DEPP1	13.012	1.554	0.394	3.946	7.95E-05	9.06E-04	no
ENSG00000163516	ANKZF1	53.836	0.866	0.219	3.945	7.99E-05	9.08E-04	no
ENSG00000173559	NABP1	48.877	1.021	0.259	3.945	7.99E-05	9.08E-04	no
ENSG00000138495	COX17	98.070	-0.859	0.218	-3.937	8.26E-05	9.33E-04	no
ENSG00000100092	SH3BP1	18.062	-1.298	0.330	-3.934	8.37E-05	9.44E-04	no
ENSG00000110628	SLC22A18	23.269	1.210	0.308	3.933	8.38E-05	9.45E-04	no
ENSG00000165626	BEND7	16.932	1.407	0.358	3.933	8.41E-05	9.46E-04	no
ENSG00000174371	EXO1	32.535	-1.087	0.277	-3.930	8.50E-05	9.57E-04	no
ENSG00000233461	n/a	11.602	-1.957	0.499	-3.923	8.73E-05	9.82E-04	no
ENSG00000167555	ZNF528	13.577	1.366	0.348	3.922	8.79E-05	9.88E-04	no
ENSG00000165704	HPRT1	65.129	-0.855	0.219	-3.909	9.29E-05	1.04E-03	no
ENSG00000164171	ITGA2	115.252	-1.787	0.457	-3.908	9.30E-05	1.04E-03	no
ENSG00000094880	CDC23	63.508	-0.877	0.225	-3.906	9.39E-05	1.05E-03	no
ENSG00000181751	MACIR	17.699	-1.371	0.351	-3.901	9.57E-05	1.06E-03	no

ENSG00000165983	PTER	32.626	0.863	0.222	3.895	9.84E-05	1.09E-03	no
ENSG00000065833	ME1	52.995	-0.741	0.191	-3.877	0.000106	0.001166	no
ENSG00000135926	TMBIM1	68.198	0.847	0.219	3.874	0.000107	0.001178	no
ENSG00000210077	MT-TV	114.303	0.849	0.219	3.872	0.000108	0.00119	no
ENSG00000158828	PINK1	57.879	0.759	0.196	3.870	0.000109	0.001195	no
ENSG00000146574	CCZ1B	25.156	-1.347	0.349	-3.863	0.000112	0.001225	no
ENSG00000099377	HSD3B7	43.996	0.913	0.236	3.863	0.000112	0.001225	no
ENSG00000204264	PSMB8	28.371	1.385	0.360	3.853	0.000117	0.00127	no
ENSG00000204876	n/a	14.368	1.125	0.292	3.852	0.000117	0.001275	no
ENSG00000198113	TOR4A	62.197	0.707	0.184	3.849	0.000119	0.001287	no
ENSG00000163050	COQ8A	25.678	-1.013	0.263	-3.846	0.00012	0.001304	no
ENSG00000101213	PTK6	11.450	1.558	0.405	3.844	0.000121	0.00131	no
ENSG00000134049	IER3IP1	56.328	-0.788	0.205	-3.842	0.000122	0.00132	no
ENSG00000167323	STIM1	52.593	0.766	0.200	3.837	0.000125	0.001347	no
ENSG00000178773	CPNE7	84.744	0.854	0.223	3.836	0.000125	0.001347	no
ENSG00000107521	HPS1	56.815	0.714	0.186	3.836	0.000125	0.001347	no
ENSG00000204227	RING1	75.044	0.726	0.189	3.836	0.000125	0.001347	no
ENSG00000101447	FAM83D	46.599	-0.930	0.243	-3.830	0.000128	0.001376	no
ENSG00000135269	TES	137.335	2.986	0.781	3.826	0.000131	0.001401	no
ENSG00000235884	LINC00941	12.446	1.464	0.385	3.800	0.000145	0.001542	no
ENSG00000133393	CEP20	77.399	-0.779	0.205	-3.796	0.000147	0.001562	no
ENSG00000005448	WDR54	52.734	-0.754	0.199	-3.793	0.000149	0.00158	no
ENSG00000135801	TAF5L	42.678	-0.850	0.224	-3.791	0.00015	0.001589	no
ENSG00000186871	ERCC6L	32.784	-0.925	0.244	-3.790	0.000151	0.001592	no
ENSG00000185869	ZNF829	10.645	2.447	0.646	3.790	0.000151	0.001595	no
ENSG00000158966	CACHD1	10.313	2.001	0.528	3.787	0.000152	0.001606	no
ENSG00000184992	BRI3BP	70.230	-0.734	0.194	-3.785	0.000154	0.001621	no
ENSG00000075340	ADD2	27.718	0.955	0.252	3.781	0.000156	0.001643	no
ENSG00000173267	SNCG	14.880	-1.595	0.422	-3.776	0.000159	0.001672	no
ENSG00000136153	LMO7	28.847	1.159	0.307	3.771	0.000163	0.001704	no
ENSG00000106351	AGFG2	10.320	1.819	0.483	3.764	0.000167	0.001741	no
ENSG00000141753	IGFBP4	42.784	0.968	0.257	3.762	0.000168	0.001751	no
ENSG00000101003	GINS1	39.917	-1.014	0.269	-3.761	0.000169	0.001758	no
ENSG00000077458	FAM76B	39.247	0.930	0.247	3.758	0.000171	0.001777	no
ENSG00000215251	FASTKD5	30.905	-0.846	0.225	-3.754	0.000174	0.001798	no
ENSG00000229833	PET100	30.226	-1.027	0.274	-3.751	0.000176	0.001819	no
ENSG00000139624	CERS5	21.884	1.009	0.269	3.748	0.000178	0.001834	no
ENSG00000168569	TMEM223	35.278	0.920	0.246	3.744	0.000181	0.001861	no
ENSG00000249992	TMEM158	79.393	0.757	0.202	3.741	0.000184	0.001883	no
ENSG00000132182	NUP210	50.023	-0.763	0.204	-3.736	0.000187	0.001919	no
ENSG00000008838	MED24	63.617	0.882	0.236	3.730	0.000191	0.001957	no
ENSG00000183340	JRKL	24.374	1.222	0.328	3.725	0.000196	0.001998	no
ENSG00000067113	PLPP1	23.931	0.996	0.267	3.724	0.000196	0.002001	no

ENSG00000148400	NOTCH1	28.694	-0.999	0.268	-3.723	0.000197	0.002007	no
ENSG00000142694	EVA1B	13.884	1.350	0.363	3.721	0.000199	0.002022	no
ENSG00000237649	KIFC1	86.491	-0.706	0.191	-3.695	0.00022	0.002222	no
ENSG00000149823	VPS51	54.885	0.828	0.224	3.693	0.000222	0.002236	no
ENSG00000164109	MAD2L1	42.227	-0.847	0.229	-3.692	0.000222	0.002239	no
ENSG00000059728	MXD1	21.706	1.292	0.351	3.683	0.00023	0.002313	no
ENSG00000129195	PIMREG	34.894	-0.957	0.260	-3.680	0.000233	0.002336	no
ENSG00000164597	COG5	30.640	0.850	0.231	3.679	0.000234	0.002342	no
ENSG00000188910	GJB3	41.786	0.924	0.252	3.671	0.000242	0.002411	no
ENSG00000085117	CD82	11.889	1.488	0.406	3.667	0.000246	0.002447	no
ENSG00000115738	ID2	44.003	-1.015	0.277	-3.661	0.000251	0.002494	no
ENSG00000103061	SLC7A6OS	46.312	0.718	0.196	3.661	0.000252	0.002499	no
ENSG00000135540	NHSL1	17.502	1.301	0.356	3.656	0.000256	0.002543	no
ENSG00000101935	AMMECR1	47.701	0.716	0.196	3.655	0.000257	0.002549	no
ENSG00000065989	PDE4A	17.122	-1.831	0.501	-3.653	0.000259	0.002566	no
ENSG00000179222	MAGED1	69.008	-0.734	0.201	-3.649	0.000264	0.0026	no
ENSG00000139370	SLC15A4	27.538	1.074	0.294	3.648	0.000264	0.002601	no
ENSG00000196517	SLC6A9	16.766	1.181	0.324	3.647	0.000265	0.002607	no
ENSG00000135452	TSPAN31	10.144	2.519	0.692	3.643	0.000269	0.002636	no
ENSG00000057294	PKP2	16.342	1.907	0.523	3.643	0.00027	0.002636	no
ENSG00000212283	SNORD89	21.218	1.329	0.366	3.634	0.000279	0.002717	no
ENSG00000172269	DPAGT1	42.679	0.794	0.219	3.626	0.000288	0.002803	no
ENSG00000124782	RREB1	46.584	0.791	0.219	3.615	0.0003	0.002909	no
ENSG00000276043	UHRF1	18.435	-1.227	0.340	-3.613	0.000303	0.002935	no
ENSG00000158161	EYA3	33.931	-0.856	0.237	-3.611	0.000305	0.002948	no
ENSG00000272763	n/a	20.300	1.799	0.499	3.604	0.000313	0.003009	no
ENSG00000197299	BLM	40.901	-0.957	0.266	-3.601	0.000317	0.00304	no
ENSG00000135476	ESPL1	23.280	-1.156	0.321	-3.601	0.000317	0.00304	no
ENSG00000116199	FAM20B	52.535	-0.730	0.203	-3.597	0.000322	0.003079	no
ENSG00000242498	ARPIN	12.085	1.375	0.382	3.596	0.000323	0.003087	no
ENSG00000181929	PRKAG1	65.694	0.720	0.201	3.592	0.000328	0.003116	no
ENSG00000086200	IPO11	36.663	-0.838	0.234	-3.587	0.000335	0.00317	no
ENSG00000185347	TEDC1	20.285	-1.000	0.279	-3.584	0.000338	0.003201	no
ENSG00000136986	DERL1	54.448	-1.006	0.281	-3.581	0.000342	0.00323	no
ENSG00000162496	DHRS3	36.596	-1.113	0.311	-3.580	0.000344	0.003248	no
ENSG00000141574	SECTM1	15.069	1.164	0.325	3.578	0.000346	0.003266	no
ENSG00000012963	UBR7	37.939	-0.964	0.270	-3.568	0.00036	0.003374	no
ENSG00000183856	IQGAP3	50.009	-0.747	0.210	-3.563	0.000367	0.003435	no
ENSG00000146278	PNRC1	86.118	-0.786	0.221	-3.560	0.00037	0.003463	no
ENSG00000008277	ADAM22	12.712	1.335	0.375	3.558	0.000373	0.003488	no
ENSG00000125637	PSD4	27.381	0.800	0.225	3.555	0.000379	0.003531	no
ENSG00000197943	PLCG2	13.113	1.391	0.392	3.551	0.000384	0.003568	no
ENSG00000235106	BRD3OS	26.006	-0.937	0.264	-3.551	0.000384	0.003573	no

ENSG00000117228	GBP1	12.834	1.327	0.374	3.550	0.000385	0.003577	no
ENSG00000185163	DDX51	31.040	0.857	0.242	3.546	0.000391	0.003625	no
ENSG00000157837	SPPL3	52.235	0.703	0.198	3.543	0.000395	0.003661	no
ENSG00000105329	TGFB1	27.682	1.141	0.323	3.531	0.000414	0.003807	no
ENSG00000089060	SLC8B1	15.743	1.434	0.406	3.529	0.000416	0.003825	no
ENSG00000059122	FLYWCH1	26.377	0.922	0.261	3.528	0.000419	0.003847	no
ENSG00000168994	PXDC1	33.188	1.233	0.351	3.513	0.000442	0.004047	no
ENSG00000156521	TYSND1	32.277	0.764	0.218	3.512	0.000445	0.004066	no
ENSG00000188010	MORN2	12.764	-1.412	0.402	-3.511	0.000446	0.004074	no
ENSG00000090975	PITPNM2	13.076	1.466	0.418	3.511	0.000447	0.004074	no
ENSG00000204516	MICB	31.863	1.273	0.363	3.510	0.000448	0.004082	no
ENSG00000026950	BTN3A1	12.002	1.566	0.446	3.510	0.000449	0.004088	no
ENSG00000213347	MXD3	11.968	-1.364	0.389	-3.509	0.00045	0.004101	no
ENSG00000135241	PNPLA8	48.264	0.772	0.220	3.505	0.000456	0.004143	no
ENSG00000164070	HSPA4L	47.455	0.748	0.213	3.503	0.00046	0.004169	no
ENSG00000111725	PRKAB1	18.968	1.263	0.362	3.491	0.00048	0.004321	no
ENSG00000247746	USP51	18.171	-1.085	0.311	-3.490	0.000483	0.004344	no
ENSG00000159110	IFNAR2	24.908	0.906	0.260	3.485	0.000493	0.004421	no
ENSG00000175130	MARCKSL1	47.536	-0.733	0.210	-3.484	0.000494	0.004431	no
ENSG00000173334	TRIB1	18.732	1.308	0.376	3.480	0.000501	0.004484	no
ENSG00000145781	COMMD10	30.509	-0.839	0.241	-3.477	0.000507	0.004529	no
ENSG00000118689	FOXO3	60.428	0.752	0.216	3.476	0.000509	0.00454	no
ENSG00000175482	POLD4	19.380	1.415	0.407	3.475	0.000511	0.00455	no
ENSG00000205544	TMEM256	40.820	-0.837	0.241	-3.473	0.000514	0.004581	no
ENSG00000158373	H2BC5	37.538	-1.038	0.299	-3.466	0.000528	0.004694	no
ENSG00000105516	DBP	25.735	-1.150	0.332	-3.459	0.000542	0.004796	no
ENSG00000233338	TLR8-AS1	12.980	-1.216	0.352	-3.452	0.000556	0.004887	no
ENSG00000167112	TRUB2	38.480	-0.739	0.214	-3.452	0.000557	0.004888	no
ENSG00000100426	ZBED4	45.726	-0.738	0.214	-3.451	0.000559	0.004902	no
ENSG00000101188	NTSR1	24.675	1.004	0.291	3.450	0.00056	0.004908	no
ENSG00000148459	PDSS1	12.004	-1.371	0.398	-3.449	0.000562	0.004926	no
ENSG00000189410	SH2D5	23.755	0.940	0.273	3.448	0.000565	0.004946	no
ENSG00000137868	STRA6	25.027	-1.009	0.293	-3.442	0.000578	0.005039	no
ENSG00000155158	TTC39B	19.739	0.982	0.285	3.442	0.000578	0.005042	no
ENSG00000141391	PRELID3A	14.274	1.324	0.385	3.440	0.000582	0.005065	no
ENSG00000198689	SLC9A6	27.467	-0.966	0.281	-3.439	0.000583	0.005067	no
ENSG00000172932	ANKRD13D	17.765	1.229	0.358	3.438	0.000585	0.005081	no
ENSG00000106615	RHEB	50.557	-0.701	0.204	-3.438	0.000585	0.005081	no
ENSG00000116191	RALGPS2	52.993	-0.829	0.241	-3.435	0.000592	0.005127	no
ENSG00000147592	LACTB2	33.712	1.008	0.294	3.433	0.000598	0.005171	no
ENSG00000139970	RTN1	14.042	-1.873	0.546	-3.433	0.000598	0.005171	no
ENSG00000186283	TOR3A	21.526	-0.987	0.288	-3.429	0.000606	0.005232	no
ENSG00000167702	KIFC2	32.285	0.894	0.261	3.421	0.000624	0.005353	no

ENSG00000170903	MSANTD4	37.235	0.778	0.228	3.419	0.000628	0.00538	no
ENSG00000154639	CXADR	33.566	-1.025	0.300	-3.418	0.000631	0.005405	no
ENSG00000011028	MRC2	40.155	-0.810	0.237	-3.413	0.000643	0.005496	no
ENSG00000005513	SOX8	18.266	-0.947	0.278	-3.412	0.000644	0.005507	no
ENSG00000113621	TXNDC15	34.221	-0.808	0.237	-3.412	0.000645	0.00551	no
ENSG00000269028	MTRNR2L12	436.050	0.791	0.232	3.402	0.000668	0.005686	no
ENSG00000189046	ALKBH2	38.540	0.822	0.242	3.398	0.00068	0.005777	no
ENSG00000083635	NUFIP1	40.365	-0.704	0.207	-3.397	0.000682	0.005787	no
ENSG00000135457	TFCP2	42.551	-0.800	0.236	-3.392	0.000695	0.00588	no
ENSG00000020577	SAMD4A	32.099	0.903	0.267	3.389	0.000703	0.005938	no
ENSG00000225648	SBDSP1	44.520	0.713	0.211	3.387	0.000706	0.005957	no
ENSG00000120333	MRPS14	45.793	-0.737	0.218	-3.387	0.000707	0.005965	no
ENSG00000152439	ZNF773	13.405	1.192	0.352	3.386	0.00071	0.005978	no
ENSG00000171606	ZNF274	26.410	0.898	0.265	3.384	0.000714	0.006009	no
ENSG00000164692	COL1A2	39.909	1.052	0.311	3.380	0.000724	0.006086	no
ENSG00000108175	ZMIZ1	42.210	0.783	0.232	3.376	0.000735	0.006166	no
ENSG00000214114	MYCBP	19.004	-1.013	0.301	-3.369	0.000754	0.006302	no
ENSG00000151553	FHIP2A	21.750	1.224	0.363	3.368	0.000757	0.006326	no
ENSG00000116729	WLS	26.711	-1.130	0.336	-3.363	0.000771	0.00643	no
ENSG00000243364	EFNA4	11.595	-1.315	0.392	-3.359	0.000782	0.006512	no
ENSG00000210196	MT-TP	284.986	1.151	0.343	3.359	0.000782	0.006512	no
ENSG00000182749	PAQR7	24.958	1.147	0.342	3.356	0.00079	0.00657	no
ENSG00000118096	IFT46	16.778	1.101	0.329	3.350	0.000809	0.006723	no
ENSG00000103024	NME3	43.004	0.721	0.215	3.347	0.000816	0.006772	no
ENSG00000153037	SRP19	44.184	-0.982	0.294	-3.344	0.000826	0.006836	no
ENSG00000275180	n/a	11.358	-1.283	0.384	-3.342	0.000833	0.006884	no
ENSG00000107485	GATA3	19.230	1.613	0.484	3.336	0.000849	0.006992	no
ENSG00000151690	MFSD6	25.029	-0.907	0.272	-3.336	0.000849	0.006992	no
ENSG00000134222	PSRC1	33.857	-0.778	0.233	-3.333	0.000861	0.007068	no
ENSG00000074590	NUAK1	35.456	0.950	0.285	3.327	0.000878	0.007199	no
ENSG00000132256	TRIM5	19.317	1.170	0.352	3.326	0.00088	0.00721	no
ENSG00000130244	FAM98C	27.198	0.911	0.274	3.326	0.000883	0.007225	no
ENSG00000197696	NMB	18.861	0.922	0.278	3.319	0.000904	0.007378	no
ENSG00000203760	CENPW	37.742	-0.844	0.254	-3.317	0.000909	0.007407	no
ENSG00000278970	HEIH	41.499	0.710	0.214	3.316	0.000912	0.007427	no
ENSG00000167766	ZNF83	41.310	0.772	0.233	3.316	0.000914	0.007431	no
ENSG00000100154	TTC28	30.520	0.832	0.251	3.310	0.000932	0.007567	no
ENSG00000136159	NUDT15	26.040	-0.977	0.295	-3.308	0.000938	0.007611	no
ENSG00000163346	PBXIP1	22.147	-1.014	0.306	-3.307	0.000942	0.007633	no
ENSG00000121211	MND1	38.411	-0.739	0.224	-3.305	0.000949	0.007673	no
ENSG00000171421	MRPL36	46.326	-0.888	0.269	-3.296	0.000981	0.007901	no
ENSG00000065809	FAM107B	51.159	-0.855	0.260	-3.295	0.000986	0.007934	no
ENSG00000110801	PSMD9	57.234	0.725	0.220	3.293	0.00099	0.007957	no

ENSG00000145703	IQGAP2	27.709	-0.822	0.250	-3.289	0.001004	0.008069	no
ENSG00000143499	SMYD2	29.913	-0.786	0.239	-3.289	0.001007	0.008084	no
ENSG00000144736	SHQ1	16.309	-1.134	0.345	-3.288	0.001007	0.008084	no
ENSG00000158552	ZFAND2B	16.887	1.296	0.394	3.288	0.00101	0.008091	no
ENSG00000165821	SALL2	17.867	-0.962	0.294	-3.273	0.001066	0.008493	no
ENSG00000163491	NEK10	10.083	-1.432	0.438	-3.268	0.001082	0.008599	no
ENSG00000143515	ATP8B2	22.661	-0.803	0.247	-3.258	0.001122	0.008852	no
ENSG00000081721	DUSP12	19.908	-1.130	0.347	-3.253	0.001142	0.008996	no
ENSG00000156463	SH3RF2	14.789	1.297	0.399	3.249	0.001157	0.009085	no
ENSG00000147509	RGS20	17.926	1.112	0.342	3.247	0.001167	0.009147	no
ENSG00000113742	CPEB4	41.271	0.910	0.280	3.245	0.001175	0.009189	no
ENSG00000266028	SRGAP2	31.396	-0.784	0.242	-3.245	0.001175	0.009189	no
ENSG00000162341	TPCN2	12.426	1.167	0.360	3.242	0.001187	0.009258	no
ENSG00000201098	RNY1	15.682	-1.252	0.386	-3.241	0.001191	0.00928	no
ENSG00000083844	ZNF264	43.711	0.736	0.227	3.240	0.001197	0.009318	no
ENSG00000173064	HECTD4	44.622	0.762	0.235	3.234	0.001219	0.009449	no
ENSG00000066322	ELOVL1	52.991	0.730	0.226	3.234	0.00122	0.009451	no
ENSG00000175832	ETV4	26.003	0.805	0.249	3.233	0.001227	0.009494	no
ENSG00000165501	LRR1	37.804	-0.785	0.243	-3.232	0.00123	0.009511	no
ENSG00000110011	DNAJC4	17.127	1.345	0.416	3.232	0.001231	0.009515	no
ENSG00000166831	RBPMS2	16.038	-1.076	0.333	-3.231	0.001235	0.009539	no
ENSG00000110844	PRPF40B	24.113	1.004	0.311	3.230	0.001237	0.009555	no
ENSG00000119138	KLF9	24.007	0.983	0.305	3.224	0.001262	0.009723	no
ENSG00000043514	TRIT1	27.993	-0.919	0.285	-3.220	0.001282	0.009847	no
ENSG00000165949	IFI27	12.425	2.677	0.834	3.210	0.001326	0.010136	no
ENSG00000183048	SLC25A10	20.625	-1.084	0.338	-3.208	0.001338	0.010202	no
ENSG00000131196	NFATC1	26.543	-0.755	0.236	-3.203	0.001359	0.010352	no
ENSG00000156671	SAMD8	36.467	0.758	0.237	3.203	0.001362	0.010356	no
ENSG00000263731	n/a	12.358	-1.350	0.422	-3.198	0.001382	0.010498	no
ENSG00000163913	IFT122	24.996	-0.899	0.282	-3.191	0.001419	0.01074	no
ENSG00000135821	GLUL	10.157	1.522	0.478	3.188	0.001433	0.010827	no
ENSG00000115594	IL1R1	21.945	0.977	0.307	3.185	0.001447	0.010906	no
ENSG00000135924	DNAJB2	25.738	1.145	0.360	3.184	0.001451	0.010929	no
ENSG00000106605	BLVRA	31.990	0.746	0.235	3.181	0.001468	0.011045	no
ENSG00000052795	FNIP2	22.156	1.119	0.353	3.171	0.001517	0.011397	no
ENSG00000196639	HRH1	17.861	1.079	0.340	3.171	0.00152	0.011417	no
ENSG00000147654	EBAG9	44.352	0.810	0.256	3.170	0.001522	0.01142	no
ENSG00000214357	NEURL1B	54.359	-0.774	0.244	-3.168	0.001535	0.011494	no
ENSG00000121940	CLCC1	35.664	-0.739	0.234	-3.162	0.001566	0.01168	no
ENSG00000080819	CPOX	18.108	-0.980	0.310	-3.162	0.001568	0.011681	no
ENSG00000149548	CCDC15	19.921	0.950	0.300	3.161	0.00157	0.011692	no
ENSG00000168268	NT5DC2	32.604	-0.899	0.284	-3.161	0.001572	0.011692	no
ENSG00000105290	APLP1	19.012	-1.122	0.355	-3.160	0.001578	0.011733	no

ENSG00000102125	TAFAZZIN	43.948	0.730	0.231	3.157	0.001597	0.011856	no
ENSG00000116691	MIIP	21.422	0.936	0.297	3.155	0.001603	0.011894	no
ENSG00000131650	KREMEN2	14.994	-1.015	0.322	-3.150	0.001634	0.012084	no
ENSG00000106803	SEC61B	120.759	-0.831	0.264	-3.149	0.001636	0.012091	no
ENSG00000129636	ITFG1	34.453	0.770	0.245	3.148	0.001642	0.012133	no
ENSG00000148572	NRBF2	31.889	0.746	0.237	3.146	0.001655	0.012213	no
ENSG00000151883	PARP8	12.405	-4.024	1.281	-3.142	0.001679	0.012329	no
ENSG00000019485	PRDM11	16.920	0.952	0.303	3.142	0.001679	0.012329	no
ENSG00000186854	TRABD2A	16.485	0.946	0.302	3.136	0.001716	0.012536	no
ENSG00000197375	SLC22A5	17.965	-1.029	0.329	-3.127	0.001764	0.012865	no
ENSG00000197183	NOL4L	13.520	-1.286	0.411	-3.127	0.001768	0.012885	no
ENSG00000246695	RASSF8-AS1	22.532	1.116	0.357	3.126	0.001773	0.01291	no
ENSG00000171488	LRRC8C	17.923	-1.153	0.369	-3.125	0.001779	0.012946	no
ENSG00000183814	LIN9	19.097	-1.116	0.357	-3.124	0.001783	0.012964	no
ENSG00000112576	CCND3	31.706	-0.782	0.250	-3.121	0.001802	0.013091	no
ENSG00000155254	MARVELD1	51.189	0.810	0.260	3.118	0.001822	0.013211	no
ENSG00000142173	COL6A2	22.957	0.953	0.306	3.117	0.001825	0.013225	no
ENSG00000154237	LRRK1	27.756	0.984	0.316	3.109	0.001878	0.013571	no
ENSG00000198890	PRMT6	11.878	-1.272	0.410	-3.105	0.001906	0.013744	no
ENSG00000187801	ZFP69B	10.258	1.486	0.479	3.104	0.001911	0.013762	no
ENSG00000148175	STOM	20.454	1.802	0.581	3.102	0.001921	0.01381	no
ENSG00000115828	QPCT	14.060	-1.109	0.358	-3.100	0.001932	0.013874	no
ENSG00000196458	ZNF605	33.796	0.799	0.258	3.098	0.001949	0.013974	no
ENSG00000148835	TAF5	27.098	-0.811	0.262	-3.098	0.00195	0.013976	no
ENSG00000178202	POGLUT3	35.090	0.782	0.252	3.097	0.001952	0.01398	no
ENSG00000100077	GRK3	17.420	-1.030	0.333	-3.092	0.00199	0.014174	no
ENSG00000110046	ATG2A	26.866	0.707	0.229	3.087	0.002021	0.014349	no
ENSG00000162062	TEDC2	10.519	-1.884	0.610	-3.087	0.002023	0.014359	no
ENSG00000125827	TMX4	59.062	-0.725	0.235	-3.087	0.002024	0.01436	no
ENSG00000178996	SNX18	40.111	-0.710	0.230	-3.083	0.00205	0.014515	no
ENSG00000197757	HOXC6	50.429	0.913	0.296	3.081	0.002066	0.014587	no
ENSG00000182197	EXT1	29.516	0.944	0.307	3.073	0.002116	0.014877	no
ENSG00000205213	LGR4	18.419	1.404	0.458	3.065	0.002179	0.015238	no
ENSG00000129810	SGO1	33.738	-0.811	0.265	-3.062	0.002201	0.015369	no
ENSG00000162236	STX5	24.469	0.834	0.273	3.057	0.002235	0.015566	no
ENSG00000166169	POLL	22.552	1.023	0.335	3.051	0.002282	0.015861	no
ENSG00000149476	TKFC	20.202	0.925	0.304	3.045	0.002325	0.016121	no
ENSG00000184281	TSSC4	50.711	0.746	0.245	3.045	0.002326	0.016122	no
ENSG00000112655	PTK7	62.903	-0.745	0.245	-3.044	0.002337	0.01619	no
ENSG00000105486	LIG1	36.635	-0.767	0.252	-3.041	0.002361	0.016337	no
ENSG00000247627	MTND4P12	23.075	0.747	0.246	3.037	0.002386	0.016452	no
ENSG00000225791	TRAM2-AS1	12.135	1.108	0.365	3.035	0.002407	0.01658	no
ENSG00000118655	DCLRE1B	15.763	-1.268	0.418	-3.031	0.002439	0.01677	no

ENSG00000131153	GINS2	40.498	-0.719	0.237	-3.030	0.002444	0.016777	no
ENSG00000147536	GINS4	26.098	-0.774	0.256	-3.029	0.002454	0.016823	no
ENSG00000187193	MT1X	41.928	0.704	0.233	3.023	0.002503	0.017125	no
ENSG00000221164	SNORA11F	12.141	1.295	0.429	3.020	0.002525	0.01726	no
ENSG00000166435	XRRA1	29.303	0.806	0.267	3.017	0.002553	0.017429	no
ENSG00000112144	CILK1	19.616	0.955	0.317	3.013	0.002588	0.017645	no
ENSG00000133816	MICAL2	47.046	0.706	0.235	3.009	0.002622	0.017817	no
ENSG00000170500	LONRF2	34.036	0.806	0.268	3.009	0.002623	0.017819	no
ENSG00000071205	ARHGAP10	26.392	-0.948	0.315	-3.005	0.002652	0.017976	no
ENSG00000088305	DNMT3B	10.958	-1.244	0.414	-3.005	0.002656	0.017988	no
ENSG00000204498	NFKBIL1	74.982	1.068	0.356	3.004	0.002661	0.018005	no
ENSG00000159267	HLCS	27.696	0.788	0.263	3.003	0.002676	0.018087	no
ENSG00000240694	PNMA2	22.303	0.943	0.314	2.999	0.002706	0.01826	no
ENSG00000029534	ANK1	41.932	0.763	0.255	2.988	0.002805	0.018812	no
ENSG00000111875	ASF1A	33.581	0.729	0.244	2.986	0.002823	0.018915	no
ENSG00000183765	CHEK2	27.492	0.867	0.291	2.985	0.002836	0.018972	no
ENSG00000068001	HYAL2	27.980	-0.802	0.270	-2.977	0.002913	0.019416	no
ENSG00000137478	FCHSD2	24.269	0.859	0.289	2.976	0.002921	0.019448	no
ENSG00000185880	TRIM69	13.487	1.181	0.397	2.975	0.00293	0.019485	no
ENSG00000176401	EID2B	25.919	-0.891	0.300	-2.973	0.002947	0.019577	no
ENSG00000143368	SF3B4	91.098	-0.848	0.285	-2.972	0.002959	0.019647	no
ENSG00000215105	TTC3P1	20.260	-0.889	0.299	-2.972	0.002963	0.019667	no
ENSG00000231131	LNCAROD	21.042	0.867	0.292	2.969	0.002986	0.019776	no
ENSG00000125726	CD70	49.997	0.955	0.322	2.969	0.002987	0.019778	no
ENSG00000170412	GPRC5C	13.774	-1.177	0.398	-2.961	0.003069	0.020221	no
ENSG00000210184	MT-TS2	62.084	1.028	0.347	2.960	0.003077	0.020249	no
ENSG00000112874	NUDT12	22.739	-0.845	0.285	-2.960	0.003081	0.020257	no
ENSG00000221909	FAM200A	22.588	0.904	0.306	2.956	0.003115	0.020451	no
ENSG00000256940	PPP1R14B-AS	12.484	1.226	0.415	2.955	0.003129	0.020513	no
ENSG00000170345	FOS	64.560	0.967	0.327	2.953	0.003144	0.0206	no
ENSG00000145740	SLC30A5	36.618	-0.726	0.246	-2.953	0.003152	0.020639	no
ENSG00000183421	RIPK4	29.899	0.812	0.275	2.950	0.003177	0.020747	no
ENSG00000106665	CLIP2	12.018	1.386	0.471	2.943	0.003253	0.021127	no
ENSG00000127328	RAB3IP	49.901	0.776	0.264	2.942	0.003256	0.021133	no
ENSG00000105136	ZNF419	19.938	0.918	0.312	2.939	0.003292	0.021338	no
ENSG00000137145	DENND4C	25.139	0.878	0.299	2.939	0.003296	0.021356	no
ENSG00000064042	LIMCH1	45.875	-1.368	0.466	-2.937	0.003311	0.021439	no
ENSG00000105696	TMEM59L	20.464	-0.788	0.270	-2.922	0.003474	0.022388	no
ENSG00000114383	TUSC2	34.612	-0.717	0.245	-2.921	0.003486	0.022456	no
ENSG00000100726	TELO2	11.161	-1.254	0.429	-2.921	0.003489	0.022462	no
ENSG00000171161	ZNF672	13.552	-1.260	0.431	-2.921	0.003492	0.022472	no
ENSG00000102078	SLC25A14	16.903	-0.961	0.329	-2.919	0.003509	0.02257	no
ENSG00000079257	LXN	11.520	-3.639	1.248	-2.916	0.00355	0.022802	no

ENSG00000167216	KATNAL2	16.369	0.992	0.341	2.911	0.003599	0.023093	no
ENSG00000146411	SLC2A12	11.506	1.471	0.506	2.909	0.003623	0.023237	no
ENSG00000175213	ZNF408	12.458	1.192	0.410	2.905	0.003668	0.023445	no
ENSG00000149289	ZC3H12C	14.856	1.340	0.462	2.902	0.003711	0.023696	no
ENSG00000160179	ABCG1	14.395	-1.006	0.347	-2.897	0.003768	0.023999	no
ENSG00000188747	NOXA1	11.623	0.945	0.326	2.897	0.003769	0.023999	no
ENSG00000116761	CTH	15.695	0.901	0.312	2.888	0.003872	0.024481	no
ENSG00000167965	MLST8	25.044	1.013	0.351	2.887	0.003885	0.024549	no
ENSG00000247092	SNHG10	12.552	-1.086	0.376	-2.886	0.003899	0.024614	no
ENSG00000136943	CTSV	27.574	-0.985	0.341	-2.885	0.003912	0.024688	no
ENSG00000137501	SYTL2	36.286	1.390	0.482	2.883	0.003945	0.024868	no
ENSG00000182362	YBEY	14.242	1.205	0.419	2.879	0.003996	0.025141	no
ENSG00000242588	n/a	24.817	0.740	0.258	2.871	0.004087	0.025646	no
ENSG00000144366	GULP1	68.951	0.762	0.266	2.866	0.004156	0.026002	no
ENSG00000187790	FANCM	29.471	-0.812	0.284	-2.863	0.004198	0.026228	no
ENSG00000130940	CASZ1	24.439	-0.931	0.325	-2.862	0.004205	0.026261	no
ENSG00000145723	GIN1	10.822	-1.228	0.429	-2.862	0.004209	0.026271	no
ENSG00000170946	DNAJC24	27.412	0.760	0.266	2.859	0.004246	0.026429	no
ENSG00000173852	DPY19L1	36.091	-0.767	0.268	-2.859	0.004245	0.026429	no
ENSG00000132563	REEP2	19.295	-0.832	0.291	-2.858	0.004262	0.026518	no
ENSG00000214944	ARHGEF28	44.654	0.838	0.293	2.857	0.004281	0.026609	no
ENSG00000139178	C1RL	18.127	0.914	0.320	2.856	0.004294	0.026641	no
ENSG00000184489	PTP4A3	12.317	1.017	0.356	2.855	0.004301	0.02666	no
ENSG00000158301	GPRASP2	14.398	-0.981	0.345	-2.846	0.004427	0.027354	no
ENSG00000105963	ADAP1	18.436	1.028	0.361	2.844	0.004453	0.027477	no
ENSG00000111224	PARP11	13.250	0.978	0.344	2.840	0.004509	0.027755	no
ENSG00000109189	USP46	23.723	-0.906	0.319	-2.837	0.004559	0.028001	no
ENSG00000177030	DEAF1	43.028	0.774	0.273	2.833	0.004613	0.028303	no
ENSG00000248593	DSTNP2	11.106	1.277	0.451	2.831	0.004638	0.028393	no
ENSG00000267750	RUNDC3A-AS1	16.548	0.919	0.324	2.831	0.004639	0.028393	no
ENSG00000170571	EMB	23.465	-1.932	0.683	-2.828	0.004687	0.028575	no
ENSG00000187231	SESTD1	15.185	-1.443	0.511	-2.827	0.004699	0.028624	no
ENSG00000067365	METTL22	13.498	1.068	0.378	2.826	0.004707	0.028631	no
ENSG00000124104	SNX21	14.658	0.916	0.324	2.824	0.004736	0.028743	no
ENSG00000167987	VPS37C	29.984	0.728	0.258	2.823	0.004759	0.028841	no
ENSG00000107537	PHYH	28.315	0.780	0.277	2.816	0.004855	0.029265	no
ENSG00000186635	ARAP1	28.574	0.774	0.275	2.814	0.004893	0.02944	no
ENSG00000187189	TSPYL4	27.381	-0.743	0.264	-2.814	0.00489	0.02944	no
ENSG00000108384	RAD51C	27.353	-0.799	0.284	-2.813	0.004916	0.029522	no
ENSG00000156973	PDE6D	24.986	-0.763	0.271	-2.812	0.004918	0.029526	no
ENSG00000185753	CXorf38	16.089	0.998	0.355	2.810	0.004949	0.029682	no
ENSG00000200090	n/a	14.629	1.143	0.407	2.806	0.005011	0.029958	no
ENSG00000168795	ZBTB5	20.266	-1.224	0.437	-2.799	0.005133	0.030538	no

ENSG00000143801	PSEN2	17.851	-0.866	0.311	-2.788	0.005308	0.031422	no
ENSG00000166199	ALKBH3	18.906	1.018	0.366	2.784	0.00537	0.031683	no
ENSG00000072609	CHFR	20.266	0.870	0.312	2.784	0.005369	0.031683	no
ENSG00000079156	OSBPL6	16.455	0.794	0.285	2.782	0.005404	0.031825	no
ENSG00000044090	CUL7	20.704	0.814	0.293	2.780	0.005437	0.031978	no
ENSG00000163545	NUAK2	24.230	-0.716	0.258	-2.780	0.00544	0.031978	no
ENSG00000167985	SDHAF2	30.387	0.734	0.265	2.770	0.005604	0.032858	no
ENSG00000104381	GDAP1	17.715	-0.988	0.357	-2.769	0.005628	0.032966	no
ENSG00000166896	ATP23	10.330	-1.324	0.478	-2.768	0.005644	0.033047	no
ENSG00000015133	CCDC88C	26.721	0.703	0.254	2.768	0.005649	0.033047	no
ENSG00000122971	ACADS	13.117	1.011	0.366	2.764	0.005717	0.033433	no
ENSG00000227036	LINC00511	15.810	1.588	0.575	2.762	0.005744	0.033539	no
ENSG00000059145	UNKL	20.391	-0.941	0.341	-2.758	0.005812	0.033853	no
ENSG00000143252	SDHC	150.634	-0.770	0.280	-2.754	0.005886	0.034181	no
ENSG00000071889	FAM3A	37.955	0.702	0.256	2.747	0.00602	0.034778	no
ENSG00000254614	n/a	11.052	1.141	0.415	2.747	0.006023	0.034778	no
ENSG00000178381	ZFAND2A	16.167	1.084	0.396	2.742	0.006113	0.035226	no
ENSG00000214756	CSKMT	29.087	0.821	0.300	2.738	0.006184	0.035569	no
ENSG00000259623	n/a	21.686	-0.827	0.303	-2.734	0.006252	0.035849	no
ENSG00000108439	PNPO	25.506	0.947	0.347	2.732	0.006296	0.036091	no
ENSG00000056558	TRAF1	14.099	1.019	0.373	2.730	0.006335	0.036281	no
ENSG00000102384	CENPI	21.531	-0.930	0.341	-2.728	0.006364	0.036402	no
ENSG00000081320	STK17B	23.092	-0.883	0.324	-2.727	0.006391	0.036465	no
ENSG00000256771	ZNF253	10.541	1.253	0.459	2.727	0.006388	0.036465	no
ENSG00000165997	ARL5B	39.636	0.751	0.276	2.725	0.006423	0.036612	no
ENSG00000213397	HAUS7	22.769	-0.732	0.269	-2.723	0.006471	0.036791	no
ENSG00000128656	CHN1	14.698	-1.024	0.377	-2.717	0.006595	0.037398	no
ENSG00000087008	ACOX3	13.028	0.937	0.345	2.714	0.006647	0.037597	no
ENSG00000197892	KIF13B	17.929	0.812	0.299	2.712	0.006681	0.037712	no
ENSG00000137760	ALKBH8	16.848	0.884	0.326	2.711	0.0067	0.037773	no
ENSG00000230148	HOXB-AS1	17.023	0.930	0.344	2.706	0.006806	0.038317	no
ENSG00000146242	TPBG	17.319	-1.062	0.393	-2.706	0.006809	0.038319	no
ENSG00000167700	MFSD3	27.823	0.786	0.291	2.704	0.006842	0.038409	no
ENSG00000131876	SNRPA1	32.260	-0.764	0.283	-2.702	0.006895	0.038625	no
ENSG00000132591	ERAL1	22.434	-0.745	0.276	-2.701	0.006903	0.038652	no
ENSG00000272079	n/a	10.940	-1.887	0.699	-2.701	0.00691	0.038652	no
ENSG00000203326	ZNF525	14.140	3.390	1.255	2.701	0.006922	0.038662	no
ENSG00000112149	CD83	14.081	1.060	0.394	2.688	0.007189	0.039954	no
ENSG00000085840	ORC1	13.615	-1.181	0.440	-2.683	0.00729	0.04043	no
ENSG00000075043	KCNQ2	31.706	-0.805	0.300	-2.681	0.007345	0.040622	no
ENSG00000160298	C21orf58	26.668	-0.742	0.277	-2.679	0.007394	0.040801	no
ENSG00000136631	VPS45	28.640	-0.737	0.275	-2.679	0.007391	0.040801	no
ENSG00000166575	TMEM135	24.354	0.734	0.274	2.675	0.007483	0.041124	no

ENSG00000147789	ZNF7	21.454	0.928	0.348	2.669	0.007606	0.041767	no
ENSG00000120696	KBTBD7	14.780	-0.981	0.368	-2.665	0.007708	0.042219	no
ENSG00000239282	CASTOR1	16.553	0.851	0.320	2.658	0.007851	0.042896	no
ENSG00000107371	EXOSC3	33.358	-0.741	0.279	-2.655	0.007936	0.043273	no
ENSG00000112855	HARS2	22.750	-0.765	0.288	-2.653	0.007981	0.043462	no
ENSG00000173846	PLK3	17.763	0.744	0.280	2.652	0.007996	0.043491	no
ENSG00000126705	AHDC1	26.574	-0.727	0.274	-2.651	0.008016	0.043583	no
ENSG00000151332	MBIP	16.966	-0.861	0.325	-2.651	0.008033	0.04364	no
ENSG00000116604	MEF2D	33.766	-0.730	0.276	-2.649	0.008072	0.043763	no
ENSG00000078487	ZCWPW1	16.513	0.800	0.302	2.649	0.008067	0.043763	no
ENSG00000183486	MX2	17.337	0.841	0.318	2.645	0.008158	0.044177	no
ENSG00000189362	NEMP2	12.632	0.967	0.366	2.645	0.008165	0.044195	no
ENSG00000279088	n/a	16.384	0.764	0.290	2.638	0.008348	0.044948	no
ENSG00000196967	ZNF585A	17.382	0.836	0.317	2.637	0.008364	0.044996	no
ENSG00000198040	ZNF84	26.661	0.756	0.287	2.635	0.008404	0.045125	no
ENSG00000164741	DLC1	29.053	-0.838	0.318	-2.634	0.008436	0.045276	no
ENSG00000138835	RGS3	20.024	-0.892	0.339	-2.634	0.008443	0.045295	no
ENSG00000152661	GJA1	24.475	1.569	0.596	2.631	0.008513	0.045636	no
ENSG00000178878	APOLD1	19.764	0.766	0.291	2.630	0.008532	0.045718	no
ENSG00000158158	CNNM4	23.097	-0.801	0.305	-2.630	0.008551	0.045801	no
ENSG00000104626	ERI1	35.605	0.713	0.273	2.616	0.008905	0.047289	no
ENSG00000104671	DCTN6	19.170	-0.894	0.342	-2.612	0.009007	0.047724	no
ENSG00000160191	PDE9A	12.005	0.907	0.348	2.603	0.009239	0.048721	no
ENSG00000092445	TYRO3	14.376	-0.869	0.334	-2.600	0.009332	0.049074	no
ENSG00000130675	MNX1	19.097	-0.795	0.306	-2.597	0.009392	0.049355	no
ENSG00000145675	PIK3R1	23.056	-0.821	0.316	-2.597	0.009415	0.049418	no
ENSG00000102781	KATNAL1	20.911	-0.761	0.294	-2.585	0.009734	0.05079	no
ENSG00000152894	PTPRK	15.832	-1.438	0.557	-2.580	0.009893	0.051498	no
ENSG00000171163	ZNF692	22.690	-0.852	0.331	-2.578	0.009924	0.051641	no
ENSG00000066583	ISOC1	27.047	-0.855	0.332	-2.576	0.010009	0.051962	no
ENSG00000271383	NBPF19	11.912	-0.937	0.364	-2.572	0.010109	0.052238	no
ENSG00000174705	SH3PXD2B	22.492	-0.808	0.314	-2.571	0.010134	0.052304	no
ENSG00000123179	EBPL	42.075	-0.715	0.279	-2.560	0.010452	0.053614	no
ENSG00000075234	TTC38	12.531	-0.960	0.375	-2.561	0.010452	0.053614	no
ENSG00000010072	SPRTN	24.245	-0.752	0.294	-2.559	0.01051	0.053826	no
ENSG00000103254	ANTKMT	25.259	-0.786	0.308	-2.553	0.010688	0.054568	no
ENSG00000126603	GLIS2	26.574	-1.083	0.424	-2.553	0.010685	0.054568	no
ENSG00000135525	MAP7	20.347	-1.252	0.493	-2.540	0.011095	0.056218	no
ENSG00000198053	SIRPA	15.235	-0.830	0.328	-2.530	0.011409	0.057458	no
ENSG00000062822	POLD1	16.404	-0.795	0.317	-2.510	0.012073	0.059875	no
ENSG00000002079	MYH16	15.842	2.628	0.405	6.497	8.20E-11	6.19E-09	yes
ENSG00000006459	KDM7A	20.969	1.081	0.308	3.506	0.000456	0.006546	yes

ENSG00000011422	PLAUR	131.985	1.274	0.203	6.285	3.29E-10	2.35E-08	yes
ENSG00000013364	MVP	48.713	0.903	0.239	3.776	0.000159	0.002752	yes
ENSG00000014216	CAPN1	71.156	0.794	0.199	3.994	6.50E-05	0.001284	yes
ENSG00000016391	CHDH	11.111	2.286	0.491	4.654	3.26E-06	9.25E-05	yes
ENSG00000018408	WWTR1	340.848	0.824	0.100	8.222	2.00E-16	2.83E-14	yes
ENSG00000023191	RNH1	160.440	0.875	0.123	7.092	1.32E-12	1.30E-10	yes
ENSG00000023330	ALAS1	46.836	1.050	0.257	4.079	4.52E-05	0.000944	yes
ENSG00000023445	BIRC3	139.420	2.357	0.144	16.377	2.77E-60	1.92E-56	yes
ENSG00000026508	CD44	874.240	1.020	0.075	13.580	5.28E-42	3.49E-39	yes
ENSG00000028203	VEZT	121.229	0.725	0.140	5.174	2.29E-07	8.54E-06	yes
ENSG00000029153	ARNTL2	29.363	1.295	0.435	2.974	0.002935	0.029065	yes
ENSG00000041353	RAB27B	130.622	2.448	0.167	14.617	2.20E-48	2.78E-45	yes
ENSG00000042493	CAPG	133.883	0.938	0.198	4.729	2.26E-06	6.68E-05	yes
ENSG00000043039	BARX2	14.526	1.719	0.371	4.634	3.59E-06	0.000101	yes
ENSG00000048052	HDAC9	42.298	1.715	0.247	6.947	3.73E-12	3.39E-10	yes
ENSG00000049249	TNFRSF9	14.223	2.046	0.382	5.354	8.59E-08	3.50E-06	yes
ENSG00000050344	NFE2L3	71.258	1.698	0.189	9.006	2.13E-19	4.30E-17	yes
ENSG00000050820	BCAR1	82.464	1.117	0.192	5.811	6.20E-09	3.44E-07	yes
ENSG00000051108	HERPUD1	68.204	1.066	0.202	5.265	1.40E-07	5.45E-06	yes
ENSG00000051825	MPHOSPH9	61.401	0.725	0.216	3.360	0.00078	0.010097	yes
ENSG00000057294	PKP2	16.342	1.818	0.501	3.633	0.000281	0.004404	yes
ENSG00000059378	PARP12	18.643	1.374	0.348	3.947	7.91E-05	0.001533	yes
ENSG00000059758	CDK17	30.264	1.324	0.292	4.531	5.88E-06	0.000156	yes
ENSG00000064012	CASP8	68.542	0.868	0.221	3.929	8.55E-05	0.001638	yes
ENSG00000065308	TRAM2	54.359	0.763	0.257	2.967	0.003006	0.029536	yes
ENSG00000065613	SLK	579.073	0.786	0.081	9.751	1.83E-22	4.47E-20	yes
ENSG00000067066	SP100	97.485	0.815	0.139	5.874	4.26E-09	2.46E-07	yes
ENSG00000067715	SYT1	38.240	0.813	0.247	3.293	0.00099	0.012297	yes
ENSG00000069702	TGFBR3	31.381	0.996	0.266	3.750	0.000177	0.002983	yes
ENSG00000071242	RPS6KA2	14.517	1.576	0.453	3.478	0.000506	0.007118	yes
ENSG00000071575	TRIB2	48.093	0.925	0.246	3.758	0.000171	0.002901	yes
ENSG00000071967	CYBRD1	23.600	0.784	0.279	2.805	0.005032	0.044191	yes
ENSG00000072609	CHFR	20.266	1.162	0.354	3.285	0.00102	0.012581	yes
ENSG00000073417	PDE8A	32.483	1.225	0.265	4.617	3.90E-06	0.000109	yes
ENSG00000074201	CLNS1A	215.795	0.759	0.115	6.582	4.63E-11	3.62E-09	yes
ENSG00000075213	SEMA3A	312.088	0.746	0.114	6.546	5.92E-11	4.57E-09	yes
ENSG00000075223	SEMA3C	148.607	2.345	0.156	15.024	5.08E-51	1.18E-47	yes
ENSG00000075618	FSCN1	157.715	1.363	0.162	8.400	4.46E-17	6.73E-15	yes
ENSG00000075711	DLG1	303.613	1.579	0.101	15.609	6.36E-55	2.21E-51	yes
ENSG00000076685	NT5C2	100.661	0.821	0.191	4.300	1.71E-05	0.000397	yes
ENSG00000077238	IL4R	26.778	0.839	0.277	3.030	0.002444	0.025359	yes
ENSG00000077458	FAM76B	39.247	0.754	0.263	2.865	0.004172	0.038136	yes
ENSG00000078114	NEBL	18.071	1.259	0.376	3.350	0.000809	0.010395	yes

ENSG00000080503	SMARCA2	36.959	1.000	0.284	3.525	0.000424	0.006244	yes
ENSG00000081041	CXCL2	15.145	3.819	0.535	7.140	9.37E-13	9.71E-11	yes
ENSG00000081923	ATP8B1	15.469	3.687	1.236	2.983	0.002854	0.028483	yes
ENSG00000085832	EPS15	91.952	1.085	0.158	6.862	6.80E-12	5.98E-10	yes
ENSG00000088826	SMOX	40.989	1.020	0.225	4.542	5.57E-06	0.000149	yes
ENSG00000089127	OAS1	10.147	3.361	0.516	6.508	7.60E-11	5.80E-09	yes
ENSG00000091136	LAMB1	112.642	0.788	0.229	3.437	0.000588	0.008054	yes
ENSG00000095383	TBC1D2	29.632	1.080	0.285	3.789	0.000151	0.002629	yes
ENSG00000099284	MACROH2A2	15.393	0.901	0.305	2.959	0.003091	0.030096	yes
ENSG00000099810	MTAP	44.128	2.185	0.650	3.360	0.000779	0.010092	yes
ENSG00000100106	TRIOBP	24.536	1.138	0.331	3.439	0.000584	0.008038	yes
ENSG00000100154	TTC28	30.520	0.891	0.258	3.450	0.00056	0.007731	yes
ENSG00000100219	XBP1	99.308	0.714	0.151	4.714	2.43E-06	7.10E-05	yes
ENSG00000100342	APOL1	16.947	1.464	0.391	3.742	0.000183	0.003067	yes
ENSG00000100441	KHNYN	53.463	0.703	0.207	3.389	0.000701	0.009293	yes
ENSG00000100612	DHRS7	55.889	0.710	0.211	3.369	0.000753	0.00978	yes
ENSG00000100852	ARHGAP5	343.284	1.532	0.106	14.497	1.27E-47	1.26E-44	yes
ENSG00000100906	NFKBIA	387.539	0.857	0.119	7.211	5.55E-13	5.84E-11	yes
ENSG00000100994	PYGB	423.073	1.071	0.132	8.140	3.95E-16	5.37E-14	yes
ENSG00000101187	SLCO4A1	131.353	1.010	0.138	7.319	2.50E-13	2.67E-11	yes
ENSG00000101213	PTK6	11.450	1.486	0.438	3.395	0.000687	0.009139	yes
ENSG00000101255	TRIB3	93.875	0.876	0.187	4.688	2.75E-06	7.94E-05	yes
ENSG00000101457	DNTTIP1	135.358	1.101	0.154	7.133	9.85E-13	1.01E-10	yes
ENSG00000101935	AMMECR1	47.701	0.949	0.212	4.489	7.17E-06	0.000185	yes
ENSG00000102218	RP2	10.472	1.310	0.446	2.935	0.003331	0.032069	yes
ENSG00000102897	LYRM1	24.556	1.106	0.314	3.519	0.000434	0.006333	yes
ENSG00000103037	SETD6	28.378	0.829	0.270	3.066	0.002167	0.023148	yes
ENSG00000103196	CRISPLD2	26.530	1.269	0.305	4.168	3.08E-05	0.000673	yes
ENSG00000103335	PIEZO1	105.019	0.757	0.137	5.507	3.65E-08	1.63E-06	yes
ENSG00000104332	SFRP1	27.576	4.637	0.526	8.817	1.18E-18	2.15E-16	yes
ENSG00000105810	CDK6	198.946	2.309	0.209	11.022	2.98E-28	1.15E-25	yes
ENSG00000105879	CBLL1	40.693	0.780	0.230	3.395	0.000686	0.009135	yes
ENSG00000105953	OGDH	109.040	0.831	0.148	5.636	1.74E-08	8.53E-07	yes
ENSG00000105993	DNAJB6	174.925	0.916	0.116	7.892	2.96E-15	3.81E-13	yes
ENSG00000106049	HIBADH	13.181	1.214	0.388	3.128	0.00176	0.019787	yes
ENSG00000106278	PTPRZ1	19.690	1.467	0.372	3.944	8.01E-05	0.00155	yes
ENSG00000106366	SERPINE1	445.748	1.636	0.197	8.323	8.58E-17	1.26E-14	yes
ENSG00000106392	C1GALT1	57.719	0.700	0.240	2.924	0.003454	0.033005	yes
ENSG00000106603	COA1	46.300	0.885	0.246	3.597	0.000322	0.00495	yes
ENSG00000107201	DDX58	51.948	1.229	0.211	5.830	5.54E-09	3.11E-07	yes
ENSG00000107438	PDLIM1	80.576	1.805	0.309	5.850	4.92E-09	2.79E-07	yes
ENSG00000107485	GATA3	19.230	1.595	0.538	2.962	0.003058	0.029879	yes
ENSG00000107554	DNMBP	51.518	1.306	0.246	5.301	1.15E-07	4.61E-06	yes

ENSG00000107798	LIPA	110.684	0.916	0.164	5.577	2.45E-08	1.16E-06	yes
ENSG00000107960	STN1	23.481	1.126	0.296	3.805	0.000142	0.002501	yes
ENSG00000107984	DKK1	86.357	0.803	0.251	3.196	0.001393	0.016179	yes
ENSG00000108342	CSF3	19.686	2.846	0.308	9.234	2.60E-20	5.47E-18	yes
ENSG00000108439	PNPO	25.506	1.026	0.335	3.060	0.002211	0.023502	yes
ENSG00000108511	HOXB6	39.271	1.193	0.285	4.181	2.90E-05	0.000639	yes
ENSG00000109452	INPP4B	21.944	2.226	0.407	5.474	4.40E-08	1.93E-06	yes
ENSG00000109572	CLCN3	99.091	0.789	0.168	4.707	2.51E-06	7.29E-05	yes
ENSG00000109929	SC5D	21.698	1.231	0.424	2.903	0.003701	0.034818	yes
ENSG00000110047	EHD1	130.784	0.935	0.162	5.777	7.60E-09	4.08E-07	yes
ENSG00000110057	UNC93B1	34.506	1.233	0.326	3.789	0.000151	0.002633	yes
ENSG00000110090	CPT1A	91.930	1.440	0.232	6.201	5.62E-10	3.79E-08	yes
ENSG00000110330	BIRC2	163.381	0.748	0.131	5.693	1.25E-08	6.32E-07	yes
ENSG00000110422	HIPK3	68.229	1.180	0.215	5.493	3.95E-08	1.75E-06	yes
ENSG00000110435	PDHX	60.792	0.946	0.188	5.040	4.66E-07	1.63E-05	yes
ENSG00000110871	COQ5	40.545	0.809	0.235	3.438	0.000586	0.008042	yes
ENSG00000110888	CAPRIN2	428.918	0.706	0.123	5.766	8.12E-09	4.31E-07	yes
ENSG00000110906	KCTD10	39.135	0.720	0.247	2.921	0.003487	0.033203	yes
ENSG00000111057	KRT18	1526.946	0.751	0.074	10.147	3.43E-24	1.04E-21	yes
ENSG00000111145	ELK3	117.773	0.751	0.134	5.584	2.34E-08	1.11E-06	yes
ENSG00000111331	OAS3	42.949	2.382	0.252	9.435	3.90E-21	8.74E-19	yes
ENSG00000111335	OAS2	11.094	3.877	0.549	7.063	1.63E-12	1.59E-10	yes
ENSG00000111450	STX2	63.817	0.803	0.189	4.260	2.04E-05	0.000467	yes
ENSG00000111671	SPSB2	10.710	1.563	0.516	3.030	0.002444	0.025359	yes
ENSG00000111696	NT5DC3	23.371	1.027	0.345	2.976	0.002918	0.028996	yes
ENSG00000111981	ULBP1	33.227	0.833	0.290	2.872	0.004083	0.03752	yes
ENSG00000112096	SOD2	68.304	1.711	0.175	9.754	1.77E-22	4.40E-20	yes
ENSG00000112715	VEGFA	117.858	0.971	0.168	5.775	7.69E-09	4.11E-07	yes
ENSG00000113070	HBEGF	84.188	0.873	0.194	4.500	6.81E-06	0.000176	yes
ENSG00000113161	HMGCR	91.332	0.790	0.163	4.829	1.37E-06	4.27E-05	yes
ENSG00000113594	LIFR	46.563	1.158	0.232	5.002	5.69E-07	1.95E-05	yes
ENSG00000114784	EIF1B	39.843	0.745	0.243	3.067	0.002165	0.023148	yes
ENSG00000115267	IFIH1	41.210	2.323	0.237	9.809	1.03E-22	2.70E-20	yes
ENSG00000115415	STAT1	161.309	0.765	0.123	6.247	4.17E-10	2.91E-08	yes
ENSG00000115594	IL1R1	21.945	1.698	0.345	4.921	8.59E-07	2.84E-05	yes
ENSG00000115902	SLC1A4	23.250	0.995	0.328	3.036	0.002398	0.025014	yes
ENSG00000116761	CTH	15.695	1.162	0.383	3.030	0.002442	0.025359	yes
ENSG00000117226	GBP3	16.684	1.699	0.533	3.189	0.001428	0.016539	yes
ENSG00000117472	TSPAN1	34.754	2.142	0.253	8.460	2.68E-17	4.14E-15	yes
ENSG00000117868	ESYT2	123.129	0.841	0.141	5.947	2.73E-09	1.63E-07	yes
ENSG00000117984	CTSD	363.134	1.007	0.133	7.582	3.41E-14	3.98E-12	yes
ENSG00000118096	IFT46	16.778	1.269	0.363	3.498	0.00047	0.006678	yes
ENSG00000118503	TNFAIP3	11.743	1.455	0.389	3.745	0.000181	0.003036	yes

ENSG00000118515	SGK1	148.656	2.047	0.141	14.538	6.96E-48	7.44E-45	yes
ENSG00000118640	VAMP8	41.593	2.264	0.485	4.667	3.05E-06	8.69E-05	yes
ENSG00000118971	CCND2	20.415	1.238	0.448	2.760	0.005772	0.048802	yes
ENSG00000119899	SLC17A5	37.376	0.700	0.249	2.811	0.004943	0.043604	yes
ENSG00000119917	IFIT3	91.516	2.902	0.187	15.521	2.49E-54	6.93E-51	yes
ENSG00000119922	IFIT2	120.866	2.212	0.173	12.801	1.61E-37	9.34E-35	yes
ENSG00000120008	WDR11	49.901	0.822	0.223	3.683	0.000231	0.003732	yes
ENSG00000120068	HOXB8	94.497	1.849	0.284	6.505	7.78E-11	5.91E-09	yes
ENSG00000120075	HOXB5	124.243	1.749	0.211	8.283	1.20E-16	1.71E-14	yes
ENSG00000120093	HOXB3	58.243	0.937	0.236	3.962	7.42E-05	0.001447	yes
ENSG00000120129	DUSP1	42.794	1.149	0.282	4.077	4.56E-05	0.000945	yes
ENSG00000120451	SNX19	40.270	1.406	0.290	4.854	1.21E-06	3.86E-05	yes
ENSG00000120458	MSANTD2	19.903	0.948	0.331	2.861	0.004224	0.038483	yes
ENSG00000120708	TGFBI	70.573	0.743	0.213	3.482	0.000498	0.007026	yes
ENSG00000120925	RNF170	16.730	1.108	0.320	3.465	0.00053	0.007365	yes
ENSG00000122550	KLHL7	17.779	0.958	0.338	2.832	0.004631	0.041321	yes
ENSG00000122557	HERPUD2	59.586	0.764	0.203	3.770	0.000163	0.002794	yes
ENSG00000122674	CCZ1	60.742	0.941	0.254	3.708	0.000209	0.003439	yes
ENSG00000122678	POLM	20.314	1.341	0.425	3.152	0.001623	0.018496	yes
ENSG00000122861	PLAU	296.797	1.706	0.587	2.905	0.003671	0.034583	yes
ENSG00000122912	SLC25A16	15.585	1.141	0.383	2.979	0.002889	0.028776	yes
ENSG00000123066	MED13L	161.992	0.947	0.175	5.396	6.81E-08	2.84E-06	yes
ENSG00000123094	RASSF8	172.841	1.518	0.149	10.217	1.66E-24	5.13E-22	yes
ENSG00000123096	SSPN	20.332	1.267	0.329	3.855	0.000116	0.00211	yes
ENSG00000123240	OPTN	166.154	0.706	0.113	6.250	4.09E-10	2.87E-08	yes
ENSG00000123609	NMI	72.742	0.897	0.199	4.514	6.37E-06	0.000167	yes
ENSG00000124104	SNX21	14.658	0.916	0.330	2.774	0.005532	0.047215	yes
ENSG00000124145	SDC4	197.324	0.933	0.134	6.955	3.53E-12	3.23E-10	yes
ENSG00000124839	RAB17	13.931	2.947	0.902	3.267	0.001087	0.013198	yes
ENSG00000124942	AHNAK	439.064	0.924	0.084	10.982	4.65E-28	1.70E-25	yes
ENSG00000125347	IRF1	57.214	0.815	0.174	4.691	2.72E-06	7.87E-05	yes
ENSG00000125398	SOX9	339.357	2.066	0.112	18.440	6.27E-76	8.71E-72	yes
ENSG00000125618	PAX8	14.817	2.463	0.416	5.917	3.27E-09	1.92E-07	yes
ENSG00000125637	PSD4	27.381	1.481	0.243	6.089	1.14E-09	7.27E-08	yes
ENSG00000125740	FOSB	67.021	0.914	0.235	3.892	9.93E-05	0.001864	yes
ENSG00000126709	IFI6	41.801	1.723	0.232	7.422	1.15E-13	1.27E-11	yes
ENSG00000126777	KTN1	2524.085	0.843	0.066	12.726	4.25E-37	2.36E-34	yes
ENSG00000126821	SGPP1	31.367	1.103	0.287	3.846	0.00012	0.002173	yes
ENSG00000126934	MAP2K2	233.020	0.730	0.131	5.562	2.67E-08	1.24E-06	yes
ENSG00000127603	MACF1	714.172	1.213	0.081	14.896	3.52E-50	5.44E-47	yes
ENSG00000128342	LIF	13.639	2.149	0.749	2.869	0.004121	0.037792	yes
ENSG00000128564	VGF	41.804	1.012	0.251	4.034	5.48E-05	0.001113	yes
ENSG00000128567	PODXL	164.094	1.890	0.285	6.627	3.42E-11	2.73E-09	yes

ENSG00000128965	CHAC1	50.149	0.905	0.256	3.530	0.000415	0.00614	yes
ENSG00000130024	PHF10	58.379	0.896	0.213	4.212	2.53E-05	0.000563	yes
ENSG00000130303	BST2	32.864	1.585	0.302	5.246	1.55E-07	5.99E-06	yes
ENSG00000130589	HELZ2	82.030	0.935	0.152	6.169	6.89E-10	4.58E-08	yes
ENSG00000130779	CLIP1	493.116	0.750	0.086	8.692	3.55E-18	5.94E-16	yes
ENSG00000131069	ACSS2	12.663	1.246	0.408	3.055	0.002247	0.023777	yes
ENSG00000132109	TRIM21	10.539	1.317	0.467	2.820	0.004803	0.042502	yes
ENSG00000132141	CCT6B	10.111	1.299	0.443	2.934	0.003345	0.032178	yes
ENSG00000132254	ARFIP2	22.151	0.960	0.345	2.788	0.005309	0.0457	yes
ENSG00000132386	SERPINF1	18.415	2.084	0.346	6.021	1.74E-09	1.07E-07	yes
ENSG00000132470	ITGB4	100.207	1.629	0.167	9.766	1.57E-22	3.96E-20	yes
ENSG00000133316	WDR74	38.893	0.760	0.247	3.080	0.002071	0.022465	yes
ENSG00000133321	PLAAT4	16.122	2.254	0.384	5.872	4.32E-09	2.47E-07	yes
ENSG00000134107	BHLHE40	22.550	1.089	0.384	2.837	0.004553	0.040894	yes
ENSG00000134824	FADS2	24.100	1.468	0.376	3.907	9.33E-05	0.001763	yes
ENSG00000134954	ETS1	77.960	0.831	0.204	4.071	4.67E-05	0.000964	yes
ENSG00000135046	ANXA1	412.100	1.121	0.082	13.631	2.62E-42	1.92E-39	yes
ENSG00000135114	OASL	32.690	2.931	0.284	10.338	4.73E-25	1.49E-22	yes
ENSG00000135241	PNPLA8	48.264	1.025	0.239	4.297	1.73E-05	0.000401	yes
ENSG00000135318	NT5E	187.939	2.074	0.150	13.820	1.92E-43	1.57E-40	yes
ENSG00000135373	EHF	65.538	1.490	0.189	7.882	3.21E-15	4.06E-13	yes
ENSG00000135378	PRRG4	33.113	1.585	0.277	5.726	1.03E-08	5.29E-07	yes
ENSG00000135452	TSPAN31	10.144	3.302	1.083	3.050	0.002287	0.02402	yes
ENSG00000135821	GLUL	10.157	1.452	0.493	2.946	0.003218	0.03114	yes
ENSG00000135842	NIBAN1	91.798	0.944	0.180	5.251	1.51E-07	5.85E-06	yes
ENSG00000135899	SP110	55.371	0.808	0.167	4.835	1.33E-06	4.18E-05	yes
ENSG00000135926	TMBIM1	68.198	0.912	0.236	3.872	0.000108	0.001993	yes
ENSG00000136048	DRAM1	21.959	0.953	0.324	2.944	0.003238	0.031283	yes
ENSG00000136051	WASHC4	103.437	0.714	0.146	4.883	1.04E-06	3.36E-05	yes
ENSG00000136193	SCRN1	139.297	0.753	0.176	4.287	1.81E-05	0.000417	yes
ENSG00000137285	TUBB2B	36.388	2.063	0.382	5.403	6.55E-08	2.74E-06	yes
ENSG00000137478	FCHSD2	24.269	1.082	0.292	3.700	0.000215	0.003527	yes
ENSG00000137492	THAP12	42.401	0.838	0.236	3.555	0.000378	0.005713	yes
ENSG00000137500	CCDC90B	64.404	0.785	0.187	4.196	2.72E-05	0.000602	yes
ENSG00000137628	DDX60	61.775	1.969	0.252	7.797	6.32E-15	7.77E-13	yes
ENSG00000137720	C11orf1	15.924	1.577	0.386	4.083	4.44E-05	0.000927	yes
ENSG00000137760	ALKBH8	16.848	1.095	0.381	2.874	0.004053	0.037341	yes
ENSG00000137831	UACA	595.936	1.473	0.091	16.194	5.55E-59	2.57E-55	yes
ENSG00000137962	ARHGAP29	523.492	0.931	0.102	9.103	8.82E-20	1.83E-17	yes
ENSG00000137965	IFI44	10.619	2.302	0.689	3.341	0.000835	0.010689	yes
ENSG00000138018	SELENOI	43.767	0.958	0.239	4.008	6.13E-05	0.001221	yes
ENSG00000138459	SLC35A5	11.217	2.653	0.758	3.501	0.000463	0.006609	yes
ENSG00000138496	PARP9	33.877	1.106	0.225	4.917	8.81E-07	2.89E-05	yes

ENSG00000138642	HERC6	13.369	2.169	0.394	5.506	3.66E-08	1.63E-06	yes
ENSG00000138646	HERC5	46.361	3.019	0.272	11.114	1.07E-28	4.25E-26	yes
ENSG00000138678	GPAT3	78.062	1.589	0.183	8.704	3.21E-18	5.58E-16	yes
ENSG00000138685	FGF2	45.520	1.107	0.187	5.928	3.07E-09	1.82E-07	yes
ENSG00000139083	ETV6	32.068	1.089	0.280	3.886	0.000102	0.001904	yes
ENSG00000139433	GLTP	82.601	1.358	0.157	8.642	5.52E-18	9.02E-16	yes
ENSG00000139926	FRMD6	38.137	1.059	0.373	2.836	0.004566	0.040918	yes
ENSG00000140105	WARS1	362.491	1.272	0.095	13.325	1.67E-40	1.05E-37	yes
ENSG00000140403	DNAJA4	13.262	2.746	0.487	5.638	1.72E-08	8.44E-07	yes
ENSG00000140931	CMTM3	46.713	0.718	0.211	3.406	0.000659	0.008843	yes
ENSG00000141068	KSR1	13.823	1.472	0.406	3.623	0.000291	0.004549	yes
ENSG00000141574	SECTM1	15.069	1.505	0.334	4.505	6.63E-06	0.000173	yes
ENSG00000141682	PMAIP1	96.891	1.560	0.167	9.330	1.06E-20	2.31E-18	yes
ENSG00000142089	IFITM3	481.099	1.089	0.124	8.805	1.31E-18	2.33E-16	yes
ENSG00000142102	PGGHG	24.862	0.865	0.296	2.921	0.003485	0.033203	yes
ENSG00000142197	DOP1B	23.313	1.014	0.254	3.999	6.36E-05	0.001259	yes
ENSG00000142694	EVA1B	13.884	1.230	0.407	3.024	0.002494	0.025768	yes
ENSG00000142798	HSPG2	37.566	1.219	0.238	5.117	3.10E-07	1.13E-05	yes
ENSG00000143153	ATP1B1	231.955	0.801	0.152	5.276	1.32E-07	5.17E-06	yes
ENSG00000144118	RALB	147.436	0.703	0.140	5.014	5.32E-07	1.83E-05	yes
ENSG00000144468	RHBDD1	17.411	1.207	0.369	3.272	0.001068	0.013077	yes
ENSG00000144802	NFKBIZ	37.663	2.702	0.268	10.096	5.73E-24	1.69E-21	yes
ENSG00000145022	TCTA	18.609	1.359	0.400	3.394	0.00069	0.009164	yes
ENSG00000145362	ANK2	17.689	1.659	0.367	4.521	6.16E-06	0.000163	yes
ENSG00000145623	OSMR	33.225	1.290	0.298	4.324	1.53E-05	0.000362	yes
ENSG00000146411	SLC2A12	11.506	1.658	0.534	3.104	0.001909	0.021083	yes
ENSG00000146535	GNA12	77.220	0.744	0.180	4.138	3.50E-05	0.000755	yes
ENSG00000146648	EGFR	299.075	1.102	0.098	11.269	1.86E-29	7.82E-27	yes
ENSG00000147251	DOCK11	136.232	1.019	0.117	8.680	3.96E-18	6.54E-16	yes
ENSG00000147509	RGS20	17.926	1.641	0.373	4.399	1.09E-05	0.000266	yes
ENSG00000148175	STOM	20.454	2.414	0.671	3.597	0.000322	0.00495	yes
ENSG00000148180	GSN	97.885	1.154	0.176	6.558	5.45E-11	4.23E-09	yes
ENSG00000148484	RSU1	103.583	0.714	0.137	5.218	1.80E-07	6.88E-06	yes
ENSG00000148719	DNAJB12	51.699	0.724	0.226	3.202	0.001362	0.015894	yes
ENSG00000149089	APIP	33.334	0.751	0.238	3.151	0.001629	0.018551	yes
ENSG00000149100	EIF3M	300.532	0.809	0.199	4.059	4.92E-05	0.001013	yes
ENSG00000149196	HIKESHI	46.521	0.895	0.252	3.548	0.000388	0.005841	yes
ENSG00000149311	ATM	103.505	0.823	0.154	5.356	8.51E-08	3.48E-06	yes
ENSG00000149485	FADS1	149.671	1.030	0.162	6.343	2.25E-10	1.64E-08	yes
ENSG00000149743	TRPT1	16.733	1.526	0.488	3.130	0.001745	0.019653	yes
ENSG00000149798	CDC42EP2	14.100	1.644	0.556	2.957	0.003108	0.030242	yes
ENSG00000151491	EPS8	70.878	1.470	0.266	5.537	3.07E-08	1.40E-06	yes
ENSG00000151657	KIN	35.877	0.818	0.242	3.376	0.000735	0.009633	yes

ENSG00000151746	BICD1	58.091	0.920	0.205	4.483	7.35E-06	0.000189	yes
ENSG00000152464	RPP38	38.179	0.801	0.227	3.536	0.000407	0.006041	yes
ENSG00000152778	IFIT5	23.241	1.099	0.277	3.973	7.11E-05	0.001394	yes
ENSG00000153827	TRIP12	512.769	0.785	0.092	8.504	1.83E-17	2.85E-15	yes
ENSG00000154027	AK5	28.914	2.608	0.300	8.696	3.45E-18	5.85E-16	yes
ENSG00000154127	UBASH3B	39.175	1.666	0.448	3.717	0.000201	0.003325	yes
ENSG00000154133	ROBO4	29.565	1.488	0.257	5.798	6.73E-09	3.71E-07	yes
ENSG00000154134	ROBO3	34.176	1.290	0.252	5.113	3.17E-07	1.15E-05	yes
ENSG00000154237	LRRK1	27.756	0.973	0.314	3.101	0.001927	0.02123	yes
ENSG00000154263	ABCA10	36.405	4.149	0.305	13.604	3.77E-42	2.62E-39	yes
ENSG00000154265	ABCA5	15.330	1.411	0.333	4.241	2.22E-05	0.000503	yes
ENSG00000154874	CCDC144B	13.993	1.160	0.328	3.538	0.000404	0.006017	yes
ENSG00000155158	TTC39B	19.739	0.913	0.295	3.097	0.001957	0.021453	yes
ENSG00000155744	FAM126B	40.289	0.714	0.234	3.059	0.002223	0.023582	yes
ENSG00000156011	PSD3	103.303	1.644	0.176	9.348	8.91E-21	1.96E-18	yes
ENSG00000156273	BACH1	36.013	1.595	0.229	6.955	3.52E-12	3.23E-10	yes
ENSG00000156374	PCGF6	16.481	1.060	0.345	3.072	0.002128	0.022868	yes
ENSG00000156384	SFR1	26.567	1.099	0.253	4.339	1.43E-05	0.000341	yes
ENSG00000156453	PCDH1	96.811	2.604	0.228	11.424	3.19E-30	1.38E-27	yes
ENSG00000156521	TYSND1	32.277	1.259	0.244	5.166	2.39E-07	8.85E-06	yes
ENSG00000156535	CD109	69.154	0.708	0.199	3.561	0.00037	0.005593	yes
ENSG00000156587	UBE2L6	28.588	1.147	0.254	4.519	6.21E-06	0.000164	yes
ENSG00000156599	ZDHHC5	85.511	0.707	0.188	3.761	0.000169	0.002866	yes
ENSG00000156603	MED19	74.429	0.850	0.193	4.398	1.09E-05	0.000267	yes
ENSG00000157483	MYO1E	69.543	0.737	0.174	4.240	2.23E-05	0.000503	yes
ENSG00000158604	TMED4	110.474	0.747	0.152	4.927	8.33E-07	2.78E-05	yes
ENSG00000158805	ZNF276	31.785	0.745	0.262	2.841	0.004496	0.040457	yes
ENSG00000159110	IFNAR2	24.908	0.918	0.280	3.274	0.001059	0.012981	yes
ENSG00000159128	IFNGR2	112.200	0.869	0.235	3.693	0.000222	0.003609	yes
ENSG00000159200	RCAN1	161.447	2.204	0.148	14.910	2.86E-50	4.96E-47	yes
ENSG00000159579	RSPRY1	75.458	0.726	0.185	3.918	8.92E-05	0.001702	yes
ENSG00000160199	PKNOX1	30.043	0.794	0.270	2.945	0.003226	0.031193	yes
ENSG00000160285	LSS	25.347	0.721	0.244	2.950	0.003176	0.030776	yes
ENSG00000161243	FBXO27	111.710	2.256	0.289	7.798	6.29E-15	7.77E-13	yes
ENSG00000161671	EMC10	47.481	1.051	0.302	3.487	0.000489	0.006929	yes
ENSG00000162302	RPS6KA4	29.971	1.196	0.297	4.033	5.50E-05	0.001115	yes
ENSG00000162366	PDZK1IP1	29.958	2.812	0.304	9.256	2.11E-20	4.52E-18	yes
ENSG00000162599	NFIA	52.397	1.223	0.265	4.612	3.98E-06	0.000111	yes
ENSG00000162614	NEXN	25.992	0.982	0.356	2.758	0.005822	0.049045	yes
ENSG00000162733	DDR2	96.566	0.764	0.174	4.380	1.19E-05	0.000287	yes
ENSG00000162772	ATF3	50.449	1.073	0.333	3.223	0.00127	0.01497	yes
ENSG00000163110	PDLIM5	165.656	0.773	0.115	6.725	1.76E-11	1.50E-09	yes
ENSG00000163249	CCNYL1	55.337	0.868	0.239	3.637	0.000275	0.004334	yes

ENSG00000163322	ABRAXAS1	14.582	1.313	0.407	3.228	0.001244	0.014765	yes
ENSG00000163347	CLDN1	81.998	1.824	0.182	10.029	1.14E-23	3.17E-21	yes
ENSG00000163359	COL6A3	40.723	0.847	0.207	4.093	4.26E-05	0.000897	yes
ENSG00000163565	IFI16	45.342	1.884	0.338	5.571	2.53E-08	1.19E-06	yes
ENSG00000163661	PTX3	49.016	3.401	0.248	13.734	6.32E-43	4.88E-40	yes
ENSG00000163735	CXCL5	57.101	2.909	0.221	13.181	1.12E-39	6.77E-37	yes
ENSG00000163738	MTHFD2L	31.123	1.132	0.261	4.343	1.40E-05	0.000334	yes
ENSG00000163739	CXCL1	39.192	3.169	0.266	11.926	8.65E-33	4.29E-30	yes
ENSG00000163762	TM4SF18	46.887	2.128	0.224	9.484	2.44E-21	5.56E-19	yes
ENSG00000163840	DTX3L	118.707	0.762	0.134	5.681	1.34E-08	6.64E-07	yes
ENSG00000164056	SPRY1	56.897	2.246	0.251	8.964	3.14E-19	6.23E-17	yes
ENSG00000164430	CGAS	63.913	0.930	0.221	4.197	2.70E-05	0.000599	yes
ENSG00000164442	CITED2	43.511	1.804	0.381	4.732	2.23E-06	6.62E-05	yes
ENSG00000164543	STK17A	150.731	0.934	0.125	7.475	7.73E-14	8.88E-12	yes
ENSG00000164626	KCNK5	25.674	0.876	0.264	3.321	0.000897	0.01129	yes
ENSG00000164692	COL1A2	39.909	1.283	0.297	4.321	1.55E-05	0.000364	yes
ENSG00000165030	NFIL3	18.286	1.458	0.498	2.925	0.00344	0.032918	yes
ENSG00000165102	HGSNAT	33.155	0.753	0.221	3.404	0.000665	0.008909	yes
ENSG00000165389	SPTSSA	86.954	1.013	0.233	4.355	1.33E-05	0.000318	yes
ENSG00000165507	DEPP1	13.012	2.078	0.404	5.139	2.77E-07	1.01E-05	yes
ENSG00000165512	ZNF22	53.863	0.767	0.198	3.869	0.000109	0.002018	yes
ENSG00000165527	ARF6	204.730	0.761	0.109	7.012	2.34E-12	2.23E-10	yes
ENSG00000165644	COMTD1	20.239	1.019	0.344	2.959	0.003083	0.030052	yes
ENSG00000165949	IFI27	12.425	2.195	0.735	2.986	0.002822	0.02825	yes
ENSG00000165996	HACD1	30.115	0.789	0.242	3.254	0.001138	0.013675	yes
ENSG00000166326	TRIM44	156.635	0.792	0.153	5.164	2.42E-07	8.93E-06	yes
ENSG00000166333	ILK	50.123	0.757	0.219	3.462	0.000536	0.007448	yes
ENSG00000166340	TPP1	99.954	1.213	0.171	7.098	1.27E-12	1.26E-10	yes
ENSG00000166401	SERPINB8	28.698	0.774	0.273	2.836	0.004563	0.040918	yes
ENSG00000166432	ZMAT1	25.245	0.914	0.297	3.076	0.002101	0.022685	yes
ENSG00000166436	TRIM66	19.773	1.412	0.374	3.771	0.000162	0.002785	yes
ENSG00000166510	CCDC68	45.340	1.931	0.222	8.714	2.94E-18	5.16E-16	yes
ENSG00000166548	TK2	42.832	1.160	0.197	5.871	4.33E-09	2.47E-07	yes
ENSG00000166575	TMEM135	24.354	0.989	0.293	3.374	0.00074	0.009669	yes
ENSG00000166750	SLFN5	65.462	1.200	0.200	5.987	2.13E-09	1.29E-07	yes
ENSG00000166822	TMEM170A	68.053	0.779	0.185	4.211	2.54E-05	0.000565	yes
ENSG00000166920	C15orf48	52.598	3.819	0.863	4.425	9.65E-06	0.000241	yes
ENSG00000167601	AXL	245.721	1.019	0.130	7.831	4.85E-15	6.07E-13	yes
ENSG00000167755	KLK6	47.867	0.879	0.282	3.112	0.001858	0.020666	yes
ENSG00000167767	KRT80	71.278	0.724	0.195	3.714	0.000204	0.003361	yes
ENSG00000167772	ANGPTL4	29.969	2.170	0.296	7.328	2.34E-13	2.54E-11	yes
ENSG00000167972	ABCA3	13.046	1.510	0.419	3.608	0.000309	0.004804	yes
ENSG00000167996	FTH1	1149.604	0.865	0.176	4.902	9.48E-07	3.08E-05	yes

ENSG00000168040	FADD	45.188	0.715	0.218	3.271	0.001071	0.013095	yes
ENSG00000168283	BMI1	16.117	1.971	0.530	3.721	0.000198	0.003286	yes
ENSG00000168461	RAB31	170.245	0.873	0.125	7.006	2.45E-12	2.31E-10	yes
ENSG00000168610	STAT3	219.845	0.706	0.156	4.524	6.07E-06	0.000161	yes
ENSG00000168876	ANKRD49	40.453	0.752	0.267	2.821	0.004792	0.042439	yes
ENSG00000169247	SH3TC2	20.910	1.339	0.297	4.503	6.71E-06	0.000175	yes
ENSG00000169252	ADRB2	18.415	3.025	1.082	2.795	0.005188	0.045188	yes
ENSG00000169429	CXCL8	28.413	3.027	0.437	6.925	4.37E-12	3.92E-10	yes
ENSG00000169504	CLIC4	112.584	0.844	0.186	4.547	5.45E-06	0.000146	yes
ENSG00000169871	TRIM56	68.286	0.748	0.192	3.890	0.0001	0.001881	yes
ENSG00000169908	TM4SF1	499.184	0.804	0.090	8.934	4.10E-19	7.84E-17	yes
ENSG00000170035	UBE2E3	82.739	1.283	0.182	7.032	2.03E-12	1.96E-10	yes
ENSG00000170421	KRT8	371.049	0.985	0.153	6.421	1.35E-10	1.01E-08	yes
ENSG00000170456	DENND5B	84.317	1.010	0.179	5.627	1.83E-08	8.92E-07	yes
ENSG00000170485	NPAS2	54.788	3.160	0.282	11.213	3.52E-29	1.44E-26	yes
ENSG00000170500	LONRF2	34.036	0.961	0.275	3.496	0.000472	0.006706	yes
ENSG00000170525	PFKFB3	30.251	0.957	0.266	3.602	0.000316	0.004879	yes
ENSG00000170677	SOCS6	106.887	1.106	0.158	7.012	2.35E-12	2.23E-10	yes
ENSG00000170689	HOXB9	64.610	1.783	0.205	8.700	3.33E-18	5.72E-16	yes
ENSG00000170802	FOXN2	47.028	0.867	0.212	4.092	4.28E-05	0.0009	yes
ENSG00000170955	CAVIN3	26.996	1.145	0.271	4.228	2.36E-05	0.000529	yes
ENSG00000171316	CHD7	136.525	1.106	0.155	7.115	1.12E-12	1.14E-10	yes
ENSG00000171365	CLCN5	65.549	0.795	0.178	4.479	7.50E-06	0.000192	yes
ENSG00000171517	LPAR3	23.627	0.825	0.279	2.959	0.003084	0.030052	yes
ENSG00000171617	ENC1	94.379	0.782	0.178	4.387	1.15E-05	0.000279	yes
ENSG00000172059	KLF11	16.531	1.527	0.519	2.942	0.003266	0.031509	yes
ENSG00000172137	CALB2	70.722	2.691	0.184	14.595	3.01E-48	3.48E-45	yes
ENSG00000172183	ISG20	22.432	0.921	0.308	2.990	0.002789	0.027975	yes
ENSG00000172216	CEBPB	103.452	1.103	0.216	5.111	3.20E-07	1.16E-05	yes
ENSG00000172269	DPAGT1	42.679	0.915	0.250	3.662	0.00025	0.003976	yes
ENSG00000172432	GTPBP2	22.519	1.468	0.444	3.303	0.000957	0.011945	yes
ENSG00000173193	PARP14	108.206	1.498	0.151	9.904	3.99E-23	1.07E-20	yes
ENSG00000173559	NABP1	48.877	0.975	0.243	4.013	6.00E-05	0.001203	yes
ENSG00000173801	JUP	76.921	0.966	0.351	2.753	0.005914	0.04945	yes
ENSG00000173848	NET1	106.924	0.912	0.148	6.142	8.17E-10	5.33E-08	yes
ENSG00000173917	HOXB2	24.613	1.322	0.377	3.509	0.00045	0.006507	yes
ENSG00000174276	ZNHIT2	12.227	1.422	0.451	3.149	0.001639	0.018648	yes
ENSG00000174456	C12orf76	12.824	1.377	0.481	2.862	0.004208	0.038388	yes
ENSG00000174804	FZD4	11.404	2.201	0.426	5.163	2.44E-07	8.98E-06	yes
ENSG00000175048	ZDHHC14	92.811	1.937	0.179	10.820	2.78E-27	9.65E-25	yes
ENSG00000175161	CADM2	27.292	1.367	0.271	5.052	4.37E-07	1.54E-05	yes
ENSG00000175220	ARHGAP1	49.748	0.819	0.215	3.817	0.000135	0.002408	yes
ENSG00000175224	ATG13	55.017	0.754	0.195	3.859	0.000114	0.00208	yes

ENSG00000175471	MCTP1	39.289	2.702	0.363	7.441	9.99E-14	1.12E-11	yes
ENSG00000175505	CLCF1	24.180	1.243	0.292	4.252	2.11E-05	0.00048	yes
ENSG00000175745	NR2F1	439.988	1.974	0.133	14.887	3.98E-50	5.53E-47	yes
ENSG00000177283	FZD8	14.024	1.239	0.380	3.261	0.001109	0.013373	yes
ENSG00000177606	JUN	442.895	1.246	0.087	14.386	6.35E-47	5.88E-44	yes
ENSG00000177946	CENPBD1	25.824	0.886	0.299	2.961	0.003067	0.029924	yes
ENSG00000178202	POGLUT3	35.090	1.180	0.256	4.616	3.91E-06	0.000109	yes
ENSG00000178397	FAM220A	40.725	0.774	0.227	3.405	0.000661	0.008861	yes
ENSG00000179431	FJX1	34.552	0.800	0.262	3.050	0.002287	0.02402	yes
ENSG00000179833	SERTAD2	51.100	1.017	0.264	3.846	0.00012	0.002173	yes
ENSG00000180263	FGD6	56.912	1.607	0.230	7.000	2.56E-12	2.41E-10	yes
ENSG00000180537	RNF182	24.381	1.778	0.313	5.674	1.40E-08	6.91E-07	yes
ENSG00000180914	OXTR	12.378	1.824	0.474	3.848	0.000119	0.002162	yes
ENSG00000180917	CMTR2	55.187	0.816	0.187	4.367	1.26E-05	0.000303	yes
ENSG00000181381	DDX60L	45.840	1.212	0.199	6.081	1.19E-09	7.61E-08	yes
ENSG00000181467	RAP2B	166.948	1.856	0.169	11.003	3.70E-28	1.39E-25	yes
ENSG00000181649	PHLDA2	293.590	1.086	0.133	8.155	3.50E-16	4.82E-14	yes
ENSG00000182568	SATB1	14.531	0.949	0.328	2.890	0.003858	0.036141	yes
ENSG00000182742	HOXB4	44.562	1.846	0.354	5.214	1.85E-07	7.01E-06	yes
ENSG00000182919	C11orf54	50.427	0.788	0.225	3.505	0.000457	0.006549	yes
ENSG00000183691	NOG	27.312	0.838	0.275	3.050	0.002288	0.02402	yes
ENSG00000183765	CHEK2	27.492	0.986	0.301	3.277	0.001048	0.01286	yes
ENSG00000184584	STING1	14.626	1.745	0.448	3.898	9.69E-05	0.001824	yes
ENSG00000184743	ATL3	94.323	1.116	0.204	5.460	4.77E-08	2.08E-06	yes
ENSG00000185112	FAM43A	57.068	0.996	0.283	3.521	0.000429	0.006283	yes
ENSG00000185306	C12orf56	14.160	1.307	0.366	3.570	0.000357	0.005418	yes
ENSG00000185745	IFIT1	36.512	2.001	0.248	8.075	6.73E-16	9.08E-14	yes
ENSG00000185869	ZNF829	10.645	2.348	0.670	3.504	0.000458	0.006557	yes
ENSG00000186635	ARAP1	28.574	0.865	0.281	3.080	0.002069	0.022454	yes
ENSG00000187134	AKR1C1	32.519	0.822	0.276	2.976	0.002921	0.029005	yes
ENSG00000187608	ISG15	103.432	1.760	0.175	10.029	1.14E-23	3.17E-21	yes
ENSG00000188227	ZNF793	15.157	1.440	0.380	3.784	0.000154	0.002676	yes
ENSG00000188290	HES4	33.677	1.037	0.295	3.517	0.000436	0.006358	yes
ENSG00000188313	PLSCR1	42.327	1.577	0.235	6.704	2.02E-11	1.69E-09	yes
ENSG00000188549	CCDC9B	42.417	1.102	0.228	4.834	1.34E-06	4.19E-05	yes
ENSG00000189046	ALKBH2	38.540	0.787	0.264	2.978	0.002901	0.02887	yes
ENSG00000189060	H1-0	103.915	0.929	0.179	5.185	2.16E-07	8.10E-06	yes
ENSG00000189143	CLDN4	249.231	0.792	0.143	5.530	3.21E-08	1.45E-06	yes
ENSG00000189223	PAX8-AS1	23.365	1.673	0.308	5.429	5.67E-08	2.40E-06	yes
ENSG00000196116	TDRD7	10.995	1.450	0.503	2.884	0.003932	0.036543	yes
ENSG00000196141	SPATS2L	392.103	1.651	0.110	15.000	7.37E-51	1.46E-47	yes
ENSG00000196182	STK40	54.856	0.906	0.190	4.775	1.79E-06	5.46E-05	yes
ENSG00000196437	ZNF569	16.428	1.203	0.414	2.903	0.003697	0.034805	yes

ENSG00000196458	ZNF605	33.796	0.810	0.275	2.948	0.003199	0.030976	yes
ENSG00000196526	AFAP1	79.723	1.331	0.178	7.457	8.83E-14	9.97E-12	yes
ENSG00000196850	PPTC7	50.740	0.900	0.240	3.748	0.000178	0.002994	yes
ENSG00000196950	SLC39A10	164.831	0.729	0.137	5.340	9.27E-08	3.75E-06	yes
ENSG00000197121	PGAP1	33.980	2.352	0.304	7.745	9.52E-15	1.14E-12	yes
ENSG00000197586	ENTPD6	66.082	1.279	0.207	6.184	6.23E-10	4.18E-08	yes
ENSG00000197696	NMB	18.861	0.958	0.287	3.334	0.000856	0.010873	yes
ENSG00000197702	PARVA	38.075	0.746	0.254	2.944	0.003245	0.031327	yes
ENSG00000197943	PLCG2	13.113	1.427	0.428	3.338	0.000842	0.010741	yes
ENSG00000198742	SMURF1	46.395	1.230	0.245	5.017	5.24E-07	1.81E-05	yes
ENSG00000198959	TGM2	180.267	1.640	0.138	11.895	1.25E-32	6.01E-30	yes
ENSG00000204116	CHIC1	11.528	2.546	0.810	3.145	0.001663	0.018879	yes
ENSG00000204264	PSMB8	28.371	1.594	0.397	4.017	5.89E-05	0.001184	yes
ENSG00000205413	SAMD9	74.455	2.857	0.292	9.799	1.14E-22	2.94E-20	yes
ENSG00000206560	ANKRD28	56.949	0.719	0.198	3.626	0.000287	0.004496	yes
ENSG00000213186	TRIM59	22.146	2.506	0.909	2.757	0.005832	0.049045	yes
ENSG00000213465	ARL2	82.328	0.921	0.171	5.374	7.70E-08	3.17E-06	yes
ENSG00000214900	LINC01588	19.829	1.517	0.309	4.905	9.36E-07	3.04E-05	yes
ENSG00000215712	TMEM242	14.341	1.594	0.418	3.812	0.000138	0.002444	yes
ENSG00000215788	TNFRSF25	23.766	0.965	0.288	3.354	0.000796	0.010287	yes
ENSG00000221869	CEBPD	36.952	2.462	0.288	8.535	1.40E-17	2.21E-15	yes
ENSG00000221963	APOL6	42.562	1.202	0.217	5.540	3.03E-08	1.38E-06	yes
ENSG00000224099	n/a	21.881	2.072	0.398	5.200	1.99E-07	7.49E-06	yes
ENSG00000227036	LINC00511	15.810	1.958	0.669	2.925	0.003448	0.032966	yes
ENSG00000230453	ANKRD18B	14.998	3.373	0.484	6.970	3.16E-12	2.93E-10	yes
ENSG00000234608	MAPKAPK5-A[illegible]	18.717	1.047	0.349	3.001	0.002688	0.027243	yes
ENSG00000234745	HLA-B	440.335	0.780	0.145	5.369	7.91E-08	3.25E-06	yes
ENSG00000235884	LINC00941	12.446	2.022	0.505	4.002	6.28E-05	0.001248	yes
ENSG00000237187	NR2F1-AS1	24.194	1.912	0.331	5.782	7.39E-09	4.00E-07	yes
ENSG00000240694	PNMA2	22.303	1.274	0.357	3.566	0.000363	0.005502	yes
ENSG00000241685	ARPC1A	87.544	0.837	0.175	4.769	1.85E-06	5.61E-05	yes
ENSG00000242498	ARPIN	12.085	1.617	0.481	3.363	0.00077	0.009986	yes
ENSG00000245694	CRNDE	33.369	0.795	0.279	2.854	0.004316	0.039167	yes
ENSG00000249992	TMEM158	79.393	0.702	0.210	3.345	0.000824	0.010557	yes
ENSG00000250072	SH3TC2-DT	61.923	1.424	0.177	8.028	9.87E-16	1.31E-13	yes
ENSG00000251194	n/a	11.750	1.009	0.361	2.794	0.005211	0.04528	yes
ENSG00000257178	n/a	19.223	1.698	0.447	3.801	0.000144	0.002538	yes
ENSG00000258655	ARHGAP5-AS1	11.456	2.814	0.505	5.568	2.58E-08	1.21E-06	yes
ENSG00000260549	MT1L	70.222	1.577	0.339	4.646	3.38E-06	9.57E-05	yes
ENSG00000263465	SRSF8	41.357	0.782	0.254	3.081	0.00206	0.022413	yes
ENSG00000271503	CCL5	10.729	3.058	0.487	6.277	3.45E-10	2.45E-08	yes
ENSG00000272763	n/a	20.300	2.911	0.654	4.454	8.41E-06	0.000213	yes
ENSG00000275342	PRAG1	27.782	1.732	0.356	4.866	1.14E-06	3.64E-05	yes

ENSG00000280138	n/a	13.370	1.477	0.529	2.791	0.005253	0.045435	yes
ENSG00000000460	C1orf112	17.316	-0.717	0.341	-2.103	0.035505	0.177567	yes
ENSG00000003096	KLHL13	10.017	-0.876	0.498	-1.758	0.078691	0.294778	yes
ENSG00000005513	SOX8	18.266	-1.118	0.302	-3.700	0.000216	0.003527	yes
ENSG00000005882	PDK2	15.652	-0.742	0.531	-1.398	0.162139	0.446761	yes
ENSG00000006016	CRLF1	36.219	-1.590	0.293	-5.436	5.45E-08	2.32E-06	yes
ENSG00000011028	MRC2	40.155	-0.739	0.216	-3.418	0.000631	0.008517	yes
ENSG00000011332	DPF1	13.729	-0.881	0.355	-2.479	0.013167	0.088461	yes
ENSG00000013810	TACC3	177.724	-0.880	0.129	-6.812	9.63E-12	8.31E-10	yes
ENSG00000014914	MTMR11	13.052	-0.914	0.382	-2.390	0.016853	0.105573	yes
ENSG00000022567	SLC45A4	19.245	-0.901	0.355	-2.540	0.011086	0.07815	yes
ENSG00000023171	GRAMD1B	25.689	-1.605	0.253	-6.345	2.22E-10	1.62E-08	yes
ENSG00000024526	DEPDC1	91.189	-0.978	0.172	-5.684	1.32E-08	6.57E-07	yes
ENSG00000025770	NCAPH2	26.115	-0.749	0.289	-2.589	0.009633	0.070552	yes
ENSG00000027001	MIPEP	11.781	-1.080	0.363	-2.975	0.002926	0.029019	yes
ENSG00000032742	IFT88	36.233	-1.094	0.236	-4.629	3.67E-06	0.000103	yes
ENSG00000035499	DEPDC1B	52.979	-0.932	0.234	-3.974	7.05E-05	0.001386	yes
ENSG00000037965	HOXC8	26.348	-0.808	0.315	-2.562	0.010406	0.074643	yes
ENSG00000039123	MTREX	315.616	-0.714	0.138	-5.178	2.25E-07	8.42E-06	yes
ENSG00000040275	SPDL1	216.233	-0.808	0.133	-6.077	1.22E-09	7.77E-08	yes
ENSG00000040608	RTN4R	19.390	-0.832	0.317	-2.627	0.00862	0.064881	yes
ENSG00000046604	DSG2	80.315	-0.951	0.192	-4.951	7.39E-07	2.50E-05	yes
ENSG00000047365	ARAP2	26.135	-1.531	1.675	-0.914	0.360606	0.672246	yes
ENSG00000047634	SCML1	10.349	-1.594	1.058	-1.507	0.131905	0.400589	yes
ENSG00000052126	PLEKHA5	42.674	-0.779	0.253	-3.077	0.002088	0.022615	yes
ENSG00000054277	OPN3	19.376	-0.790	0.391	-2.017	0.043677	0.204328	yes
ENSG00000055163	CYFIP2	12.939	-0.946	0.352	-2.688	0.007193	0.056879	yes
ENSG00000058085	LAMC2	35.005	-1.910	1.220	-1.566	0.117315	0.374964	yes
ENSG00000058091	CDK14	10.484	-2.309	0.875	-2.638	0.00834	0.063336	yes
ENSG00000062038	CDH3	16.660	-1.914	1.236	-1.549	0.121471	0.382701	yes
ENSG00000064666	CNN2	148.689	-0.970	0.140	-6.939	3.96E-12	3.57E-10	yes
ENSG00000065361	ERBB3	16.914	-1.121	0.419	-2.676	0.007458	0.058377	yes
ENSG00000065717	TLE2	39.585	-0.782	0.239	-3.274	0.001062	0.013013	yes
ENSG00000065989	PDE4A	17.122	-1.957	0.443	-4.419	9.92E-06	0.000246	yes
ENSG00000066583	ISOC1	27.047	-0.949	0.349	-2.720	0.006523	0.053371	yes
ENSG00000066629	EML1	11.549	-0.851	0.383	-2.222	0.026252	0.145087	yes
ENSG00000067798	NAV3	25.808	-1.613	0.318	-5.078	3.82E-07	1.36E-05	yes
ENSG00000068489	PRR11	301.358	-0.724	0.129	-5.619	1.92E-08	9.26E-07	yes
ENSG00000070814	TCOF1	240.185	-0.858	0.103	-8.345	7.13E-17	1.05E-14	yes
ENSG00000072571	HMMR	255.950	-0.982	0.091	-10.736	6.87E-27	2.33E-24	yes
ENSG00000072682	P4HA2	39.119	-0.838	0.216	-3.885	0.000102	0.001909	yes
ENSG00000073050	XRCC1	27.342	-0.723	0.274	-2.644	0.008191	0.062551	yes
ENSG00000074211	PPP2R2C	45.950	-1.160	0.202	-5.740	9.46E-09	4.89E-07	yes

ENSG00000074416	MGLL	39.075	-0.908	0.977	-0.929	0.352876	0.664523	yes
ENSG00000074855	ANO8	21.505	-0.707	0.292	-2.417	0.015654	0.100366	yes
ENSG00000075218	GTSE1	76.992	-0.743	0.172	-4.323	1.54E-05	0.000363	yes
ENSG00000075391	RASAL2	115.531	-1.614	0.160	-10.064	7.95E-24	2.30E-21	yes
ENSG00000075461	CACNG4	16.713	-2.081	0.396	-5.251	1.51E-07	5.85E-06	yes
ENSG00000075702	WDR62	18.573	-0.866	0.362	-2.392	0.016753	0.105135	yes
ENSG00000076382	SPAG5	53.531	-0.750	0.196	-3.819	0.000134	0.002387	yes
ENSG00000076662	ICAM3	11.392	-1.500	0.462	-3.247	0.001166	0.013966	yes
ENSG00000077152	UBE2T	51.482	-0.769	0.266	-2.885	0.003918	0.03651	yes
ENSG00000077782	FGFR1	75.197	-0.846	0.155	-5.473	4.42E-08	1.93E-06	yes
ENSG00000078401	EDN1	31.992	-0.868	0.545	-1.593	0.111179	0.362999	yes
ENSG00000079257	LXN	11.520	-1.757	0.911	-1.929	0.053705	0.2326	yes
ENSG00000079616	KIF22	91.445	-0.934	0.168	-5.553	2.81E-08	1.29E-06	yes
ENSG00000080824	HSP90AA1	5750.170	-0.703	0.064	-10.976	5.01E-28	1.78E-25	yes
ENSG00000082516	GEMIN5	131.756	-0.757	0.110	-6.866	6.62E-12	5.86E-10	yes
ENSG00000083838	ZNF446	11.585	-0.980	0.434	-2.261	0.023782	0.13503	yes
ENSG00000085662	AKR1B1	93.028	-0.884	0.144	-6.136	8.49E-10	5.51E-08	yes
ENSG00000085872	CHERP	80.801	-0.738	0.160	-4.603	4.17E-06	0.000115	yes
ENSG00000086200	IPO11	36.663	-0.797	0.227	-3.512	0.000445	0.006465	yes
ENSG00000087253	LPCAT2	25.343	-2.750	1.139	-2.414	0.01576	0.10086	yes
ENSG00000087510	TFAP2C	13.644	-0.799	0.515	-1.553	0.120519	0.381101	yes
ENSG00000087586	AURKA	238.063	-0.740	0.109	-6.795	1.08E-11	9.29E-10	yes
ENSG00000088305	DNMT3B	10.958	-1.483	0.390	-3.802	0.000144	0.002531	yes
ENSG00000088992	TESC	10.676	-1.629	0.409	-3.983	6.80E-05	0.001338	yes
ENSG00000089820	ARHGAP4	20.853	-0.914	0.983	-0.930	0.35249	0.664327	yes
ENSG00000090776	EFNB1	11.299	-0.958	0.385	-2.488	0.012852	0.087102	yes
ENSG00000090889	KIF4A	208.039	-0.716	0.111	-6.441	1.18E-10	8.90E-09	yes
ENSG00000090932	DLL3	104.509	-0.793	0.399	-1.988	0.046761	0.213017	yes
ENSG00000092853	CLSPN	141.170	-0.730	0.148	-4.919	8.70E-07	2.86E-05	yes
ENSG00000094804	CDC6	77.746	-0.715	0.166	-4.294	1.75E-05	0.000406	yes
ENSG00000094914	AAAS	30.350	-0.737	0.284	-2.598	0.009373	0.069383	yes
ENSG00000096092	TMEM14A	47.522	-0.801	0.250	-3.202	0.001366	0.015925	yes
ENSG00000100092	SH3BP1	18.062	-1.059	0.322	-3.285	0.00102	0.012581	yes
ENSG00000100116	GCAT	14.035	-0.919	0.347	-2.650	0.008039	0.06178	yes
ENSG00000100292	HMOX1	24.568	-0.777	0.319	-2.435	0.014899	0.096881	yes
ENSG00000100359	SGSM3	18.541	-0.749	0.359	-2.084	0.037136	0.183164	yes
ENSG00000100558	PLEK2	23.392	-0.989	1.121	-0.882	0.377515	0.68368	yes
ENSG00000100577	GSTZ1	18.356	-1.404	0.372	-3.773	0.000161	0.002769	yes
ENSG00000101098	RIMS4	20.572	-0.911	0.306	-2.972	0.002958	0.029235	yes
ENSG00000101333	PLCB4	88.337	-1.907	1.213	-1.573	0.115809	0.371778	yes
ENSG00000101335	MYL9	85.051	-1.056	0.160	-6.598	4.18E-11	3.32E-09	yes
ENSG00000102078	SLC25A14	16.903	-0.755	0.325	-2.326	0.020012	0.119896	yes
ENSG00000102543	CDADC1	14.256	-0.751	0.354	-2.118	0.034185	0.173917	yes

ENSG00000102554	KLF5	20.944	-0.888	0.497	-1.787	0.073865	0.283869	yes
ENSG00000103260	METRN	44.496	-1.037	0.322	-3.218	0.001291	0.01517	yes
ENSG00000103540	CCP110	82.302	-0.729	0.177	-4.122	3.76E-05	0.000801	yes
ENSG00000103653	CSK	24.531	-1.049	0.274	-3.824	0.000131	0.00235	yes
ENSG00000103876	FAH	16.418	-0.946	0.304	-3.108	0.001882	0.02087	yes
ENSG00000104147	OIP5	32.791	-0.704	0.305	-2.311	0.020836	0.123135	yes
ENSG00000104369	JPH1	14.344	-1.065	0.590	-1.804	0.07117	0.277061	yes
ENSG00000104611	SH2D4A	22.176	-1.178	1.327	-0.887	0.374892	0.682607	yes
ENSG00000104738	MCM4	265.707	-0.838	0.126	-6.635	3.24E-11	2.62E-09	yes
ENSG00000104763	ASAH1	21.866	-0.772	0.324	-2.384	0.017146	0.106973	yes
ENSG00000104853	CLPTM1	48.791	-0.799	0.280	-2.850	0.004375	0.03963	yes
ENSG00000104889	RNASEH2A	59.822	-0.788	0.189	-4.161	3.17E-05	0.000691	yes
ENSG00000104967	NOVA2	16.460	-0.711	0.327	-2.170	0.02998	0.158742	yes
ENSG00000105011	ASF1B	61.695	-0.707	0.219	-3.228	0.001247	0.014771	yes
ENSG00000105048	TNNT1	190.949	-0.700	0.122	-5.719	1.07E-08	5.48E-07	yes
ENSG00000105290	APLP1	19.012	-0.718	0.341	-2.108	0.034996	0.176171	yes
ENSG00000105419	MEIS3	31.176	-1.047	0.210	-4.976	6.48E-07	2.21E-05	yes
ENSG00000105486	LIG1	36.635	-0.862	0.261	-3.304	0.000952	0.011908	yes
ENSG00000105516	DBP	25.735	-0.866	0.318	-2.726	0.006411	0.052668	yes
ENSG00000105520	PLPPR2	17.950	-1.296	0.366	-3.536	0.000406	0.006028	yes
ENSG00000105605	CACNG7	19.515	-2.083	0.372	-5.595	2.21E-08	1.05E-06	yes
ENSG00000105662	CRTC1	11.701	-0.705	0.389	-1.814	0.069664	0.273888	yes
ENSG00000105696	TMEM59L	20.464	-1.213	0.287	-4.233	2.31E-05	0.000519	yes
ENSG00000105722	ERF	58.182	-0.856	0.307	-2.784	0.005363	0.046048	yes
ENSG00000106484	MEST	13.910	-1.330	0.794	-1.674	0.094043	0.327805	yes
ENSG00000106546	AHR	34.028	-2.497	0.379	-6.590	4.41E-11	3.47E-09	yes
ENSG00000107331	ABCA2	14.591	-0.798	0.366	-2.180	0.029265	0.156342	yes
ENSG00000107738	VSIR	13.400	-0.952	0.364	-2.615	0.008911	0.066602	yes
ENSG00000107821	KAZALD1	10.252	-1.448	0.502	-2.884	0.003923	0.036513	yes
ENSG00000108813	DLX4	10.558	-0.730	0.376	-1.944	0.051867	0.227834	yes
ENSG00000108852	MPP2	16.627	-1.472	0.340	-4.324	1.53E-05	0.000362	yes
ENSG00000109016	DHRS7B	13.020	-0.851	0.396	-2.146	0.031901	0.16663	yes
ENSG00000109321	AREG	454.603	-1.048	0.110	-9.538	1.45E-21	3.37E-19	yes
ENSG00000109466	KLHL2	12.159	-0.952	0.580	-1.640	0.101021	0.342817	yes
ENSG00000109501	WFS1	14.028	-1.130	0.387	-2.922	0.003482	0.033203	yes
ENSG00000110723	EXPH5	26.007	-0.951	0.545	-1.743	0.08128	0.299473	yes
ENSG00000111907	TPD52L1	12.488	-0.932	0.533	-1.749	0.08037	0.297934	yes
ENSG00000112576	CCND3	31.706	-0.917	0.265	-3.462	0.000537	0.00745	yes
ENSG00000112742	TTK	86.223	-0.970	0.178	-5.445	5.18E-08	2.22E-06	yes
ENSG00000112984	KIF20A	59.365	-1.200	0.204	-5.892	3.81E-09	2.20E-07	yes
ENSG00000113580	NR3C1	134.069	-0.829	0.144	-5.748	9.04E-09	4.74E-07	yes
ENSG00000113645	WWC1	88.862	-0.751	0.204	-3.689	0.000225	0.003658	yes
ENSG00000114405	C3orf14	12.508	-2.029	1.466	-1.384	0.16631	0.453477	yes

ENSG00000115289	PCGF1	10.325	-0.900	0.527	-1.707	0.087841	0.314227	yes
ENSG00000115738	ID2	44.003	-1.171	0.266	-4.400	1.08E-05	0.000266	yes
ENSG00000115828	QPCT	14.060	-1.316	0.362	-3.636	0.000277	0.004354	yes
ENSG00000116191	RALGPS2	52.993	-0.827	0.227	-3.643	0.00027	0.004254	yes
ENSG00000116729	WLS	26.711	-1.162	0.301	-3.861	0.000113	0.002071	yes
ENSG00000116962	NID1	43.832	-1.228	0.636	-1.931	0.053531	0.232207	yes
ENSG00000117115	PADI2	43.220	-1.454	1.565	-0.929	0.35286	0.664523	yes
ENSG00000117399	CDC20	204.479	-0.800	0.112	-7.133	9.81E-13	1.01E-10	yes
ENSG00000117586	TNFSF4	15.978	-1.499	0.417	-3.591	0.00033	0.005046	yes
ENSG00000117592	PRDX6	357.748	-0.951	0.079	-12.012	3.08E-33	1.58E-30	yes
ENSG00000117650	NEK2	85.914	-1.294	0.171	-7.588	3.24E-14	3.82E-12	yes
ENSG00000117724	CENPF	1443.623	-0.912	0.064	-14.259	3.94E-46	3.43E-43	yes
ENSG00000118193	KIF14	153.807	-0.710	0.128	-5.561	2.68E-08	1.24E-06	yes
ENSG00000119782	FKBP1B	10.765	-1.737	0.515	-3.374	0.000741	0.009681	yes
ENSG00000120334	CENPL	34.968	-0.971	0.254	-3.823	0.000132	0.002356	yes
ENSG00000120694	HSPH1	865.135	-0.808	0.102	-7.888	3.07E-15	3.91E-13	yes
ENSG00000120885	CLU	188.876	-0.703	0.139	-5.060	4.20E-07	1.48E-05	yes
ENSG00000120913	PDLIM2	84.276	-0.808	0.262	-3.083	0.002052	0.022339	yes
ENSG00000121152	NCAPH	73.047	-0.960	0.194	-4.943	7.68E-07	2.58E-05	yes
ENSG00000121211	MND1	38.411	-0.824	0.233	-3.540	0.000401	0.005995	yes
ENSG00000122335	SERAC1	11.986	-0.868	0.403	-2.157	0.031025	0.163097	yes
ENSG00000122952	ZWINT	55.869	-0.812	0.191	-4.247	2.17E-05	0.000491	yes
ENSG00000123297	TSFM	28.171	-0.706	0.271	-2.604	0.009216	0.068367	yes
ENSG00000123485	HJURP	173.850	-0.790	0.118	-6.705	2.02E-11	1.69E-09	yes
ENSG00000124191	TOX2	54.233	-0.876	0.339	-2.586	0.009697	0.070762	yes
ENSG00000124313	IQSEC2	10.075	-0.723	0.443	-1.633	0.102517	0.345727	yes
ENSG00000125144	MT1G	15.969	-3.201	0.606	-5.278	1.31E-07	5.12E-06	yes
ENSG00000126822	PLEKHG3	31.102	-0.924	0.321	-2.877	0.004019	0.037128	yes
ENSG00000127220	ABHD8	10.510	-0.766	0.439	-1.746	0.080809	0.298925	yes
ENSG00000127399	LRRC61	17.119	-2.419	1.569	-1.542	0.123084	0.385599	yes
ENSG00000127564	PKMYT1	49.425	-0.727	0.207	-3.504	0.000459	0.00657	yes
ENSG00000127586	CHTF18	17.880	-0.759	0.341	-2.228	0.025897	0.143807	yes
ENSG00000128581	IFT22	37.694	-0.835	0.227	-3.682	0.000232	0.003735	yes
ENSG00000128708	HAT1	62.487	-0.796	0.204	-3.912	9.15E-05	0.001737	yes
ENSG00000129354	AP1M2	18.392	-1.170	1.331	-0.879	0.379475	0.68573	yes
ENSG00000130600	H19	277.993	-1.453	0.246	-5.900	3.63E-09	2.12E-07	yes
ENSG00000131188	PRR7	17.222	-0.902	0.348	-2.588	0.00966	0.070677	yes
ENSG00000131495	NDUFA2	138.899	-0.734	0.127	-5.790	7.05E-09	3.84E-07	yes
ENSG00000131747	TOP2A	2194.679	-0.910	0.091	-9.973	2.00E-23	5.44E-21	yes
ENSG00000132122	SPATA6	12.746	-1.574	0.384	-4.103	4.07E-05	0.00086	yes
ENSG00000132326	PER2	24.147	-0.720	0.327	-2.203	0.027613	0.149965	yes
ENSG00000132563	REEP2	19.295	-0.731	0.284	-2.570	0.010161	0.073415	yes
ENSG00000132879	FBXO44	31.521	-0.821	0.246	-3.338	0.000843	0.010741	yes

ENSG00000133119	RFC3	119.447	-0.800	0.132	-6.071	1.27E-09	8.00E-08	yes
ENSG00000133131	MORC4	56.898	-0.858	0.202	-4.254	2.10E-05	0.000477	yes
ENSG00000133134	BEX2	38.878	-1.101	0.270	-4.075	4.60E-05	0.00095	yes
ENSG00000133265	HSPBP1	51.251	-0.807	0.246	-3.282	0.001031	0.012693	yes
ENSG00000134057	CCNB1	391.313	-0.841	0.094	-8.938	3.95E-19	7.73E-17	yes
ENSG00000134138	MEIS2	57.569	-1.471	0.219	-6.705	2.02E-11	1.69E-09	yes
ENSG00000134146	DPH6	49.999	-1.552	0.207	-7.482	7.30E-14	8.45E-12	yes
ENSG00000134668	SPOCD1	36.352	-1.097	0.194	-5.668	1.44E-08	7.11E-07	yes
ENSG00000134709	HOOK1	18.913	-1.531	1.676	-0.914	0.360934	0.672589	yes
ENSG00000134755	DSC2	18.487	-1.392	0.678	-2.053	0.040028	0.192105	yes
ENSG00000135083	CCNJL	15.053	-1.329	0.775	-1.714	0.086511	0.311879	yes
ENSG00000135272	MDFIC	19.135	-0.997	0.322	-3.097	0.001958	0.021453	yes
ENSG00000135525	MAP7	20.347	-1.424	0.414	-3.438	0.000585	0.008042	yes
ENSG00000135596	MICAL1	18.664	-0.743	0.300	-2.478	0.013207	0.088627	yes
ENSG00000135723	FHOD1	31.429	-1.115	0.236	-4.725	2.30E-06	6.78E-05	yes
ENSG00000135749	PCNX2	18.500	-1.441	0.319	-4.512	6.41E-06	0.000168	yes
ENSG00000136108	CKAP2	229.587	-0.833	0.113	-7.373	1.67E-13	1.82E-11	yes
ENSG00000136542	GALNT5	11.747	-1.531	1.681	-0.911	0.362443	0.673773	yes
ENSG00000136928	GABBR2	11.517	-2.255	0.526	-4.287	1.81E-05	0.000417	yes
ENSG00000136982	DSCC1	22.049	-0.925	0.361	-2.562	0.010405	0.074643	yes
ENSG00000137812	KNL1	117.039	-0.765	0.149	-5.142	2.72E-07	9.94E-06	yes
ENSG00000137868	STRA6	25.027	-1.139	0.281	-4.052	5.09E-05	0.001044	yes
ENSG00000137941	TTLL7	22.932	-0.781	0.409	-1.909	0.056278	0.239926	yes
ENSG00000138061	CYP1B1	73.894	-0.731	0.262	-2.792	0.005244	0.045424	yes
ENSG00000138347	MYPN	75.149	-0.852	0.148	-5.764	8.19E-09	4.33E-07	yes
ENSG00000138495	COX17	98.070	-0.785	0.229	-3.426	0.000613	0.008335	yes
ENSG00000138735	PDE5A	22.276	-3.325	0.654	-5.087	3.65E-07	1.30E-05	yes
ENSG00000138764	CCNG2	13.142	-1.077	0.378	-2.851	0.004357	0.039518	yes
ENSG00000139266	MARCHF9	10.080	-0.712	0.431	-1.651	0.098658	0.337792	yes
ENSG00000139291	TMEM19	22.814	-0.756	0.441	-1.715	0.086268	0.311489	yes
ENSG00000139318	DUSP6	145.802	-1.656	0.137	-12.073	1.48E-33	7.88E-31	yes
ENSG00000139970	RTN1	14.042	-0.765	0.437	-1.752	0.079695	0.296539	yes
ENSG00000139998	RAB15	12.410	-0.935	0.360	-2.597	0.009414	0.069464	yes
ENSG00000140511	HAPLN3	13.347	-2.173	1.202	-1.808	0.07061	0.276259	yes
ENSG00000140525	FANCI	60.134	-1.021	0.201	-5.088	3.61E-07	1.29E-05	yes
ENSG00000140545	MFGE8	192.956	-0.717	0.242	-2.967	0.003012	0.029554	yes
ENSG00000141404	GNAL	22.467	-0.908	0.289	-3.140	0.00169	0.01911	yes
ENSG00000142875	PRKACB	47.738	-1.598	0.288	-5.557	2.75E-08	1.26E-06	yes
ENSG00000143228	NUF2	164.063	-0.884	0.121	-7.321	2.47E-13	2.66E-11	yes
ENSG00000143315	PIGM	54.328	-0.745	0.194	-3.841	0.000123	0.002213	yes
ENSG00000143320	CRABP2	78.103	-0.913	0.980	-0.932	0.351374	0.663237	yes
ENSG00000143363	PRUNE1	18.903	-0.833	0.353	-2.361	0.01824	0.111988	yes
ENSG00000143375	CGN	33.657	-1.806	1.005	-1.798	0.072129	0.279075	yes

ENSG00000143393	PI4KB	33.676	-0.848	0.232	-3.656	0.000257	0.004065	yes
ENSG00000143476	DTL	84.056	-0.912	0.160	-5.699	1.20E-08	6.11E-07	yes
ENSG00000143537	ADAM15	109.083	-1.074	0.259	-4.141	3.45E-05	0.000746	yes
ENSG00000143622	RIT1	27.433	-0.712	0.278	-2.560	0.010468	0.074896	yes
ENSG00000143674	MAP3K21	17.559	-1.614	0.418	-3.865	0.000111	0.002044	yes
ENSG00000143799	PARP1	330.206	-0.779	0.103	-7.590	3.21E-14	3.81E-12	yes
ENSG00000144369	FAM171B	12.276	-1.268	0.594	-2.136	0.032689	0.169682	yes
ENSG00000144455	SUMF1	11.987	-0.771	0.498	-1.548	0.121637	0.382716	yes
ENSG00000144815	NXPE3	17.830	-1.373	0.450	-3.055	0.002252	0.023789	yes
ENSG00000144840	RABL3	13.098	-1.091	0.433	-2.518	0.011812	0.081693	yes
ENSG00000145284	SCD5	20.504	-0.782	0.303	-2.578	0.009941	0.072164	yes
ENSG00000145358	DDIT4L	12.735	-2.484	0.442	-5.617	1.94E-08	9.34E-07	yes
ENSG00000145386	CCNA2	75.270	-0.717	0.176	-4.078	4.55E-05	0.000945	yes
ENSG00000145506	NKD2	18.597	-0.889	0.292	-3.041	0.002361	0.024644	yes
ENSG00000145675	PIK3R1	23.056	-1.865	0.345	-5.408	6.36E-08	2.68E-06	yes
ENSG00000145703	IQGAP2	27.709	-1.098	0.266	-4.122	3.76E-05	0.000801	yes
ENSG00000145782	ATG12	156.832	-0.748	0.155	-4.833	1.34E-06	4.19E-05	yes
ENSG00000146373	RNF217	19.839	-0.776	0.342	-2.269	0.023248	0.133145	yes
ENSG00000146918	NCAPG2	122.114	-0.742	0.152	-4.880	1.06E-06	3.42E-05	yes
ENSG00000147050	KDM6A	12.229	-2.456	1.399	-1.756	0.07911	0.295394	yes
ENSG00000147394	ZNF185	16.411	-2.419	1.572	-1.539	0.123898	0.386767	yes
ENSG00000147676	MAL2	274.898	-2.038	1.447	-1.408	0.159014	0.442931	yes
ENSG00000147689	FAM83A	54.002	-1.531	1.672	-0.916	0.359791	0.671342	yes
ENSG00000147813	NAPRT	23.527	-1.691	0.690	-2.452	0.014208	0.093436	yes
ENSG00000147889	CDKN2A	45.796	-2.451	1.391	-1.762	0.078042	0.293407	yes
ENSG00000148200	NR6A1	13.184	-1.498	0.390	-3.838	0.000124	0.002233	yes
ENSG00000148225	WDR31	12.758	-0.772	0.356	-2.171	0.029895	0.158536	yes
ENSG00000148400	NOTCH1	28.694	-0.745	0.262	-2.846	0.004427	0.040019	yes
ENSG00000148468	FAM171A1	45.487	-0.790	0.199	-3.967	7.26E-05	0.001421	yes
ENSG00000148835	TAF5	27.098	-0.735	0.267	-2.756	0.005846	0.049063	yes
ENSG00000149591	TAGLN	19.839	-0.856	0.257	-3.328	0.000876	0.011059	yes
ENSG00000149679	CABLES2	15.229	-0.723	0.349	-2.072	0.038244	0.186789	yes
ENSG00000150782	IL18	11.466	-0.701	0.713	-0.984	0.325036	0.638599	yes
ENSG00000151726	ACSL1	18.752	-0.796	0.318	-2.506	0.012221	0.083899	yes
ENSG00000151849	CENPJ	80.286	-1.079	0.153	-7.073	1.52E-12	1.48E-10	yes
ENSG00000151883	PARP8	12.405	-0.963	0.770	-1.250	0.211119	0.516061	yes
ENSG00000152422	XRCC4	68.626	-0.712	0.160	-4.436	9.15E-06	0.000229	yes
ENSG00000152465	NMT2	127.566	-1.178	0.133	-8.835	1.00E-18	1.88E-16	yes
ENSG00000152527	PLEKHH2	24.305	-0.878	0.385	-2.278	0.022723	0.131144	yes
ENSG00000153044	CENPH	39.999	-0.915	0.238	-3.850	0.000118	0.002149	yes
ENSG00000153132	CLGN	16.538	-0.750	0.353	-2.123	0.033755	0.17274	yes
ENSG00000153208	MERTK	10.607	-1.299	0.551	-2.355	0.018498	0.113173	yes
ENSG00000153993	SEMA3D	25.490	-1.188	0.226	-5.246	1.56E-07	5.99E-06	yes

ENSG00000154310	TNIK	18.270	-0.723	0.632	-1.144	0.252829	0.565033	yes
ENSG00000154920	EME1	16.818	-1.439	0.364	-3.949	7.86E-05	0.001526	yes
ENSG00000155324	GRAMD2B	33.279	-0.870	0.267	-3.256	0.00113	0.013598	yes
ENSG00000155858	LSM11	13.677	-0.798	0.359	-2.221	0.026356	0.145238	yes
ENSG00000156052	GNAQ	37.853	-1.177	0.341	-3.451	0.000559	0.007719	yes
ENSG00000156136	DCK	12.334	-1.256	0.520	-2.417	0.01567	0.100427	yes
ENSG00000156510	HKDC1	104.578	-1.868	0.192	-9.748	1.88E-22	4.50E-20	yes
ENSG00000156970	BUB1B	93.113	-0.771	0.165	-4.684	2.81E-06	8.04E-05	yes
ENSG00000157214	STEAP2	12.756	-1.867	0.687	-2.718	0.006573	0.05363	yes
ENSG00000157216	SSBP3	29.819	-0.762	0.266	-2.863	0.004201	0.038377	yes
ENSG00000158301	GPRASP2	14.398	-1.249	0.356	-3.508	0.000452	0.006512	yes
ENSG00000158373	H2BC5	37.538	-0.994	0.293	-3.391	0.000697	0.009247	yes
ENSG00000158402	CDC25C	44.425	-1.236	0.213	-5.795	6.84E-09	3.75E-07	yes
ENSG00000158406	H4C8	10.864	-0.741	0.536	-1.382	0.167019	0.454566	yes
ENSG00000158710	TAGLN2	436.757	-0.981	0.184	-5.329	9.85E-08	3.97E-06	yes
ENSG00000158769	F11R	43.982	-0.837	0.328	-2.548	0.010828	0.076757	yes
ENSG00000159167	STC1	41.811	-1.821	0.234	-7.770	7.83E-15	9.55E-13	yes
ENSG00000159217	IGF2BP1	22.330	-1.589	1.262	-1.259	0.207925	0.511637	yes
ENSG00000160179	ABCG1	14.395	-1.231	0.335	-3.672	0.00024	0.00385	yes
ENSG00000160298	C21orf58	26.668	-0.751	0.250	-3.004	0.002666	0.027081	yes
ENSG00000160392	C19orf47	19.219	-0.737	0.349	-2.115	0.034449	0.174432	yes
ENSG00000160446	ZDHHC12	19.040	-0.826	0.335	-2.463	0.013793	0.091606	yes
ENSG00000160570	DEDD2	22.097	-0.961	0.319	-3.013	0.00259	0.026423	yes
ENSG00000160803	UBQLN4	12.352	-1.081	0.392	-2.757	0.005833	0.049045	yes
ENSG00000160957	RECQL4	36.389	-0.831	0.264	-3.153	0.001618	0.018462	yes
ENSG00000161010	MRNIP	29.062	-0.758	0.239	-3.168	0.001537	0.017622	yes
ENSG00000161677	JOSD2	13.895	-0.746	0.366	-2.039	0.041404	0.196747	yes
ENSG00000161682	FAM171A2	10.651	-1.142	0.437	-2.613	0.008967	0.066878	yes
ENSG00000161791	FMNL3	10.553	-1.097	0.455	-2.414	0.015795	0.100989	yes
ENSG00000161847	RAVER1	20.139	-0.981	0.316	-3.101	0.001929	0.021234	yes
ENSG00000162062	TEDC2	10.519	-0.700	0.515	-1.360	0.173918	0.464951	yes
ENSG00000162063	CCNF	37.878	-0.986	0.221	-4.469	7.86E-06	0.0002	yes
ENSG00000162174	ASRGL1	32.097	-0.781	0.282	-2.765	0.005697	0.048321	yes
ENSG00000162396	PARS2	10.259	-0.707	0.456	-1.552	0.120599	0.381252	yes
ENSG00000162496	DHRS3	36.596	-1.165	0.300	-3.879	0.000105	0.001947	yes
ENSG00000162576	MXRA8	25.029	-1.177	1.332	-0.884	0.376894	0.683334	yes
ENSG00000162852	CNST	21.297	-0.891	0.300	-2.970	0.002976	0.029326	yes
ENSG00000163006	CCDC138	20.148	-0.728	0.321	-2.266	0.023481	0.134021	yes
ENSG00000163013	FBXO41	16.403	-0.754	0.288	-2.616	0.00891	0.066602	yes
ENSG00000163171	CDC42EP3	248.106	-0.962	0.135	-7.113	1.13E-12	1.14E-10	yes
ENSG00000163346	PBXIP1	22.147	-0.823	0.303	-2.721	0.006517	0.053356	yes
ENSG00000163435	ELF3	47.249	-0.940	0.275	-3.423	0.000619	0.008397	yes
ENSG00000163463	KRTCAP2	13.832	-0.927	0.415	-2.234	0.025472	0.142015	yes

ENSG00000163491	NEK10	10.083	-1.467	0.384	-3.823	0.000132	0.002355	yes
ENSG00000163508	EOMES	12.104	-1.805	0.336	-5.378	7.54E-08	3.12E-06	yes
ENSG00000163545	NUAK2	24.230	-0.996	0.283	-3.522	0.000429	0.006283	yes
ENSG00000163624	CDS1	17.693	-1.499	0.601	-2.496	0.01256	0.085458	yes
ENSG00000163918	RFC4	43.933	-0.776	0.203	-3.814	0.000137	0.002431	yes
ENSG00000164171	ITGA2	115.252	-1.285	0.413	-3.115	0.001839	0.020505	yes
ENSG00000164611	PTTG1	309.045	-0.999	0.094	-10.682	1.23E-26	4.07E-24	yes
ENSG00000164687	FABP5	15.280	-1.338	0.403	-3.324	0.000886	0.011168	yes
ENSG00000164695	CHMP4C	67.574	-1.453	1.563	-0.929	0.35287	0.664523	yes
ENSG00000164855	TMEM184A	24.251	-0.945	0.315	-3.001	0.00269	0.027245	yes
ENSG00000165238	WNK2	23.439	-0.905	0.277	-3.263	0.001101	0.013317	yes
ENSG00000165480	SKA3	38.990	-0.935	0.246	-3.799	0.000146	0.002553	yes
ENSG00000165801	ARHGEF40	11.192	-1.273	0.410	-3.103	0.001919	0.021175	yes
ENSG00000165810	BTNL9	19.743	-1.106	0.273	-4.045	5.22E-05	0.001069	yes
ENSG00000165821	SALL2	17.867	-0.973	0.295	-3.299	0.000972	0.012109	yes
ENSG00000165891	E2F7	27.636	-0.746	0.332	-2.245	0.024773	0.139057	yes
ENSG00000165895	ARHGAP42	15.050	-1.323	1.080	-1.225	0.220499	0.528118	yes
ENSG00000166825	ANPEP	23.494	-2.996	1.455	-2.059	0.039454	0.190142	yes
ENSG00000166831	RBPMS2	16.038	-0.831	0.355	-2.339	0.019354	0.117274	yes
ENSG00000166974	MAPRE2	13.284	-0.971	0.410	-2.367	0.017921	0.110613	yes
ENSG00000167641	PPP1R14A	95.843	-2.155	0.250	-8.619	6.74E-18	1.09E-15	yes
ENSG00000167645	YIF1B	17.355	-0.923	0.339	-2.726	0.006414	0.052668	yes
ENSG00000167670	CHAF1A	86.951	-0.819	0.169	-4.836	1.32E-06	4.17E-05	yes
ENSG00000167680	SEMA6B	11.757	-0.919	0.422	-2.176	0.029558	0.157068	yes
ENSG00000167703	SLC43A2	19.583	-1.875	0.313	-5.992	2.07E-09	1.26E-07	yes
ENSG00000168505	GBX2	11.990	-1.536	0.335	-4.584	4.57E-06	0.000125	yes
ENSG00000168672	LRATD2	16.175	-0.958	0.760	-1.260	0.207553	0.511423	yes
ENSG00000168779	SHOX2	116.185	-0.804	0.170	-4.736	2.17E-06	6.48E-05	yes
ENSG00000169230	PRELID1	255.705	-0.806	0.118	-6.856	7.07E-12	6.18E-10	yes
ENSG00000169241	SLC50A1	59.779	-0.716	0.189	-3.791	0.00015	0.002622	yes
ENSG00000169258	GPRIN1	13.928	-0.943	0.369	-2.553	0.01067	0.076027	yes
ENSG00000169306	IL1RAPL1	16.472	-2.432	0.390	-6.231	4.63E-10	3.20E-08	yes
ENSG00000169679	BUB1	150.409	-0.981	0.127	-7.755	8.86E-15	1.07E-12	yes
ENSG00000169855	ROBO1	29.656	-1.040	0.260	-3.999	6.36E-05	0.001259	yes
ENSG00000170412	GPRC5C	13.774	-1.263	0.457	-2.764	0.005708	0.048386	yes
ENSG00000170468	RIOX1	28.973	-0.706	0.253	-2.788	0.005301	0.045663	yes
ENSG00000170522	ELOVL6	43.007	-0.960	0.248	-3.877	0.000106	0.001961	yes
ENSG00000170571	EMB	23.465	-3.637	0.904	-4.024	5.73E-05	0.001161	yes
ENSG00000170734	POLH	17.629	-0.876	0.329	-2.664	0.007727	0.059941	yes
ENSG00000171163	ZNF692	22.690	-0.703	0.337	-2.087	0.036871	0.182374	yes
ENSG00000171241	SHCBP1	69.567	-0.743	0.181	-4.114	3.89E-05	0.000826	yes
ENSG00000171604	CXXC5	96.837	-0.859	0.190	-4.517	6.27E-06	0.000165	yes
ENSG00000171729	TMEM51	25.660	-0.734	0.299	-2.456	0.014035	0.092638	yes

ENSG00000171861	MRM3	26.804	-0.802	0.258	-3.105	0.001905	0.021052	yes
ENSG00000171864	PRND	18.586	-1.204	0.343	-3.508	0.000451	0.006507	yes
ENSG00000172062	SMN1	13.632	-0.745	0.423	-1.760	0.078353	0.294228	yes
ENSG00000172081	MOB3A	18.268	-0.724	0.305	-2.378	0.01742	0.108146	yes
ENSG00000172123	SLFN12	22.239	-2.010	0.992	-2.027	0.042684	0.200498	yes
ENSG00000172244	C5orf34	10.657	-0.955	0.408	-2.340	0.019287	0.116955	yes
ENSG00000172331	BPGM	11.761	-0.803	0.506	-1.589	0.112158	0.364867	yes
ENSG00000172965	MIR4435-2H(	117.841	-0.710	0.165	-4.315	1.59E-05	0.000374	yes
ENSG00000173207	CKS1B	202.286	-0.866	0.210	-4.131	3.61E-05	0.000776	yes
ENSG00000173267	SNCG	14.880	-2.072	0.411	-5.047	4.48E-07	1.57E-05	yes
ENSG00000174371	EXO1	32.535	-0.945	0.256	-3.694	0.00022	0.003595	yes
ENSG00000175137	SH3BP5L	34.705	-0.895	0.307	-2.910	0.003617	0.034184	yes
ENSG00000175315	CST6	215.160	-1.455	1.561	-0.932	0.351417	0.663237	yes
ENSG00000175455	CCDC14	148.041	-0.751	0.121	-6.212	5.24E-10	3.59E-08	yes
ENSG00000176170	SPHK1	13.173	-1.848	0.800	-2.308	0.020984	0.123605	yes
ENSG00000176749	CDK5R1	21.261	-0.881	0.326	-2.703	0.006866	0.055146	yes
ENSG00000177303	CASKIN2	10.965	-0.926	0.451	-2.054	0.039963	0.191958	yes
ENSG00000177570	SAMD12	11.656	-1.531	1.686	-0.908	0.363755	0.674588	yes
ENSG00000177706	FAM20C	18.957	-1.076	0.333	-3.232	0.00123	0.014628	yes
ENSG00000177707	NECTIN3	51.121	-1.220	0.205	-5.956	2.59E-09	1.56E-07	yes
ENSG00000178307	TMEM11	47.082	-0.815	0.218	-3.733	0.00019	0.003174	yes
ENSG00000178531	CTXN1	45.577	-0.921	0.232	-3.963	7.40E-05	0.001444	yes
ENSG00000178999	AURKB	143.141	-0.772	0.136	-5.686	1.30E-08	6.52E-07	yes
ENSG00000179348	GATA2	41.393	-0.857	0.202	-4.235	2.28E-05	0.000514	yes
ENSG00000179981	TSHZ1	25.705	-0.888	0.260	-3.415	0.000639	0.008609	yes
ENSG00000180448	ARHGAP45	13.507	-1.035	0.431	-2.399	0.016433	0.103547	yes
ENSG00000180530	NRIP1	64.931	-1.473	0.236	-6.231	4.65E-10	3.20E-08	yes
ENSG00000180596	H2BC4	28.847	-0.736	0.312	-2.361	0.018203	0.111861	yes
ENSG00000180806	HOXC9	36.063	-1.531	1.671	-0.916	0.359559	0.671342	yes
ENSG00000181104	F2R	181.861	-1.180	0.122	-9.648	5.03E-22	1.18E-19	yes
ENSG00000181444	ZNF467	15.461	-0.801	0.375	-2.137	0.032635	0.169505	yes
ENSG00000181744	DIPK2A	11.601	-1.346	0.438	-3.075	0.002104	0.022698	yes
ENSG00000181885	CLDN7	53.187	-2.039	1.452	-1.405	0.16006	0.444336	yes
ENSG00000182405	PGBD4	17.528	-0.841	0.339	-2.478	0.01321	0.088627	yes
ENSG00000182518	FAM104B	19.792	-0.785	0.317	-2.472	0.013432	0.089765	yes
ENSG00000182809	CRIP2	81.013	-1.623	0.198	-8.203	2.34E-16	3.28E-14	yes
ENSG00000183018	SPNS2	14.700	-2.420	1.575	-1.537	0.124412	0.387535	yes
ENSG00000183048	SLC25A10	20.625	-0.772	0.324	-2.381	0.017248	0.107414	yes
ENSG00000183668	PSG9	29.969	-3.093	0.269	-11.515	1.11E-30	4.99E-28	yes
ENSG00000183779	ZNF703	11.472	-1.013	0.391	-2.591	0.009578	0.070377	yes
ENSG00000183814	LIN9	19.097	-0.926	0.371	-2.499	0.012439	0.084842	yes
ENSG00000183856	IQGAP3	50.009	-1.011	0.206	-4.919	8.70E-07	2.86E-05	yes
ENSG00000184205	TSPYL2	68.858	-0.936	0.206	-4.535	5.76E-06	0.000154	yes

ENSG00000184226	PCDH9	11.464	-1.157	0.531	-2.177	0.02947	0.156859	yes
ENSG00000184260	H2AC20	91.065	-0.888	0.205	-4.323	1.54E-05	0.000363	yes
ENSG00000184292	TACSTD2	73.482	-1.531	1.670	-0.917	0.359256	0.671172	yes
ENSG00000184349	EFNA5	53.554	-1.117	0.222	-5.039	4.68E-07	1.63E-05	yes
ENSG00000184357	H1-5	35.168	-0.747	0.244	-3.066	0.002167	0.023148	yes
ENSG00000184661	CDCA2	114.754	-0.714	0.133	-5.369	7.94E-08	3.25E-06	yes
ENSG00000184986	TMEM121	15.077	-1.693	0.347	-4.887	1.02E-06	3.32E-05	yes
ENSG00000185347	TEDC1	20.285	-0.988	0.277	-3.570	0.000357	0.005418	yes
ENSG00000185483	ROR1	11.721	-1.558	0.558	-2.792	0.005238	0.045424	yes
ENSG00000186472	PCLO	25.715	-1.957	0.357	-5.479	4.27E-08	1.88E-06	yes
ENSG00000186481	ANKRD20A5P	10.246	-0.956	0.661	-1.447	0.147815	0.424679	yes
ENSG00000186871	ERCC6L	32.784	-0.863	0.265	-3.262	0.001106	0.013349	yes
ENSG00000187098	MITF	30.326	-3.554	0.403	-8.821	1.13E-18	2.10E-16	yes
ENSG00000187231	SESTD1	15.185	-1.327	0.433	-3.063	0.002191	0.023362	yes
ENSG00000187244	BCAM	17.898	-0.978	0.485	-2.019	0.043498	0.203625	yes
ENSG00000187634	SAMD11	18.020	-0.964	0.642	-1.502	0.133207	0.402926	yes
ENSG00000187778	MCRS1	49.050	-0.827	0.275	-3.009	0.002618	0.026666	yes
ENSG00000188010	MORN2	12.764	-1.637	0.425	-3.854	0.000116	0.002116	yes
ENSG00000188641	DPYD	99.498	-0.772	0.180	-4.292	1.77E-05	0.000409	yes
ENSG00000189171	S100A13	150.115	-1.083	0.178	-6.098	1.08E-09	6.92E-08	yes
ENSG00000189337	KAZN	54.575	-1.225	0.210	-5.832	5.48E-09	3.09E-07	yes
ENSG00000196368	NUDT11	21.822	-1.836	0.310	-5.924	3.14E-09	1.85E-07	yes
ENSG00000196611	MMP1	15.449	-1.135	0.401	-2.831	0.004643	0.041382	yes
ENSG00000196739	COL27A1	47.447	-0.929	0.168	-5.535	3.12E-08	1.41E-06	yes
ENSG00000196878	LAMB3	23.311	-1.591	1.037	-1.534	0.125041	0.388924	yes
ENSG00000196954	CASP4	98.654	-0.849	0.142	-5.981	2.21E-09	1.34E-07	yes
ENSG00000197043	ANXA6	76.321	-0.947	0.179	-5.297	1.18E-07	4.71E-06	yes
ENSG00000197061	H4C3	1312.132	-0.743	0.140	-5.293	1.20E-07	4.76E-06	yes
ENSG00000197142	ACSL5	13.205	-1.449	1.576	-0.919	0.357953	0.669999	yes
ENSG00000197183	NOL4L	13.520	-0.818	0.362	-2.262	0.023709	0.134784	yes
ENSG00000197555	SIPA1L1	36.571	-1.136	0.281	-4.047	5.20E-05	0.001065	yes
ENSG00000197782	ZNF780A	38.623	-0.882	0.348	-2.533	0.011294	0.079128	yes
ENSG00000197948	FCHSD1	16.177	-0.890	0.362	-2.459	0.013937	0.092286	yes
ENSG00000198055	GRK6	69.473	-0.815	0.169	-4.834	1.34E-06	4.19E-05	yes
ENSG00000198626	RYR2	46.657	-1.185	0.263	-4.510	6.49E-06	0.000169	yes
ENSG00000198863	RUNDC1	38.679	-0.782	0.256	-3.055	0.002253	0.023789	yes
ENSG00000198892	SHISA4	19.047	-0.757	0.333	-2.275	0.022879	0.13174	yes
ENSG00000199377	RNU5F-1	34.949	-0.936	0.361	-2.591	0.009564	0.070311	yes
ENSG00000200090	n/a	14.629	-0.787	0.376	-2.095	0.036172	0.180151	yes
ENSG00000201098	RNY1	15.682	-1.767	0.458	-3.862	0.000113	0.002065	yes
ENSG00000201592	n/a	10.502	-1.026	0.418	-2.457	0.014015	0.092595	yes
ENSG00000203668	CHML	49.819	-0.942	0.219	-4.304	1.68E-05	0.000392	yes
ENSG00000204186	ZDBF2	10.875	-2.892	1.146	-2.523	0.01163	0.080711	yes

ENSG00000204308	RNF5	39.642	-0.945	0.230	-4.109	3.98E-05	0.000844	yes
ENSG00000204397	CARD16	23.929	-2.335	0.277	-8.430	3.47E-17	5.29E-15	yes
ENSG00000204536	CCHCR1	14.102	-0.922	0.348	-2.647	0.008111	0.062227	yes
ENSG00000204650	LINC02210	18.728	-0.886	0.323	-2.743	0.006081	0.050656	yes
ENSG00000204859	ZBTB48	10.898	-0.940	0.432	-2.174	0.029669	0.157516	yes
ENSG00000205544	TMEM256	40.820	-0.742	0.247	-2.999	0.002705	0.027351	yes
ENSG00000205922	ONECUT3	24.923	-2.413	1.568	-1.539	0.123751	0.386767	yes
ENSG00000211459	MT-RNR1	48996.297	-0.716	0.260	-2.752	0.005914	0.04945	yes
ENSG00000213347	MXD3	11.968	-1.362	0.403	-3.382	0.000719	0.009455	yes
ENSG00000213397	HAUS7	22.769	-0.883	0.287	-3.081	0.002066	0.022443	yes
ENSG00000214357	NEURL1B	54.359	-0.884	0.251	-3.522	0.000428	0.006283	yes
ENSG00000214575	CPEB1	10.082	-1.017	0.823	-1.235	0.216752	0.522824	yes
ENSG00000215105	TTC3P1	20.260	-1.372	0.300	-4.565	5.00E-06	0.000135	yes
ENSG00000220804	LINC01881	12.899	-0.813	0.396	-2.054	0.039985	0.191966	yes
ENSG00000221926	TRIM16	14.178	-0.862	0.425	-2.029	0.042474	0.199908	yes
ENSG00000222041	CYTOR	94.123	-1.063	0.177	-6.013	1.82E-09	1.12E-07	yes
ENSG00000223784	LINP1	14.137	-2.511	0.764	-3.285	0.001018	0.012575	yes
ENSG00000223949	ROR1-AS1	32.242	-1.891	0.226	-8.379	5.35E-17	7.99E-15	yes
ENSG00000224287	MSL3P1	10.134	-2.408	1.584	-1.520	0.128418	0.394657	yes
ENSG00000226137	BAIAP2-DT	18.637	-0.886	0.357	-2.480	0.013146	0.088362	yes
ENSG00000228716	DHFR	41.073	-0.860	0.234	-3.682	0.000231	0.003732	yes
ENSG00000228742	LINC02577	32.876	-0.781	0.228	-3.421	0.000624	0.008444	yes
ENSG00000231607	DLEU2	13.922	-1.311	0.446	-2.938	0.003302	0.031815	yes
ENSG00000233461	n/a	11.602	-1.348	0.554	-2.433	0.014975	0.09725	yes
ENSG00000235609	n/a	40.588	-2.084	0.200	-10.399	2.50E-25	8.09E-23	yes
ENSG00000237649	KIFC1	86.491	-0.805	0.193	-4.174	2.99E-05	0.000657	yes
ENSG00000238105	GOLGA2P5	17.072	-0.784	0.311	-2.524	0.011619	0.080676	yes
ENSG00000239521	CASTOR3	17.491	-0.795	0.322	-2.471	0.013492	0.090087	yes
ENSG00000240616	RPS6P25	10.249	-0.894	0.414	-2.158	0.030914	0.162571	yes
ENSG00000241404	EGFL8	14.521	-0.883	0.387	-2.282	0.022503	0.130764	yes
ENSG00000242114	MTFP1	18.047	-0.758	0.360	-2.106	0.035206	0.17683	yes
ENSG00000242195	SRRM1P2	67.808	-1.297	0.199	-6.528	6.69E-11	5.13E-09	yes
ENSG00000250222	n/a	12.153	-0.999	0.359	-2.784	0.005369	0.046048	yes
ENSG00000253276	CCDC71L	212.122	-1.031	0.250	-4.119	3.81E-05	0.000811	yes
ENSG00000254901	BORCS8	29.635	-1.132	0.277	-4.091	4.30E-05	0.000903	yes
ENSG00000255823	MTRNR2L8	11.095	-0.702	0.422	-1.662	0.096425	0.332853	yes
ENSG00000257698	GIHCG	18.271	-1.174	0.418	-2.808	0.004983	0.04387	yes
ENSG00000258301	VASH1-AS1	10.431	-0.707	0.459	-1.538	0.123943	0.386767	yes
ENSG00000264229	RNU4ATAC	2672.982	-1.122	0.243	-4.614	3.95E-06	0.00011	yes
ENSG00000266094	RASSF5	10.447	-0.911	0.492	-1.851	0.064224	0.26193	yes
ENSG00000269343	ZNF587B	15.681	-0.984	0.386	-2.549	0.010812	0.076682	yes
ENSG00000269713	NBPF9	19.080	-0.915	0.294	-3.112	0.001856	0.020665	yes
ENSG00000270885	RASL10B	16.681	-0.778	0.314	-2.475	0.013316	0.08916	yes

ENSG00000272398	CD24	17.220	-1.169	0.955	-1.224	0.220949	0.528729	yes
ENSG00000272620	AFAP1-AS1	44.173	-3.000	1.448	-2.071	0.038323	0.186883	yes
ENSG00000273706	LHX1	127.982	-1.191	0.138	-8.611	7.23E-18	1.15E-15	yes
ENSG00000275180	n/a	11.358	-1.101	0.393	-2.803	0.005055	0.044288	yes
ENSG00000276043	UHRF1	18.435	-0.967	0.331	-2.918	0.003522	0.033498	yes
ENSG00000276368	H2AC14	55.015	-1.018	0.253	-4.023	5.75E-05	0.001163	yes
ENSG00000277268	LHX1-DT	38.591	-0.868	0.245	-3.549	0.000386	0.005823	yes
