# Supplementary material for: Transcriptomic and functional analysis of ANGPTL4 overexpression in pancreatic cancer nominates targets that reverse chemoresistance
Source: BMC Cancer. 2023 Jun 8;23:524. doi: 10.1186/s12885-023-11010-1 (PMC10251551; doi:10.1186/s12885-023-11010-1)
Supplement: Supplementary file 5 — Additional file 5: Table S3.txt [file 12885_2023_11010_MOESM5_ESM.pdf]

Supplementary Table 3: 114 Gemcitabine Resistance Genes

#	HGNC Symbol	Ensembl Gene
1	BMI1	ENSG00000168283
2	POLD3	ENSG00000077514
3	PARP1	ENSG00000143799
4	ANO1	ENSG00000131620
5	SCEL	ENSG00000136155
6	NET1	ENSG00000173848
7	BNIP3	ENSG00000176171
8	CCDC148	ENSG00000153237
9	SH3RF2	ENSG00000156463
10	CACNA1D	ENSG00000157388
11	AP1M2	ENSG00000129354
12	C4orf19	ENSG00000154274
13	VGLL1	ENSG00000102243
14	INPP4B	ENSG00000109452
15	INSIG2	ENSG00000125629
16	SLC29A1	ENSG00000112759
17	dCK	ENSG00000156136
18	RRM1	ENSG00000167325
19	RRM2	ENSG00000171848
20	TOR1A	ENSG00000136827
21	VIPR1	ENSG00000114812
22	ATP11C	ENSG00000101974
23	MTSS1	ENSG00000170873
24	SAFB	ENSG00000160633
25	RALBP1	ENSG00000017797
26	SMAD2	ENSG00000175387
27	ptk2	ENSG00000169398
28	TRAF6	ENSG00000175104
29	MADD	ENSG00000110514
30	PPP3CB	ENSG00000107758
31	TSC1	ENSG00000165699
32	STAT5A	ENSG00000126561
33	SOCS5	ENSG00000171150
34	JADE3	ENSG00000102221
35	PIK3C3	ENSG00000078142
36	UNC13B	ENSG00000198722
37	MAP3K7	ENSG00000135341
38	LTBP1	ENSG00000049323
39	ITGA9	ENSG00000144668
40	GNAQ	ENSG00000156052
41	KAT6A	ENSG00000083168

42	SKIP	ENSG00000100603
43	C5	ENSG00000106804
44	PPP1R15A	ENSG00000087074
45	LRP3	ENSG00000130881
46	SLC9A3	ENSG00000066230
47	SAC3D1	ENSG00000168061
48	SLC25A51	ENSG00000122696
49	SEMA5A	ENSG00000112902
50	ELN	ENSG00000049540
51	ZC3H11A	ENSG00000058673
52	OGT	ENSG00000147162
53	WAPL	ENSG00000062650
54	UAP1	ENSG00000117143
55	INPP5E	ENSG00000148384
56	HECA	ENSG00000112406
57	RPS21	ENSG00000171858
58	PCSK7	ENSG00000160613
59	SEC23B	ENSG00000101310
60	OGA	ENSG00000198408
61	LTV1	ENSG00000135521
62	HAGH	ENSG00000063854
63	LAMP2	ENSG00000005893
64	NFKBIB	ENSG00000104825
65	AKT1	ENSG00000142208
66	MAPK1	ENSG00000100030
67	HIF1A	ENSG00000100644
68	SHH	ENSG00000164690
69	PI3KCB	ENSG00000051382
70	MAP3K10	ENSG00000130758
71	ABCG2	ENSG00000118777
72	ALDH1A1	ENSG00000165092
73	CD24	ENSG00000272398
74	FZD	No data available
75	DLV1	ENSG00000107404
76	APC	ENSG00000134982
77	GSK3A	ENSG00000105723
78	AXIN2	ENSG00000168646
79	NLK	ENSG00000087095
80	NFAM1	ENSG00000235568
81	WNT5A	ENSG00000114251
82	ZEB1	ENSG00000148516
83	AXIN1	ENSG00000103126
84	DLL1	ENSG00000198719

85	JAG1	ENSG00000101384
86	CSNK1A1	ENSG00000113712
87	SNAIL	No data available
88	WNT2	ENSG00000105989
89	PDGFRA	ENSG00000134853
90	CD44	ENSG00000026508
91	MET	ENSG00000105976
92	JAG2	ENSG00000184916
93	DLL3	ENSG00000090932
94	DLL4	ENSG00000128917
95	NOTCH1	ENSG00000148400
96	NOTCH2	ENSG00000134250
97	NOTCH3	ENSG00000074181
98	NOTCH4	ENSG00000204301
99	IHH	ENSG00000163501
100	PTCH1	ENSG00000185920
101	PTCH2	ENSG00000117425
102	SMO	ENSG00000128602
103	GLI1	ENSG00000111087
104	GLI2	ENSG00000074047
105	GLI3	ENSG00000106571
106	CTNNB1	ENSG00000168036
107	WNT1	ENSG00000125084
108	FZD5	ENSG00000163251
109	FZD4	ENSG00000174804
110	FZD6	ENSG00000164930
111	FZD8	ENSG00000177283
112	SNAI1	ENSG00000124216
113	SNAI2	ENSG00000019549
114	SNAI3	ENSG00000185669
