# Supplementary material for: Transcriptomic and functional analysis of ANGPTL4 overexpression in pancreatic cancer nominates targets that reverse chemoresistance
Source: BMC Cancer. 2023 Jun 8;23:524. doi: 10.1186/s12885-023-11010-1 (PMC10251551; doi:10.1186/s12885-023-11010-1)
Supplement: Supplementary file 6 — Additional file 6: Table S4.txt [file 12885_2023_11010_MOESM6_ESM.pdf]

Supplementary Table 4: AMIGO2 Panther GO biological process EMT results for with or without gemcitabine analysis

#	Gene ID	Mapped IDs	Gene name	Panther Family/Subfamily	Panther Protein Class	Species	Genes from Gemcitabine Treatment DEG List
1	HUMAN|HGNC=3372|UniProtKB=O95936	EOMES	Eomesodermin homolog	EOMESODERMIN HOMOLOG (PTHR11267:SF13)	Rel homology transcription factor	Homo sapiens	Yes
2	HUMAN|HGNC=11269|UniProtKB=O43609	SPRY1	Protein sprouty homolog 1	PROTEIN SPROUTY HOMOLOG 1 (PTHR12365:SF10)	scaffold/adaptor protein	Homo sapiens	Yes
3	HUMAN|HGNC=11774|UniProtKB=Q03167	TGFBR3	Transforming growth factor beta receptor type 3	TRANSFORMING GROWTH FACTOR BETA RECEPTOR TYPE 3 (PTHR14002:SF7)	transmembrane signal receptor	Homo sapiens	Yes
4	HUMAN|HGNC=11654|UniProtKB=Q13428	TCOF1	Treacle protein	TREACLE PROTEIN (PTHR20787:SF10)	RNA metabolism protein	Homo sapiens	Yes
5	HUMAN|HGNC=3688|UniProtKB=P11362	FGFR1	Fibroblast growth factor receptor 1	FIBROBLAST GROWTH FACTOR RECEPTOR 1 (PTHR24416:SF131)	transmembrane signal receptor	Homo sapiens	Yes
6	HUMAN|HGNC=20861|UniProtKB=Q9ULF5	SLC39A10	Zinc transporter ZIP10	ZINC TRANSPORTER ZIP10 (PTHR12191:SF14)	secondary carrier transporter	Homo sapiens	Yes
7	HUMAN|HGNC=11204|UniProtKB=P48436	SOX9	Transcription factor SOX-9	TRANSCRIPTION FACTOR SOX-9 (PTHR45803:SF1)	-	Homo sapiens	Yes
8	HUMAN|HGNC=10776|UniProtKB=Q8N474	SFRP1	Secreted frizzled-related protein 1	SECRETED FRIZZLED-RELATED PROTEIN 1 (PTHR11309:SF87)	transmembrane signal receptor	Homo sapiens	Yes
9	HUMAN|HGNC=7866|UniProtKB=Q13253	NOG	Noggin	NOGGIN (PTHR10494:SF5)	intercellular signal molecule	Homo sapiens	Yes
10	HUMAN|HGNC=7881|UniProtKB=P46531	NOTCH1	Neurogenic locus notch homolog protein 1	NEUROGENIC LOCUS NOTCH HOMOLOG PROTEIN 1 (PTHR45836:SF12)	-	Homo sapiens	Yes
11	HUMAN|HGNC=3176|UniProtKB=P05305	EDN1	Endothelin-1	ENDOTHELIN-1 (PTHR13874:SF10)	peptide hormone	Homo sapiens	Yes
12	HUMAN|HGNC=193|UniProtKB=Q13444	ADAM15	Disintegrin and metalloproteinase domain-containing protein 15	DISINTEGRIN AND METALLOPROTEINASE DOMAIN-CONTAINING PROTEIN 15 (PTHR11905:SF130)	metalloprotease	Homo sapiens	Yes
1	HUMAN|HGNC=3372|UniProtKB=O95936	EOMES	Eomesodermin homolog	EOMESODERMIN HOMOLOG (PTHR11267:SF13)	Rel homology transcription factor	Homo sapiens	No
2	HUMAN|HGNC=5034|UniProtKB=Q99729	HNRNPAB	Heterogeneous nuclear ribonucleoprotein A/B	HETEROGENEOUS NUCLEAR RIBONUCLEOPROTEIN A/B (PTHR48033:SF1)	RNA metabolism protein	Homo sapiens	No
3	HUMAN|HGNC=11269|UniProtKB=O43609	SPRY1	Protein sprouty homolog 1	PROTEIN SPROUTY HOMOLOG 1 (PTHR12365:SF10)	scaffold/adaptor protein	Homo sapiens	No
4	HUMAN|HGNC=11774|UniProtKB=Q03167	TGFBR3	Transforming growth factor beta receptor type 3	TRANSFORMING GROWTH FACTOR BETA RECEPTOR TYPE 3 (PTHR14002:SF7)	transmembrane signal receptor	Homo sapiens	No
5	HUMAN|HGNC=4186|UniProtKB=P52951	GBX2	Homeobox protein GBX-2	HOMEOBOX PROTEIN GBX-2 (PTHR24334:SF3)	homeodomain transcription factor	Homo sapiens	No
6	HUMAN|HGNC=16122|UniProtKB=Q9H4H8	FAM83D	Protein FAM83D	PROTEIN FAM83B-RELATED (PTHR16181:SF28)	-	Homo sapiens	No
7	HUMAN|HGNC=10494|UniProtKB=P26447	S100A4	Protein S100-A4	PROTEIN S100-A4 (PTHR11639:SF51)	calmodulin-related	Homo sapiens	No
8	HUMAN|HGNC=11654|UniProtKB=Q13428	TCOF1	Treacle protein	TREACLE PROTEIN (PTHR20787:SF10)	RNA metabolism protein	Homo sapiens	No
9	HUMAN|HGNC=24042|UniProtKB=Q9GZV5	WWTR1	WW domain-containing transcription regulator protein 1	WW DOMAIN-CONTAINING TRANSCRIPTION REGULATOR PROTEIN 1 (PTHR17616:SF6)	transcription cofactor	Homo sapiens	No
10	HUMAN|HGNC=11203|UniProtKB=P57073	SOX8	Transcription factor SOX-8	TRANSCRIPTION FACTOR SOX-8 (PTHR45803:SF2)	-	Homo sapiens	No
11	HUMAN|HGNC=3688|UniProtKB=P11362	FGFR1	Fibroblast growth factor receptor 1	FIBROBLAST GROWTH FACTOR RECEPTOR 1 (PTHR24416:SF131)	transmembrane signal receptor	Homo sapiens	No
12	HUMAN|HGNC=20861|UniProtKB=Q9ULF5	SLC39A10	Zinc transporter ZIP10	ZINC TRANSPORTER ZIP10 (PTHR12191:SF14)	secondary carrier transporter	Homo sapiens	No
13	HUMAN|HGNC=11204|UniProtKB=P48436	SOX9	Transcription factor SOX-9	TRANSCRIPTION FACTOR SOX-9 (PTHR45803:SF1)	-	Homo sapiens	No
14	HUMAN|HGNC=10776|UniProtKB=Q8N474	SFRP1	Secreted frizzled-related protein 1	SECRETED FRIZZLED-RELATED PROTEIN 1 (PTHR11309:SF87)	transmembrane signal receptor	Homo sapiens	No
15	HUMAN|HGNC=1987|UniProtKB=Q99967	CITED2	Cbp/p300-interacting transactivator 2	CBP/P300-INTERACTING TRANSACTIVATOR 2 (PTHR17045:SF7)	transcription cofactor	Homo sapiens	No
16	HUMAN|HGNC=21024|UniProtKB=Q14999	CUL7	Cullin-7	CULLIN-7 (PTHR22771:SF3)	-	Homo sapiens	No
17	HUMAN|HGNC=10723|UniProtKB=Q14563	SEMA3A	Semaphorin-3A	SEMAPHORIN-3A (PTHR11036:SF23)	membrane-bound signaling molecule	Homo sapiens	No
18	HUMAN|HGNC=11362|UniProtKB=P42224	STAT1	Signal transducer and activator of transcription 1-alpha/beta	SIGNAL TRANSDUCER AND ACTIVATOR OF TRANSCRIPTION 1-ALPHA/BETA (PTHR11801:SF18)	DNA-binding transcription factor	Homo sapiens	No
19	HUMAN|HGNC=10725|UniProtKB=Q99985	SEMA3C	Semaphorin-3C	SEMAPHORIN-3C (PTHR11036:SF25)	membrane-bound signaling molecule	Homo sapiens	No
20	HUMAN|HGNC=7881|UniProtKB=P46531	NOTCH1	Neurogenic locus notch homolog protein 1	NEUROGENIC LOCUS NOTCH HOMOLOG PROTEIN 1 (PTHR45836:SF12)	-	Homo sapiens	No
21	HUMAN|HGNC=11766|UniProtKB=P01137	TGFB1	Transforming growth factor beta-1 proprotein	TRANSFORMING GROWTH FACTOR BETA-1 PROPROTEIN (PTHR11848:SF125)	growth factor	Homo sapiens	No
22	HUMAN|HGNC=3512|UniProtKB=Q16394	EXT1	Exostosin-1	EXOSTOSIN-1 (PTHR11062:SF97)	glycosyltransferase	Homo sapiens	No
