# Supplementary material for: Transcriptomic and functional analysis of ANGPTL4 overexpression in pancreatic cancer nominates targets that reverse chemoresistance
Source: BMC Cancer. 2023 Jun 8;23:524. doi: 10.1186/s12885-023-11010-1 (PMC10251551; doi:10.1186/s12885-023-11010-1)
Supplement: Supplementary file 7 — Additional file 7: Table S5.txt [file 12885_2023_11010_MOESM7_ESM.pdf]

Supplementary Table 5: AMIGO2 Panther GO biological process complete analysis for DEG retrieved from with or without gemcitabine analysis

Analysis Type:	PANTHER Overrepresentation Test (Released 20221013)
Annotation Version and Release Date:	GO Ontology database DOI: 10.5281/zenodo.7709866 Released 2023-03-06
Analyzed List:	upload_1 (Homo sapiens)
Reference List:	Homo sapiens (all genes in database)
Test Type:	FISHER
Correction:	FDR

GO biological process complete	Homo sapiens REFLIST (20589)	upload_1 (941)	upload_1 (expected)	upload_1 (over/ under)	upload_1 (fold Enrichment)	upload_1 (raw P-value)	upload_1 (FDR)	Genes from Gemcitabine Treatment DEG List
biological_process (GO:0008150)	17752	877	811.34	+	1.08	4.45E-11	2.24E-08	yes
cellular process (GO:0009987)	14600	757	667.28	+	1.13	7.36E-11	3.38E-08	yes
biological regulation (GO:0065007)	12190	675	557.13	+	1.21	6.77E-15	7.05E-12	yes
regulation of biological process (GO:0050789)	11772	668	538.03	+	1.24	1.58E-17	1.23E-13	yes
regulation of cellular process (GO:0050794)	11006	633	503.02	+	1.26	4.20E-17	1.31E-13	yes
response to stimulus (GO:0050896)	8196	462	374.59	+	1.23	1.88E-08	3.81E-06	yes
metabolic process (GO:0008152)	8105	423	370.43	+	1.14	7.33E-04	2.75E-02	yes
regulation of metabolic process (GO:0019222)	6681	412	305.35	+	1.35	1.39E-12	1.03E-09	yes
positive regulation of biological process (GO:0048518)	6234	412	284.92	+	1.45	1.89E-17	9.84E-14	yes
regulation of macromolecule metabolic process (GO:0060255)	6162	388	281.63	+	1.38	7.46E-13	6.46E-10	yes
primary metabolic process (GO:0044238)	7199	385	329.02	+	1.17	2.15E-04	1.03E-02	yes
positive regulation of cellular process (GO:0048522)	5523	378	252.42	+	1.5	4.93E-18	7.69E-14	yes
cellular response to stimulus (GO:0051716)	6443	376	294.47	+	1.28	4.74E-08	8.60E-06	yes
developmental process (GO:0032502)	5732	376	261.98	+	1.44	5.77E-15	6.43E-12	yes
multicellular organismal process (GO:0032501)	6712	375	306.77	+	1.22	5.41E-06	5.25E-04	yes
negative regulation of biological process (GO:0048519)	5336	364	243.88	+	1.49	5.99E-17	1.56E-13	yes
regulation of primary metabolic process (GO:0080090)	5797	363	264.95	+	1.37	2.02E-11	1.05E-08	yes
nitrogen compound metabolic process (GO:0006807)	6649	358	303.89	+	1.18	2.81E-04	1.27E-02	yes
regulation of nitrogen compound metabolic process (GO:0051171)	5624	356	257.04	+	1.39	7.96E-12	5.17E-09	yes
anatomical structure development (GO:0048856)	5193	350	237.34	+	1.47	2.72E-15	4.25E-12	yes
cellular component organization or biogenesis (GO:0071840)	5700	344	260.51	+	1.32	8.05E-09	1.77E-06	yes
regulation of cellular metabolic process (GO:0031323)	5447	338	248.95	+	1.36	4.79E-10	1.63E-07	yes
cellular component organization (GO:0016043)	5497	338	251.23	+	1.35	1.39E-09	3.73E-07	yes
negative regulation of cellular process (GO:0048523)	4735	326	216.41	+	1.51	3.39E-15	4.81E-12	yes
cellular metabolic process (GO:0044237)	5808	312	265.45	+	1.18	1.14E-03	3.78E-02	yes

cell communication (GO:0007154)	5244	311	239.67	+	1.3	3.84E-07	5.21E-05	yes
signaling (GO:0023052)	5081	305	232.22	+	1.31	1.90E-07	2.85E-05	yes
signal transduction (GO:0007165)	4783	289	218.6	+	1.32	3.04E-07	4.20E-05	yes
regulation of gene expression (GO:0010468)	4847	288	221.53	+	1.3	1.42E-06	1.70E-04	yes
regulation of response to stimulus (GO:0048583)	3954	285	180.71	+	1.58	2.05E-15	3.56E-12	yes
multicellular organism development (GO:0007275)	3969	275	181.4	+	1.52	7.95E-13	6.53E-10	yes
positive regulation of metabolic process (GO:0009893)	3834	263	175.23	+	1.5	1.12E-11	6.45E-09	yes
regulation of cell communication (GO:0010646)	3402	256	155.49	+	1.65	1.10E-15	2.14E-12	yes
regulation of signaling (GO:0023051)	3393	253	155.07	+	1.63	4.47E-15	5.37E-12	yes
system development (GO:0048731)	3553	248	162.39	+	1.53	9.55E-12	5.73E-09	yes
positive regulation of macromolecule metabolic process (GO:0010604)	3524	248	161.06	+	1.54	4.34E-12	3.08E-09	yes
regulation of biosynthetic process (GO:0009889)	4135	242	188.99	+	1.28	4.76E-05	3.00E-03	yes
response to stress (GO:0006950)	3407	237	155.71	+	1.52	5.02E-11	2.37E-08	yes
regulation of nucleobase-containing compound metabolic process (GO:0019219)	4016	237	183.55	+	1.29	3.28E-05	2.24E-03	yes
regulation of cellular biosynthetic process (GO:0031326)	4026	235	184	+	1.28	6.90E-05	3.94E-03	yes
cellular developmental process (GO:0048869)	3599	233	164.49	+	1.42	4.54E-08	8.33E-06	yes
cell differentiation (GO:0030154)	3575	232	163.39	+	1.42	4.18E-08	7.76E-06	yes
response to chemical (GO:0042221)	3899	231	178.2	+	1.3	3.24E-05	2.23E-03	yes
regulation of macromolecule biosynthetic process (GO:0010556)	3917	230	179.02	+	1.28	5.81E-05	3.46E-03	yes
regulation of signal transduction (GO:0009966)	2990	229	136.66	+	1.68	1.05E-14	1.03E-11	yes
positive regulation of nitrogen compound metabolic process (GO:0051173)	3128	221	142.96	+	1.55	9.23E-11	4.00E-08	yes
animal organ development (GO:0048513)	2925	220	133.68	+	1.65	2.45E-13	2.25E-10	yes
regulation of RNA metabolic process (GO:0051252)	3727	217	170.34	+	1.27	1.75E-04	8.60E-03	yes
regulation of molecular function (GO:0065009)	2567	214	117.32	+	1.82	2.81E-17	1.10E-13	yes
regulation of multicellular organismal process (GO:0051239)	2980	210	136.2	+	1.54	3.50E-10	1.30E-07	yes
positive regulation of cellular metabolic process (GO:0031325)	2971	207	135.79	+	1.52	1.46E-09	3.79E-07	yes
regulation of RNA biosynthetic process (GO:2001141)	3431	206	156.81	+	1.31	4.99E-05	3.09E-03	yes
regulation of DNA-templated transcription (GO:0006355)	3406	205	155.67	+	1.32	4.73E-05	3.00E-03	yes
regulation of nucleic acid-templated transcription (GO:1903506)	3408	205	155.76	+	1.32	4.76E-05	3.01E-03	yes
regulation of cellular component organization (GO:0051128)	2445	199	111.75	+	1.78	4.14E-15	5.39E-12	yes
organelle organization (GO:0006996)	3050	195	139.4	+	1.4	1.89E-06	2.17E-04	yes
negative regulation of metabolic process (GO:0009892)	2976	193	136.02	+	1.42	9.13E-07	1.18E-04	yes
regulation of developmental process (GO:0050793)	2460	188	112.43	+	1.67	7.16E-12	4.86E-09	yes
response to organic substance (GO:0010033)	2590	186	118.37	+	1.57	1.40E-09	3.69E-07	yes

negative regulation of macromolecule metabolic process (GO:0010605)	2746	185	125.5	+	1.47	1.36E-07	2.12E-05	yes
regulation of protein metabolic process (GO:0051246)	2486	182	113.62	+	1.6	5.19E-10	1.72E-07	yes
anatomical structure morphogenesis (GO:0009653)	2233	178	102.06	+	1.74	1.25E-12	9.73E-10	yes
regulation of catalytic activity (GO:0050790)	1825	167	83.41	+	2	1.01E-16	2.26E-13	yes
response to external stimulus (GO:0009605)	2410	167	110.15	+	1.52	1.40E-07	2.15E-05	yes
positive regulation of response to stimulus (GO:0048584)	2197	165	100.41	+	1.64	6.70E-10	2.13E-07	yes
macromolecule modification (GO:0043412)	2796	165	127.79	+	1.29	8.25E-04	3.01E-02	yes
negative regulation of nitrogen compound metabolic process (GO:0051172)	2375	158	108.55	+	1.46	2.99E-06	3.07E-04	yes
protein modification process (GO:0036211)	2573	156	117.6	+	1.33	3.67E-04	1.61E-02	yes
cellular response to chemical stimulus (GO:0070887)	2405	154	109.92	+	1.4	3.56E-05	2.40E-03	yes
cell development (GO:0048468)	2169	151	99.13	+	1.52	5.02E-07	6.70E-05	yes
nervous system development (GO:0007399)	2222	150	101.55	+	1.48	2.60E-06	2.72E-04	yes
macromolecule localization (GO:0033036)	2368	149	108.23	+	1.38	9.68E-05	5.27E-03	yes
regulation of transcription by RNA polymerase II (GO:0006357)	2418	147	110.51	+	1.33	5.50E-04	2.23E-02	yes
tissue development (GO:0009888)	1745	145	79.75	+	1.82	1.69E-11	9.08E-09	yes
regulation of localization (GO:0032879)	2133	144	97.49	+	1.48	4.82E-06	4.80E-04	yes
positive regulation of biosynthetic process (GO:0009891)	2073	144	94.74	+	1.52	9.57E-07	1.22E-04	yes
regulation of cell population proliferation (GO:0042127)	1676	141	76.6	+	1.84	1.17E-11	6.50E-09	yes
immune system process (GO:0002376)	2256	140	103.11	+	1.36	3.06E-04	1.37E-02	yes
negative regulation of cellular metabolic process (GO:0031324)	2214	139	101.19	+	1.37	2.18E-04	1.04E-02	yes
regulation of intracellular signal transduction (GO:1902531)	1727	137	78.93	+	1.74	1.08E-09	3.06E-07	yes
positive regulation of cellular biosynthetic process (GO:0031328)	2014	137	92.05	+	1.49	6.11E-06	5.82E-04	yes
positive regulation of cell communication (GO:0010647)	1729	137	79.02	+	1.73	1.12E-09	3.11E-07	yes
positive regulation of nucleobase-containing compound metabolic process (GO:0045935)	2044	137	93.42	+	1.47	1.18E-05	1.02E-03	yes
positive regulation of signaling (GO:0023056)	1728	136	78.98	+	1.72	2.24E-09	5.37E-07	yes
cellular response to organic substance (GO:0071310)	1776	134	81.17	+	1.65	2.98E-08	5.81E-06	yes
positive regulation of macromolecule biosynthetic process (GO:0010557)	1934	133	88.39	+	1.5	5.41E-06	5.28E-04	yes
cell surface receptor signaling pathway (GO:0007166)	2087	130	95.38	+	1.36	5.31E-04	2.17E-02	yes
regulation of cell differentiation (GO:0045595)	1556	127	71.12	+	1.79	1.17E-09	3.19E-07	yes
regulation of protein modification process (GO:0031399)	1471	126	67.23	+	1.87	7.85E-11	3.50E-08	yes
phosphorus metabolic process (GO:0006793)	1841	125	84.14	+	1.49	1.73E-05	1.33E-03	yes
intracellular signal transduction (GO:0035556)	1520	125	69.47	+	1.8	8.19E-10	2.51E-07	yes
phosphate-containing compound metabolic process (GO:0006796)	1815	124	82.95	+	1.49	1.51E-05	1.19E-03	yes

positive regulation of signal transduction (GO:0009967)	1551	123	70.89	+	1.74	1.09E-08	2.30E-06	yes
positive regulation of molecular function (GO:0044093)	1517	123	69.33	+	1.77	2.58E-09	6.09E-07	yes
regulation of transport (GO:0051049)	1776	122	81.17	+	1.5	1.27E-05	1.09E-03	yes
positive regulation of RNA metabolic process (GO:0051254)	1847	121	84.42	+	1.43	1.27E-04	6.60E-03	yes
regulation of cell death (GO:0010941)	1668	121	76.23	+	1.59	1.19E-06	1.45E-04	yes
regulation of multicellular organismal development (GO:2000026)	1399	120	63.94	+	1.88	1.74E-10	6.98E-08	yes
positive regulation of RNA biosynthetic process (GO:1902680)	1714	118	78.34	+	1.51	2.03E-05	1.51E-03	yes
positive regulation of nucleic acid-templated transcription (GO:1903508)	1707	118	78.02	+	1.51	1.51E-05	1.20E-03	yes
positive regulation of DNA-templated transcription (GO:0045893)	1707	118	78.02	+	1.51	1.51E-05	1.20E-03	yes
positive regulation of multicellular organismal process (GO:0051240)	1653	116	75.55	+	1.54	8.69E-06	7.75E-04	yes
response to oxygen-containing compound (GO:1901700)	1679	114	76.74	+	1.49	4.91E-05	3.05E-03	yes
positive regulation of protein metabolic process (GO:0051247)	1446	113	66.09	+	1.71	1.05E-07	1.66E-05	yes
regulation of phosphorus metabolic process (GO:0051174)	1323	113	60.47	+	1.87	8.11E-10	2.53E-07	yes
regulation of phosphate metabolic process (GO:0019220)	1322	112	60.42	+	1.85	1.76E-09	4.36E-07	yes
negative regulation of response to stimulus (GO:0048585)	1640	112	74.95	+	1.49	4.13E-05	2.68E-03	yes
regulation of programmed cell death (GO:0043067)	1510	112	69.01	+	1.62	1.35E-06	1.63E-04	yes
regulation of response to stress (GO:0080134)	1451	112	66.32	+	1.69	2.21E-07	3.29E-05	yes
cellular response to stress (GO:0033554)	1610	110	73.58	+	1.49	4.68E-05	2.98E-03	yes
cell cycle (GO:0007049)	1267	110	57.91	+	1.9	5.78E-10	1.88E-07	yes
negative regulation of biosynthetic process (GO:0009890)	1638	108	74.86	+	1.44	2.36E-04	1.10E-02	yes
regulation of apoptotic process (GO:0042981)	1466	108	67	+	1.61	2.58E-06	2.72E-04	yes
negative regulation of cellular biosynthetic process (GO:0031327)	1589	107	72.62	+	1.47	1.17E-04	6.24E-03	yes
negative regulation of nucleobase-containing compound metabolic process (GO:0045934)	1560	107	71.3	+	1.5	6.07E-05	3.59E-03	yes
biological process involved in interspecies interaction between organisms (GO:0044419)	1542	106	70.48	+	1.5	5.58E-05	3.38E-03	yes
regulation of immune system process (GO:0002682)	1480	105	67.64	+	1.55	1.73E-05	1.33E-03	yes
negative regulation of macromolecule biosynthetic process (GO:0010558)	1547	104	70.7	+	1.47	1.60E-04	8.00E-03	yes
regulation of cell cycle (GO:0051726)	1120	102	51.19	+	1.99	3.20E-10	1.22E-07	yes
regulation of phosphorylation (GO:0042325)	1177	102	53.79	+	1.9	3.18E-09	7.40E-07	yes
positive regulation of developmental process (GO:0051094)	1314	101	60.06	+	1.68	1.06E-06	1.33E-04	yes
regulation of organelle organization (GO:0033043)	1176	99	53.75	+	1.84	2.54E-08	5.01E-06	yes
response to biotic stimulus (GO:0009607)	1411	99	64.49	+	1.54	4.79E-05	3.00E-03	yes
defense response (GO:0006952)	1417	98	64.76	+	1.51	8.65E-05	4.74E-03	yes
regulation of protein phosphorylation (GO:0001932)	1079	97	49.31	+	1.97	1.67E-09	4.27E-07	yes

response to other organism (GO:0051707)	1363	97	62.29	+	1.56	3.59E-05	2.40E-03	yes
negative regulation of RNA metabolic process (GO:0051253)	1441	97	65.86	+	1.47	2.73E-04	1.25E-02	yes
response to external biotic stimulus (GO:0043207)	1366	97	62.43	+	1.55	3.69E-05	2.41E-03	yes
negative regulation of RNA biosynthetic process (GO:1902679)	1330	96	60.79	+	1.58	2.19E-05	1.60E-03	yes
positive regulation of catalytic activity (GO:0043085)	1110	96	50.73	+	1.89	1.07E-08	2.28E-06	yes
negative regulation of nucleic acid-templated transcription (GO:1903507)	1316	96	60.15	+	1.6	1.47E-05	1.18E-03	yes
negative regulation of DNA-templated transcription (GO:0045892)	1315	96	60.1	+	1.6	1.46E-05	1.18E-03	yes
regulation of locomotion (GO:0040012)	1034	94	47.26	+	1.99	2.01E-09	4.90E-07	yes
regulation of cell motility (GO:2000145)	989	92	45.2	+	2.04	8.93E-10	2.63E-07	yes
positive regulation of cellular component organization (GO:0051130)	1122	92	51.28	+	1.79	2.25E-07	3.28E-05	yes
epithelium development (GO:0060429)	1098	91	50.18	+	1.81	1.69E-07	2.56E-05	yes
neurogenesis (GO:0022008)	1310	90	59.87	+	1.5	2.45E-04	1.13E-02	yes
cytoskeleton organization (GO:0007010)	1247	89	56.99	+	1.56	7.48E-05	4.24E-03	yes
positive regulation of transcription by RNA polymerase II (GO:0045944)	1256	89	57.4	+	1.55	8.16E-05	4.52E-03	yes
regulation of cell migration (GO:0030334)	930	88	42.5	+	2.07	8.34E-10	2.50E-07	yes
animal organ morphogenesis (GO:0009887)	989	86	45.2	+	1.9	5.84E-08	1.03E-05	yes
cell death (GO:0008219)	1122	86	51.28	+	1.68	8.49E-06	7.66E-04	yes
cell cycle process (GO:0022402)	878	86	40.13	+	2.14	2.75E-10	1.07E-07	yes
regulation of response to external stimulus (GO:0032101)	1058	85	48.35	+	1.76	1.58E-06	1.86E-04	yes
embryo development (GO:0009790)	1056	85	48.26	+	1.76	1.54E-06	1.82E-04	yes
regulation of transferase activity (GO:0051338)	851	84	38.89	+	2.16	3.97E-10	1.41E-07	yes
programmed cell death (GO:0012501)	1088	84	49.73	+	1.69	8.84E-06	7.84E-04	yes
response to abiotic stimulus (GO:0009628)	1117	84	51.05	+	1.65	2.21E-05	1.59E-03	yes
tube development (GO:0035295)	897	84	41	+	2.05	3.27E-09	7.50E-07	yes
regulation of anatomical structure morphogenesis (GO:0022603)	871	84	39.81	+	2.11	1.06E-09	3.07E-07	yes
apoptotic process (GO:0006915)	1045	83	47.76	+	1.74	2.99E-06	3.09E-04	yes
negative regulation of signal transduction (GO:0009968)	1265	83	57.82	+	1.44	1.51E-03	4.80E-02	yes
positive regulation of gene expression (GO:0010628)	1184	83	54.11	+	1.53	2.17E-04	1.03E-02	yes
positive regulation of cell population proliferation (GO:0008284)	952	83	43.51	+	1.91	8.37E-08	1.39E-05	yes
regulation of cell development (GO:0060284)	827	82	37.8	+	2.17	4.31E-10	1.49E-07	yes
negative regulation of multicellular organismal process (GO:0051241)	1122	82	51.28	+	1.6	6.11E-05	3.60E-03	yes
cellular response to oxygen-containing compound (GO:1901701)	1150	81	52.56	+	1.54	2.34E-04	1.09E-02	yes
anatomical structure formation involved in morphogenesis (GO:0048646)	959	81	43.83	+	1.85	4.85E-07	6.52E-05	yes
positive regulation of protein modification process (GO:0031401)	954	81	43.6	+	1.86	3.23E-07	4.42E-05	yes
negative regulation of cell death (GO:0060548)	1022	81	46.71	+	1.73	4.76E-06	4.76E-04	yes

positive regulation of intracellular signal transduction (GO:1902533)	1005	80	45.93	+	1.74	4.17E-06	4.19E-04	yes
regulation of hydrolase activity (GO:0051336)	966	80	44.15	+	1.81	1.19E-06	1.46E-04	yes
positive regulation of cell differentiation (GO:0045597)	854	78	39.03	+	2	4.08E-08	7.66E-06	yes
circulatory system development (GO:0072359)	920	77	42.05	+	1.83	1.08E-06	1.35E-04	yes
cell adhesion (GO:0007155)	960	77	43.88	+	1.75	5.29E-06	5.19E-04	yes
negative regulation of molecular function (GO:0044092)	985	77	45.02	+	1.71	1.38E-05	1.15E-03	yes
defense response to other organism (GO:0098542)	997	77	45.57	+	1.69	2.18E-05	1.60E-03	yes
response to nitrogen compound (GO:1901698)	1057	76	48.31	+	1.57	2.33E-04	1.10E-02	yes
central nervous system development (GO:0007417)	1055	74	48.22	+	1.53	5.44E-04	2.22E-02	yes
negative regulation of transcription by RNA polymerase II (GO:0000122)	987	74	45.11	+	1.64	7.65E-05	4.33E-03	yes
cell motility (GO:0048870)	1040	72	47.53	+	1.51	8.71E-04	3.14E-02	yes
regulation of cellular component biogenesis (GO:0044087)	967	72	44.2	+	1.63	1.24E-04	6.52E-03	yes
negative regulation of programmed cell death (GO:0043069)	919	72	42	+	1.71	2.20E-05	1.59E-03	yes
positive regulation of phosphorus metabolic process (GO:0010562)	848	72	38.76	+	1.86	2.01E-06	2.21E-04	yes
positive regulation of phosphate metabolic process (GO:0045937)	848	72	38.76	+	1.86	2.01E-06	2.19E-04	yes
regulation of kinase activity (GO:0043549)	713	71	32.59	+	2.18	6.39E-09	1.42E-06	yes
regulation of cell adhesion (GO:0030155)	789	71	36.06	+	1.97	2.43E-07	3.44E-05	yes
response to cytokine (GO:0034097)	817	70	37.34	+	1.87	2.00E-06	2.21E-04	yes
cell migration (GO:0016477)	875	70	39.99	+	1.75	1.95E-05	1.46E-03	yes
negative regulation of apoptotic process (GO:0043066)	892	70	40.77	+	1.72	3.32E-05	2.24E-03	yes
mitotic cell cycle (GO:0000278)	616	69	28.15	+	2.45	1.10E-10	4.53E-08	yes
response to lipid (GO:0033993)	851	69	38.89	+	1.77	1.51E-05	1.19E-03	yes
response to organonitrogen compound (GO:0010243)	983	68	44.93	+	1.51	1.13E-03	3.75E-02	yes
positive regulation of transport (GO:0051050)	924	68	42.23	+	1.61	2.36E-04	1.10E-02	yes
positive regulation of phosphorylation (GO:0042327)	774	68	35.37	+	1.92	1.09E-06	1.35E-04	yes
regulation of cell cycle process (GO:0010564)	726	67	33.18	+	2.02	2.99E-07	4.17E-05	yes
response to organic cyclic compound (GO:0014070)	871	66	39.81	+	1.66	1.53E-04	7.74E-03	yes
phosphorylation (GO:0016310)	883	66	40.36	+	1.64	2.33E-04	1.10E-02	yes
tube morphogenesis (GO:0035239)	681	66	31.12	+	2.12	7.44E-08	1.29E-05	yes
regulation of protein kinase activity (GO:0045859)	619	66	28.29	+	2.33	1.72E-09	4.33E-07	yes
mitotic cell cycle process (GO:1903047)	522	65	23.86	+	2.72	8.52E-12	5.32E-09	yes
positive regulation of protein phosphorylation (GO:0001934)	714	65	32.63	+	1.99	6.34E-07	8.38E-05	yes
DNA metabolic process (GO:0006259)	789	65	36.06	+	1.8	1.59E-05	1.25E-03	yes
hemopoiesis (GO:0030097)	684	65	31.26	+	2.08	1.40E-07	2.16E-05	yes
regulation of cell projection organization (GO:0031344)	657	64	30.03	+	2.13	7.72E-08	1.32E-05	yes

Unclassified (UNCLASSIFIED)	2837	64	129.66	-	0.49	4.45E-11	2.17E-08	yes
negative regulation of cellular component organization (GO:0051129)	711	63	32.5	+	1.94	2.37E-06	2.51E-04	yes
regulation of plasma membrane bounded cell projection organization (GO:0120035)	643	63	29.39	+	2.14	9.16E-08	1.50E-05	yes
cell division (GO:0051301)	521	62	23.81	+	2.6	1.00E-10	4.23E-08	yes
cellular response to cytokine stimulus (GO:0071345)	719	61	32.86	+	1.86	1.04E-05	9.04E-04	yes
innate immune response (GO:0045087)	752	61	34.37	+	1.77	4.85E-05	3.03E-03	yes
regulation of defense response (GO:0031347)	748	61	34.19	+	1.78	3.30E-05	2.24E-03	yes
cell population proliferation (GO:0008283)	728	60	33.27	+	1.8	3.68E-05	2.42E-03	yes
regulation of cytokine production (GO:0001817)	779	59	35.6	+	1.66	3.70E-04	1.62E-02	yes
negative regulation of cell population proliferation (GO:0008285)	708	59	32.36	+	1.82	2.89E-05	2.02E-03	yes
cell activation (GO:0001775)	724	58	33.09	+	1.75	8.03E-05	4.49E-03	yes
embryo development ending in birth or egg hatching (GO:0009792)	684	58	31.26	+	1.86	2.11E-05	1.56E-03	yes
protein phosphorylation (GO:0006468)	708	56	32.36	+	1.73	1.93E-04	9.36E-03	yes
chordate embryonic development (GO:0043009)	662	56	30.26	+	1.85	3.59E-05	2.40E-03	yes
regulation of proteolysis (GO:0030162)	711	56	32.5	+	1.72	1.99E-04	9.58E-03	yes
positive regulation of cell motility (GO:2000147)	559	54	25.55	+	2.11	1.03E-06	1.30E-04	yes
positive regulation of locomotion (GO:0040017)	575	54	26.28	+	2.05	2.35E-06	2.51E-04	yes
negative regulation of catalytic activity (GO:0043086)	636	53	29.07	+	1.82	8.65E-05	4.75E-03	yes
positive regulation of transferase activity (GO:0051347)	524	51	23.95	+	2.13	2.11E-06	2.29E-04	yes
chromosome organization (GO:0051276)	456	51	20.84	+	2.45	3.19E-08	6.15E-06	yes
positive regulation of cell migration (GO:0030335)	534	51	24.41	+	2.09	2.82E-06	2.94E-04	yes
negative regulation of cell differentiation (GO:0045596)	671	50	30.67	+	1.63	1.50E-03	4.80E-02	yes
leukocyte activation (GO:0045321)	601	50	27.47	+	1.82	1.24E-04	6.50E-03	yes
regulation of cell activation (GO:0050865)	652	50	29.8	+	1.68	6.81E-04	2.63E-02	yes
epithelial cell differentiation (GO:0030855)	631	50	28.84	+	1.73	3.77E-04	1.62E-02	yes
regulation of mitotic cell cycle (GO:0007346)	495	49	22.62	+	2.17	1.89E-06	2.15E-04	yes
regulation of growth (GO:0040008)	625	49	28.57	+	1.72	5.15E-04	2.11E-02	yes
cell-cell adhesion (GO:0098609)	539	49	24.63	+	1.99	2.07E-05	1.53E-03	yes
gland development (GO:0048732)	425	48	19.42	+	2.47	6.91E-08	1.21E-05	yes
vasculature development (GO:0001944)	537	47	24.54	+	1.92	5.43E-05	3.30E-03	yes
cellular response to lipid (GO:0071396)	527	47	24.09	+	1.95	4.25E-05	2.74E-03	yes
embryonic morphogenesis (GO:0048598)	592	47	27.06	+	1.74	5.25E-04	2.15E-02	yes
actin filament-based process (GO:0030029)	601	47	27.47	+	1.71	8.16E-04	2.99E-02	yes
sensory organ development (GO:0007423)	593	46	27.1	+	1.7	1.07E-03	3.62E-02	yes
regulation of cell cycle phase transition (GO:1901987)	435	46	19.88	+	2.31	6.96E-07	9.12E-05	yes
blood vessel development (GO:0001568)	517	46	23.63	+	1.95	5.66E-05	3.40E-03	yes
tissue morphogenesis (GO:0048729)	571	46	26.1	+	1.76	4.24E-04	1.80E-02	yes

regulation of cytoskeleton organization (GO:0051493)	521	46	23.81	+	1.93	6.12E-05	3.59E-03	yes
regulation of leukocyte activation (GO:0002694)	595	46	27.19	+	1.69	1.10E-03	3.69E-02	yes
regulation of nervous system development (GO:0051960)	467	45	21.34	+	2.11	8.86E-06	7.81E-04	yes
response to virus (GO:0009615)	367	45	16.77	+	2.68	2.53E-08	5.05E-06	yes
negative regulation of immune system process (GO:0002683)	480	45	21.94	+	2.05	1.84E-05	1.40E-03	yes
leukocyte differentiation (GO:0002521)	424	44	19.38	+	2.27	1.82E-06	2.10E-04	yes
embryonic organ development (GO:0048568)	459	44	20.98	+	2.1	1.26E-05	1.08E-03	yes
regulation of neuron projection development (GO:0010975)	450	44	20.57	+	2.14	9.50E-06	8.33E-04	yes
regulation of vesicle-mediated transport (GO:0060627)	536	44	24.5	+	1.8	4.08E-04	1.75E-02	yes
heart development (GO:0007507)	560	44	25.59	+	1.72	1.11E-03	3.72E-02	yes
regulation of protein serine/threonine kinase activity (GO:0071900)	368	43	16.82	+	2.56	1.29E-07	2.03E-05	yes
locomotion (GO:0040011)	541	43	24.73	+	1.74	9.20E-04	3.28E-02	yes
actin cytoskeleton organization (GO:0030036)	540	43	24.68	+	1.74	9.08E-04	3.24E-02	yes
blood vessel morphogenesis (GO:0048514)	429	42	19.61	+	2.14	1.72E-05	1.33E-03	yes
positive regulation of cell development (GO:0010720)	439	42	20.06	+	2.09	2.20E-05	1.60E-03	yes
regulation of response to biotic stimulus (GO:0002831)	478	42	21.85	+	1.92	1.26E-04	6.56E-03	yes
positive regulation of cell adhesion (GO:0045785)	486	41	22.21	+	1.85	3.39E-04	1.51E-02	yes
positive regulation of kinase activity (GO:0033674)	431	41	19.7	+	2.08	3.11E-05	2.15E-03	yes
skeletal system development (GO:0001501)	518	41	23.67	+	1.73	1.50E-03	4.79E-02	yes
response to growth factor (GO:0070848)	509	41	23.26	+	1.76	9.44E-04	3.30E-02	yes
regulation of lymphocyte activation (GO:0051249)	503	41	22.99	+	1.78	8.74E-04	3.13E-02	yes
lymphocyte activation (GO:0046649)	478	41	21.85	+	1.88	2.83E-04	1.28E-02	yes
negative regulation of cell cycle (GO:0045786)	363	40	16.59	+	2.41	1.53E-06	1.82E-04	yes
regulation of actin filament-based process (GO:0032970)	390	40	17.82	+	2.24	7.10E-06	6.60E-04	yes
regulation of mitotic cell cycle phase transition (GO:1901990)	334	40	15.27	+	2.62	2.32E-07	3.35E-05	yes
regulation of anatomical structure size (GO:0090066)	503	40	22.99	+	1.74	1.31E-03	4.24E-02	yes
regulation of neurogenesis (GO:0050767)	386	40	17.64	+	2.27	5.98E-06	5.73E-04	yes
cellular response to growth factor stimulus (GO:0071363)	478	39	21.85	+	1.79	9.74E-04	3.38E-02	yes
regulation of hemopoiesis (GO:1903706)	401	39	18.33	+	2.13	2.86E-05	2.01E-03	yes
cytokine-mediated signaling pathway (GO:0019221)	372	38	17	+	2.24	1.34E-05	1.13E-03	yes
chromosome segregation (GO:0007059)	325	38	14.85	+	2.56	7.15E-07	9.30E-05	yes
response to wounding (GO:0009611)	453	38	20.7	+	1.84	7.05E-04	2.70E-02	yes
regulation of protein-containing complex assembly (GO:0043254)	422	38	19.29	+	1.97	1.83E-04	8.94E-03	yes
defense response to symbiont (GO:0140546)	266	37	12.16	+	3.04	1.76E-08	3.61E-06	yes
defense response to virus (GO:0051607)	265	37	12.11	+	3.05	1.61E-08	3.35E-06	yes

positive regulation of cell projection organization (GO:0031346)	354	37	16.18	+	2.29	1.35E-05	1.13E-03	yes
positive regulation of protein kinase activity (GO:0045860)	365	37	16.68	+	2.22	2.01E-05	1.50E-03	yes
angiogenesis (GO:0001525)	336	36	15.36	+	2.34	8.53E-06	7.65E-04	yes
growth (GO:0040007)	419	36	19.15	+	1.88	6.63E-04	2.57E-02	yes
developmental growth (GO:0048589)	419	36	19.15	+	1.88	6.63E-04	2.56E-02	yes
regulation of peptidase activity (GO:0052547)	408	35	18.65	+	1.88	8.62E-04	3.12E-02	yes
negative regulation of response to external stimulus (GO:0032102)	416	35	19.01	+	1.84	1.01E-03	3.47E-02	yes
regulation of binding (GO:0051098)	370	35	16.91	+	2.07	1.20E-04	6.37E-03	yes
regulation of innate immune response (GO:0045088)	385	34	17.6	+	1.93	6.07E-04	2.42E-02	yes
regulation of cellular component size (GO:0032535)	366	34	16.73	+	2.03	2.69E-04	1.23E-02	yes
negative regulation of locomotion (GO:0040013)	338	34	15.45	+	2.2	5.24E-05	3.22E-03	yes
positive regulation of defense response (GO:0031349)	389	34	17.78	+	1.91	6.62E-04	2.57E-02	yes
lipid localization (GO:0010876)	383	34	17.5	+	1.94	5.85E-04	2.35E-02	yes
negative regulation of organelle organization (GO:0010639)	341	34	15.59	+	2.18	5.90E-05	3.50E-03	yes
regulation of supramolecular fiber organization (GO:1902903)	381	34	17.41	+	1.95	5.67E-04	2.29E-02	yes
regulation of epithelial cell proliferation (GO:0050678)	372	33	17	+	1.94	7.72E-04	2.86E-02	yes
axonogenesis (GO:0007409)	364	32	16.64	+	1.92	1.06E-03	3.60E-02	yes
G protein-coupled receptor signaling pathway (GO:0007186)	1230	32	56.22	-	0.57	6.23E-04	2.45E-02	yes
renal system development (GO:0072001)	313	32	14.31	+	2.24	8.05E-05	4.49E-03	yes
regulation of small GTPase mediated signal transduction (GO:0051056)	304	32	13.89	+	2.3	3.72E-05	2.42E-03	yes
organelle fission (GO:0048285)	334	32	15.27	+	2.1	2.32E-04	1.10E-02	yes
negative regulation of cell cycle process (GO:0010948)	275	32	12.57	+	2.55	6.26E-06	5.92E-04	yes
lipid transport (GO:0006869)	345	31	15.77	+	1.97	7.81E-04	2.88E-02	yes
kidney development (GO:0001822)	303	31	13.85	+	2.24	1.10E-04	5.89E-03	yes
regulation of actin cytoskeleton organization (GO:0032956)	348	31	15.9	+	1.95	8.41E-04	3.05E-02	yes
embryonic organ morphogenesis (GO:0048562)	302	31	13.8	+	2.25	1.08E-04	5.79E-03	yes
nuclear division (GO:0000280)	308	31	14.08	+	2.2	1.25E-04	6.53E-03	yes
mononuclear cell differentiation (GO:1903131)	335	30	15.31	+	1.96	1.04E-03	3.56E-02	yes
regulation of leukocyte differentiation (GO:1902105)	319	30	14.58	+	2.06	4.74E-04	1.98E-02	yes
negative regulation of cell motility (GO:2000146)	301	30	13.76	+	2.18	1.85E-04	8.97E-03	yes
nuclear chromosome segregation (GO:0098813)	235	30	10.74	+	2.79	1.94E-06	2.18E-04	yes
apoptotic signaling pathway (GO:0097190)	313	30	14.31	+	2.1	4.17E-04	1.78E-02	yes
wound healing (GO:0042060)	338	30	15.45	+	1.94	1.11E-03	3.72E-02	yes
negative regulation of cell adhesion (GO:0007162)	292	29	13.35	+	2.17	2.56E-04	1.17E-02	yes

regulation of endopeptidase activity (GO:0052548)	320	29	14.63	+	1.98	8.73E-04	3.14E-02	yes
negative regulation of cell migration (GO:0030336)	286	29	13.07	+	2.22	2.21E-04	1.05E-02	yes
regulation of epithelial cell migration (GO:0010632)	229	29	10.47	+	2.77	3.30E-06	3.35E-04	yes
regulation of chromosome organization (GO:0033044)	251	28	11.47	+	2.44	4.34E-05	2.77E-03	yes
response to decreased oxygen levels (GO:0036293)	288	28	13.16	+	2.13	4.14E-04	1.77E-02	yes
myeloid cell differentiation (GO:0030099)	285	28	13.03	+	2.15	3.76E-04	1.63E-02	yes
mesenchyme development (GO:0060485)	243	27	11.11	+	2.43	6.31E-05	3.69E-03	yes
negative regulation of cell cycle phase transition (GO:1901988)	229	27	10.47	+	2.58	3.28E-05	2.24E-03	yes
response to hypoxia (GO:0001666)	274	27	12.52	+	2.16	4.94E-04	2.04E-02	yes
cell cycle phase transition (GO:0044770)	175	27	8	+	3.38	2.40E-07	3.44E-05	yes
anterior/posterior pattern specification (GO:0009952)	209	27	9.55	+	2.83	5.12E-06	5.06E-04	yes
regulation of viral process (GO:0050792)	166	27	7.59	+	3.56	9.35E-08	1.52E-05	yes
regulation of cell morphogenesis (GO:0022604)	247	27	11.29	+	2.39	7.88E-05	4.42E-03	yes
mitotic cell cycle phase transition (GO:0044772)	164	26	7.5	+	3.47	2.51E-07	3.53E-05	yes
homeostasis of number of cells (GO:0048872)	270	26	12.34	+	2.11	7.86E-04	2.89E-02	yes
regulation of leukocyte proliferation (GO:0070663)	269	26	12.29	+	2.11	7.63E-04	2.84E-02	yes
gliogenesis (GO:0042063)	266	26	12.16	+	2.14	7.04E-04	2.71E-02	yes
axon guidance (GO:0007411)	232	25	10.6	+	2.36	1.75E-04	8.60E-03	yes
positive regulation of cell cycle process (GO:0090068)	253	25	11.56	+	2.16	9.02E-04	3.23E-02	yes
positive regulation of neurogenesis (GO:0050769)	240	25	10.97	+	2.28	3.55E-04	1.56E-02	yes
neuron projection guidance (GO:0097485)	232	25	10.6	+	2.36	1.75E-04	8.58E-03	yes
regulation of protein modification by small protein conjugation or removal (GO:1903320)	257	25	11.75	+	2.13	9.78E-04	3.38E-02	yes
regulation of chromosome segregation (GO:0051983)	132	24	6.03	+	3.98	7.80E-08	1.32E-05	yes
regulation of muscle system process (GO:0090257)	234	24	10.69	+	2.24	5.48E-04	2.23E-02	yes
regulation of protein ubiquitination (GO:0031396)	215	24	9.83	+	2.44	1.59E-04	7.93E-03	yes
cell cycle checkpoint signaling (GO:0000075)	161	24	7.36	+	3.26	1.90E-06	2.15E-04	yes
negative regulation of mitotic cell cycle (GO:0045930)	214	24	9.78	+	2.45	1.51E-04	7.70E-03	yes
positive regulation of protein serine/threonine kinase activity (GO:0071902)	197	23	9	+	2.55	1.53E-04	7.75E-03	yes
negative regulation of mitotic cell cycle phase transition (GO:1901991)	167	23	7.63	+	3.01	9.98E-06	8.70E-04	yes
mesenchymal cell differentiation (GO:0048762)	171	23	7.82	+	2.94	1.41E-05	1.16E-03	yes
mitotic nuclear division (GO:0140014)	161	23	7.36	+	3.13	5.83E-06	5.62E-04	yes
skeletal system morphogenesis (GO:0048705)	232	23	10.6	+	2.17	9.91E-04	3.41E-02	yes
regulation of cell-substrate adhesion (GO:0010810)	219	23	10.01	+	2.3	6.45E-04	2.52E-02	yes

regulation of response to cytokine stimulus (GO:0060759)	164	23	7.5	+	3.07	7.65E-06	7.03E-04	yes
regulation of muscle contraction (GO:0006937)	168	22	7.68	+	2.87	3.10E-05	2.15E-03	yes
regulation of myeloid cell differentiation (GO:0045637)	200	22	9.14	+	2.41	3.55E-04	1.57E-02	yes
response to mechanical stimulus (GO:0009612)	222	22	10.15	+	2.17	1.32E-03	4.27E-02	yes
positive regulation of epithelial cell proliferation (GO:0050679)	209	22	9.55	+	2.3	5.64E-04	2.28E-02	yes
regulation of MAP kinase activity (GO:0043405)	184	22	8.41	+	2.62	1.05E-04	5.68E-03	yes
positive regulation of protein-containing complex assembly (GO:0031334)	201	22	9.19	+	2.39	3.72E-04	1.61E-02	yes
regulation of viral life cycle (GO:1903900)	143	22	6.54	+	3.37	3.20E-06	3.26E-04	yes
sensory perception (GO:0007600)	995	22	45.48	-	0.48	1.52E-04	7.75E-03	yes
glial cell differentiation (GO:0010001)	206	21	9.42	+	2.23	1.45E-03	4.65E-02	yes
regulation of cytokine-mediated signaling pathway (GO:0001959)	153	21	6.99	+	3	2.51E-05	1.78E-03	yes
mitotic cell cycle checkpoint signaling (GO:0007093)	127	21	5.8	+	3.62	1.94E-06	2.16E-04	yes
spindle organization (GO:0007051)	163	21	7.45	+	2.82	5.76E-05	3.45E-03	yes
regulation of nuclear division (GO:0051783)	146	21	6.67	+	3.15	1.34E-05	1.13E-03	yes
stem cell differentiation (GO:0048863)	183	21	8.36	+	2.51	3.55E-04	1.57E-02	yes
sister chromatid segregation (GO:0000819)	139	21	6.35	+	3.31	6.83E-06	6.39E-04	yes
regulation of Ras protein signal transduction (GO:0046578)	190	21	8.68	+	2.42	4.71E-04	1.97E-02	yes
myeloid leukocyte differentiation (GO:0002573)	150	20	6.86	+	2.92	5.59E-05	3.37E-03	yes
negative regulation of viral process (GO:0048525)	94	20	4.3	+	4.66	1.04E-07	1.67E-05	yes
regulation of epithelial cell differentiation (GO:0030856)	154	20	7.04	+	2.84	7.76E-05	4.37E-03	yes
mitotic sister chromatid segregation (GO:0000070)	135	20	6.17	+	3.24	1.45E-05	1.19E-03	yes
regulation of endothelial cell migration (GO:0010594)	169	20	7.72	+	2.59	2.41E-04	1.12E-02	yes
regulation of cell cycle G1/S phase transition (GO:1902806)	192	20	8.78	+	2.28	1.12E-03	3.74E-02	yes
regulation of mitotic nuclear division (GO:0007088)	118	19	5.39	+	3.52	8.29E-06	7.52E-04	yes
morphogenesis of a branching structure (GO:0001763)	175	19	8	+	2.38	1.05E-03	3.60E-02	yes
kidney epithelium development (GO:0072073)	140	19	6.4	+	2.97	6.87E-05	3.94E-03	yes
regulation of G1/S transition of mitotic cell cycle (GO:2000045)	164	19	7.5	+	2.53	7.04E-04	2.70E-02	yes
negative regulation of lymphocyte activation (GO:0051250)	165	19	7.54	+	2.52	7.23E-04	2.75E-02	yes
positive regulation of epithelial cell migration (GO:0010634)	151	19	6.9	+	2.75	1.68E-04	8.32E-03	yes
morphogenesis of a branching epithelium (GO:0061138)	165	19	7.54	+	2.52	7.23E-04	2.74E-02	yes
regulation of cell cycle G2/M phase transition (GO:1902749)	112	18	5.12	+	3.52	1.46E-05	1.18E-03	yes
regulation of response to wounding (GO:1903034)	160	18	7.31	+	2.46	1.17E-03	3.83E-02	yes

regulation of sister chromatid segregation (GO:0033045)	106	18	4.84	+	3.72	7.47E-06	6.90E-04	yes
regulation of chromosome separation (GO:1905818)	74	18	3.38	+	5.32	7.97E-08	1.34E-05	yes
regulation of axonogenesis (GO:0050770)	157	18	7.18	+	2.51	1.07E-03	3.60E-02	yes
negative regulation of binding (GO:0051100)	162	18	7.4	+	2.43	1.25E-03	4.10E-02	yes
microtubule cytoskeleton organization involved in mitosis (GO:1902850)	130	18	5.94	+	3.03	8.41E-05	4.64E-03	yes
liver development (GO:0001889)	135	17	6.17	+	2.76	3.61E-04	1.59E-02	yes
nephron development (GO:0072006)	138	17	6.31	+	2.7	4.54E-04	1.91E-02	yes
regulation of viral genome replication (GO:0045069)	86	17	3.93	+	4.33	2.24E-06	2.41E-04	yes
regulation of metaphase/anaphase transition of cell cycle (GO:1902099)	94	17	4.3	+	3.96	6.39E-06	6.01E-04	yes
regulation of type I interferon production (GO:0032479)	119	17	5.44	+	3.13	9.32E-05	5.08E-03	yes
regulation of G2/M transition of mitotic cell cycle (GO:0010389)	100	17	4.57	+	3.72	1.31E-05	1.11E-03	yes
hepaticobiliary system development (GO:0061008)	138	17	6.31	+	2.7	4.54E-04	1.91E-02	yes
mitotic spindle organization (GO:0007052)	95	16	4.34	+	3.69	2.59E-05	1.83E-03	yes
regulation of animal organ morphogenesis (GO:2000027)	129	16	5.9	+	2.71	6.17E-04	2.44E-02	yes
mammary gland development (GO:0030879)	129	16	5.9	+	2.71	6.17E-04	2.43E-02	yes
regulation of cyclin-dependent protein serine/threonine kinase activity (GO:0000079)	112	16	5.12	+	3.13	1.48E-04	7.59E-03	yes
regulation of cell shape (GO:0008360)	140	16	6.4	+	2.5	1.36E-03	4.36E-02	yes
regulation of cyclin-dependent protein kinase activity (GO:1904029)	115	16	5.26	+	3.04	1.95E-04	9.41E-03	yes
regulation of mitotic metaphase/anaphase transition (GO:0030071)	91	16	4.16	+	3.85	1.62E-05	1.26E-03	yes
negative regulation of response to biotic stimulus (GO:0002832)	133	16	6.08	+	2.63	8.30E-04	3.02E-02	yes
nephron epithelium development (GO:0072009)	109	15	4.98	+	3.01	3.39E-04	1.51E-02	yes
negative regulation of chromosome organization (GO:2001251)	79	14	3.61	+	3.88	5.01E-05	3.09E-03	yes
mesonephric tubule development (GO:0072164)	89	14	4.07	+	3.44	1.55E-04	7.81E-03	yes
mesonephric epithelium development (GO:0072163)	89	14	4.07	+	3.44	1.55E-04	7.79E-03	yes
mesonephros development (GO:0001823)	93	14	4.25	+	3.29	2.33E-04	1.09E-02	yes
hematopoietic progenitor cell differentiation (GO:0002244)	106	14	4.84	+	2.89	7.62E-04	2.84E-02	yes
ureteric bud development (GO:0001657)	88	14	4.02	+	3.48	1.40E-04	7.22E-03	yes
negative regulation of viral genome replication (GO:0045071)	56	14	2.56	+	5.47	1.66E-06	1.93E-04	yes
positive regulation of type I interferon production (GO:0032481)	71	14	3.24	+	4.31	1.77E-05	1.36E-03	yes
gland morphogenesis (GO:0022612)	106	14	4.84	+	2.89	7.62E-04	2.84E-02	yes
neural crest cell differentiation (GO:0014033)	93	13	4.25	+	3.06	7.24E-04	2.74E-02	yes
stem cell development (GO:0048864)	86	13	3.93	+	3.31	3.72E-04	1.62E-02	yes
embryonic skeletal system morphogenesis (GO:0048704)	95	13	4.34	+	2.99	8.65E-04	3.12E-02	yes
positive regulation of smooth muscle cell proliferation (GO:0048661)	85	13	3.88	+	3.35	3.37E-04	1.51E-02	yes

regulation of fibroblast proliferation (GO:0048145)	89	13	4.07	+	3.2	4.99E-04	2.06E-02	yes
renal tubule development (GO:0061326)	91	13	4.16	+	3.13	6.03E-04	2.41E-02	yes
negative regulation of innate immune response (GO:0045824)	86	13	3.93	+	3.31	3.72E-04	1.62E-02	yes
epithelial to mesenchymal transition (GO:0001837)	81	12	3.7	+	3.24	7.25E-04	2.73E-02	yes
nephron tubule development (GO:0072080)	86	12	3.93	+	3.05	1.16E-03	3.82E-02	yes
neural crest cell development (GO:0014032)	80	12	3.66	+	3.28	6.57E-04	2.56E-02	yes
regulation of mitotic sister chromatid segregation (GO:0033047)	55	12	2.51	+	4.77	2.95E-05	2.06E-03	yes
detection of stimulus (GO:0051606)	686	12	31.35	-	0.38	1.48E-04	7.58E-03	yes
post-translational protein modification (GO:0043687)	65	12	2.97	+	4.04	1.21E-04	6.41E-03	yes
regulation of defense response to virus (GO:0050688)	85	12	3.88	+	3.09	1.06E-03	3.59E-02	yes
regulation of cardiac muscle contraction (GO:0055117)	77	12	3.52	+	3.41	4.85E-04	2.01E-02	yes
regulation of mitotic sister chromatid separation (GO:0010965)	59	12	2.7	+	4.45	5.37E-05	3.27E-03	yes
protein localization to chromosome (GO:0034502)	81	12	3.7	+	3.24	7.25E-04	2.73E-02	yes
positive regulation of response to cytokine stimulus (GO:0060760)	65	12	2.97	+	4.04	1.21E-04	6.39E-03	yes
outflow tract morphogenesis (GO:0003151)	77	12	3.52	+	3.41	4.85E-04	2.01E-02	yes
nephron epithelium morphogenesis (GO:0072088)	70	11	3.2	+	3.44	7.74E-04	2.86E-02	yes
nephron tubule morphogenesis (GO:0072078)	68	11	3.11	+	3.54	6.25E-04	2.45E-02	yes
positive regulation of DNA biosynthetic process (GO:2000573)	75	11	3.43	+	3.21	1.28E-03	4.18E-02	yes
nephron morphogenesis (GO:0072028)	73	11	3.34	+	3.3	1.05E-03	3.59E-02	yes
cell cycle G2/M phase transition (GO:0044839)	57	11	2.61	+	4.22	1.64E-04	8.12E-03	yes
regulation of interferon-beta production (GO:0032648)	60	11	2.74	+	4.01	2.43E-04	1.12E-02	yes
negative regulation of nuclear division (GO:0051784)	48	11	2.19	+	5.01	4.28E-05	2.75E-03	yes
regulation of morphogenesis of an epithelium (GO:1905330)	65	11	2.97	+	3.7	4.46E-04	1.89E-02	yes
positive regulation of epithelial cell differentiation (GO:0030858)	61	11	2.79	+	3.95	2.76E-04	1.25E-02	yes
renal tubule morphogenesis (GO:0061333)	72	11	3.29	+	3.34	9.52E-04	3.33E-02	yes
response to type I interferon (GO:0034340)	54	11	2.47	+	4.46	1.08E-04	5.80E-03	yes
regulation of protein maturation (GO:1903317)	73	11	3.34	+	3.3	1.05E-03	3.58E-02	yes
negative regulation of cell cycle G2/M phase transition (GO:1902750)	65	10	2.97	+	3.37	1.53E-03	4.86E-02	yes
interferon-mediated signaling pathway (GO:0140888)	59	10	2.7	+	3.71	7.93E-04	2.91E-02	yes
positive regulation of cytokine-mediated signaling pathway (GO:0001961)	58	10	2.65	+	3.77	7.05E-04	2.69E-02	yes
mesonephric tubule morphogenesis (GO:0072171)	58	10	2.65	+	3.77	7.05E-04	2.68E-02	yes
negative regulation of chromosome segregation (GO:0051985)	36	10	1.65	+	6.08	2.36E-05	1.69E-03	yes
positive regulation of interferon-beta production (GO:0032728)	42	10	1.92	+	5.21	7.21E-05	4.11E-03	yes

negative regulation of metaphase/anaphase transition of cell cycle (GO:1902100)	35	10	1.6	+	6.25	1.92E-05	1.45E-03	yes
negative regulation of chromosome separation (GO:1905819)	35	10	1.6	+	6.25	1.92E-05	1.45E-03	yes
regulation of kidney development (GO:0090183)	36	10	1.65	+	6.08	2.36E-05	1.68E-03	yes
spindle checkpoint signaling (GO:0031577)	31	10	1.42	+	7.06	7.90E-06	7.21E-04	yes
regulation of defense response to virus by host (GO:0050691)	47	10	2.15	+	4.66	1.62E-04	8.03E-03	yes
positive regulation of fibroblast proliferation (GO:0048146)	54	10	2.47	+	4.05	4.30E-04	1.82E-02	yes
G2/M transition of mitotic cell cycle (GO:0000086)	52	10	2.38	+	4.21	3.30E-04	1.48E-02	yes
negative regulation of G2/M transition of mitotic cell cycle (GO:0010972)	63	10	2.88	+	3.47	1.24E-03	4.06E-02	yes
ureteric bud morphogenesis (GO:0060675)	57	10	2.61	+	3.84	6.26E-04	2.45E-02	yes
negative regulation of mitotic sister chromatid separation (GO:2000816)	33	9	1.51	+	5.97	6.79E-05	3.96E-03	yes
mitotic spindle assembly checkpoint signaling (GO:0007094)	30	9	1.37	+	6.56	3.63E-05	2.41E-03	yes
cellular response to type I interferon (GO:0071357)	46	9	2.1	+	4.28	5.83E-04	2.34E-02	yes
negative regulation of mitotic sister chromatid segregation (GO:0033048)	33	9	1.51	+	5.97	6.79E-05	3.94E-03	yes
negative regulation of sister chromatid segregation (GO:0033046)	33	9	1.51	+	5.97	6.79E-05	3.93E-03	yes
mitotic spindle checkpoint signaling (GO:0071174)	30	9	1.37	+	6.56	3.63E-05	2.40E-03	yes
spindle assembly checkpoint signaling (GO:0071173)	30	9	1.37	+	6.56	3.63E-05	2.39E-03	yes
positive regulation of morphogenesis of an epithelium (GO:1905332)	37	9	1.69	+	5.32	1.44E-04	7.39E-03	yes
response to interferon-beta (GO:0035456)	35	9	1.6	+	5.63	9.99E-05	5.41E-03	yes
regulation of morphogenesis of a branching structure (GO:0060688)	52	9	2.38	+	3.79	1.26E-03	4.11E-02	yes
cytosolic pattern recognition receptor signaling pathway (GO:0002753)	50	9	2.29	+	3.94	9.86E-04	3.40E-02	yes
negative regulation of mitotic metaphase/anaphase transition (GO:0045841)	33	9	1.51	+	5.97	6.79E-05	3.91E-03	yes
negative regulation of mitotic nuclear division (GO:0045839)	41	9	1.87	+	4.8	2.79E-04	1.27E-02	yes
positive regulation of miRNA transcription (GO:1902895)	51	9	2.33	+	3.86	1.12E-03	3.72E-02	yes
regulation of actin filament-based movement (GO:1903115)	40	8	1.83	+	4.38	1.03E-03	3.53E-02	yes
positive regulation of defense response to virus by host (GO:0002230)	35	8	1.6	+	5	4.80E-04	2.00E-02	yes
cell communication involved in cardiac conduction (GO:0086065)	43	8	1.97	+	4.07	1.55E-03	4.91E-02	yes
regulation of cardiac muscle cell contraction (GO:0086004)	34	8	1.55	+	5.15	4.06E-04	1.75E-02	yes
olfactory bulb development (GO:0021772)	33	7	1.51	+	4.64	1.57E-03	4.97E-02	yes
positive regulation of animal organ morphogenesis (GO:0110110)	32	7	1.46	+	4.79	1.35E-03	4.36E-02	yes
regulation of nephron tubule epithelial cell differentiation (GO:0072182)	13	7	0.59	+	11.78	1.40E-05	1.16E-03	yes

regulation of epithelial cell differentiation involved in kidney development (GO:2000696)	17	7	0.78	+	9.01	5.36E-05	3.28E-03	yes
positive regulation of chromosome segregation (GO:0051984)	29	7	1.33	+	5.28	8.18E-04	2.99E-02	yes
positive regulation of chromosome separation (GO:1905820)	31	7	1.42	+	4.94	1.15E-03	3.78E-02	yes
regulation of cardiac muscle cell action potential (GO:0098901)	30	7	1.37	+	5.11	9.72E-04	3.38E-02	yes
regulation of branching involved in ureteric bud morphogenesis (GO:0090189)	22	7	1.01	+	6.96	2.00E-04	9.59E-03	yes
response to gonadotropin (GO:0034698)	30	7	1.37	+	5.11	9.72E-04	3.38E-02	yes
cellular response to gonadotropin stimulus (GO:0071371)	20	6	0.91	+	6.56	7.44E-04	2.79E-02	yes
cellular response to exogenous dsRNA (GO:0071360)	18	6	0.82	+	7.29	4.69E-04	1.97E-02	yes
cellular response to dsRNA (GO:0071359)	23	6	1.05	+	5.71	1.37E-03	4.41E-02	yes
regulation of ventricular cardiac muscle cell action potential (GO:0098911)	12	6	0.55	+	10.94	8.10E-05	4.50E-03	yes
positive regulation of branching involved in ureteric bud morphogenesis (GO:0090190)	19	6	0.87	+	6.91	5.94E-04	2.38E-02	yes
positive regulation of cell migration involved in sprouting angiogenesis (GO:0090050)	21	6	0.96	+	6.25	9.22E-04	3.28E-02	yes
regulation of transcription involved in G1/S transition of mitotic cell cycle (GO:0000083)	22	6	1.01	+	5.97	1.13E-03	3.75E-02	yes
response to interferon-alpha (GO:0035455)	21	6	0.96	+	6.25	9.22E-04	3.27E-02	yes
dendrite self-avoidance (GO:0070593)	14	5	0.64	+	7.81	1.10E-03	3.69E-02	yes
regulation of metanephric nephron tubule epithelial cell differentiation (GO:0072307)	7	4	0.32	+	12.5	9.35E-04	3.31E-02	yes
ureter morphogenesis (GO:0072197)	7	4	0.32	+	12.5	9.35E-04	3.30E-02	yes
positive regulation of epithelial cell differentiation involved in kidney development (GO:2000698)	7	4	0.32	+	12.5	9.35E-04	3.29E-02	yes
regulation of microvillus assembly (GO:0032534)	6	4	0.27	+	14.59	6.16E-04	2.45E-02	yes
positive regulation of chromosome condensation (GO:1905821)	8	4	0.37	+	10.94	1.35E-03	4.38E-02	yes
interleukin-27-mediated signaling pathway (GO:0070106)	7	4	0.32	+	12.5	9.35E-04	3.29E-02	yes
bundle of His cell-Purkinje myocyte adhesion involved in cell communication (GO:0086073)	6	4	0.27	+	14.59	6.16E-04	2.44E-02	yes
cardiac muscle cell-cardiac muscle cell adhesion (GO:0086042)	7	4	0.32	+	12.5	9.35E-04	3.28E-02	yes
response to luteinizing hormone (GO:0034699)	8	4	0.37	+	10.94	1.35E-03	4.37E-02	yes
detection of stimulus involved in sensory perception (GO:0050906)	565	3	25.82	-	0.12	5.45E-08	9.78E-06	yes
detection of chemical stimulus (GO:0009593)	524	3	23.95	-	0.13	2.24E-07	3.30E-05	yes
sensory perception of chemical stimulus (GO:0007606)	549	2	25.09	-	0.08	9.45E-09	2.05E-06	yes
sensory perception of smell (GO:0007608)	470	1	21.48	-	0.05	3.20E-08	6.09E-06	yes
detection of chemical stimulus involved in sensory perception of smell (GO:0050911)	443	0	20.25	-	< 0.01	4.60E-09	1.04E-06	yes
detection of chemical stimulus involved in sensory perception (GO:0050907)	491	0	22.44	-	< 0.01	3.94E-10	1.43E-07	yes

positive regulation of fibroblast apoptotic process (GO:2000271)	10	7	0.57	+	12.31	1.55E-05	1.02E-03	no
positive regulation of chromosome condensation (GO:1905821)	8	5	0.46	+	10.99	4.00E-04	1.53E-02	no
double-strand break repair via break-induced replication (GO:0000727)	12	7	0.68	+	10.26	3.65E-05	2.09E-03	no
DNA strand elongation involved in DNA replication (GO:0006271)	15	8	0.85	+	9.38	1.63E-05	1.05E-03	no
mitotic DNA replication (GO:1902969)	14	7	0.8	+	8.79	7.66E-05	3.92E-03	no
DNA unwinding involved in DNA replication (GO:0006268)	21	10	1.19	+	8.37	3.08E-06	2.67E-04	no
regulation of fibroblast apoptotic process (GO:2000269)	19	9	1.08	+	8.33	1.00E-05	7.06E-04	no
positive regulation of branching involved in ureteric bud morphogenesis (GO:0090190)	19	9	1.08	+	8.33	1.00E-05	7.03E-04	no
positive regulation of DNA-directed DNA polymerase activity (GO:1900264)	13	6	0.74	+	8.11	3.55E-04	1.39E-02	no
regulation of DNA-directed DNA polymerase activity (GO:1900262)	13	6	0.74	+	8.11	3.55E-04	1.39E-02	no
regulation of chromosome condensation (GO:0060623)	11	5	0.63	+	7.99	1.19E-03	3.80E-02	no
DNA strand elongation (GO:0022616)	20	9	1.14	+	7.91	1.38E-05	9.25E-04	no
dendrite self-avoidance (GO:0070593)	14	6	0.8	+	7.54	4.84E-04	1.77E-02	no
nuclear DNA replication (GO:0033260)	26	11	1.48	+	7.44	2.45E-06	2.18E-04	no
positive regulation of transcription from RNA polymerase II promoter in response to endoplasmic reticulum stress (GO:1990440)	12	5	0.68	+	7.33	1.61E-03	4.87E-02	no
skin morphogenesis (GO:0043589)	12	5	0.68	+	7.33	1.61E-03	4.86E-02	no
regulation of branching involved in ureteric bud morphogenesis (GO:0090189)	22	9	1.25	+	7.19	2.52E-05	1.51E-03	no
cell cycle DNA replication (GO:0044786)	27	11	1.54	+	7.16	3.28E-06	2.80E-04	no
positive regulation of chromosome separation (GO:1905820)	31	12	1.76	+	6.81	1.81E-06	1.66E-04	no
spindle checkpoint signaling (GO:0031577)	31	12	1.76	+	6.81	1.81E-06	1.65E-04	no
regulation of attachment of spindle microtubules to kinetochore (GO:0051988)	19	7	1.08	+	6.48	3.42E-04	1.36E-02	no
mitotic spindle assembly checkpoint signaling (GO:0007094)	30	11	1.71	+	6.45	7.42E-06	5.54E-04	no
mitotic spindle checkpoint signaling (GO:0071174)	30	11	1.71	+	6.45	7.42E-06	5.51E-04	no
spindle assembly checkpoint signaling (GO:0071173)	30	11	1.71	+	6.45	7.42E-06	5.49E-04	no
regulation of mammary gland epithelial cell proliferation (GO:0033599)	17	6	0.97	+	6.21	1.10E-03	3.57E-02	no
mitotic chromosome condensation (GO:0007076)	17	6	0.97	+	6.21	1.10E-03	3.57E-02	no
regulation of chemokine (C-X-C motif) ligand 2 production (GO:2000341)	17	6	0.97	+	6.21	1.10E-03	3.56E-02	no
positive regulation of chromosome segregation (GO:0051984)	29	10	1.65	+	6.06	2.98E-05	1.75E-03	no
negative regulation of metaphase/anaphase transition of cell cycle (GO:1902100)	35	12	1.99	+	6.03	5.04E-06	4.04E-04	no
negative regulation of chromosome separation (GO:1905819)	35	12	1.99	+	6.03	5.04E-06	4.02E-04	no
response to interferon-alpha (GO:0035455)	21	7	1.19	+	5.86	5.60E-04	1.99E-02	no
negative regulation of mitotic sister chromatid separation (GO:2000816)	33	11	1.88	+	5.86	1.55E-05	1.02E-03	no

negative regulation of chromosome segregation (GO:0051985)	36	12	2.05	+	5.86	6.40E-06	4.89E-04	no
negative regulation of mitotic sister chromatid segregation (GO:0033048)	33	11	1.88	+	5.86	1.55E-05	1.02E-03	no
negative regulation of sister chromatid segregation (GO:0033046)	33	11	1.88	+	5.86	1.55E-05	1.01E-03	no
negative regulation of mitotic metaphase/anaphase transition (GO:0045841)	33	11	1.88	+	5.86	1.55E-05	1.01E-03	no
positive regulation of morphogenesis of an epithelium (GO:1905332)	37	12	2.1	+	5.7	8.06E-06	5.91E-04	no
integrated stress response signaling (GO:0140467)	32	10	1.82	+	5.49	5.94E-05	3.18E-03	no
regulation of chromosome separation (GO:1905818)	74	23	4.21	+	5.46	1.28E-09	3.50E-07	no
positive regulation of interferon-beta production (GO:0032728)	42	13	2.39	+	5.44	5.20E-06	4.06E-04	no
regulation of kidney development (GO:0090183)	36	11	2.05	+	5.37	3.04E-05	1.76E-03	no
release of cytochrome c from mitochondria (GO:0001836)	23	7	1.31	+	5.35	8.77E-04	2.92E-02	no
negative regulation of viral genome replication (GO:0045071)	56	17	3.19	+	5.34	2.39E-07	3.06E-05	no
response to X-ray (GO:0010165)	30	9	1.71	+	5.27	1.80E-04	7.81E-03	no
DNA replication initiation (GO:0006270)	28	8	1.59	+	5.02	5.39E-04	1.93E-02	no
response to interferon-beta (GO:0035456)	35	10	1.99	+	5.02	1.11E-04	5.34E-03	no
regulation of mitotic sister chromatid separation (GO:0010965)	59	16	3.36	+	4.77	1.89E-06	1.71E-04	no
negative regulation of mitotic nuclear division (GO:0045839)	41	11	2.33	+	4.72	8.23E-05	4.13E-03	no
regulation of interferon-beta production (GO:0032648)	60	16	3.41	+	4.69	2.27E-06	2.04E-04	no
cellular response to glucose starvation (GO:0042149)	50	13	2.84	+	4.57	2.51E-05	1.51E-03	no
liver regeneration (GO:0097421)	31	8	1.76	+	4.54	9.49E-04	3.13E-02	no
xenobiotic transport (GO:0042908)	43	11	2.45	+	4.5	1.18E-04	5.61E-03	no
negative regulation of viral process (GO:0048525)	94	24	5.35	+	4.49	1.47E-08	3.07E-06	no
regulation of mitotic sister chromatid segregation (GO:0033047)	55	14	3.13	+	4.48	1.52E-05	1.01E-03	no
negative regulation of nuclear division (GO:0051784)	48	12	2.73	+	4.4	7.07E-05	3.68E-03	no
DNA duplex unwinding (GO:0032508)	76	19	4.32	+	4.4	6.06E-07	6.44E-05	no
regulation of transcription from RNA polymerase II promoter in response to stress (GO:0043618)	36	9	2.05	+	4.4	5.62E-04	1.98E-02	no
DNA geometric change (GO:0032392)	82	20	4.66	+	4.29	4.32E-07	4.96E-05	no
regulation of endoplasmic reticulum stress-induced intrinsic apoptotic signaling pathway (GO:1902235)	33	8	1.88	+	4.26	1.34E-03	4.17E-02	no
ribonucleotide catabolic process (GO:0009261)	50	12	2.84	+	4.22	9.88E-05	4.84E-03	no
DNA damage response, signal transduction by p53 class mediator (GO:0030330)	42	10	2.39	+	4.19	3.92E-04	1.50E-02	no
positive regulation of miRNA transcription (GO:1902895)	51	12	2.9	+	4.14	1.16E-04	5.53E-03	no
regulation of viral genome replication (GO:0045069)	86	20	4.89	+	4.09	8.23E-07	8.34E-05	no

purine ribonucleotide catabolic process (GO:0009154)	43	10	2.45	+	4.09	4.60E-04	1.71E-02	no
purine nucleotide catabolic process (GO:0006195)	52	12	2.96	+	4.06	1.36E-04	6.26E-03	no
purine-containing compound catabolic process (GO:0072523)	57	13	3.24	+	4.01	8.03E-05	4.09E-03	no
regulation of chromosome segregation (GO:0051983)	132	30	7.51	+	4	2.54E-09	6.40E-07	no
positive regulation of type I interferon production (GO:0032481)	71	16	4.04	+	3.96	1.42E-05	9.50E-04	no
response to type I interferon (GO:0034340)	54	12	3.07	+	3.91	1.85E-04	7.99E-03	no
regulation of erythrocyte differentiation (GO:0045646)	45	10	2.56	+	3.91	6.26E-04	2.17E-02	no
mitotic sister chromatid segregation (GO:0000070)	135	30	7.68	+	3.91	3.99E-09	9.43E-07	no
DNA conformation change (GO:0071103)	91	20	5.18	+	3.86	1.76E-06	1.63E-04	no
somatic diversification of immune receptors via germline recombination within a single locus (GO:0002562)	41	9	2.33	+	3.86	1.25E-03	3.94E-02	no
somatic cell DNA recombination (GO:0016444)	41	9	2.33	+	3.86	1.25E-03	3.93E-02	no
sister chromatid segregation (GO:0000819)	139	30	7.91	+	3.79	7.13E-09	1.61E-06	no
regulation of morphogenesis of an epithelium (GO:1905330)	65	14	3.7	+	3.79	7.38E-05	3.81E-03	no
regulation of DNA-templated transcription in response to stress (GO:0043620)	42	9	2.39	+	3.77	1.44E-03	4.46E-02	no
somatic diversification of immunoglobulins (GO:0016445)	42	9	2.39	+	3.77	1.44E-03	4.46E-02	no
regulation of metaphase/anaphase transition of cell cycle (GO:1902099)	94	20	5.35	+	3.74	2.70E-06	2.38E-04	no
somatic diversification of immune receptors (GO:0002200)	52	11	2.96	+	3.72	4.88E-04	1.78E-02	no
regulation of DNA-templated DNA replication (GO:0090329)	52	11	2.96	+	3.72	4.88E-04	1.77E-02	no
mitotic nuclear division (GO:0140014)	161	34	9.16	+	3.71	1.17E-09	3.32E-07	no
positive regulation of miRNA metabolic process (GO:2000630)	57	12	3.24	+	3.7	2.85E-04	1.15E-02	no
regulation of mitotic metaphase/anaphase transition (GO:0030071)	91	19	5.18	+	3.67	6.04E-06	4.64E-04	no
regulation of extrinsic apoptotic signaling pathway via death domain receptors (GO:1902041)	48	10	2.73	+	3.66	9.65E-04	3.16E-02	no
regulation of sister chromatid segregation (GO:0033045)	106	22	6.03	+	3.65	1.25E-06	1.19E-04	no
skeletal muscle cell differentiation (GO:0035914)	54	11	3.07	+	3.58	6.43E-04	2.22E-02	no
regulation of mitotic nuclear division (GO:0007088)	118	24	6.71	+	3.58	5.70E-07	6.18E-05	no
regulation of viral life cycle (GO:1903900)	143	29	8.13	+	3.57	4.15E-08	7.35E-06	no
regulation of miRNA transcription (GO:1902893)	70	14	3.98	+	3.52	1.47E-04	6.57E-03	no
DNA-templated DNA replication (GO:0006261)	135	27	7.68	+	3.52	1.54E-07	2.10E-05	no
protein kinase B signaling (GO:0043491)	51	10	2.9	+	3.45	1.44E-03	4.46E-02	no
regulation of viral-induced cytoplasmic pattern recognition receptor signaling pathway (GO:0039531)	57	11	3.24	+	3.39	9.51E-04	3.13E-02	no
regulation of viral process (GO:0050792)	166	32	9.44	+	3.39	2.39E-08	4.67E-06	no

G2/M transition of mitotic cell cycle (GO:0000086)	52	10	2.96	+	3.38	1.64E-03	4.94E-02	no
regulation of morphogenesis of a branching structure (GO:0060688)	52	10	2.96	+	3.38	1.64E-03	4.93E-02	no
animal organ regeneration (GO:0031100)	68	13	3.87	+	3.36	3.67E-04	1.43E-02	no
DNA replication (GO:0006260)	199	38	11.32	+	3.36	1.50E-09	3.90E-07	no
negative regulation of chromosome organization (GO:2001251)	79	15	4.49	+	3.34	1.43E-04	6.46E-03	no
tumor necrosis factor-mediated signaling pathway (GO:0033209)	58	11	3.3	+	3.33	1.08E-03	3.52E-02	no
signal transduction by p53 class mediator (GO:0072331)	90	17	5.12	+	3.32	5.58E-05	3.00E-03	no
double-strand break repair via homologous recombination (GO:0000724)	117	22	6.65	+	3.31	5.10E-06	4.02E-04	no
nuclear chromosome segregation (GO:0098813)	235	44	13.37	+	3.29	1.34E-10	4.75E-08	no
positive regulation of mitotic cell cycle phase transition (GO:1901992)	91	17	5.18	+	3.28	6.29E-05	3.33E-03	no
positive regulation of DNA biosynthetic process (GO:2000573)	75	14	4.27	+	3.28	2.75E-04	1.11E-02	no
microtubule cytoskeleton organization involved in mitosis (GO:1902850)	130	24	7.39	+	3.25	2.55E-06	2.26E-04	no
positive regulation of cell cycle phase transition (GO:1901989)	114	21	6.48	+	3.24	1.09E-05	7.58E-04	no
response to osmotic stress (GO:0006970)	76	14	4.32	+	3.24	3.09E-04	1.24E-02	no
circadian regulation of gene expression (GO:0032922)	71	13	4.04	+	3.22	5.27E-04	1.90E-02	no
recombinational repair (GO:0000725)	121	22	6.88	+	3.2	8.16E-06	5.95E-04	no
nucleotide catabolic process (GO:0009166)	77	14	4.38	+	3.2	3.48E-04	1.37E-02	no
protein K48-linked ubiquitination (GO:0070936)	72	13	4.1	+	3.17	5.92E-04	2.09E-02	no
mitotic spindle organization (GO:0007052)	95	17	5.4	+	3.15	1.00E-04	4.89E-03	no
regulation of response to endoplasmic reticulum stress (GO:1905897)	84	15	4.78	+	3.14	2.57E-04	1.06E-02	no
response to unfolded protein (GO:0006986)	119	21	6.77	+	3.1	1.93E-05	1.22E-03	no
telomere maintenance (GO:0000723)	97	17	5.52	+	3.08	1.25E-04	5.88E-03	no
nucleoside phosphate catabolic process (GO:1901292)	86	15	4.89	+	3.07	3.22E-04	1.28E-02	no
defense response to virus (GO:0051607)	265	46	15.07	+	3.05	4.54E-10	1.39E-07	no
positive regulation of mitotic cell cycle (GO:0045931)	121	21	6.88	+	3.05	2.40E-05	1.47E-03	no
DNA biosynthetic process (GO:0071897)	98	17	5.57	+	3.05	1.40E-04	6.38E-03	no
intrinsic apoptotic signaling pathway in response to DNA damage (GO:0008630)	75	13	4.27	+	3.05	8.30E-04	2.77E-02	no
defense response to symbiont (GO:0140546)	266	46	15.13	+	3.04	5.05E-10	1.52E-07	no
epithelial to mesenchymal transition (GO:0001837)	81	14	4.61	+	3.04	5.45E-04	1.95E-02	no
regulation of miRNA metabolic process (GO:2000628)	81	14	4.61	+	3.04	5.45E-04	1.95E-02	no
regulation of nuclear division (GO:0051783)	146	25	8.3	+	3.01	5.17E-06	4.05E-04	no
chromosome organization (GO:0051276)	456	78	25.94	+	3.01	7.27E-16	9.46E-13	no
hematopoietic progenitor cell differentiation (GO:0002244)	106	18	6.03	+	2.99	1.14E-04	5.46E-03	no
telomere organization (GO:0032200)	107	18	6.09	+	2.96	1.27E-04	5.94E-03	no

regulation of type I interferon production (GO:0032479)	119	20	6.77	+	2.96	5.55E-05	3.00E-03	no
positive regulation of axonogenesis (GO:0050772)	78	13	4.44	+	2.93	1.14E-03	3.67E-02	no
double-strand break repair (GO:0006302)	210	35	11.94	+	2.93	1.30E-07	1.80E-05	no
positive regulation of smooth muscle cell proliferation (GO:0048661)	85	14	4.83	+	2.9	8.30E-04	2.77E-02	no
negative regulation of epithelial cell proliferation (GO:0050680)	140	23	7.96	+	2.89	2.19E-05	1.34E-03	no
chromosome segregation (GO:0007059)	325	53	18.48	+	2.87	1.63E-10	5.65E-08	no
positive regulation of protein polymerization (GO:0032273)	92	15	5.23	+	2.87	6.03E-04	2.12E-02	no
regulation of ERBB signaling pathway (GO:1901184)	80	13	4.55	+	2.86	1.40E-03	4.34E-02	no
myeloid leukocyte differentiation (GO:0002573)	150	24	8.53	+	2.81	2.15E-05	1.33E-03	no
positive regulation of proteasomal ubiquitin-dependent protein catabolic process (GO:0032436)	94	15	5.35	+	2.81	7.35E-04	2.49E-02	no
positive regulation of protein localization to nucleus (GO:1900182)	94	15	5.35	+	2.81	7.35E-04	2.49E-02	no
positive regulation of protein-containing complex assembly (GO:0031334)	201	32	11.43	+	2.8	1.08E-06	1.06E-04	no
ureteric bud development (GO:0001657)	88	14	5.01	+	2.8	1.12E-03	3.61E-02	no
mitotic cell cycle process (GO:1903047)	522	83	29.69	+	2.8	5.14E-15	3.82E-12	no
response to virus (GO:0009615)	367	58	20.87	+	2.78	6.62E-11	2.40E-08	no
negative regulation of response to biotic stimulus (GO:0002832)	133	21	7.56	+	2.78	8.12E-05	4.10E-03	no
mitotic cell cycle checkpoint signaling (GO:0007093)	127	20	7.22	+	2.77	1.23E-04	5.77E-03	no
mesonephric tubule development (GO:0072164)	89	14	5.06	+	2.77	1.23E-03	3.92E-02	no
mesonephric epithelium development (GO:0072163)	89	14	5.06	+	2.77	1.23E-03	3.91E-02	no
kidney epithelium development (GO:0072073)	140	22	7.96	+	2.76	5.92E-05	3.17E-03	no
response to topologically incorrect protein (GO:0035966)	141	22	8.02	+	2.74	6.50E-05	3.41E-03	no
response to ionizing radiation (GO:0010212)	144	22	8.19	+	2.69	8.56E-05	4.28E-03	no
mitotic cell cycle phase transition (GO:0044772)	164	25	9.33	+	2.68	3.00E-05	1.75E-03	no
positive regulation of ubiquitin-dependent protein catabolic process (GO:2000060)	112	17	6.37	+	2.67	5.61E-04	1.98E-02	no
positive regulation of cell cycle process (GO:0090068)	253	38	14.39	+	2.64	4.95E-07	5.48E-05	no
cell division (GO:0051301)	521	78	29.63	+	2.63	4.71E-13	2.37E-10	no
negative regulation of mitotic cell cycle phase transition (GO:1901991)	167	25	9.5	+	2.63	6.25E-05	3.32E-03	no
cell cycle checkpoint signaling (GO:0000075)	161	24	9.16	+	2.62	9.33E-05	4.59E-03	no
liver development (GO:0001889)	135	20	7.68	+	2.6	2.54E-04	1.05E-02	no
mitotic cell cycle (GO:0000278)	616	91	35.04	+	2.6	1.25E-14	7.78E-12	no
positive regulation of epithelial cell migration (GO:0010634)	151	22	8.59	+	2.56	2.25E-04	9.39E-03	no
viral entry into host cell (GO:0046718)	110	16	6.26	+	2.56	1.20E-03	3.85E-02	no
intrinsic apoptotic signaling pathway (GO:0097193)	165	24	9.38	+	2.56	1.11E-04	5.32E-03	no

hepaticobiliary system development (GO:0061008)	138	20	7.85	+	2.55	4.83E-04	1.77E-02	no
regulation of epithelial cell migration (GO:0010632)	229	33	13.02	+	2.53	5.53E-06	4.29E-04	no
negative regulation of supramolecular fiber organization (GO:1902904)	160	23	9.1	+	2.53	1.74E-04	7.62E-03	no
regulation of mitotic cell cycle phase transition (GO:1901990)	334	48	19	+	2.53	5.49E-08	9.31E-06	no
cell cycle phase transition (GO:0044770)	175	25	9.95	+	2.51	9.21E-05	4.56E-03	no
nuclear division (GO:0000280)	308	44	17.52	+	2.51	2.42E-07	3.07E-05	no
regulation of endothelial cell migration (GO:0010594)	169	24	9.61	+	2.5	1.38E-04	6.31E-03	no
response to transforming growth factor beta (GO:0071559)	162	23	9.21	+	2.5	1.94E-04	8.26E-03	no
cellular response to transforming growth factor beta stimulus (GO:0071560)	155	22	8.82	+	2.5	2.74E-04	1.11E-02	no
spindle organization (GO:0007051)	163	23	9.27	+	2.48	2.05E-04	8.66E-03	no
positive regulation of cell cycle (GO:0045787)	346	48	19.68	+	2.44	1.16E-07	1.71E-05	no
cellular response to starvation (GO:0009267)	173	24	9.84	+	2.44	1.77E-04	7.72E-03	no
tube formation (GO:0035148)	152	21	8.65	+	2.43	4.85E-04	1.77E-02	no
organelle fission (GO:0048285)	334	46	19	+	2.42	2.57E-07	3.23E-05	no
organophosphate catabolic process (GO:0046434)	138	19	7.85	+	2.42	9.63E-04	3.16E-02	no
DNA recombination (GO:0006310)	248	34	14.11	+	2.41	1.22E-05	8.25E-04	no
establishment of cell polarity (GO:0030010)	133	18	7.56	+	2.38	1.52E-03	4.65E-02	no
regulation of myeloid cell differentiation (GO:0045637)	200	27	11.38	+	2.37	1.42E-04	6.46E-03	no
cellular response to tumor necrosis factor (GO:0071356)	180	24	10.24	+	2.34	4.22E-04	1.59E-02	no
epithelial cell proliferation (GO:0050673)	174	23	9.9	+	2.32	6.18E-04	2.15E-02	no
regulation of chromosome organization (GO:0033044)	251	33	14.28	+	2.31	2.98E-05	1.74E-03	no
cellular response to radiation (GO:0071478)	183	24	10.41	+	2.31	4.66E-04	1.72E-02	no
regulation of cell cycle phase transition (GO:1901987)	435	57	24.74	+	2.3	4.74E-08	8.13E-06	no
cell cycle process (GO:0022402)	878	115	49.94	+	2.3	5.64E-15	4.00E-12	no
negative regulation of cell migration (GO:0030336)	286	37	16.27	+	2.27	1.56E-05	1.01E-03	no
negative regulation of organelle organization (GO:0010639)	341	44	19.39	+	2.27	3.09E-06	2.66E-04	no
mesenchymal cell differentiation (GO:0048762)	171	22	9.73	+	2.26	1.00E-03	3.27E-02	no
DNA metabolic process (GO:0006259)	789	101	44.87	+	2.25	1.01E-12	4.77E-10	no
immune system development (GO:0002520)	165	21	9.38	+	2.24	1.46E-03	4.50E-02	no
DNA repair (GO:0006281)	511	65	29.06	+	2.24	1.73E-08	3.55E-06	no
negative regulation of cell cycle phase transition (GO:1901988)	229	29	13.02	+	2.23	2.21E-04	9.26E-03	no
regulation of epithelial cell proliferation (GO:0050678)	372	47	21.16	+	2.22	1.81E-06	1.67E-04	no
negative regulation of cell motility (GO:2000146)	301	38	17.12	+	2.22	2.49E-05	1.51E-03	no
response to tumor necrosis factor (GO:0034612)	206	26	11.72	+	2.22	3.69E-04	1.43E-02	no
negative regulation of mitotic cell cycle (GO:0045930)	214	27	12.17	+	2.22	4.26E-04	1.60E-02	no

positive regulation of innate immune response (GO:0045089)	254	32	14.45	+	2.22	9.37E-05	4.60E-03	no
regulation of protein ubiquitination (GO:0031396)	215	27	12.23	+	2.21	4.36E-04	1.63E-02	no
cellular response to nutrient levels (GO:0031669)	231	29	13.14	+	2.21	2.35E-04	9.73E-03	no
regulation of mitotic cell cycle (GO:0007346)	495	62	28.15	+	2.2	5.78E-08	9.70E-06	no
regulation of small GTPase mediated signal transduction (GO:0051056)	304	38	17.29	+	2.2	2.73E-05	1.62E-03	no
response to endoplasmic reticulum stress (GO:0034976)	224	28	12.74	+	2.2	3.29E-04	1.31E-02	no
negative regulation of growth (GO:0045926)	250	31	14.22	+	2.18	1.49E-04	6.62E-03	no
response to starvation (GO:0042594)	212	26	12.06	+	2.16	6.86E-04	2.35E-02	no
cellular response to extracellular stimulus (GO:0031668)	261	32	14.84	+	2.16	1.97E-04	8.36E-03	no
kidney development (GO:0001822)	303	37	17.23	+	2.15	4.77E-05	2.62E-03	no
regulation of protein polymerization (GO:0032271)	197	24	11.2	+	2.14	1.30E-03	4.07E-02	no
response to decreased oxygen levels (GO:0036293)	288	35	16.38	+	2.14	8.75E-05	4.36E-03	no
renal system development (GO:0072001)	313	38	17.8	+	2.13	3.97E-05	2.25E-03	no
cellular response to external stimulus (GO:0071496)	330	40	18.77	+	2.13	3.38E-05	1.95E-03	no
regulation of cell cycle process (GO:0010564)	726	88	41.29	+	2.13	3.60E-10	1.12E-07	no
positive regulation of neurogenesis (GO:0050769)	240	29	13.65	+	2.12	3.44E-04	1.36E-02	no
DNA damage response (GO:0006974)	765	92	43.51	+	2.11	2.08E-10	6.77E-08	no
regulation of innate immune response (GO:0045088)	385	46	21.9	+	2.1	1.13E-05	7.77E-04	no
myeloid cell differentiation (GO:0030099)	285	34	16.21	+	2.1	1.42E-04	6.47E-03	no
regulation of response to biotic stimulus (GO:0002831)	478	57	27.19	+	2.1	8.56E-07	8.62E-05	no
lipid transport (GO:0006869)	345	41	19.62	+	2.09	3.24E-05	1.88E-03	no
regulation of cell cycle (GO:0051726)	1120	133	63.7	+	2.09	2.90E-14	1.62E-11	no
regulation of protein-containing complex assembly (GO:0043254)	422	50	24	+	2.08	6.46E-06	4.89E-04	no
negative regulation of cell cycle (GO:0045786)	363	43	20.65	+	2.08	2.91E-05	1.72E-03	no
regulation of cysteine-type endopeptidase activity (GO:2000116)	228	27	12.97	+	2.08	7.18E-04	2.45E-02	no
negative regulation of locomotion (GO:0040013)	338	40	19.22	+	2.08	4.45E-05	2.46E-03	no
establishment or maintenance of cell polarity (GO:0007163)	203	24	11.55	+	2.08	1.58E-03	4.83E-02	no
biological process involved in symbiotic interaction (GO:0044403)	271	32	15.41	+	2.08	2.69E-04	1.09E-02	no
positive regulation of DNA metabolic process (GO:0051054)	305	36	17.35	+	2.08	1.35E-04	6.22E-03	no
cellular response to lipid (GO:0071396)	527	62	29.97	+	2.07	4.14E-07	4.78E-05	no
gland development (GO:0048732)	425	50	24.17	+	2.07	6.93E-06	5.22E-04	no
response to hypoxia (GO:0001666)	274	32	15.58	+	2.05	3.06E-04	1.23E-02	no
positive regulation of response to biotic stimulus (GO:0002833)	283	33	16.1	+	2.05	3.46E-04	1.36E-02	no
negative regulation of cell cycle process (GO:0010948)	275	32	15.64	+	2.05	4.69E-04	1.73E-02	no

regulation of I-kappaB kinase/NF-kappaB signaling (GO:0043122)	259	30	14.73	+	2.04	5.50E-04	1.96E-02	no
cellular response to abiotic stimulus (GO:0071214)	329	38	18.71	+	2.03	1.48E-04	6.61E-03	no
cellular response to environmental stimulus (GO:0104004)	329	38	18.71	+	2.03	1.48E-04	6.59E-03	no
hemopoiesis (GO:0030097)	684	79	38.9	+	2.03	2.75E-08	5.17E-06	no
cellular response to chemical stress (GO:0062197)	286	33	16.27	+	2.03	3.71E-04	1.43E-02	no
homeostasis of number of cells (GO:0048872)	270	31	15.36	+	2.02	6.70E-04	2.30E-02	no
growth (GO:0040007)	419	48	23.83	+	2.01	1.69E-05	1.09E-03	no
developmental growth (GO:0048589)	419	48	23.83	+	2.01	1.69E-05	1.08E-03	no
cell cycle (GO:0007049)	1267	145	72.06	+	2.01	2.69E-14	1.56E-11	no
response to growth factor (GO:0070848)	509	58	28.95	+	2	2.79E-06	2.43E-04	no
cellular response to biotic stimulus (GO:0071216)	237	27	13.48	+	2	1.37E-03	4.25E-02	no
response to oxygen levels (GO:0070482)	316	36	17.97	+	2	2.84E-04	1.14E-02	no
regulation of cell morphogenesis (GO:0022604)	247	28	14.05	+	1.99	1.12E-03	3.61E-02	no
response to alcohol (GO:0097305)	256	29	14.56	+	1.99	1.27E-03	3.98E-02	no
regulation of neuron death (GO:1901214)	327	37	18.6	+	1.99	2.27E-04	9.45E-03	no
cellular response to growth factor stimulus (GO:0071363)	478	54	27.19	+	1.99	8.92E-06	6.38E-04	no
regulation of protein modification by small protein conjugation or removal (GO:1903320)	257	29	14.62	+	1.98	1.29E-03	4.04E-02	no
cell population proliferation (GO:0008283)	728	82	41.41	+	1.98	3.09E-08	5.61E-06	no
lipid localization (GO:0010876)	383	43	21.78	+	1.97	7.64E-05	3.92E-03	no
negative regulation of cytokine production (GO:0001818)	295	33	16.78	+	1.97	7.28E-04	2.48E-02	no
regulation of hemopoiesis (GO:1903706)	401	44	22.81	+	1.93	1.08E-04	5.25E-03	no
positive regulation of cell development (GO:0010720)	439	48	24.97	+	1.92	5.99E-05	3.19E-03	no
positive regulation of proteolysis (GO:0045862)	367	40	20.87	+	1.92	2.24E-04	9.38E-03	no
cellular response to stress (GO:0033554)	1610	175	91.57	+	1.91	3.39E-15	2.94E-12	no
positive regulation of cellular component biogenesis (GO:0044089)	508	55	28.89	+	1.9	2.49E-05	1.51E-03	no
response to lipid (GO:0033993)	851	92	48.4	+	1.9	3.27E-08	5.87E-06	no
positive regulation of defense response (GO:0031349)	389	42	22.12	+	1.9	2.08E-04	8.77E-03	no
positive regulation of nervous system development (GO:0051962)	289	31	16.44	+	1.89	1.59E-03	4.83E-02	no
negative regulation of cellular component organization (GO:0051129)	711	76	40.44	+	1.88	7.18E-07	7.37E-05	no
regulation of cellular response to stress (GO:0080135)	694	74	39.47	+	1.87	1.19E-06	1.14E-04	no
leukocyte differentiation (GO:0002521)	424	45	24.12	+	1.87	1.70E-04	7.48E-03	no
regulation of protein serine/threonine kinase activity (GO:0071900)	368	39	20.93	+	1.86	5.08E-04	1.83E-02	no
chordate embryonic development (GO:0043009)	662	70	37.65	+	1.86	3.57E-06	3.03E-04	no
apoptotic signaling pathway (GO:0097190)	313	33	17.8	+	1.85	1.57E-03	4.80E-02	no

regulation of apoptotic signaling pathway (GO:2001233)	371	39	21.1	+	1.85	5.49E-04	1.96E-02	no
regulation of DNA metabolic process (GO:0051052)	533	56	30.31	+	1.85	3.83E-05	2.18E-03	no
regulation of supramolecular fiber organization (GO:1902903)	381	40	21.67	+	1.85	6.24E-04	2.17E-02	no
positive regulation of cell projection organization (GO:0031346)	354	37	20.13	+	1.84	9.13E-04	3.02E-02	no
embryo development ending in birth or egg hatching (GO:0009792)	684	71	38.9	+	1.83	5.07E-06	4.02E-04	no
negative regulation of cell population proliferation (GO:0008285)	708	73	40.27	+	1.81	4.90E-06	3.94E-04	no
regulation of response to stress (GO:0080134)	1451	149	82.53	+	1.81	2.43E-11	9.48E-09	no
response to radiation (GO:0009314)	448	46	25.48	+	1.81	3.60E-04	1.40E-02	no
regulation of actin filament-based process (GO:0032970)	390	40	22.18	+	1.8	7.42E-04	2.51E-02	no
transcription by RNA polymerase II (GO:0006366)	361	37	20.53	+	1.8	1.50E-03	4.61E-02	no
response to nutrient levels (GO:0031667)	479	49	27.24	+	1.8	1.90E-04	8.16E-03	no
response to extracellular stimulus (GO:0009991)	509	52	28.95	+	1.8	1.32E-04	6.15E-03	no
positive regulation of transferase activity (GO:0051347)	524	53	29.8	+	1.78	1.59E-04	7.04E-03	no
regulation of anatomical structure morphogenesis (GO:0022603)	871	88	49.54	+	1.78	1.08E-06	1.06E-04	no
positive regulation of cellular component organization (GO:0051130)	1122	113	63.81	+	1.77	2.57E-08	4.89E-06	no
defense response to other organism (GO:0098542)	997	100	56.7	+	1.76	2.07E-07	2.74E-05	no
response to organic cyclic compound (GO:0014070)	871	87	49.54	+	1.76	1.60E-06	1.51E-04	no
regulation of intracellular signal transduction (GO:1902531)	1727	172	98.22	+	1.75	6.19E-12	2.61E-09	no
apoptotic process (GO:0006915)	1045	104	59.43	+	1.75	1.81E-07	2.43E-05	no
intracellular signal transduction (GO:0035556)	1520	151	86.45	+	1.75	1.84E-10	6.24E-08	no
regulation of defense response (GO:0031347)	748	74	42.54	+	1.74	1.30E-05	8.78E-04	no
positive regulation of programmed cell death (GO:0043068)	527	52	29.97	+	1.73	3.44E-04	1.36E-02	no
innate immune response (GO:0045087)	752	74	42.77	+	1.73	1.86E-05	1.18E-03	no
lymphocyte activation (GO:0046649)	478	47	27.19	+	1.73	7.82E-04	2.63E-02	no
tube development (GO:0035295)	897	88	51.02	+	1.72	3.11E-06	2.66E-04	no
regulation of cell development (GO:0060284)	827	81	47.04	+	1.72	7.90E-06	5.82E-04	no
response to cytokine (GO:0034097)	817	80	46.47	+	1.72	9.89E-06	7.02E-04	no
regulation of organelle organization (GO:0033043)	1176	115	66.89	+	1.72	7.51E-08	1.22E-05	no
protein localization to organelle (GO:0033365)	728	71	41.41	+	1.71	4.00E-05	2.25E-03	no
positive regulation of cell death (GO:0010942)	595	58	33.84	+	1.71	1.93E-04	8.25E-03	no
programmed cell death (GO:0012501)	1088	106	61.88	+	1.71	3.13E-07	3.85E-05	no
endocytosis (GO:0006897)	534	52	30.37	+	1.71	3.89E-04	1.49E-02	no
regulation of programmed cell death (GO:0043067)	1510	147	85.88	+	1.71	1.42E-09	3.75E-07	no

cellular response to organic cyclic compound (GO:0071407)	504	49	28.67	+	1.71	7.52E-04	2.53E-02	no
positive regulation of organelle organization (GO:0010638)	515	50	29.29	+	1.71	6.06E-04	2.12E-02	no
tube morphogenesis (GO:0035239)	681	66	38.73	+	1.7	9.26E-05	4.57E-03	no
negative regulation of cell death (GO:0060548)	1022	99	58.13	+	1.7	1.16E-06	1.12E-04	no
regulation of apoptotic process (GO:0042981)	1466	142	83.38	+	1.7	3.54E-09	8.51E-07	no
negative regulation of programmed cell death (GO:0043069)	919	89	52.27	+	1.7	4.06E-06	3.37E-04	no
positive regulation of phosphorus metabolic process (GO:0010562)	848	82	48.23	+	1.7	1.02E-05	7.16E-04	no
positive regulation of phosphate metabolic process (GO:0045937)	848	82	48.23	+	1.7	1.02E-05	7.13E-04	no
skeletal system development (GO:0001501)	518	50	29.46	+	1.7	6.37E-04	2.20E-02	no
positive regulation of apoptotic process (GO:0043065)	508	49	28.89	+	1.7	7.90E-04	2.64E-02	no
heart development (GO:0007507)	560	54	31.85	+	1.7	3.74E-04	1.44E-02	no
negative regulation of apoptotic process (GO:0043066)	892	86	50.73	+	1.7	8.29E-06	6.02E-04	no
tissue morphogenesis (GO:0048729)	571	55	32.48	+	1.69	4.09E-04	1.56E-02	no
cell death (GO:0008219)	1122	108	63.81	+	1.69	4.50E-07	5.05E-05	no
positive regulation of response to external stimulus (GO:0032103)	531	51	30.2	+	1.69	7.25E-04	2.47E-02	no
regulation of growth (GO:0040008)	625	60	35.55	+	1.69	1.99E-04	8.42E-03	no
regulation of cell migration (GO:0030334)	930	89	52.89	+	1.68	6.42E-06	4.89E-04	no
positive regulation of intracellular signal transduction (GO:1902533)	1005	96	57.16	+	1.68	2.71E-06	2.38E-04	no
positive regulation of protein modification process (GO:0031401)	954	91	54.26	+	1.68	5.90E-06	4.56E-04	no
regulation of cell death (GO:0010941)	1668	159	94.87	+	1.68	9.79E-10	2.83E-07	no
microtubule cytoskeleton organization (GO:0000226)	556	53	31.62	+	1.68	6.81E-04	2.34E-02	no
regulation of protein kinase activity (GO:0045859)	619	59	35.21	+	1.68	2.63E-04	1.07E-02	no
regulation of transferase activity (GO:0051338)	851	81	48.4	+	1.67	2.06E-05	1.29E-03	no
animal organ morphogenesis (GO:0009887)	989	94	56.25	+	1.67	4.42E-06	3.63E-04	no
cell migration (GO:0016477)	875	83	49.77	+	1.67	1.89E-05	1.20E-03	no
positive regulation of cell differentiation (GO:0045597)	854	81	48.57	+	1.67	2.17E-05	1.34E-03	no
leukocyte activation (GO:0045321)	601	57	34.18	+	1.67	4.11E-04	1.56E-02	no
response to stress (GO:0006950)	3407	323	193.77	+	1.67	4.73E-20	7.38E-16	no
regulation of phosphate metabolic process (GO:0019220)	1322	125	75.19	+	1.66	1.27E-07	1.80E-05	no
regulation of phosphorus metabolic process (GO:0051174)	1323	125	75.25	+	1.66	1.29E-07	1.79E-05	no
positive regulation of phosphorylation (GO:0042327)	774	73	44.02	+	1.66	6.78E-05	3.55E-03	no
locomotion (GO:0040011)	541	51	30.77	+	1.66	1.13E-03	3.63E-02	no
regulation of proteolysis (GO:0030162)	711	67	40.44	+	1.66	1.34E-04	6.23E-03	no
response to abiotic stimulus (GO:0009628)	1117	105	63.53	+	1.65	1.67E-06	1.56E-04	no
positive regulation of immune response (GO:0050778)	621	58	35.32	+	1.64	5.09E-04	1.83E-02	no

biological process involved in interspecies interaction between organisms (GO:0044419)	1542	144	87.7	+	1.64	2.17E-08	4.35E-06	no
negative regulation of signal transduction (GO:0009968)	1265	118	71.95	+	1.64	5.23E-07	5.71E-05	no
regulation of catalytic activity (GO:0050790)	1825	170	103.8	+	1.64	1.20E-09	3.33E-07	no
regulation of cell motility (GO:2000145)	989	92	56.25	+	1.64	1.19E-05	8.10E-04	no
cellular response to organic substance (GO:0071310)	1776	165	101.01	+	1.63	2.79E-09	6.92E-07	no
epithelium development (GO:0060429)	1098	102	62.45	+	1.63	4.85E-06	3.92E-04	no
positive regulation of protein metabolic process (GO:0051247)	1446	134	82.24	+	1.63	1.15E-07	1.71E-05	no
regulation of response to external stimulus (GO:0032101)	1058	98	60.17	+	1.63	8.60E-06	6.18E-04	no
regulation of cell adhesion (GO:0030155)	789	73	44.87	+	1.63	1.45E-04	6.55E-03	no
positive regulation of protein phosphorylation (GO:0001934)	714	66	40.61	+	1.63	2.59E-04	1.06E-02	no
negative regulation of transcription by RNA polymerase II (GO:0000122)	987	91	56.14	+	1.62	2.13E-05	1.33E-03	no
cellular response to oxygen-containing compound (GO:1901701)	1150	106	65.41	+	1.62	4.17E-06	3.44E-04	no
regulation of locomotion (GO:0040012)	1034	95	58.81	+	1.62	1.32E-05	8.91E-04	no
cellular response to cytokine stimulus (GO:0071345)	719	66	40.89	+	1.61	3.60E-04	1.40E-02	no
regulation of protein modification process (GO:0031399)	1471	135	83.66	+	1.61	1.95E-07	2.60E-05	no
regulation of phosphorylation (GO:0042325)	1177	108	66.94	+	1.61	3.89E-06	3.26E-04	no
response to other organism (GO:0051707)	1363	125	77.52	+	1.61	5.07E-07	5.57E-05	no
response to external biotic stimulus (GO:0043207)	1366	125	77.69	+	1.61	6.83E-07	7.06E-05	no
response to organic substance (GO:0010033)	2590	237	147.31	+	1.61	8.86E-13	4.32E-10	no
regulation of kinase activity (GO:0043549)	713	65	40.55	+	1.6	4.64E-04	1.72E-02	no
defense response (GO:0006952)	1417	129	80.59	+	1.6	5.71E-07	6.15E-05	no
regulation of signal transduction (GO:0009966)	2990	272	170.06	+	1.6	1.77E-14	1.06E-11	no
positive regulation of molecular function (GO:0044093)	1517	138	86.28	+	1.6	2.12E-07	2.79E-05	no
negative regulation of response to stimulus (GO:0048585)	1640	149	93.28	+	1.6	6.96E-08	1.14E-05	no
response to biotic stimulus (GO:0009607)	1411	127	80.25	+	1.58	1.03E-06	1.01E-04	no
regulation of protein phosphorylation (GO:0001932)	1079	97	61.37	+	1.58	2.58E-05	1.54E-03	no
nucleobase-containing compound biosynthetic process (GO:0034654)	1035	93	58.87	+	1.58	4.33E-05	2.41E-03	no
positive regulation of signal transduction (GO:0009967)	1551	139	88.21	+	1.58	3.83E-07	4.47E-05	no
regulation of cellular component organization (GO:0051128)	2445	219	139.06	+	1.57	5.98E-11	2.22E-08	no
positive regulation of nitrogen compound metabolic process (GO:0051173)	3128	280	177.91	+	1.57	5.03E-14	2.71E-11	no
tissue development (GO:0009888)	1745	156	99.25	+	1.57	8.77E-08	1.34E-05	no
regulation of cell communication (GO:0010646)	3402	304	193.49	+	1.57	2.31E-15	2.40E-12	no
regulation of multicellular organismal development (GO:2000026)	1399	125	79.57	+	1.57	2.19E-06	1.97E-04	no
regulation of MAPK cascade (GO:0043408)	672	60	38.22	+	1.57	1.12E-03	3.61E-02	no

negative regulation of gene expression (GO:0010629)	941	84	53.52	+	1.57	1.19E-04	5.61E-03	no
positive regulation of RNA biosynthetic process (GO:1902680)	1714	153	97.48	+	1.57	1.25E-07	1.79E-05	no
circulatory system development (GO:0072359)	920	82	52.33	+	1.57	1.38E-04	6.34E-03	no
embryo development (GO:0009790)	1056	94	60.06	+	1.57	5.12E-05	2.78E-03	no
negative regulation of cell communication (GO:0010648)	1360	121	77.35	+	1.56	3.87E-06	3.27E-04	no
regulation of cellular component biogenesis (GO:0044087)	967	86	55	+	1.56	1.09E-04	5.29E-03	no
negative regulation of signaling (GO:0023057)	1361	121	77.41	+	1.56	3.91E-06	3.26E-04	no
regulation of cell population proliferation (GO:0042127)	1676	149	95.32	+	1.56	2.27E-07	2.95E-05	no
regulation of signaling (GO:0023051)	3393	301	192.98	+	1.56	7.97E-15	5.19E-12	no
heterocycle biosynthetic process (GO:0018130)	1106	98	62.9	+	1.56	4.21E-05	2.36E-03	no
regulation of cytokine production (GO:0001817)	779	69	44.31	+	1.56	6.04E-04	2.11E-02	no
positive regulation of macromolecule metabolic process (GO:0010604)	3524	312	200.43	+	1.56	2.88E-15	2.64E-12	no
positive regulation of cellular biosynthetic process (GO:0031328)	2014	178	114.55	+	1.55	1.47E-08	3.09E-06	no
positive regulation of catalytic activity (GO:0043085)	1110	98	63.13	+	1.55	4.40E-05	2.44E-03	no
positive regulation of biosynthetic process (GO:0009891)	2073	183	117.9	+	1.55	9.36E-09	2.00E-06	no
positive regulation of nucleobase-containing compound metabolic process (GO:0045935)	2044	180	116.25	+	1.55	1.76E-08	3.57E-06	no
response to nitrogen compound (GO:1901698)	1057	93	60.12	+	1.55	9.05E-05	4.50E-03	no
response to external stimulus (GO:0009605)	2410	212	137.07	+	1.55	6.77E-10	1.99E-07	no
positive regulation of macromolecule biosynthetic process (GO:0010557)	1934	170	110	+	1.55	4.72E-08	8.18E-06	no
positive regulation of nucleic acid-templated transcription (GO:1903508)	1707	150	97.09	+	1.55	3.80E-07	4.49E-05	no
positive regulation of DNA-templated transcription (GO:0045893)	1707	150	97.09	+	1.55	3.80E-07	4.46E-05	no
regulation of molecular function (GO:0065009)	2567	225	146	+	1.54	1.96E-10	6.52E-08	no
positive regulation of transcription by RNA polymerase II (GO:0045944)	1256	110	71.44	+	1.54	2.31E-05	1.41E-03	no
positive regulation of response to stimulus (GO:0048584)	2197	192	124.95	+	1.54	9.01E-09	1.95E-06	no
positive regulation of signaling (GO:0023056)	1728	151	98.28	+	1.54	4.46E-07	5.04E-05	no
positive regulation of cell communication (GO:0010647)	1729	151	98.34	+	1.54	4.52E-07	5.03E-05	no
organic cyclic compound biosynthetic process (GO:1901362)	1238	108	70.41	+	1.53	2.73E-05	1.62E-03	no
positive regulation of cellular metabolic process (GO:0031325)	2971	259	168.98	+	1.53	8.10E-12	3.33E-09	no
response to oxygen-containing compound (GO:1901700)	1679	146	95.49	+	1.53	9.65E-07	9.65E-05	no
regulation of hydrolase activity (GO:0051336)	966	84	54.94	+	1.53	2.55E-04	1.05E-02	no
aromatic compound biosynthetic process (GO:0019438)	1116	97	63.47	+	1.53	8.10E-05	4.12E-03	no
negative regulation of DNA-templated transcription (GO:0045892)	1315	114	74.79	+	1.52	2.03E-05	1.28E-03	no

cellular response to endogenous stimulus (GO:0071495)	1200	104	68.25	+	1.52	4.83E-05	2.65E-03	no
positive regulation of RNA metabolic process (GO:0051254)	1847	160	105.05	+	1.52	3.22E-07	3.93E-05	no
negative regulation of nucleic acid-templated transcription (GO:1903507)	1316	114	74.85	+	1.52	2.06E-05	1.29E-03	no
cytoskeleton organization (GO:0007010)	1247	108	70.92	+	1.52	3.77E-05	2.15E-03	no
organelle organization (GO:0006996)	3050	264	173.47	+	1.52	9.94E-12	3.98E-09	no
positive regulation of metabolic process (GO:0009893)	3834	331	218.06	+	1.52	6.54E-15	4.44E-12	no
regulation of response to stimulus (GO:0048583)	3954	341	224.88	+	1.52	2.83E-15	2.76E-12	no
negative regulation of RNA biosynthetic process (GO:1902679)	1330	114	75.64	+	1.51	3.74E-05	2.14E-03	no
cellular response to chemical stimulus (GO:0070887)	2405	206	136.78	+	1.51	8.64E-09	1.90E-06	no
regulation of cell differentiation (GO:0045595)	1556	133	88.5	+	1.5	8.38E-06	6.05E-04	no
response to organonitrogen compound (GO:0010243)	983	84	55.91	+	1.5	4.76E-04	1.75E-02	no
organonitrogen compound catabolic process (GO:1901565)	1113	95	63.3	+	1.5	1.77E-04	7.74E-03	no
regulation of protein metabolic process (GO:0051246)	2486	212	141.39	+	1.5	7.91E-09	1.76E-06	no
positive regulation of cell population proliferation (GO:0008284)	952	81	54.15	+	1.5	6.63E-04	2.28E-02	no
phosphorylation (GO:0016310)	883	75	50.22	+	1.49	1.21E-03	3.86E-02	no
nucleic acid metabolic process (GO:0090304)	2279	193	129.62	+	1.49	7.72E-08	1.22E-05	no
positive regulation of developmental process (GO:0051094)	1314	111	74.73	+	1.49	7.47E-05	3.85E-03	no
negative regulation of cellular process (GO:0048523)	4735	399	269.3	+	1.48	8.18E-17	1.82E-13	no
positive regulation of multicellular organismal process (GO:0051240)	1653	139	94.01	+	1.48	1.13E-05	7.77E-04	no
regulation of immune system process (GO:0002682)	1480	124	84.18	+	1.47	4.16E-05	2.34E-03	no
positive regulation of gene expression (GO:0010628)	1184	99	67.34	+	1.47	2.67E-04	1.09E-02	no
negative regulation of biological process (GO:0048519)	5336	446	303.49	+	1.47	8.07E-19	4.20E-15	no
response to endogenous stimulus (GO:0009719)	1462	122	83.15	+	1.47	4.95E-05	2.70E-03	no
negative regulation of macromolecule biosynthetic process (GO:0010558)	1547	128	87.99	+	1.45	4.70E-05	2.59E-03	no
macromolecule catabolic process (GO:0009057)	1004	83	57.1	+	1.45	1.45E-03	4.46E-02	no
regulation of developmental process (GO:0050793)	2460	202	139.91	+	1.44	2.59E-07	3.24E-05	no
positive regulation of cellular process (GO:0048522)	5523	452	314.12	+	1.44	1.98E-17	6.17E-14	no
negative regulation of macromolecule metabolic process (GO:0010605)	2746	224	156.18	+	1.43	8.40E-08	1.30E-05	no
negative regulation of nucleobase-containing compound metabolic process (GO:0045934)	1560	127	88.73	+	1.43	1.01E-04	4.93E-03	no
nucleobase-containing compound metabolic process (GO:0006139)	2794	227	158.91	+	1.43	8.27E-08	1.29E-05	no
negative regulation of biosynthetic process (GO:0009890)	1638	133	93.16	+	1.43	7.32E-05	3.80E-03	no

negative regulation of RNA metabolic process (GO:0051253)	1441	117	81.96	+	1.43	2.31E-04	9.59E-03	no
positive regulation of biological process (GO:0048518)	6234	503	354.56	+	1.42	6.33E-19	4.94E-15	no
negative regulation of cellular biosynthetic process (GO:0031327)	1589	128	90.37	+	1.42	1.45E-04	6.53E-03	no
phosphate-containing compound metabolic process (GO:0006796)	1815	146	103.23	+	1.41	5.08E-05	2.76E-03	no
heterocycle metabolic process (GO:0046483)	2969	238	168.86	+	1.41	1.07E-07	1.62E-05	no
phosphorus metabolic process (GO:0006793)	1841	147	104.71	+	1.4	7.04E-05	3.68E-03	no
protein-containing complex assembly (GO:0065003)	1267	101	72.06	+	1.4	1.25E-03	3.94E-02	no
regulation of primary metabolic process (GO:0080090)	5797	462	329.7	+	1.4	7.07E-16	1.00E-12	no
regulation of nitrogen compound metabolic process (GO:0051171)	5624	448	319.87	+	1.4	3.43E-15	2.68E-12	no
establishment of protein localization (GO:0045184)	1284	102	73.03	+	1.4	1.33E-03	4.16E-02	no
macromolecule localization (GO:0033036)	2368	188	134.68	+	1.4	7.03E-06	5.27E-04	no
protein-containing complex organization (GO:0043933)	1412	112	80.31	+	1.39	7.65E-04	2.57E-02	no
animal organ development (GO:0048513)	2925	232	166.36	+	1.39	3.57E-07	4.26E-05	no
regulation of multicellular organismal process (GO:0051239)	2980	235	169.49	+	1.39	4.40E-07	5.01E-05	no
negative regulation of nitrogen compound metabolic process (GO:0051172)	2375	187	135.08	+	1.38	1.12E-05	7.71E-04	no
organic cyclic compound metabolic process (GO:1901360)	3255	256	185.13	+	1.38	1.29E-07	1.81E-05	no
negative regulation of metabolic process (GO:0009892)	2976	234	169.26	+	1.38	6.65E-07	6.92E-05	no
regulation of macromolecule metabolic process (GO:0060255)	6162	484	350.46	+	1.38	9.95E-16	1.19E-12	no
anatomical structure morphogenesis (GO:0009653)	2233	175	127	+	1.38	2.96E-05	1.74E-03	no
cellular component organization (GO:0016043)	5497	430	312.64	+	1.38	3.28E-13	1.70E-10	no
cellular nitrogen compound biosynthetic process (GO:0044271)	1627	127	92.54	+	1.37	6.02E-04	2.12E-02	no
cellular macromolecule localization (GO:0070727)	1935	151	110.05	+	1.37	1.53E-04	6.79E-03	no
regulation of metabolic process (GO:0019222)	6681	521	379.98	+	1.37	7.59E-17	1.97E-13	no
cellular aromatic compound metabolic process (GO:0006725)	3014	235	171.42	+	1.37	1.17E-06	1.12E-04	no
protein localization (GO:0008104)	1929	150	109.71	+	1.37	1.84E-04	7.99E-03	no
regulation of cellular metabolic process (GO:0031323)	5447	423	309.8	+	1.37	2.02E-12	8.76E-10	no
regulation of RNA biosynthetic process (GO:2001141)	3431	266	195.14	+	1.36	2.29E-07	2.96E-05	no
response to chemical (GO:0042221)	3899	302	221.76	+	1.36	2.50E-08	4.81E-06	no
cell development (GO:0048468)	2169	168	123.36	+	1.36	8.17E-05	4.11E-03	no
cellular component organization or biogenesis (GO:0071840)	5700	440	324.19	+	1.36	1.22E-12	5.59E-10	no
regulation of nucleobase-containing compound metabolic process (GO:0019219)	4016	309	228.41	+	1.35	2.87E-08	5.34E-06	no
organic substance transport (GO:0071702)	2210	170	125.69	+	1.35	1.13E-04	5.43E-03	no

regulation of DNA-templated transcription (GO:0006355)	3406	262	193.72	+	1.35	6.06E-07	6.48E-05	no
regulation of localization (GO:0032879)	2133	164	121.31	+	1.35	1.34E-04	6.21E-03	no
regulation of nucleic acid-templated transcription (GO:1903506)	3408	262	193.83	+	1.35	6.11E-07	6.44E-05	no
system development (GO:0048731)	3553	273	202.08	+	1.35	3.32E-07	4.01E-05	no
regulation of macromolecule biosynthetic process (GO:0010556)	3917	299	222.78	+	1.34	1.18E-07	1.72E-05	no
negative regulation of cellular metabolic process (GO:0031324)	2214	169	125.92	+	1.34	1.69E-04	7.46E-03	no
regulation of transcription by RNA polymerase II (GO:0006357)	2418	184	137.52	+	1.34	8.11E-05	4.11E-03	no
regulation of biosynthetic process (GO:0009889)	4135	314	235.18	+	1.34	7.55E-08	1.21E-05	no
immune system process (GO:0002376)	2256	171	128.31	+	1.33	1.93E-04	8.26E-03	no
regulation of cellular biosynthetic process (GO:0031326)	4026	305	228.98	+	1.33	1.59E-07	2.15E-05	no
cellular nitrogen compound metabolic process (GO:0034641)	3344	252	190.19	+	1.32	4.66E-06	3.79E-04	no
regulation of RNA metabolic process (GO:0051252)	3727	280	211.97	+	1.32	1.46E-06	1.38E-04	no
cellular component assembly (GO:0022607)	2413	181	137.24	+	1.32	2.03E-04	8.59E-03	no
multicellular organism development (GO:0007275)	3969	296	225.74	+	1.31	1.01E-06	1.00E-04	no
regulation of gene expression (GO:0010468)	4847	361	275.67	+	1.31	3.04E-08	5.58E-06	no
cellular localization (GO:0051641)	2701	201	153.62	+	1.31	1.19E-04	5.61E-03	no
protein modification process (GO:0036211)	2573	189	146.34	+	1.29	4.22E-04	1.59E-02	no
cellular biosynthetic process (GO:0044249)	2221	163	126.32	+	1.29	1.25E-03	3.94E-02	no
cellular component biogenesis (GO:0044085)	2648	194	150.61	+	1.29	4.19E-04	1.58E-02	no
cellular metabolic process (GO:0044237)	5808	425	330.33	+	1.29	5.63E-09	1.31E-06	no
anatomical structure development (GO:0048856)	5193	379	295.35	+	1.28	1.09E-07	1.63E-05	no
macromolecule modification (GO:0043412)	2796	203	159.02	+	1.28	4.10E-04	1.56E-02	no
cellular response to stimulus (GO:0051716)	6443	463	366.45	+	1.26	7.06E-09	1.62E-06	no
localization (GO:0051179)	4498	322	255.82	+	1.26	9.86E-06	7.03E-04	no
macromolecule metabolic process (GO:0043170)	5934	424	337.5	+	1.26	1.19E-07	1.72E-05	no
establishment of localization (GO:0051234)	3934	281	223.75	+	1.26	6.40E-05	3.38E-03	no
developmental process (GO:0032502)	5732	406	326.01	+	1.25	7.46E-07	7.61E-05	no
nitrogen compound metabolic process (GO:0006807)	6649	469	378.16	+	1.24	5.95E-08	9.88E-06	no
cell differentiation (GO:0030154)	3575	252	203.33	+	1.24	3.79E-04	1.46E-02	no
response to stimulus (GO:0050896)	8196	577	466.15	+	1.24	2.09E-10	6.64E-08	no
cellular developmental process (GO:0048869)	3599	253	204.69	+	1.24	4.53E-04	1.68E-02	no
regulation of cellular process (GO:0050794)	11006	769	625.97	+	1.23	1.83E-16	2.85E-13	no
primary metabolic process (GO:0044238)	7199	503	409.44	+	1.23	4.34E-08	7.60E-06	no
metabolic process (GO:0008152)	8105	565	460.97	+	1.23	2.43E-09	6.22E-07	no
protein metabolic process (GO:0019538)	3867	269	219.94	+	1.22	4.89E-04	1.77E-02	no
regulation of biological process (GO:0050789)	11772	816	669.53	+	1.22	1.16E-17	4.52E-14	no
organic substance metabolic process (GO:0071704)	7694	526	437.6	+	1.2	3.05E-07	3.77E-05	no

organonitrogen compound metabolic process (GO:1901564)	4908	333	279.14	+	1.19	4.38E-04	1.64E-02	no
biological regulation (GO:0065007)	12190	827	693.31	+	1.19	3.42E-15	2.81E-12	no
cell communication (GO:0007154)	5244	353	298.25	+	1.18	4.52E-04	1.68E-02	no
signal transduction (GO:0007165)	4783	320	272.03	+	1.18	1.59E-03	4.84E-02	no
signaling (GO:0023052)	5081	339	288.98	+	1.17	1.23E-03	3.91E-02	no
cellular process (GO:0009987)	14600	953	830.38	+	1.15	1.35E-15	1.50E-12	no
biological_process (GO:0008150)	17752	1100	1009.65	+	1.09	1.74E-16	3.40E-13	no
nervous system process (GO:0050877)	1460	51	83.04	-	0.61	1.88E-04	8.10E-03	no
G protein-coupled receptor signaling pathway (GO:0007186)	1230	32	69.96	-	0.46	6.22E-07	6.52E-05	no
sensory perception (GO:0007600)	995	25	56.59	-	0.44	4.58E-06	3.74E-04	no
Unclassified (UNCLASSIFIED)	2837	71	161.35	-	0.44	1.74E-16	3.02E-13	no
detection of stimulus (GO:0051606)	686	11	39.02	-	0.28	3.36E-07	4.03E-05	no
detection of chemical stimulus (GO:0009593)	524	5	29.8	-	0.17	7.71E-08	1.23E-05	no
sensory perception of chemical stimulus (GO:0007606)	549	4	31.22	-	0.13	2.83E-09	6.91E-07	no
detection of stimulus involved in sensory perception (GO:0050906)	565	4	32.13	-	0.12	1.36E-09	3.66E-07	no
sensory perception of smell (GO:0007608)	470	3	26.73	-	0.11	2.30E-08	4.54E-06	no
cilium-dependent cell motility (GO:0060285)	136	0	7.74	-	< 0.01	8.98E-04	2.98E-02	no
cilium or flagellum-dependent cell motility (GO:0001539)	136	0	7.74	-	< 0.01	8.98E-04	2.98E-02	no
detection of chemical stimulus involved in sensory perception of smell (GO:0050911)	443	0	25.2	-	< 0.01	3.20E-11	1.22E-08	no
detection of chemical stimulus involved in sensory perception (GO:0050907)	491	0	27.93	-	< 0.01	1.89E-12	8.43E-10	no
