# Supplementary material for: Transcriptomic and functional analysis of ANGPTL4 overexpression in pancreatic cancer nominates targets that reverse chemoresistance
Source: BMC Cancer. 2023 Jun 8;23:524. doi: 10.1186/s12885-023-11010-1 (PMC10251551; doi:10.1186/s12885-023-11010-1)
Supplement: Supplementary file 8 — Additional file 8: Table S6.txt [file 12885_2023_11010_MOESM8_ESM.pdf]

Supplementary Table 6: List of 42 DEG genes for both ANGPTL4 expression and gemcitabine

HGNC Symbol
MYH16
KDM7A
PLAUR
MVP
CHDH
WWTR1
RNH1
ALAS1
FAM76B
SMARCA2
CXCL2
ATP8B1
EPS15
SMOX
OAS1
TBC1D2
TTC28
XBP1
APOL1
ARHGAP5
NFKBIA
PYGB
SLCO4A1
PTK6
TRIB3
DNTTIP1
AMMECR1
LYRM1
CRISPLD2
SFRP1
CDK6
OGDH
DNAJB6
PTPRZ1
SERPINE1
DDX58
PDLIM1
GATA3
DNMBP
STN1
CSF3

PNPO

resistance
