# Supplementary material for: Transcriptomic and functional analysis of ANGPTL4 overexpression in pancreatic cancer nominates targets that reverse chemoresistance
Source: BMC Cancer. 2023 Jun 8;23:524. doi: 10.1186/s12885-023-11010-1 (PMC10251551; doi:10.1186/s12885-023-11010-1)
Supplement: Supplementary file 9 — Additional file 9: Table S7.txt [file 12885_2023_11010_MOESM9_ESM.pdf]

Supplementary Table 7: Correlation analysis of TCGA-PAAD data using DEG gene list from MP2_ANGPTL4_OE vs MP2_ANGPTL4_KD analysis with or without gemcitabine treatment

Gemcitabine treatment	row	column	correaltion (r^2)	pvalue	row	column	correaltion (r^2)	pvalue
yes	ANGPTL4	CLCF1	0.482	9.27E-12	CLCF1	Survival	-0.379	1.82E-07
yes	ANGPTL4	TGM2	0.475	2.10E-11	TGM2	Survival	-0.325	9.40E-06
yes	ANGPTL4	CEBPB	0.474	2.38E-11	CEBPB	Survival	-0.329	7.55E-06
yes	ANGPTL4	PLAU	0.454	2.03E-10	PLAU	Survival	-0.348	1.91E-06
yes	ANGPTL4	PLAUR	0.386	9.90E-08	PLAUR	Survival	-0.318	1.53E-05
yes	ANGPTL4	APOL1	0.372	3.10E-07	APOL1	Survival	-0.287	0.00010078
yes	ANGPTL4	CD44	0.364	5.80E-07	CD44	Survival	-0.373	3.02E-07
yes	ANGPTL4	BHLHE40	0.363	6.42E-07	BHLHE40	Survival	-0.319	1.39E-05
yes	ANGPTL4	ITGB4	0.359	8.63E-07	ITGB4	Survival	-0.297	5.80E-05
yes	ANGPTL4	ARNTL2	0.357	1.00E-06	ARNTL2	Survival	-0.280	0.00015296
yes	ANGPTL4	RAP2B	0.352	1.50E-06	RAP2B	Survival	-0.293	7.37E-05
yes	ANGPTL4	NT5E	0.339	3.65E-06	NT5E	Survival	-0.295	6.40E-05
yes	ANGPTL4	UBASH3B	0.335	4.76E-06	UBASH3B	Survival	-0.300	4.63E-05
yes	ANGPTL4	TM4SF1	0.335	4.99E-06	TM4SF1	Survival	-0.287	0.00010357
yes	ANGPTL4	RALB	0.335	5.02E-06	RALB	Survival	-0.382	1.44E-07
no	ANGPTL4	DDIT4	0.534	1.60E-14	DDIT4	Survival	-0.352	1.48E-06
no	ANGPTL4	CMTM7	0.481	1.05E-11	CMTM7	Survival	-0.360	7.93E-07
no	ANGPTL4	TGM2	0.475	2.10E-11	TGM2	Survival	-0.325	9.40E-06
no	ANGPTL4	CEBPB	0.474	2.38E-11	CEBPB	Survival	-0.329	7.55E-06
no	ANGPTL4	PLAUR	0.386	9.90E-08	PLAUR	Survival	-0.318	1.53E-05
no	ANGPTL4	APOL1	0.372	3.10E-07	APOL1	Survival	-0.287	0.00010078
no	ANGPTL4	GJB3	0.372	3.18E-07	GJB3	Survival	-0.353	1.37E-06
no	ANGPTL4	CD44	0.364	5.80E-07	CD44	Survival	-0.373	3.02E-07
no	ANGPTL4	ITGB4	0.359	8.63E-07	ITGB4	Survival	-0.297	5.80E-05
no	ANGPTL4	RHOF	0.354	1.21E-06	RHOF	Survival	-0.414	9.03E-09
no	ANGPTL4	CAV2	0.354	1.22E-06	CAV2	Survival	-0.340	3.45E-06
no	ANGPTL4	RAP2B	0.352	1.50E-06	RAP2B	Survival	-0.293	7.37E-05
no	ANGPTL4	NT5E	0.339	3.65E-06	NT5E	Survival	-0.295	6.40E-05
no	ANGPTL4	TM4SF1	0.335	4.99E-06	TM4SF1	Survival	-0.287	0.00010357
no	ANGPTL4	RALB	0.335	5.02E-06	RALB	Survival	-0.382	1.44E-07
no	ANGPTL4	SDC4	0.334	5.04E-06	SDC4	Survival	-0.304	3.64E-05
