# Supplementary material for: Transcriptomic and functional analysis of ANGPTL4 overexpression in pancreatic cancer nominates targets that reverse chemoresistance
Source: BMC Cancer. 2023 Jun 8;23:524. doi: 10.1186/s12885-023-11010-1 (PMC10251551; doi:10.1186/s12885-023-11010-1)
Supplement: Supplementary file 10 — Additional file 10: Table S8.txt [file 12885_2023_11010_MOESM10_ESM.pdf]

Supplementary Table 8: Pathway analysis results from DEG from MP2_ANGPTL4_OE vs MP2_ANGPTL4_KD analysis

HGNC Symbol	Ensembl Gene	log2 FoldChange	padj	RTK	Notch	PIK3K-AKT	MAPK	RAS	MTOR	Extra Cellular Matrix	Angiogenesis	NFKB	TGFB	TNF SIGNALING	HIF1	Description
ENSG00000164692	COL1A2	1.05174507	0.00608569			X				X						collagen type I alpha 2 chain [Source:HGNC Symbol;Acc:HGNC:2198]
ENSG00000163359	COL6A3	1.06215621	5.93E-07			X				X						collagen type VI alpha 3 chain [Source:HGNC Symbol;Acc:HGNC:2213]
ENSG00000146648	EGFR	1.29021197	6.54E-40			X	X	X		X					X	epidermal growth factor receptor [Source:HGNC Symbol;Acc:HGNC:3236]
ENSG00000145675	PIK3R1	-0.8208581	0.04941779			X		X	X	X				X	X	phosphoinositide-3-kinase regulatory subunit 1 [Source:HGNC Symbol;Acc:HGNC:8979]
ENSG00000142173	COL6A2	0.9527075	0.01322492			X				X						collagen type VI alpha 2 chain [Source:HGNC Symbol;Acc:HGNC:2212]
ENSG00000138685	FGF2	1.64001621	2.52E-17			X	X	X		X						fibroblast growth factor 2 [Source:HGNC Symbol;Acc:HGNC:3676]
ENSG00000132470	ITGB4	2.16782864	1.51E-35			X				X						integrin subunit beta 4 [Source:HGNC Symbol;Acc:HGNC:6158]
ENSG00000126934	MAP2K2	1.08827795	1.58E-15			X	X	X	X	X					X	mitogen-activated protein kinase kinase 2 [Source:HGNC Symbol;Acc:HGNC:6842]
ENSG00000118971	CCND2	2.45910658	4.08E-06			X				X						cyclin D2 [Source:HGNC Symbol;Acc:HGNC:1583]
ENSG00000112715	VEGFA	1.00958653	4.83E-09	x		X	X	X		X					X	vascular endothelial growth factor A [Source:HGNC Symbol;Acc:HGNC:12680]
ENSG00000112576	CCND3	-0.7815831	0.01309118			X				X						cyclin D3 [Source:HGNC Symbol;Acc:HGNC:1585]
ENSG00000247596	TWF2	1.00044919	5.03E-06							X						twinfilin actin binding protein 2 [Source:HGNC Symbol;Acc:HGNC:9621]
ENSG00000242498	ARPIN	1.37539404	0.00308745							X						actin related protein 2/3 complex inhibitor [Source:HGNC Symbol;Acc:HGNC:28782]
ENSG00000241685	ARPC1A	0.7700163	3.48E-05							X						actin related protein 2/3 complex subunit 1A [Source:HGNC Symbol;Acc:HGNC:703]
ENSG00000197324	LRP10	0.86561696	1.63E-06							X						LDL receptor related protein 10 [Source:HGNC Symbol;Acc:HGNC:14553]
ENSG00000196914	ARHGEF12	0.87362959	4.12E-10							X						Rho guanine nucleotide exchange factor 12 [Source:HGNC Symbol;Acc:HGNC:14193]
ENSG00000196526	AFAP1	1.93406149	6.30E-27							X						actin filament associated protein 1 [Source:HGNC Symbol;Acc:HGNC:24017]
ENSG00000189143	CLDN4	0.88738427	3.31E-09							X						claudin 4 [Source:HGNC Symbol;Acc:HGNC:2046]
ENSG00000183856	IQGAP3	-0.7473013	0.00343476							X						IQ motif containing GTPase activating protein 3 [Source:HGNC Symbol;Acc:HGNC:20669]
ENSG00000181929	PRKAG1	0.7203521	0.00311602							X	X					protein kinase AMP-activated non-catalytic subunit gamma 1 [Source:HGNC Symbol;Acc:HGNC:9385]
ENSG00000177606	JUN	1.46533239	2.60E-76				X			X				X		Jun proto-oncogene, AP-1 transcription factor subunit [Source:HGNC Symbol;Acc:HGNC:6204]
ENSG00000166333	ILK	0.82609866	0.00065069							X						integrin linked kinase [Source:HGNC Symbol;Acc:HGNC:6040]
ENSG00000163347	CLDN1	2.30926492	1.27E-40							X						claudin 1 [Source:HGNC Symbol;Acc:HGNC:2032]
ENSG00000162733	DDR2	1.24715432	8.01E-12	x						X						discoidin domain receptor tyrosine kinase 2 [Source:HGNC Symbol;Acc:HGNC:2731]
ENSG00000151491	EPS8	2.07406874	2.22E-10							X						epidermal growth factor receptor pathway substrate 8 [Source:HGNC Symbol;Acc:HGNC:3420]
ENSG00000148180	GSN	1.65833359	5.18E-18							X						gelsolin [Source:HGNC Symbol;Acc:HGNC:4620]
ENSG00000145703	IQGAP2	-0.8222412	0.00806923							X						IQ motif containing GTPase activating protein 2 [Source:HGNC Symbol;Acc:HGNC:6111]
ENSG00000143621	ILF2	-0.9182398	7.17E-14							X						interleukin enhancer binding factor 2 [Source:HGNC Symbol;Acc:HGNC:6037]
ENSG00000142798	HSPG2	1.3060925	5.44E-07							X						heparan sulfate proteoglycan 2 [Source:HGNC Symbol;Acc:HGNC:5273]
ENSG00000142330	CAPN10	-0.8837466	0.07913583							X						calpain 10 [Source:HGNC Symbol;Acc:HGNC:1477]
ENSG00000139734	DIAPH3	-0.9810457	2.83E-16							X						diaphanous related formin 3 [Source:HGNC Symbol;Acc:HGNC:15480]
ENSG00000139725	RHOF	1.03585557	2.76E-05							X						ras homolog family member F, filopodia associated [Source:HGNC Symbol;Acc:HGNC:15703]
ENSG00000136943	CTSV	-0.9850849	0.02468769							X						cathepsin V [Source:HGNC Symbol;Acc:HGNC:2538]
ENSG00000132646	PCNA	-0.857796	9.40E-06							X						proliferating cell nuclear antigen [Source:HGNC Symbol;Acc:HGNC:8729]
ENSG00000124145	SDC4	1.0208095	5.76E-14							X						syndecan 4 [Source:HGNC Symbol;Acc:HGNC:10661]
ENSG00000120708	TGFBI	0.82204711	0.00051109							X						transforming growth factor beta induced [Source:HGNC Symbol;Acc:HGNC:11771]
ENSG00000117984	CTSD	1.40793211	6.23E-25							X						cathepsin D [Source:HGNC Symbol;Acc:HGNC:2529]
ENSG00000116584	ARHGEF2	0.75720292	9.98E-05							X						Rho/Rac guanine nucleotide exchange factor 2 [Source:HGNC Symbol;Acc:HGNC:682]
ENSG00000115594	IL1R1	0.97733147	0.01090607				X			X		X				interleukin 1 receptor type 1 [Source:HGNC Symbol;Acc:HGNC:5993]
ENSG00000111725	PRKAB1	1.26260234	0.00432138							X	X					protein kinase AMP-activated non-catalytic subunit beta 1 [Source:HGNC Symbol;Acc:HGNC:9378]
ENSG00000110330	BIRC2	1.15897148	6.68E-20							X		X		X		baculoviral IAP repeat containing 2 [Source:HGNC Symbol;Acc:HGNC:590]
ENSG00000106366	SERPINE1	1.71793858	3.01E-17							X					X	serpin family E member 1 [Source:HGNC Symbol;Acc:HGNC:8583]
ENSG00000105971	CAV2	1.11343361	1.27E-06							X						caveolin 2 [Source:HGNC Symbol;Acc:HGNC:1528]
ENSG00000105329	TGFB1	1.14053495	0.0038067				X			X			X			transforming growth factor beta 1 [Source:HGNC Symbol;Acc:HGNC:11766]
ENSG00000100852	ARHGAP5	1.6313948	9.59E-53							X						Rho GTPase activating protein 5 [Source:HGNC Symbol;Acc:HGNC:675]
ENSG00000085832	EPS15	0.79988838	5.40E-07							X						epidermal growth factor receptor pathway substrate 15 [Source:HGNC Symbol;Acc:HGNC:3419]
ENSG00000084112	SSH1	0.77249691	0.000153							X						slingshot protein phosphatase 1 [Source:HGNC Symbol;Acc:HGNC:30579]
ENSG00000080503	SMARCA2	1.96671123	9.82E-08							X						SWI/SNF related, matrix associated, actin dependent regulator of chromatin, subfamily a, member 2 [Source:HGNC Symbol;Acc:HGNC:11098]

ENSG00000075711	DLG1	1.56519072	3.51E-52							X						discs large MAGUK scaffold protein 1 [Source:HGNC Symbol;Acc:HGNC:2900]
ENSG00000075618	FSCN1	1.8429407	6.06E-32							X						fascin actin-bundling protein 1 [Source:HGNC Symbol;Acc:HGNC:11148]
ENSG00000072571	HMMR	-1.0431829	8.25E-29							X						hyaluronan mediated motility receptor [Source:HGNC Symbol;Acc:HGNC:5012]
ENSG00000069702	TGFBR3	1.31130991	3.73E-06							X						transforming growth factor beta receptor 3 [Source:HGNC Symbol;Acc:HGNC:11774]
ENSG00000050820	BCAR1	0.89241441	2.09E-05							X						BCAR1 scaffold protein, Cas family member [Source:HGNC Symbol;Acc:HGNC:971]
ENSG00000042493	CAPG	1.40020523	2.27E-11							X						capping actin protein, gelsolin like [Source:HGNC Symbol;Acc:HGNC:1474]
ENSG00000026508	CD44	0.97128601	2.21E-36							X						CD44 molecule (Indian blood group) [Source:HGNC Symbol;Acc:HGNC:1681]
ENSG00000023445	BIRC3	2.29088949	3.59E-72							X		X		X		baculoviral IAP repeat containing 3 [Source:HGNC Symbol;Acc:HGNC:591]
ENSG00000077782	FGFR1	-1.1990805	4.42E-13	x		X	X	X		X						fibroblast growth factor receptor 1 [Source:HGNC Symbol;Acc:HGNC:3688]
ENSG00000072682	P4HA2	-1.2010348	2.53E-07							X						prolyl 4-hydroxylase subunit alpha 2 [Source:HGNC Symbol;Acc:HGNC:8547]
ENSG00000074211	PPP2R2C	-1.431944	5.67E-12			X				X						protein phosphatase 2 regulatory subunit Bgamma [Source:HGNC Symbol;Acc:HGNC:9306]
ENSG00000181104	F2R	-1.5238348	1.52E-30			X				X						coagulation factor II thrombin receptor [Source:HGNC Symbol;Acc:HGNC:3537]
ENSG00000142875	PRKACB	-1.5719502	3.49E-06				X	X		X						protein kinase cAMP-activated catalytic subunit beta [Source:HGNC Symbol;Acc:HGNC:9381]
ENSG00000164171	ITGA2	-1.7871498	0.00103898			X				X						integrin subunit alpha 2 [Source:HGNC Symbol;Acc:HGNC:6137]
ENSG00000169306	IL1RAPL1	-2.0239219	1.50E-05							X						interleukin 1 receptor accessory protein like 1 [Source:HGNC Symbol;Acc:HGNC:5996]
ENSG00000196611	MMP1	-2.1998129	6.26E-05							X						matrix metallopeptidase 1 [Source:HGNC Symbol;Acc:HGNC:7155]
ENSG00000108342	CSF3	3.0665071	7.25E-29	x		X										colony stimulating factor 3 [Source:HGNC Symbol;Acc:HGNC:2438]
ENSG00000105810	CDK6	2.97163644	5.23E-53			X										cyclin dependent kinase 6 [Source:HGNC Symbol;Acc:HGNC:1777]
ENSG00000118515	SGK1	2.27112501	1.86E-62			X			X							serum/glucocorticoid regulated kinase 1 [Source:HGNC Symbol;Acc:HGNC:10810]
ENSG00000171552	BCL2L1	1.53907412	1.08E-07			X		X				X				BCL2 like 1 [Source:HGNC Symbol;Acc:HGNC:992]
ENSG00000171517	LPAR3	1.39467386	1.85E-06			X										lysophosphatidic acid receptor 3 [Source:HGNC Symbol;Acc:HGNC:14298]
ENSG00000168209	DDIT4	1.06477855	2.92E-06			X										DNA damage inducible transcript 4 [Source:HGNC Symbol;Acc:HGNC:24944]
ENSG00000167965	MLST8	1.01322785	0.02454895			X			X		X					MTOR associated protein, LST8 homolog [Source:HGNC Symbol;Acc:HGNC:24825]
ENSG00000159110	IFNAR2	0.90591756	0.00442067			X										interferon alpha and beta receptor subunit 2 [Source:HGNC Symbol;Acc:HGNC:5433]
ENSG00000128272	ATF4	0.88624762	4.17E-09			X	X							X		activating transcription factor 4 [Source:HGNC Symbol;Acc:HGNC:786]
ENSG00000118689	FOXO3	0.75228084	0.00454049			X										forkhead box O3 [Source:HGNC Symbol;Acc:HGNC:3821]
ENSG00000106615	RHEB	-0.7014471	0.00508062			X			X		X					Ras homolog, mTORC1 binding [Source:HGNC Symbol;Acc:HGNC:10011]
ENSG00000080824	HSP90AA1	-1.0018819	1.15E-52			X					X					heat shock protein 90 alpha family class A member 1 [Source:HGNC Symbol;Acc:HGNC:5253]
ENSG00000109321	AREG	-1.1116935	6.40E-22			X	X									amphiregulin [Source:HGNC Symbol;Acc:HGNC:651]
ENSG00000243364	EFNA4	-1.3153421	0.00651192			X	X	X								ephrin A4 [Source:HGNC Symbol;Acc:HGNC:3224]
ENSG00000184349	EFNA5	-1.6461496	2.96E-11			X	X	X								ephrin A5 [Source:HGNC Symbol;Acc:HGNC:3225]
ENSG00000081041	CXCL2	3.57312971	2.35E-14									X		X		C-X-C motif chemokine ligand 2 [Source:HGNC Symbol;Acc:HGNC:4603]
ENSG00000169429	CXCL8	3.06218352	9.22E-12									X				C-X-C motif chemokine ligand 8 [Source:HGNC Symbol;Acc:HGNC:6025]
ENSG00000163739	CXCL1	2.83695335	7.47E-36									X		X		C-X-C motif chemokine ligand 1 [Source:HGNC Symbol;Acc:HGNC:4602]
ENSG00000118503	TNFAIP3	1.88124069	1.82E-06									X		X		TNF alpha induced protein 3 [Source:HGNC Symbol;Acc:HGNC:11896]
ENSG00000116717	GADD45A	1.47231859	1.58E-45				X					X				growth arrest and DNA damage inducible alpha [Source:HGNC Symbol;Acc:HGNC:4095]
ENSG00000104856	RELB	1.44412326	6.27E-09				X					X				RELB proto-oncogene, NF-kB subunit [Source:HGNC Symbol;Acc:HGNC:9956]
ENSG00000197943	PLCG2	1.3911277	0.00356793					X				X			X	phospholipase C gamma 2 [Source:HGNC Symbol;Acc:HGNC:9066]
ENSG00000056558	TRAF1	1.01878692	0.03628074									X		X		TNF receptor associated factor 1 [Source:HGNC Symbol;Acc:HGNC:12031]
ENSG00000121060	TRIM25	0.91804367	1.08E-12									X				tripartite motif containing 25 [Source:HGNC Symbol;Acc:HGNC:12932]
ENSG00000100906	NFKBIA	0.8222283	8.22E-11									X		X		NFKB inhibitor alpha [Source:HGNC Symbol;Acc:HGNC:7797]
ENSG00000111321	LTBR	0.80203738	0.00016421									X			X	lymphotoxin beta receptor [Source:HGNC Symbol;Acc:HGNC:6718]
ENSG00000149311	ATM	0.73192289	3.25E-05								X	X				ATM serine/threonine kinase [Source:HGNC Symbol;Acc:HGNC:795]
ENSG00000143799	PARP1	-0.7575778	3.98E-12									X				poly(ADP-ribose) polymerase 1 [Source:HGNC Symbol;Acc:HGNC:270]
ENSG00000071242	RPS6KA2	2.15943903	5.01E-06				X		X							ribosomal protein S6 kinase A2 [Source:HGNC Symbol;Acc:HGNC:10431]
ENSG00000130766	SESN2	1.8209808	9.32E-37						X							sestrin 2 [Source:HGNC Symbol;Acc:HGNC:20746]
ENSG00000174804	FZD4	1.74620214	7.34E-05						X							frizzled class receptor 4 [Source:HGNC Symbol;Acc:HGNC:4042]
ENSG00000103257	SLC7A5	1.30985335	7.00E-17						X							solute carrier family 7 member 5 [Source:HGNC Symbol;Acc:HGNC:11063]
ENSG00000052795	FNIP2	1.11927327	0.01139713						X							folliculin interacting protein 2 [Source:HGNC Symbol;Acc:HGNC:29280]
ENSG00000106070	GRB10	1.03315855	1.10E-06						X							growth factor receptor bound protein 10 [Source:HGNC Symbol;Acc:HGNC:4564]

ENSG00000130779	CLIP1	0.99280546	1.19E-29						X							CAP-Gly domain containing linker protein 1 [Source:HGNC Symbol;Acc:HGNC:10461]
ENSG00000239282	CASTOR1	0.85109626	0.04289612						X							cytosolic arginine sensor for mTORC1 subunit 1 [Source:HGNC Symbol;Acc:HGNC:34423]
ENSG00000168003	SLC3A2	0.80537299	3.22E-07						X							solute carrier family 3 member 2 [Source:HGNC Symbol;Acc:HGNC:11026]
ENSG00000145604	SKP2	-0.9049038	0.00070327						X							S-phase kinase associated protein 2 [Source:HGNC Symbol;Acc:HGNC:10901]
ENSG00000100726	TELO2	-1.2538611	0.02246188						X							telomere maintenance 2 [Source:HGNC Symbol;Acc:HGNC:29099]
ENSG00000145358	DDIT4L	-2.6130451	1.53E-08						X							DNA damage inducible transcript 4 like [Source:HGNC Symbol;Acc:HGNC:30555]
ENSG00000198925	ATG9A	0.98236548	0.00012216								X					autophagy related 9A [Source:HGNC Symbol;Acc:HGNC:22408]
ENSG00000167987	VPS37C	0.72807486	0.02884073								X					VPS37C subunit of ESCRT-I [Source:HGNC Symbol;Acc:HGNC:26097]
ENSG00000164741	DLC1	-0.8376075	0.04527561								X					DLC1 Rho GTPase activating protein [Source:HGNC Symbol;Acc:HGNC:2897]
ENSG00000158828	PINK1	0.7592507	0.00119544								X					PTEN induced kinase 1 [Source:HGNC Symbol;Acc:HGNC:14581]
ENSG00000140941	MAP1LC3B	0.95591442	8.69E-14								X					microtubule associated protein 1 light chain 3 beta [Source:HGNC Symbol;Acc:HGNC:13352]
ENSG00000139112	GABARAPL1	1.30417222	2.34E-06								X					GABA type A receptor associated protein like 1 [Source:HGNC Symbol;Acc:HGNC:4068]
ENSG00000137285	TUBB2B	3.16751393	1.97E-12								X					tubulin beta 2B class IIb [Source:HGNC Symbol;Acc:HGNC:30829]
ENSG00000110497	AMBRA1	1.43453185	1.79E-05								X					autophagy and beclin 1 regulator 1 [Source:HGNC Symbol;Acc:HGNC:25990]
ENSG00000109971	HSPA8	-0.8176342	2.50E-14				X				X					heat shock protein family A (Hsp70) member 8 [Source:HGNC Symbol;Acc:HGNC:5241]
ENSG00000074319	TSG101	0.91985685	2.74E-06								X					tumor susceptibility 101 [Source:HGNC Symbol;Acc:HGNC:15971]
ENSG00000032742	IFT88	-1.1159913	7.30E-05								X					intraflagellar transport 88 [Source:HGNC Symbol;Acc:HGNC:20606]
ENSG00000204388	HSPA1B	-1.5535942	2.67E-07				X									heat shock protein family A (Hsp70) member 1B [Source:HGNC Symbol;Acc:HGNC:5233]
ENSG00000175197	DDIT3	1.30934941	2.16E-09				X									DNA damage inducible transcript 3 [Source:HGNC Symbol;Acc:HGNC:2726]
ENSG00000170345	FOS	0.96670504	0.02059999				X							X		Fos proto-oncogene, AP-1 transcription factor subunit [Source:HGNC Symbol;Acc:HGNC:3796]
ENSG00000142408	CACNG8	0.70087874	6.41E-05				X									calcium voltage-gated channel auxiliary subunit gamma 8 [Source:HGNC Symbol;Acc:HGNC:13628]
ENSG00000139318	DUSP6	-2.0689486	6.60E-49				X									dual specificity phosphatase 6 [Source:HGNC Symbol;Acc:HGNC:3072]
ENSG00000138166	DUSP5	3.09561866	4.70E-09				X									dual specificity phosphatase 5 [Source:HGNC Symbol;Acc:HGNC:3071]
ENSG00000131196	NFATC1	-0.7546608	0.01035181				X									nuclear factor of activated T cells 1 [Source:HGNC Symbol;Acc:HGNC:7775]
ENSG00000075461	CACNG4	-1.7865064	3.25E-05				X									calcium voltage-gated channel auxiliary subunit gamma 4 [Source:HGNC Symbol;Acc:HGNC:1408]
ENSG00000169733	RFNG	0.73737487	0.00043724		X											RFNG O-fucosylpeptide 3-beta-N-acetylglucosaminyltransferase [Source:HGNC Symbol;Acc:HGNC:9974]
ENSG00000163840	DTX3L	1.07497	1.42E-14		X											deltex E3 ubiquitin ligase 3L [Source:HGNC Symbol;Acc:HGNC:30323]
ENSG00000148400	NOTCH1	-0.9986385	0.00200746		X											notch receptor 1 [Source:HGNC Symbol;Acc:HGNC:7881]
ENSG00000143801	PSEN2	-0.8661833	0.03142169		X											presenilin 2 [Source:HGNC Symbol;Acc:HGNC:9509]
ENSG00000134250	NOTCH2	-0.7006488	0.00051098		X											notch receptor 2 [Source:HGNC Symbol;Acc:HGNC:7882]
ENSG00000100342	APOL1	2.48116154	1.71E-07		X											apolipoprotein L1 [Source:HGNC Symbol;Acc:HGNC:618]
ENSG00000204217	BMPR2	0.84863886	4.79E-05										X			bone morphogenetic protein receptor type 2 [Source:HGNC Symbol;Acc:HGNC:1078]
ENSG00000198742	SMURF1	1.35528098	6.05E-08										X			SMAD specific E3 ubiquitin protein ligase 1 [Source:HGNC Symbol;Acc:HGNC:16807]
ENSG00000115738	ID2	-1.0154861	0.00249446										X			inhibitor of DNA binding 2 [Source:HGNC Symbol;Acc:HGNC:5361]
ENSG00000113658	SMAD5	-0.7429178	1.19E-10										X			SMAD family member 5 [Source:HGNC Symbol;Acc:HGNC:6771]
ENSG00000113558	SKP1	-0.7194287	8.62E-16										X			S-phase kinase associated protein 1 [Source:HGNC Symbol;Acc:HGNC:10899]
ENSG00000100387	RBX1	-0.7802642	3.70E-05										X		X	ring-box 1 [Source:HGNC Symbol;Acc:HGNC:9928]
ENSG00000271503	CCL5	4.27573986	7.14E-15											X		C-C motif chemokine ligand 5 [Source:HGNC Symbol;Acc:HGNC:10632]
ENSG00000172216	CEBPB	1.24574732	5.54E-09											X		CCAAT enhancer binding protein beta [Source:HGNC Symbol;Acc:HGNC:1834]
ENSG00000168404	MLKL	1.44577841	0.00012485											X		mixed lineage kinase domain like pseudokinase [Source:HGNC Symbol;Acc:HGNC:26617]
ENSG00000163735	CXCL5	2.69800517	5.33E-42											X		C-X-C motif chemokine ligand 5 [Source:HGNC Symbol;Acc:HGNC:10642]
ENSG00000125347	IRF1	0.94828505	1.33E-06											X		interferon regulatory factor 1 [Source:HGNC Symbol;Acc:HGNC:6116]
ENSG00000165527	ARF6	0.82372665	4.87E-14					X								ADP ribosylation factor 6 [Source:HGNC Symbol;Acc:HGNC:659]
ENSG00000144118	RALB	0.9491103	6.07E-11					X								RAS like proto-oncogene B [Source:HGNC Symbol;Acc:HGNC:9840]
ENSG00000134954	ETS1	1.19167042	1.16E-09					X								ETS proto-oncogene 1, transcription factor [Source:HGNC Symbol;Acc:HGNC:3488]
ENSG00000075391	RASAL2	-2.1760086	1.94E-31					X								RAS protein activator like 2 [Source:HGNC Symbol;Acc:HGNC:9874]
ENSG00000113070	HBEGF	1.64130511	6.42E-17	x												heparin binding EGF like growth factor [Source:HGNC Symbol;Acc:HGNC:3059]
ENSG00000168610	STAT3	0.97476839	6.71E-09												X	signal transducer and activator of transcription 3 [Source:HGNC Symbol;Acc:HGNC:11364]
ENSG00000156510	HKDC1	-2.5992454	6.74E-38												X	hexokinase domain containing 1 [Source:HGNC Symbol;Acc:HGNC:23302]
ENSG00000072274	TFRC	-0.739833	1.68E-07												X	transferrin receptor [Source:HGNC Symbol;Acc:HGNC:11763]
